# Circulating tumour DNA analysis to direct therapy in advanced breast cancer (plasmaMATCH): a multicentre, multicohort, phase 2a, platform trial

**DOI:** 10.1016/S1470-2045(20)30444-7

**Published:** 2020-10

**Authors:** Nicholas C Turner, Belinda Kingston, Lucy S Kilburn, Sarah Kernaghan, Andrew M Wardley, Iain R Macpherson, Richard D Baird, Rebecca Roylance, Peter Stephens, Olga Oikonomidou, Jeremy P Braybrooke, Mark Tuthill, Jacinta Abraham, Matthew C Winter, Hannah Bye, Michael Hubank, Heidrun Gevensleben, Ros Cutts, Claire Snowdon, Daniel Rea, David Cameron, Abeer Shaaban, Katrina Randle, Sue Martin, Katie Wilkinson, Laura Moretti, Judith M Bliss, Alistair Ring

**Affiliations:** a Breast Cancer Now Toby Robins Research Centre, Institute of Cancer Research, London, UK; b Clinical Trials and Statistics Unit, Institute of Cancer Research, London, UK; c National Institute for Health Research Centre for Molecular Pathology, Institute of Cancer Research, London, UK; d Breast Unit, Royal Marsden National Health Service (NHS) Foundation Trust, London, UK; e National Institute for Health Research Manchester Clinical Research Facility, Christie NHS Foundation Trust, Manchester, UK; f Division of Cancer Sciences, School of Medical Sciences, Faculty of Biology Medicine & Health, University of Manchester, Manchester, UK; g Institute of Cancer Sciences, University of Glasgow, Glasgow, UK; h Cancer Research UK Cambridge Centre, Cambridge, UK; i University College London Hospitals NHS Foundation Trust, London, UK; j National Institute for Health Research University College London Hospitals Biomedical Research Centre, London, UK; k Royal Devon and Exeter NHS Foundation Trust, Exeter, UK; l Cancer Research UK Edinburgh Centre, Edinburgh Cancer Centre, Western General Hospital, University of Edinburgh, Edinburgh, UK; m University Hospitals Bristol NHS Foundation Trust, Bristol, UK; n Oxford University Hospitals NHS Foundation Trust, Oxford, UK; o Velindre University NHS Trust, Cardiff, UK; p Weston Park Hospital, Sheffield Teaching Hospitals NHS Foundation Trust, Sheffield, UK; q Royal Marsden NHS Foundation Trust, Sutton, UK; r Institute of Pathology, Bonn University Hospital, Bonn, Germany; s University Hospitals Birmingham NHS Foundation Trust, Birmingham, UK; t Independent Cancer Patients' Voice, London, UK

## Abstract

**Background:**

Circulating tumour DNA (ctDNA) testing might provide a current assessment of the genomic profile of advanced cancer, without the need to repeat tumour biopsy. We aimed to assess the accuracy of ctDNA testing in advanced breast cancer and the ability of ctDNA testing to select patients for mutation-directed therapy.

**Methods:**

We did an open-label, multicohort, phase 2a, platform trial of ctDNA testing in 18 UK hospitals. Participants were women (aged ≥18 years) with histologically confirmed advanced breast cancer and an Eastern Cooperative Oncology Group performance status 0–2. Patients had completed at least one previous line of treatment for advanced breast cancer or relapsed within 12 months of neoadjuvant or adjuvant chemotherapy. Patients were recruited into four parallel treatment cohorts matched to mutations identified in ctDNA: cohort A comprised patients with *ESR1* mutations (treated with intramuscular extended-dose fulvestrant 500 mg); cohort B comprised patients with *HER2* mutations (treated with oral neratinib 240 mg, and if oestrogen receptor-positive with intramuscular standard-dose fulvestrant); cohort C comprised patients with *AKT1* mutations and oestrogen receptor-positive cancer (treated with oral capivasertib 400 mg plus intramuscular standard-dose fulvestrant); and cohort D comprised patients with *AKT1* mutations and oestrogen receptor-negative cancer or *PTEN* mutation (treated with oral capivasertib 480 mg). Each cohort had a primary endpoint of confirmed objective response rate. For cohort A, 13 or more responses among 78 evaluable patients were required to infer activity and three or more among 16 were required for cohorts B, C, and D. Recruitment to all cohorts is complete and long-term follow-up is ongoing. This trial is registered with ClinicalTrials.gov, NCT03182634; the European Clinical Trials database, EudraCT2015-003735-36; and the ISRCTN registry, ISRCTN16945804.

**Findings:**

Between Dec 21, 2016, and April 26, 2019, 1051 patients registered for the study, with ctDNA results available for 1034 patients. Agreement between ctDNA digital PCR and targeted sequencing was 96–99% (n=800, kappa 0·89–0·93). Sensitivity of digital PCR ctDNA testing for mutations identified in tissue sequencing was 93% (95% CI 83–98) overall and 98% (87–100) with contemporaneous biopsies. In all cohorts, combined median follow-up was 14·4 months (IQR 7·0–23·7). Cohorts B and C met or exceeded the target number of responses, with five (25% [95% CI 9–49]) of 20 patients in cohort B and four (22% [6–48]) of 18 patients in cohort C having a response. Cohorts A and D did not reach the target number of responses, with six (8% [95% CI 3–17]) of 74 in cohort A and two (11% [1–33]) of 19 patients in cohort D having a response. The most common grade 3–4 adverse events were raised gamma-glutamyltransferase (13 [16%] of 80 patients; cohort A); diarrhoea (four [25%] of 20; cohort B); fatigue (four [22%] of 18; cohort C); and rash (five [26%] of 19; cohort D). 17 serious adverse reactions occurred in 11 patients, and there was one treatment-related death caused by grade 4 dyspnoea (in cohort C).

**Interpretation:**

ctDNA testing offers accurate, rapid genotyping that enables the selection of mutation-directed therapies for patients with breast cancer, with sufficient clinical validity for adoption into routine clinical practice. Our results demonstrate clinically relevant activity of targeted therapies against rare *HER2* and *AKT1* mutations, confirming these mutations could be targetable for breast cancer treatment.

**Funding:**

Cancer Research UK, AstraZeneca, and Puma Biotechnology.

## Introduction

Multiple tumour mutations are potentially targetable for advanced breast cancer treatment. Some of these mutations are common, such as activating *PIK3CA* mutations that are targetable with PI3K inhibitors, including the recently approved alpelisib.^1^ Other potentially targetable mutations, such as *HER2* (also known as *ERBB2*) and *AKT1* mutations, are rare genetic events, occurring in approximately 5% of patients with advanced breast cancer.^2^, ^3^ Nonetheless, these mutations represent attractive therapeutic opportunities, with early-phase studies based on tissue genotyping showing high response rates with matched targeted therapies.^4^, ^5^ Frequently, mutations in the oestrogen receptor (*ESR1), HER2*, and *AKT1* can be acquired in advanced disease, even if not detectable in the archival primary tumour.^2^, ^6^, ^7^ Therefore, archival primary tumour tissue cannot be assumed to be representative of the advanced disease genomic profile.

**Research in context**

**Evidence before this study**

We searched PubMed on June 11, 2020, for clinical trials published between Jan 1, 2000, and Dec 31, 2019, with the terms “circulating tumour DNA”, “cell free DNA”, “plasma DNA”, “liquid biopsy”, and “ctDNA”, with no restriction on language, and identified 212 results. Circulating tumour DNA (ctDNA) analysis in multiple retrospective trials has been shown to accurately genotype mutations found in the tumour. ctDNA analysis therefore has the potential to transform the selection of targeted therapies for patients with advanced cancer. In 2019, the TARGET trial reported a potential role for ctDNA testing in 100 patients with advanced cancers in an early-phase clinical trial setting. However, there has been uncertainty about the validity of ctDNA testing in routine practice, as there have been few large prospective studies to assess the accuracy and utility of ctDNA testing. In addition, sensitivity has not been perfect in previous retrospective studies, suggesting the potential for false negative ctDNA results, and, in routine clinical practice, reflex testing of tumour tissue is advised to confirm negative results. In 2018, the American Society of Clinical Oncology and College of American Pathologists guidelines committee on ctDNA analysis concluded that the absence of prospective trials was one of the major weaknesses in the evidence for bringing ctDNA testing to routine practice, with a need for trials that recruited patients solely on the basis of ctDNA testing without tissue testing beforehand.

**Added value of this study**

plasmaMATCH is, to our knowledge, the first large, prospective, multicentre study assessing the feasibility and clinical utility of ctDNA analysis to direct therapy in patients with advanced breast cancer. We recruited 1051 patients for ctDNA testing, from both academic and general hospitals, and tested with two orthogonal ctDNA analysis techniques. We found high agreement between ctDNA assays, and high sensitivity for mutations identified in tissue sequencing, especially with contemporaneous biopsies. Patients with rare, potentially targetable mutations in *HER2* and *AKT1* in ctDNA had clinically important responses with the HER2 inhibitor neratinib and AKT inhibitor capivasertib, respectively, similar to activity seen in previous tissue sequencing-directed trials. These findings confirm that these mutations are targetable for breast cancer therapy, and demonstrate the validity and clinical utility of using ctDNA testing to screen patients for rare mutations.

**Implications of all the available evidence**

These findings show that ctDNA testing for mutations has sufficient accuracy for widespread adoption in clinical practice, with the assays used. The high sensitivity of ctDNA testing for tissue mutations calls into question the need for reflex tissue testing for negative ctDNA results, within the pretreated metastatic breast cancer patient population studied. This study also shows the potential of a novel liquid biopsy platform to screen for rare oncogenic mutations in breast cancer, and how this approach could transform clinical trials with efficient and rapid mutation screening.

Mutation analysis can be obtained from genomic analysis of tissue-based biopsies from metastatic disease; however, this process is invasive and potentially limited by tumour heterogeneity and temporal tumour evolution.^8^, ^9^ In addition, mutations might not be present at the time of relapse, and only later develop during treatment for advanced cancer, posing a clinical challenge in regards to acquisition of longitudinal tissue biopsies. Highly sensitive assays have been developed in the past 5 years to analyse circulating tumour DNA (ctDNA), which is released into the plasma in small quantities as cancer cells die, providing the opportunity for a scalable, non-invasive approach to profile tumours for somatic mutations.^10^ Retrospective studies show high agreement between ctDNA analysis and tumour tissue-based analysis in patients with advanced cancer.^11^ However, previous prospective studies comparing commercially available ctDNA assays have shown there might be substantial discordance in ctDNA testing results,^12^, ^13^ raising concerns over whether ctDNA testing is ready for widespread clinical adoption.^14^

We aimed to assess the clinical validity of ctDNA testing, and to investigate the clinical utility of using ctDNA to select targeted therapies for patients without previous tissue testing.

## Methods

### Study design and participants

plasmaMATCH is a multicohort, open-label, non-randomised, phase 2a clinical trial platform run across 18 UK hospitals (appendix p 2). Investigators at UK hospitals registered eligible patients with the Institute of Cancer Research Clinical Trials and Statistics Unit (ICR-CTSU) for ctDNA testing. Those with potentially targetable mutations identified in ctDNA testing (*ESR1, HER2, AKT1*, or *PTEN*) were offered entry into one of four parallel treatment cohorts (A–D) according to the mutation identified, with therapies matched to mutations. A fifth cohort (E) recruiting patients with triple-negative breast cancer with no targetable mutation, designated to receive olaparib plus the ATR inhibitor AZD6738, is ongoing and will be reported separately. Eligible patients were women at least 18 years of age with histologically confirmed advanced breast cancer that was not suitable for treatment with radical or curative intent, who had measurable disease, an Eastern Cooperative Oncology Group performance status of 0–2, estimated life expectancy of more than 3 months, and were suitable for a baseline advanced disease biopsy or had an archival advanced disease biopsy available for subsequent retrospective sequencing and comparison with ctDNA. Patients were required to have had disease progression on radiological or clinical assessment at registration (with radiological confirmation required before treatment cohort entry), and to have completed at least one previous line of treatment for advanced breast cancer, or relapsed within 12 months of neoadjuvant or adjuvant chemotherapy. Patients with HER2-positive breast cancer must have had at least two previous lines of HER2-targeted therapy in the advanced setting (or one line if no further HER2-targeted therapies were available). An approved protocol amendment implemented on Feb 19, 2018, after 515 patients had been recruited, required a maximum of two previous lines of chemotherapy, antibody–drug conjugate, or immunotherapy. Exclusion criteria for ctDNA testing included uncontrolled CNS or cardiac disease, ongoing toxicities of grade 1 or higher from previous treatments, and malignancies of other types within the past 3 years. Cohort-specific eligibility criteria are given in the protocol (appendix).

The study was co-sponsored by the Institute of Cancer Research and the Royal Marsden National Health Service (NHS) Foundation Trust, London, UK, and approved by a Research Ethics Committee (16/SC/0271). All participants gave written informed consent before registration for ctDNA testing, and again before treatment cohort entry. Safety and efficacy data were reviewed regularly by an independent data monitoring committee. Trial oversight was provided by an independent trial steering committee.

### Procedures

ctDNA testing was done with two technologies. Digital droplet PCR was done at a central laboratory in the National Institute for Health Research Centre for Molecular Pathology at the Royal Marsden NHS Foundation Trust and Institute of Cancer Research prospectively in all patients, for mutations in *PIK3CA, ESR1, HER2,* and *AKT1* (appendix p 4). From July 10, 2018 (after recruitment of 680 patients), prospective testing also included error-corrected targeted sequencing with Guardant360 (Guardant Health; Redwood City, CA, USA) for a panel of 73 genes including *PIK3CA, ESR1, HER2, AKT1, PTEN*, and *TP53*, with retrospective sequencing for previously enrolled patients. For comparison with ctDNA results, tumour tissue sequencing using advanced disease tissue biopsies was done retrospectively for patients who entered a treatment cohort (appendix p 5). Testing for *PIK3CA* mutations was included to test the validity of the *PIK3CA* ctDNA testing, but was not used for entry to therapeutic cohorts as phase 3 studies of treatments for breast cancer with *PIK3CA* mutations were ongoing when plasmaMATCH started recruitment.^1^ A positive result by either ctDNA assay was sufficient for cohort entry. If more than one mutation was identified, entry to cohorts B–D took preference to cohort A.

Cohort A included individuals with *ESR1* mutations; they received extended-dose 500 mg fulvestrant (a selective oestrogen receptor downregulator) administered intramuscularly on days 1, 8, and 15 in cycle 1, and days 1 and 15 in cycle 2 onwards, on a 28-day cycle. Pharmacokinetic analysis samples were collected predose on cycles 2–4 and compared with a historical population model for standard-dose fulvestrant. In cohort A, as a prespecified exploratory analysis, *ESR1* mutations were determined to be clonally dominant or subclonal, with a clonally dominant mutation indicating a summed *ESR1* allele fraction of 50% or greater of maximum allele fraction detected in the sample by targeted sequencing to correct for variations in the purity of ctDNA in plasma DNA (appendix p 5).

Cohort B included individuals with *HER2* mutations; they received 240 mg neratinib (an irreversible pan-HER tyrosine kinase inhibitor) orally once a day on a continuous schedule. In patients with oestrogen receptor-positive breast cancer, this treatment was administered together with fulvestrant 500 mg intramuscularly at standard dosing (days 1 and 15 in cycle 1 and day 1 in cycle 2 onwards on a 28-day cycle).

Cohort C included individuals with *AKT1* mutations and oestrogen receptor-positive breast cancer; they received 400 mg capivasertib (selective AKT inhibitor) orally twice a day for 4 days on followed by 3 days off continuously with fulvestrant 500 mg intramuscularly at standard dosing.

Cohort D included individuals with an AKT pathway activating mutation (mutations in *AKT1* with oestrogen receptor-negative breast cancer, or *PTEN* inactivating mutations or homozygous deletion [irrespective of oestrogen receptor status]; full criteria are described in the appendix p 6). Patients with mutations identified in previous tumour sequencing done outside of plasmaMATCH were also eligible for this cohort. This cohort received 480 mg capivasertib monotherapy orally, twice a day for 4 days on, followed by 3 days off, continuously.

In addition to the eligibility criteria for ctDNA testing, for cohort assignment patients with a relevant targetable mutation had to have adequate haematological, renal, and hepatic function (adequate defined as absolute neutrophil count ≥1·0 × 10^9^ cells per L, platelet count ≥100 × 10^9^ per L, haemoglobin ≥9 g/dL, serum creatinine ≤1·5 × the upper limit of normal [ULN], total bilirubin ≤1·5 × ULN, alanine aminotransferase and aspartate aminotransferase ≤3 × ULN [or ≤5 × ULN in the presence of liver metastases]). For cohorts C and D, patients were excluded if baseline glycated haemoglobin (HbA_1c_) was ≥8·0% (64 mmol/mol) or fasting plasma glucose was ≥7·0 mmol/L (126 mg/dL), or they had poorly controlled diabetes. Patients were eligible for cohort entry with detection of a mutation at any allele fraction, and clonal dominance was not considered in eligibility.

Once enrolled into a cohort, treatment was given until disease progression, unacceptable toxicity, or pregnancy. Participants could also discontinue from trial treatment at any time at their own request or be discontinued at the discretion of the treating clinician. Within each cohort, dose modifications were permitted for patients experiencing toxicities related to treatment.

Patients underwent CT or MRI scan and bone scan at baseline, with CT or MRI scan repeated every 8 weeks until 32 weeks after commencing treatment, and every 12 weeks thereafter, for disease evaluation using Response Evaluation Criteria in Solid Tumors (RECIST) version 1.1 criteria. There was no independent central review of disease outcome. Laboratory assessments, adverse event recording, and vital signs were performed every 4 weeks at a minimum. Toxicity was assessed using National Cancer Institute Common Terminology Criteria for Adverse Events version 4. Coding was done with use of the Medical Dictionary for Regulatory Activities version 22.

### Outcomes

The primary endpoint for cohorts A–D was confirmed objective response rate defined as a confirmed complete response or partial response according to RECIST criteria at any point during trial treatment. Secondary endpoints included duration of response (defined as time from the first documentation of complete response or partial response until date of disease progression or last date of follow-up), clinical benefit rate (defined as complete response, partial response, or stable disease for more than 6 months during trial treatment), progression-free survival (defined as time from cohort entry to first date of either confirmed progression of disease according to RECIST criteria or death from any cause), safety and tolerability of therapies, frequency of mutations, accuracy of ctDNA testing by agreement between ctDNA mutation status and tissue mutation status and the proportion of patients entering a cohort, and pharmacokinetics (for cohorts A and B). Prespecified exploratory endpoints included confirmed response rate in clonally dominant versus subclonal mutations in cohort A.

### Statistical analysis

All cohorts used a single-stage A'Hern design with α 5%, to have 80% power. Cohort A assumed an unacceptable response rate of 10% and a target response rate of 20% in the final design, requiring 13 or more responses from 78 evaluable patients to infer activity. This assumption was an approved amendment (on May 1, 2018) to the original two-stage design to account for the ctDNA testing detecting subclonal mutations as well as clonal mutations where the response rate was expected to be lower (appendix p 6). Cohorts B, C, and D each assumed an unacceptable response rate of 5% and a target response rate of 25%, requiring three or more responses from 16 evaluable patients. Over-recruitment into cohorts B, C, and D was allowed while ctDNA testing remained active. We estimated that 1000 patients would be required to enter ctDNA testing to recruit sufficient patients for cohorts B and C. This number gave 85% probability of identifying 25 patients for a mutation with prevalence of 3% for each of cohorts B, C, and D individually, allowing for 36% attrition between ctDNA screening and cohort entry.

Objective response rate, duration of response, and clinical benefit rate were measured in an evaluable population defined as those patients with measurable disease per RECIST at baseline and at least one on-treatment assessment; patients who stopped treatment because of intolerable toxicity or death without having a scan after baseline were evaluable and recorded as non-responders. Proportions and two-sided 95% CIs for estimation purposes were reported for each cohort and in prespecified subgroup analyses (by clonally dominant versus subclonal mutations in cohort A, compared using a Fisher's exact test, by hormone receptor status in cohort B, and by *AKT1* or *PTEN* mutation in cohort D). Analysis of clonality of *HER2* mutations in cohort B and of *AKT1* in cohort C was a post-hoc analysis.

In post-hoc exploratory analyses, response rates for each cohort were reported by *PIK3CA* and *TP53* mutation status and, for cohort A, tissue mutation status. For inference purposes, thus corresponding to the design characteristics underpinning the trial's hypothesis testing (ie, alpha 5%, one-sided), proportions and two-sided 90% CIs are reported.

Progression-free survival was measured in the intention-to-treat population. Kaplan-Meier curves were plotted and median progression-free survival is reported with 95% CIs for each cohort. Patients who were alive and progression free were censored at date of last follow-up; patients who had non-RECIST confirmed progression (eg, clinically judged progression or radiologically confirmed but lesions not measured according to RECIST) were censored at the date progression was reported. The safety population included all patients who had at least one dose of treatment, regardless of their eligibility, and treatment-emergent adverse events where more than 10% of patients reported any grade of the adverse event or any patients reported the adverse event at grade 3 or higher were presented. In addition, pharmacokinetics were reported as a percentage change from an approved historical population pharmacokinetic model for standard-dosing 500 mg fulvestrant, in cohort A only. Pharmacokinetic data for cohort B will be reported elsewhere. Safety was assessed on the basis of the incidence of adverse events.

Agreement between digital PCR and targeted sequencing was assessed with a kappa score and 95% CIs. Comparison of ctDNA with retrospective tissue biopsy sequencing was reported with sensitivity, specificity, and 95% CIs.

Analyses used a database snapshot taken on Nov 6, 2019. Where reported, p values of less than 0·05 were deemed significant. All analyses were conducted using Stata (version 15.1). This study is registered with ClinicalTrials.gov, NCT03182634; the European Clinical Trials database, EudraCT2015-003735-36; and the ISRCTN registry, ISRCTN16945804.

### Role of the funding source

The funders of the study had no role in study design, data collection, data analysis, data interpretation, or writing of the report. AstraZeneca and Puma Biotechnology reviewed the final version of the report, but had no role in the decision to submit the manuscript for publication. The corresponding author had full access to all of the data and the final responsibility to submit for publication.

## Results

**Figure 1:**
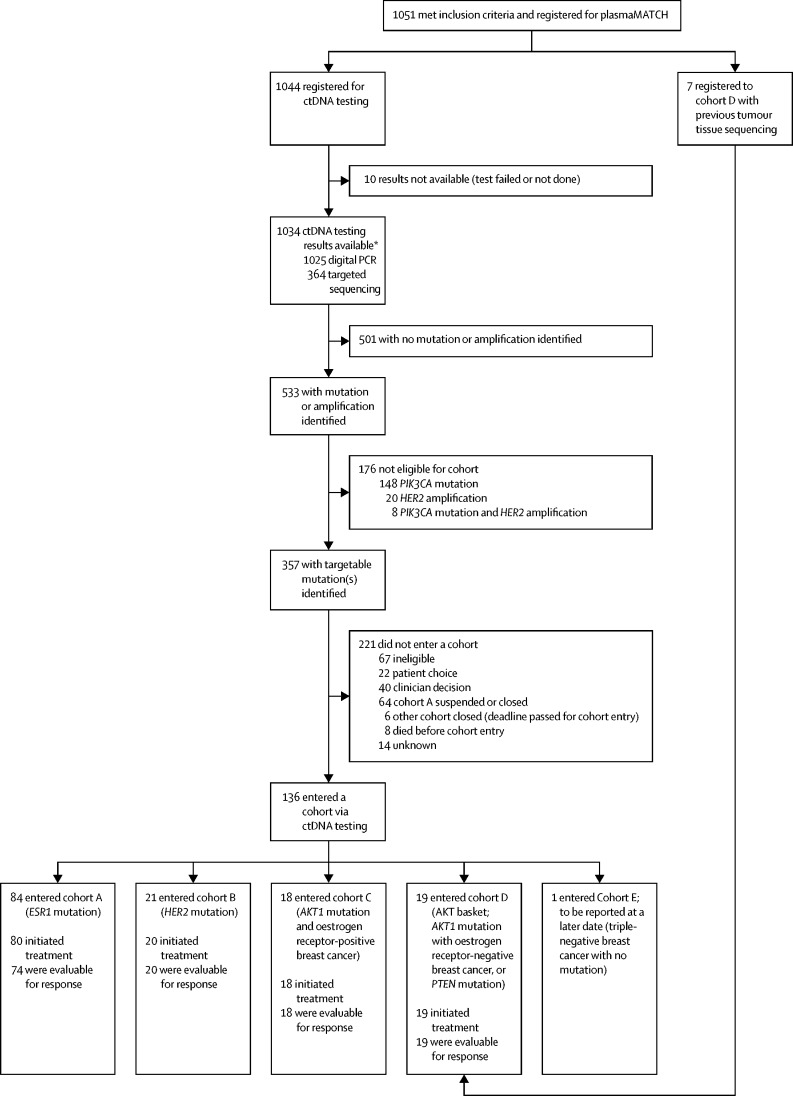
Trial profile Further detail on accuracy of ctDNA testing is provided in the appendix (p 17). ctDNA=circulating tumour DNA. *436 additional samples were analysed by targeted sequencing retrospectively; these were not used for determining cohort suitability; agreement between digital PCR and targeted sequencing (n=800) was as follows: *AKT1* kappa 0·93 (95% CI 0·87–0·99), *HER2* kappa 0.89 (0·79–0·98), *ESR1* kappa 0·90 (0·86–0·93), and *PIK3CA* kappa 0·92 (0·89–0·95).

Between Dec 21, 2016, and April 26, 2019, 1051 patients were registered into the study (1044 via ctDNA screening, seven via previous tumour sequencing; figure 1; appendix p 17) with ctDNA results available for 1034 patients (99·0%). Digital PCR results were available for 1025 patients (98·2%) and targeted sequencing results were available for 800 patients (76·6%; 364 prospective and 436 retrospective). The median time from blood draw to ctDNA results was 13 days (IQR 11–15) for digital PCR and 10 days (8–11) for sequencing. Patients had a median of one (IQR 0–2) previous lines of chemotherapy, and a median of two (1–3) previous lines of systemic therapy (table).

**Table:** Baseline characteristics

			**All registered patients (n=1051)**	**Patients with *ESR1* mutation (n=222)**		**Patients with *HER2* mutation (n=36)**		**Patients with oestrogen receptor-positive breast cancer and *AKT1* mutation (n=30)**		**Patients with oestrogen receptor-negative breast cancer and *AKT1* mutation or *PTEN* mutation (n=37)**	
				Entered cohort A (n=84)	Did not enter cohort A (n=138)	Entered cohort B (n=21)	Did not enter cohort B (n=15)	Entered cohort C (n=18)	Did not enter cohort C (n=12)	Entered cohort D (n=19)	Did not enter cohort D (n=18)
Age group at registration (years)											
	<50		285 (27·1%)	18 (21·4%)	32 (23·2%)	2 (9·5%)	2 (13·3%)	3 (16·7%)	2 (16·7%)	7 (36·8%)	3 (16·7%)
	50–59		348 (33·1%)	36 (42·9%)	48 (34·8%)	9 (42·9%)	3 (20·0%)	7 (38·9%)	3 (25·0%)	7 (36·8%)	11 (61·1%)
	60–69		265 (25·2%)	20 (23·8%)	36 (26·1%)	6 (28·6%)	8 (53·3%)	6 (33·3%)	4 (33·3%)	4 (21·1%)	3 (16·7%)
	≥70		153 (14·6%)	10 (11·9%)	22 (15·9%)	4 (19·0%)	2 (13·3%)	2 (11·1%)	3 (25·0%)	1 (5·3%)	1 (5·6%)
Metastatic disease present at diagnosis			146 (13·9%)	18 (21·4%)	20 (14·5%)	2 (9·5%)	2 (13·3%)	5 (27·8%)	2 (16·7%)	3 (15·8%)	2 (11·1%)
Histological type at tumour diagnosis											
	Ductal		777 (73·9%)	63 (75·0%)	103 (74·6%)	9 (42·9%)	6 (40·0%)	13 (72·2%)	9 (75·0%)	14 (73·7%)	11 (61·1%)
	Lobular		98 (9·3%)	9 (10·7%)	12 (8·7%)	8 (38·1%)	3 (20·0%)	3 (16·7%)	1 (8·3%)	2 (10·5%)	4 (22·2%)
	Mixed ductal and lobular		38 (3·6%)	5 (6·0%)	5 (3·6%)	1 (4·8%)	2 (13·3%)	1 (5·6%)	1 (8·3%)	2 (10·5%)	0
	Mixed ductal and mucinous		3 (0·3%)	1 (1·2%)	0	0	0	0	0	0	1 (5·6%)
	Other invasive		13 (1·2%)	0	2 (1·4%)	0	1 (6·7%)	0	0	0	1 (5·6%)
	Ductal carcinoma in situ		2* (0·2%)	1 (1·2%)	1 (0·7%)	0	0	0	0	0	0
	Not known or missing		120 (11·4%)	5 (6·0%)	15 (10·9%)	3 (14·3%)	3 (20·0%)	1 (5·6%)	1 (8·3%)	1 (5·3%)	1 (5·6%)
Tumour grade											
	1		48 (4·6%)	7 (8·3%)	10 (7·2%)	2 (9·5%)	1 (6·7%)	1 (5·6%)	1 (8·3%)	0	0
	2		402 (38·2%)	37 (44·0%)	70 (50·7%)	11 (52·4%)	6 (40·0%)	11 (61·1%)	8 (66·7%)	11 (57·9%)	6 (33·3%)
	3		445 (42·3%)	28 (33·3%)	44 (31·9%)	4 (19·0%)	5 (33·3%)	5 (27·8%)	1 (8·3%)	6 (31·6%)	12 (66·7%)
	Not known or missing		156 (14·8%)	12 (14·3%)	14 (10·1%)	4 (19·0%)	3 (20·0%)	1 (5·6%)	2 (16·7%)	2 (10·5%)	0
Molecular subtype†											
	HR positive, HER2 negative		676 (64·3%)	80 (95·2%)	125 (90·6%)	17 (81·0%)	9 (60·0%)	16 (88·9%)	9 (75·0%)	13 (68·4%)	10 (55·6%)
	HR positive, HER2 positive		65 (6·2%)	3 (3·6%)	0	1 (4·8%)	1 (6·7%)	1 (5·6%)	0	0	0
	HR negative, HER2 positive		36 (3·4%)	0	0	2 (9·5%)	2 (13·3%)	0	0	0	1 (5·6%)
	Triple-negative breast cancer		179 (17·0%)	0	1 (0·7%)	1 (4·8%)	1 (6·7%)	0	0	6 (31·6%)	5 (27·8%)
	HR positive, HER2 unknown		39 (3·7%)	1 (1·2%)	7 (5·1%)	0	1 (6·7%)	1 (5·6%)	3 (25·0%)	0	1 (5·6%)
	Other‡		14 (1·3%)	0	1 (0·7%)	0	1 (6·7%)	0	0	0	1 (5·6%)
	Not known or missing		42 (4·0%)	0	4 (2·9%)	0	0	0	0	0	0
Disease sites at diagnosis											
	Visceral		824 (78·4%)	78 (92·9%)	114 (82·6%)	18 (85·7%)	12 (80·0%)	17 (94·4%)	10 (83·3%)	14 (73·7%)	11 (61·1%)
	Soft tissue or nodal		668 (63·6%)	56 (66·7%)	66 (47·8%)	12 (57·1%)	11 (73·3%)	11 (61·1%)	5 (41·7%)	15 (78·9%)	15 (83·3%)
	Bone		638 (60·7%)	76 (90·5%)	107 (77·5%)	16 (76·2%)	9 (60·0%)	18 (100·0%)	11 (91·7%)	12 (63·2%)	12 (66·7%)
Treatment received for locally advanced or metastatic disease before study registration											
	Chemotherapy		728 (69·3%)	55 (65·5%)	88 (63·8%)	18 (85·7%)	10 (66·7%)	15 (83·3%)	8 (66·7%)	12 (63·2%)	9 (50·0%)
		1 line	345 (32·8%)	26 (31·0%)	38 (27·5%)	7 (33·3%)	4 (26·7%)	8 (44·4%)	5 (41·7%)	7 (36·8%)	4 (22·2%)
		2 lines	201 (19·1%)	13 (15·5%)	27 (19·6%)	9 (42·9%)	3 (20·0%)	3 (16·7%)	3 (25·0%)	4 (21·1%)	3 (16·7%)
		>2 lines§	181 (17·2%)	16 (19·0%)	22 (15·9%)	2 (9·5%)	3 (20·0%)	4 (22·2%)	0	1 (5·3%)	2 (11·1%)
		Unknown	1 (0·1%)	0	1 (0·7%)	0	0	0	0	0	0
	Endocrine therapy¶		685 (65·2%)	79 (94·0%)	127 (92·0%)	14 (66·7%)	11 (73·3%)	18 (100·0%)	12 (100·0%)	11 (84·6%)	7 (63·6%)
		1 line	323 (30·7%)	34 (40·5%)	45 (32·6%)	3 (14·3%)	3 (20·0%)	9 (50·0%)	8 (66·7%)	8 (61·5%)	3 (27·3%)
		2 lines	230 (21·9%)	36 (42·9%)	49 (35·5%)	6 (28·6%)	5 (33·3%)	6 (33·3%)	1 (8·3%)	3 (23·1%)	4 (36·4%)
		3 lines	117 (11·1%)	8 (9·5%)	30 (21·7%)	5 (23·8%)	2 (13·3%)	3 (16·7%)	3 (25·0%)	0	0
		>3 lines	15 (1·4%)	1 (1·2%)	3 (2·2%)	0	1 (6·7%)	0	0	0	0
	Total lines of systemic therapy										
		0	93 (8·9%)	2 (2·4%)	6 (4·3%)	0	1 (6·7%)	0	0	3 (15·8%)	6 (33·3%)
		1	275 (26·2%)	15 (17·9%)	25 (18·1%)	3 (14·3%)	3 (20·0%)	1 (5·6%)	3 (25·0%)	5 (26·3%)	3 (16·7%)
		2	243 (23·1%)	23 (27·4%)	30 (21·7%)	4 (19·0%)	3 (20·0%)	6 (33·3%)	2 (16·7%)	5 (26·3%)	5 (27·8%)
		3	178 (16·9%)	16 (19·0%)	28 (20·3%)	8 (38·1%)	4 (26·7%)	5 (27·8%)	5 (41·7%)	4 (21·1%)	2 (11·1%)
		4	109 (10·4%)	12 (14·3%)	18 (13·0%)	4 (19·0%)	2 (13·3%)	3 (16·7%)	2 (16·7%)	2 (10·5%)	0
		5	88 (8·4%)	11 (13·1%)	19 (13·8%)	2 (9·5%)	0	0	0	0	2 (11·1%)
		>5	65 (6·2%)	5 (6·0%)	12 (8·7%)	0	2 (13·3%)	3 (16·7%)	0	0	0
	Other systemic therapy‖		421 (40·1%)	41 (48·8%)	59 (42·8%)	11 (52·4%)	8 (53·3%)	9 (50·0%)	4 (33·3%)	11 (57·9%)	7 (38·9%)
		Anti-HER2 therapy	89 (8·5%)	3 (3·6%)	1 (0·7%)	3 (14·3%)	3 (20·0%)	1 (5·6%)	0	0	0
		mTOR inhibitor (everolimus or vistusertib)	116 (11·0%)	18 (21·4%)	31 (22·5%)	5 (23·8%)	2 (13·3%)	3 (16·7%)	1 (8·3%)	3 (15·8%)	2 (11·1%)
		CDK4/6 inhibitor (palbociclib, ribociclib, or abemaciclib)	89 (8·5%)	8 (9·5%)	14 (10·1%)	1 (4·8%)	1 (6·7%)	6 (33·3%)	0	4 (21·1%)	1 (5·6%)
		Immunotherapy (atezolizumab or pembrolizumab)	20 (1·9%)	0	0	0	0	0	0	0	0
		Other	45 (4·3%)	1** (1·2%)	6 (4·3%)	1†† (4·8%)	2 (13·3%)	0	3 (25·0%)	2‡‡ (10·5%)	1 (5·6%)

Data are n (%). HR=hormone receptor (oestrogen or progesterone receptor).

* Two patients originally had ductal carcinoma in situ only as their primary diagnosis, but relapsed with invasive advanced cancer.

† Determined at local hospitals from recurrence biopsy (or primary biopsy if recurrence biopsy unavailable).

‡ Other molecular subtypes were: four HR negative, HER2 unknown; nine HR unknown, HER2 negative; one HR unknown, HER2 positive.

§ Study was amended after 515 patients had been recruited to require a maximum of two previous lines of chemotherapy.

¶ For patients with oestrogen receptor-negative breast cancer and *AKT1* mutation or *PTEN* mutation the denominator is patients with HR-positive disease only (for those who entered cohort D n=13; for those that did not enter cohort D n=11).

‖ Patients may be included in more than one type of systemic therapy, but patients are only included once in each category (eg, if a patient had trastuzumab and pertuzumab they are counted once in the anti-HER2 therapy category).

** Taselisib.

†† Capivasertib.

‡‡ Lucitanib (n=1) and olaparib (n=1).

A somatic mutation was detected in 743 (93%) of 800 patients with ctDNA targeted sequencing results (appendix p 18). *ESR1* mutations were found almost exclusively in hormone receptor-positive breast cancer, were found at lower average allele fractions than other mutations, and were frequently polyclonal (appendix pp 17–18). *HER2* mutations were found least frequently in triple-negative breast cancer, while *AKT1* mutations were found at a similar frequency in hormone receptor-positive HER2-negative breast cancer and triple-negative breast cancer (appendix p 18).

Gene-level agreement for mutation identification between ctDNA digital PCR and targeted sequencing (n=800) was 96–99% (kappa 0·89–0·93; figure 1). Individual mutation agreements were also high (appendix p 19). An advanced tissue biopsy was sequenced retrospectively for 77 patients who entered a cohort. Sensitivity (or percent-positive agreement reflecting the absence of gold standard) for digital PCR was 93% (95% CI 83–98) overall and 98% (87–100) in patients with contemporaneous biopsies (appendix pp 17, 20). By contrast, digital PCR sensitivity in patients with time-discordant biopsies was 85% (95% CI 66–96; appendix p 20). Sensitivity for targeted sequencing was 95% (95% CI 87–99) overall and 100% (92–100) in patients with contemporaneous biopsies (appendix p 21). Sensitivity for time-discordant biopsises is show in the appendix (p 20). Specificity for both digital PCR and sequencing (or percent-negative agreement reflecting the absence of gold standard) was high for *AKT1, HER2*, and *PIK3CA*, varying by gene (appendix p 17). *ESR1* mutations had lower percent-negative agreement.

Mutations were identified in 533 (51·1%) of 1044 patients registered for ctDNA testing and 357 (34·5%) of 1034 with results had targetable mutations eligible for cohort entry, of whom 136 entered one of the five available cohorts. In patients who entered cohorts A–D, combined median follow-up was 14·4 months (IQR 7·0–23·7). The most common reason for not entering a cohort was that the patient was ineligible based on the specific eligibility criteria for the relevant cohort, or in the case of cohort A (*ESR1* mutation), 64 patients did not enter because of cohort A being suspended (while the protocol amendment to change the design was ongoing) or closed (figure 1; appendix p 7).

**Figure 2:**
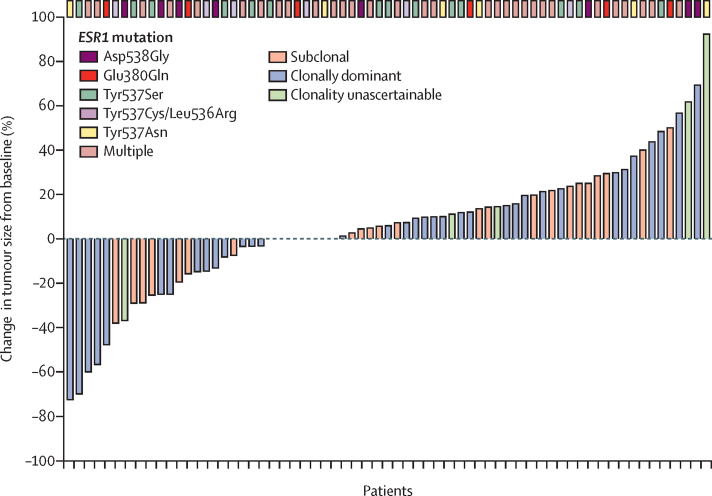
Extended-dose fulvestrant in *ESR1*-mutant breast cancer (cohort A) Waterfall plot of maximum change in tumour size in individual patients with *ESR1* mutations in ctDNA treated with extended-dose fulvestrant. ctDNA=circulating tumour DNA.

84 (38%) of 222 patients with an *ESR1* mutation in ctDNA identified while cohort A was open to recruitment were enrolled in cohort A (figure 1, table). The most common *ESR1* mutations detected in plasma were Asp538Gly (45 [54%] of 84), Tyr537Ser (31 [37%]), and Glu380Gln (29 [35%]). 74 patients were evaluable for response: four patients did not start treatment and six did not have on-treatment RECIST-assessable imaging. Six (8% [95% CI 3–17]) of 74 patients had a confirmed partial response with a median duration of response of 7·0 months (IQR 3·7–8·3) and four patients continuing on treatment at data cutoff (figure 2). The clinical benefit rate was 12 (16% [95% CI 9–27]) of 74 patients. 69 (82%) of 84 patients had a RECIST-confirmed progression event or death. Median progression-free survival was 2·2 months (95% CI 1·8–3·6; appendix p 22). In a pre-planned exploratory analysis, five (12% [95% CI 4–26]) of 41 patients with clonally dominant *ESR1* mutations, and none (0% [0–13]) of 27 patients with subclonal mutations had a confirmed response (p=0·15; 27 [40%] of 68 *ESR1* mutations were subclonal); six patients had unknown clonality. The most common grade 3 or grade 4 adverse event was increased gamma-glutamyltransferase (13 [16%] of 80 patients; appendix p 9). One patient had a serious adverse reaction: grade 3 superior sagital sinus thrombosis. There were no treatment-related deaths and 41 (49%) all-cause deaths reported (38 breast cancer and three unknown cause). The main reason for treatment discontinuation was disease progression (72 [95%] of 76 patients; appendix p 8). Two patients had reductions of fulvestrant dosing frequency. 29 patients did not have a day 15 injection given on at least one occasion. Ten delays to treatment were reported in nine patients. The median relative dose intensity in patients starting treatment was 100% (IQR 97–100, range 33–104). Pharmacokinetic analysis was consistent with elevated fulvestrant exposure compared with standard-dosing 500 mg fulvestrant in a historical population pharmacokinetic model (appendix p 10). Fulvestrant activity was similar in patients with and without *ESR1* mutations in tissue sequencing (appendix p 29).

**Figure 3:**
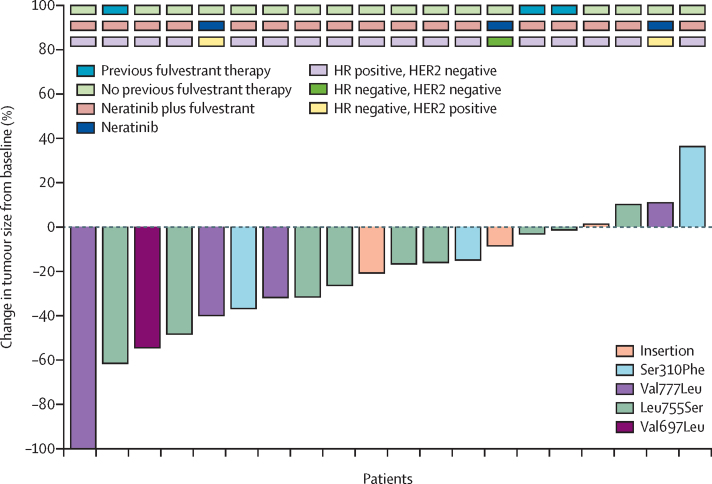
Neratinib in *HER2*-mutant breast cancer (cohort B) Waterfall plot of maximum change in tumour size in individual patients with *HER2* mutations in ctDNA treated with neratinib alone or neratinib plus fulvestrant. HR=hormone receptor. ctDNA=circulating tumour DNA.

21 (58%) of 36 patients with an *HER2* mutation in ctDNA were enrolled in cohort B (figure 1, table). The most common *HER2* mutations detected in plasma were Leu755Ser (ten [48%] of 21 patients), Val777Leu (four [19%]), and Ser310Phe (three [14%]). 20 patients were evaluable for response, as one patient did not start treatment. Five (25% [95% CI 9–49) of 20 patients had a confirmed response (one complete and four partial), and an additional three patients had unconfirmed partial responses (figure 3). Four (25% [95% CI 7–52]) of the protocol-specified first 16 evaluable patients had a response. One patient had a complete response, ongoing at 29 months duration. Median duration of response was 5·7 months (IQR 3·7–9·7) with three patients continuing on treatment at data cutoff. The clinical benefit rate was nine (45% [95% CI 23–68]) of 20 patients. 16 (76%) of 21 patients had a RECIST-confirmed progression event or death. Median progression-free survival was 5·4 months (95% CI 3·4–9·1; appendix p 23). In the subgroup of patients with hormone receptor-positive HER2-negative breast cancer treated with neratinib and fulvestrant, four (24% [95% CI 7–50]) of 17 patients had a confirmed response (figure 3). The single triple-negative patient with *HER2* mutation did not respond. In a post-hoc analysis, three (16%) of 19 *HER2* mutations were subclonal (appendix p 24). The most common grade 3 or grade 4 adverse events were diarrhoea (four [20%] of 20 patients) and hypertension (three [15%]; appendix p 11). Four serious adverse reactions were reported in three patients (appendix pp 11–12). There were no treatment-related deaths and 13 (62%) all-cause deaths reported (12 breast cancer and one unknown cause). The main reason for treatment discontinuation was disease progression (16 [94%] of 17 patients; appendix p 8). All 17 patients with oestrogen receptor-positive breast cancer who started treatment received all doses of fulvestrant. Neratinib dose was reduced to 160 mg for six (30%) of 20 patients, and one of these patients had a further dose reduction to 120 mg. The median relative dose intensity of patients starting treatment was 92% (IQR 84–99; range 59–100) for neratinib and 100% (100–100; 96–102) for fulvestrant.

**Figure 4:**
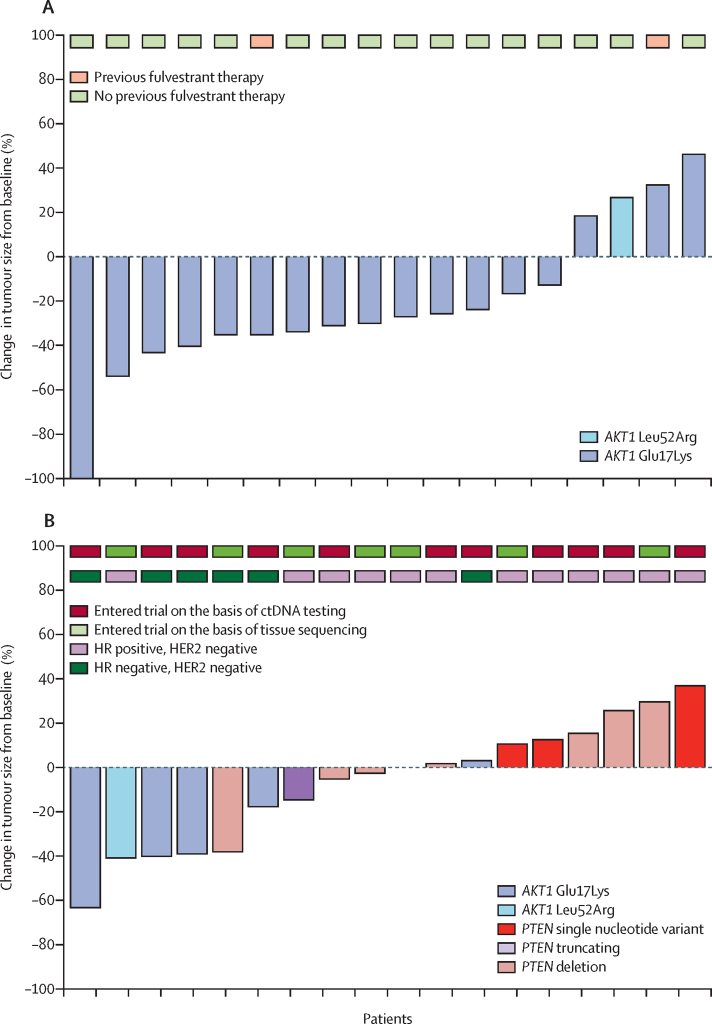
Capivasertib in *AKT1*-mutant and *PTEN*-mutant breast cancer (cohorts C and D) (A) Waterfall plot of maximum change in tumour size in individual patients with HR-positive cancer and *AKT1* mutations in ctDNA, treated with capivasertib plus fulvestrant (cohort C). (B) Waterfall plot of maximum change in tumour size in individual patients with *AKT1* mutations and HR-negative breast cancer, or with activating *PTEN* mutations, treated with capivasertib (cohort D). ctDNA=circulating tumour DNA. HR=hormone receptor. *PTEN* truncating=*PTEN* truncating nonsense or frameshift mutation. *PTEN* deletion=*PTEN* homozygous deletion.

18 (60%) of 30 patients with an *AKT1* mutation in ctDNA and oestrogen receptor-positive cancer were enrolled in cohort C (figure 1; table). The most common mutation detected was Glu17Lys (17 [94%] of 18 patients), and Leu52Arg was detected in one patient (6%). All 18 patients were evaluable; four (22% [95% CI 6–48]) patients had a confirmed partial response, and an additional four patients had unconfirmed partial responses (figure 4A). Three (19% [4–46]) of the protocol-specified first 16 evaluable patients had a response. Median duration of response was 7·5 months (IQR 4·1–9·8) with four patients continuing on treatment at data cutoff. The clinical benefit rate was seven (39% [95% CI 17–64]) of 18 patients. 12 (67%) of 18 patients had a RECIST-confirmed progression event or death. Median progression-free survival was 10·2 months (95% CI 3·2–18·2; appendix p 25). In a post-hoc analysis, four (23%) of 17 *AKT1* mutations (clonality was assessable in 17 patients) were subclonal (appendix p 24). The most common grade 3 or 4 adverse events were fatigue (four [22%] of 18 patients), rash (three [17%]), diarrhoea (two [11%]), and hyperglycaemia (two [11%]; appendix pp 13–14). Eight serious adverse reactions were reported in four patients (appendix p 14). There was one treatment-related death caused by grade 4 dyspnoea and six (33%) breast cancer deaths reported. The main reason for treatment discontinuation was disease progression (11 [85%] of 14 patients; appendix p 8). All 18 patients received all doses of fulvestrant. Capivasertib dose was reduced to 320 mg in seven (39%) of 18 patients, and three of these patients had a further reduction to 240 mg. The median relative dose intensity was 88% (IQR 70–99, range 25–101) for capivasertib and 99% (IQR 97–100, range 94–102) for fulvestrant.

19 patients were enrolled in cohort D, 12 following ctDNA testing and seven following tumour testing (figure 1, table 1). The mutations detected were *AKT1* Glu17Lys (five [26%] of 19 patients), *AKT1* Leu52Arg (one [5%]), *PTEN* inactivating mutation (12 [63%]), and *PTEN* homozygous deletion (one [5%]). All 19 patients were evaluable, and two (11% [95% CI 1–33]) patients had a confirmed partial response (figure 4B). Two (33%, 95% CI 4–78) of the six patients with *AKT1* mutations responded, and there were two further unconfirmed responses in these patients. None of the patients with *PTEN* genomic alterations responded. Two (13% [95% CI 2–38]) of the protocol-specified first 16 patients responded. Median duration of response was 3·9 months (IQR 3·7–4·2) with one patient continuing on treatment at data cutoff. Two (11% [95% CI 1–33]) of 19 patients had a clinical benefit. 13 (68%) of 19 patients had a RECIST-confirmed progression event or death. Median progression-free survival was 3·4 months (95% CI 1·8–5·5; appendix p 26). The most common grade 3 or grade 4 adverse events were rash (five [26%] of 19 patients), hypertension (two [11%]), aminotransferase increase (two [11%]), gamma-glutamyltransferase increase (two [11%]), and vomiting (two [11%]; appendix p 15). Four serious adverse reactions were reported in three patients (appendix p 15). There were no treatment-related deaths and ten (53%) breast cancer deaths reported. The main reason for treatment discontinuation was disease progression (15 [83%] of 17 patients; appendix p 8). Capivasertib dose was reduced in five (26%) of 19 patients (four to 400 mg and one to 320 mg). The median relative dose intensity of capivasertib was 94% (IQR 63–100, range 42–102).

In post-hoc analyses, the response rates in cohorts A–D did not vary by *PIK3CA* or *TP53* co-mutational status (appendix pp 27–28).

## Discussion

In this large, prospective trial of ctDNA testing in advanced breast cancer, we found that ctDNA testing was highly accurate, with high agreement between different ctDNA testing techniques, and high sensitivity for mutations identified in advanced breast cancer tissue biopsies. ctDNA testing identified patients with rare targetable mutations and these patients were recruited into cohorts that were given targeted therapies (matched to mutations) without confirmatory tumour testing, with activity comparable to previous studies involving tumour tissue testing.^4^, ^5^ We enrolled more than 1000 patients across the UK in less than 3 years, and the dynamic trial platform design allowed for the simultaneous evaluation of multiple targeted treatment options.

The availability and accuracy of ctDNA testing shown in this study compares favourably with tissue-based mutation testing. Nearly all patients (99%) received a result from ctDNA testing, contrasting with previous tumour sequencing studies where results were typically received in only 70–90% of patients.^15^, ^16^ In addition, previous tumour sequencing studies generally only included patients with disease that could be biopsied, which is not a constraint for ctDNA testing. Results were received relatively quickly after blood draw, compared with results for tissue-based testing, and this led to a high conversion rate of patients with ctDNA mutations into the corresponding treatment cohort. The accuracy of ctDNA testing was also similar to that achieved with tissue sequencing.^17^ Discordance between ctDNA results was still observed for patients at low allele frequency mutations, suggesting further potential for assay development. *ESR1* mutations had lower percent-negative agreement, probably reflecting the subclonality of acquired *ESR1* mutations, with ctDNA detecting mutations present in metastasic sites other than the one biopsied. Nevertheless, the degree of sensitivity observed in this study suggests that, within the patient population of advanced disease patients recruited, ctDNA testing could replace tissue-based mutation analysis. However, we note that tissue biopsy will remain important for immunohistochemistry, and for copy number-based assessment. Digital PCR offered similar accuracy to sequencing, with substantial cost efficiency, although this comparison was limited to the specific mutations analysed. The academic clinical laboratory doing the digital PCR assay achieved the trial target turnaround time of results within 14 days. A shorter turnaround time could easily be achieved if required in clinical practice, resulting in a cost-efficient method of ctDNA analysis. 533 (51·1%) of 1044 patients who underwent ctDNA testing had a potentially targetable mutation (*PIK3CA, ESR1, HER2, AKT1*, or *PTEN*), indicating a potential value for ctDNA testing.

Our results confirm clinically relevant activity of targeted therapies against rare activating mutations in breast cancer. In a previous phase 1 study with an expansion cohort of those with *HER2*-mutant breast cancer identified in tissue, who were given neratinib, there was a 32% unconfirmed response rate after 8 weeks of treatment.^4^ In our study, neratinib for *HER2*-mutant breast cancer identified by ctDNA testing had comparable activity to that observed when guided by tissue testing, with durable responses. Similarly, capivasertib had high activity in patients with ctDNA-identified *AKT1* mutations, both in hormone receptor-positive cancer with fulvestrant and in hormone receptor-negative cancer as a single agent, again confirming the results of a previous phase 1 study.^5^ These results confirm the high activity of these drugs against *HER2* and *AKT1* mutations, and strongly support the need for registration trials, facilitated by a ctDNA testing programme.

Our study did not show benefit from increasing the dose of fulvestrant in patients with *ESR1* mutations in ctDNA. Previous research has suggested that fulvestrant at standard doses does not maximally inhibit or degrade mutated *ESR1*,^18^ and we assessed whether more frequent administration of fulvestrant would increase therapeutic utility. Although exposure was increased in later cycles, this was insufficient to enhance activity, with the response rate remaining similar to that previously reported.^19^, ^20^ We note however that our study recruited a heavily pretreated population, and this might have reduced the activity of fulvestrant. More potent oestrogen receptor inhibitors, such as novel oral oestrogen receptor degraders and modulators, are also likely to be required.^21^ We found that patients with Tyr537Ser *ESR1* mutations were no less sensitive to fulvestrant than those with other *ESR1* mutations, and that *ESR1* mutations were frequently subclonal, with detection of *ESR1* mutations in ctDNA that were not present in contemporaneous single site tissue biopsies, reflecting the limited sampling of single site tissue biopsies. Fulvestrant activity was similar in patients with and without *ESR1* mutations in tissue sequencing.

Our study has limitations. Inclusion of relatively heavily pretreated patients might reduce activity of the targeted drugs, especially in cohort A, and future ctDNA selection trials might benefit from more restrictive entry criteria. The study was designed to assess the activity of therapies against specific genomic events, but it did not target *PIK3CA* mutations,^1^ and as a result relatively few of the patients registered to the trial had a response to therapy (17 [1·6%] of 1051 patients). However, mutation-directed therapy with alpelisib is now approved to target *PIK3CA* mutations, and our study shows the clinical validity of using ctDNA to direct therapy. Cohort D was designed as a basket cohort from the outset, to explore the activity of capivasertib against different AKT pathway activating mutations. Only cohort D allowed entry of patients with previous tissue sequencing results, as it was anticipated that ctDNA testing alone might not recruit sufficient patients. Although we identified low activity of capivasertib in *PTEN*-mutant cancers when used as a single agent, AKT inhibition in combination with paclitaxel chemotherapy might be efficacious in *PTEN*-mutant cancers.^22^, ^23^ Capivasertib plus fulvestrant might be efficacious in endocrine-resistant oestrogen receptor-positive breast cancer without mutation selection, as shown in the FAKTION trial.^24^ It is not possible to robustly compare plasmaMATCH with FAKTION, as patients enrolled in plasmaMATCH had more previous lines of treatment, and *AKT1* mutations were not assessed and would be few in number in FAKTION.^24^

In conclusion, we show that ctDNA testing, with the assays employed in this study, has sufficient accuracy for widespread adoption in routine clinical practice to identify patients with breast cancer who are suitable for licensed targeted therapies, such as *PIK3CA*-mutant breast cancer, with the transformative potential of efficient and rapid screening for clinical trials. A high proportion of patients with specific targeted mutations were able to enrol on the matching treatment cohort, with clinically important activity observed with therapies matched to *AKT1* and *HER2* mutations. With mutation-matching therapy now approved in breast cancer, with alpelisib for *PIK3CA*-mutant disease, ctDNA testing can be seen as a standard-of-care test for both common and rare targetable genetic events.

## Data sharing

De-identified individual participant data, together with a data dictionary defining each field in the dataset, will be made available to other researchers on request, subject to approval of a formal data access request in accordance with the ICR-CTSU data and sample access policy. Trial documentation including the protocol are available on request by contacting plasmamatch-icrctsu@icr.ac.uk. The ICR-CTSU supports the wider dissemination of information from the research it conducts, and increased cooperation between investigators. Trial data are collected, managed, stored, shared, and archived according to ICR-CTSU standard operating procedures to ensure the enduring quality, integrity, and utility of the data. Formal requests for data sharing are considered in line with ICR-CTSU procedures, with due regard given to funder and sponsor guidelines. Requests are to be made via a standard proforma describing the nature of the proposed research and extent of data requirements. Data recipients are required to enter a formal data sharing agreement, which describes the conditions for data release and requirements for data transfer, storage, archiving, publication, and intellectual property. Requests are reviewed by the trial management group in terms of scientific merit and ethical considerations including patient consent. Data sharing is undertaken if proposed projects have a sound scientific or patient benefit rationale, as agreed by the trial management group and approved by the trial steering committee, as required. Restrictions relating to patient confidentiality and consent will be lessened by aggregating and anonymising identifiable patient data. Additionally, all indirect identifiers that may lead to deductive disclosures will be removed in line with Cancer Research UK data sharing guidelines. Additional documents may be shared if approved by the trial management group and trial steering committee—eg, statistical analysis plan and informed consent form.
