# Supplementary material for: Circulating tumour DNA analysis to direct therapy in advanced breast cancer (plasmaMATCH): a multicentre, multicohort, phase 2a, platform trial
Source: Lancet Oncol. 2020 Oct;21(10):1296–308. doi: 10.1016/S1470-2045(20)30444-7 (PMC7599319; doi:10.1016/S1470-2045(20)30444-7)
Supplement: Supplementary appendix [file mmc1.pdf]

THE LANCET
Oncology

Supplementary appendix

This appendix formed part of the original submission and has been peer reviewed. We post it as supplied by the authors.

Supplement to: Turner NC, Kingston B, Kilburn LS, et al. Circulating tumour DNA analysis to direct therapy in advanced breast cancer (plasmaMATCH): a multicentre, multicohort, phase 2a, platform trial. *Lancet Oncol* 2020; published online Sept 10. http://dx.doi.org/10.1016/S1470-2045(20)30444-7.

SUPPLEMENTARY MATERIAL

Circulating tumour DNA analysis to direct therapy in advanced breast cancer (plasmaMATCH): a multicentre, multicohort, phase 2a platform trial

plasmaMATCH Trial Investigators

Hospital	Principal Investigator	Other Trial Investigators	Number of patients registered for ctDNA screening
Royal Marsden Hospital NHS Foundation Trust	Alistair Ring	Nicholas Turner, Alicia Okines, Stephen Johnston, Marina Parton, Zoe Kemp, Charlotte Fribbens, Emma Kipps, Alexandra Pender, Tazia Irfan, Sophie McGrath, Mark Allen, Jill Noble, Nicolo Battisti	328
Christie Hospital, Manchester	Andrew Wardley	Anne Armstrong, Sacha Howell, Juliette Loncaster, Lubna Bhatt, Abbasi Chittalia, Vivek Misra	185
Beatson West of Scotland Cancer Centre	Iain Macpherson	Sarah Slater, Rosie Stevens, Sophie Barrett, Mark Baxter	81
Addenbrooke's Hospital	Richard Baird	Emma Beddowes, Margaret Moody, Charles Wilson, Carlos Caldas, Helena Earl	77
Royal Devon and Exeter Hospital	Peter Stephens	Andrew Goodman, Kate Scatchard, Jenny Forrest, David Hwang, Chris Hamilton	62
Western General Hospital, Edinburgh	Olga Oikonomidou	Larry Hayward, Peter Hall, David Cameron, Helen Creedon, Caroline Michie, Carolyn Bedi, Jane Parry	46
Bristol Haematology and Oncology Centre	Jeremy Braybrooke	Vivek Mohan, Charles Comins, Jessica Jenkins	42
Churchill Hospital, Oxford	Mark Tuthill	Nicola Levitt, Rene Roux, Andrew Eichholz, Bernadette Lavery, Sileida Oliveros	41
University College London Hospitals NHS Foundation Trust	Rebecca Roylance	Uzma Asghar	34
Velindre Hospital, Cardiff	Jacinta Abraham	Simon Waters, Annabel Borley, Helen Passant, Theresa Howe	28
Weston Park Hospital, Sheffield	Matthew Winter	Caroline Wilson, Om Prakash Purohit	22
Royal Bournemouth Hospital	Tamas Hickish	-	20
Royal Cornwall Hospital	Alastair Thomson	Duncan Wheatley	19
St Bartholomew's Hospital, London	Peter Schmid	Melissa Phillips, Virginia Wolstenholme	18
Southampton General Hospital	Ellen Copson	Jennifer Bradbury, Chern Lee, Clare Crowley, Peter Simmonds	17
Derriford Hospital, Plymouth	Rebecca Goranova	Sidharth Dubey, Udaiveer Panwar	13
Maidstone Hospital	Charlotte Abson	Russell Burcombe, Catherine Harper-Wynne	9
Clatterbridge Cancer Centre	Carlo Palmieri	-	8

plasmaMATCH Additional Trial Team Members

**The Institute of Cancer Research – Clinical Trials and Statistics Unit**	
Michelle Frost	Clinical Trials Programme Manager
James Morden	Senior Statistician
Micki Hill	Trial Statistician
Grace Elwood	Data Manager
Joanne Haviland	Principal Statistician
Ellie Grainger	Trial Statistician
Sarah Fynn	Data Manager
Karen Picton	Trial Administrative Officer
Angelica Gomez Roig	Analyst Programmer
Oleg Zhitkov	Data Manager
Vanshika Shama	Trial Manager
Joan Ekajey	Data Manager
Charlotte Emery	Trial Statistician
Abimbola Aderibigbe	Data Manager
**The Institute of Cancer Research- Molecular Oncology**	
Isaac Garcia-Murillas	Staff Scientist
Sarah Hrebien	Higher Scientific Officer
Giselle Walsh	Scientific Officer
Charlotte Fribbens	Clinical Research Fellow
**The Royal Marsden NHS Foundation Trust - BioBank / Biomedical Research Centre**	
Janine Salter	Biobank Manager
Alison MacDonald	Operations and Quality Assurance Manager
Aoife Ruane	Lead Biological Specimen Co-ordinator
Priya Yoganathan	Clinical Development and Improvement Manager
Nhu-Tam Nguyen	Senior Biological Specimen Co-ordinator and Data Manager
James Vango	Senior Biological Specimen Co-ordinator
Gosia Najgebauer	Senior Biological Specimen Co-ordinator
Paula Proszek	Research Scientist

Supplementary methods

Statistical analyses

ICR-CTSU undertook all central statistical monitoring, interim and final analyses.

Blood sampling

30ml of blood was collected in 3 x 10mL cell-free DNA BCT® Streck tubes. Tubes were inverted 8 to 10 times prior to shipment at ambient temperature to the central laboratory (Centre for Molecular Pathology, Royal Marsden, Sutton). At Centre for Molecular Pathology, the sample was spun at 1600g for 10 minutes at room temperature, then plasma removed (above pellet) and spun again at 1600g for 10 minutes at room temperature. The plasma was isolated and aliquoted into 4.5mL cryotubes and stored until analysis at -80℃.

At the commencement of the plasmaMATCH trial, the ctDNA was analysed solely using digital PCR (dPCR) assays at the Centre for Molecular Pathology. From July 2018, an additional 10mL of blood was collected in a cell-free DNA BCT® Streck tube and shipped at ambient temperature to Guardant Health (Redwood City, California, USA) for targeted sequencing. Upon receipt, blood samples were spun at 1600g for 10 minutes at 10℃. Plasma was isolated and further spun at 3220g for 10 minutes at 10℃ prior to plasma aliquoting and storage at 2℃ for immediate processing, or -80℃ for delayed analysis.

Targeted sequencing (Guardant360, Guardant Health) was performed on plasma in parallel to the dPCR analysis undertaken at the Centre for Molecular Pathology, with hotspot mutations identified with either technology making a patient eligible for Cohorts B to D. Cohort A continued to recruit solely on the basis of the dPCR results to facilitate the exploratory assessment of *ESR1* clonality.

Digital PCR analysis

DNA was extracted from 8mL of screening plasma using the automated QiaSymphony platform (Qiagen, Hombrechtikon), and eluted into 120uL buffer, respectively, followed by Qubit quantification (Thermo Scientific). DNA was tested for hotspot mutation status using multiplex and singleplex assays. *PIK3CA* hotspot mutations in E542K (*1624G>A*), E545K (*1633G>A*), H1047R (*3140A>G*) and H1047L (*3140A>T*) were assayed with a PIK3CA multiplex (sequence previously published[1]), and two *ESR1* multiplex assays targeting E380Q (*c.1138G>C*), L536R (*c.1607T>G*), Y537C (*c.1610A>G*), D538G (*c.1613A>G*) (dHsaMDXE91450042) and S463P (*c.1387T>C*), Y537N (*c.1609T>A*), Y537S (*c.1610A>C*) (dHsaMDXE65719815). Singleplex droplet digital (dPCR) assays target the *AKT1* hotspot mutation E17K (*c.49G>A*), *HER2* (also known as *ERBB2*) hotspot mutations S310F (*c.929C>T*), S310Y (*c.929C>A*), L755S (*c.2264T>C*), V777L (*c.2329G>T*), and insertions *P780_Y781insGSP* (c.2339_2340ins), and *A775_G776insYVMA* (c.2325_2326ins). DNA extracted from 0.5ml plasma, nuclease free water, supermix and the respective assay were combined and partitioned into droplets using an Automated Droplet Generator (QX200 AutoDG). The samples underwent 40 cycles of PCR on a thermal cycler (C1000 Touch Thermal Cycler) prior to analysis on a QX200 Digital Reader. dPCR assays were considered positive when there were a minimum of 10,000 total droplets per well, with two or more positive FAM droplets (mutant), and >300 VIC droplets (wild type). Each positive result was repeated in a separate assay, with a second positive result required for validation. Mutation copies per mL and allele frequency (AF) were calculated using Poisson probability based on the ratio mutant to wild-type ratio of droplets and plasma mL equivalent of DNA assayed.

Targeted sequencing

The Guardant360 targeted panel identifies single nucleotide variants (SNVs), indels, copy number alterations and fusions within protein-coding regions of 74 genes, including *ESR1, HER2, AKT1, PTEN*, and *PIK3CA.* Cell-free DNA was extracted from plasma using the QIAamp Circulating Nucleic Acid Kit (Qiagen) according to the manufacturer's instructions, followed by quantification. Cell-free DNA subsequently underwent library preparation, described in Lanman *et al*[2]. In brief, libraries created from individually barcoded DNA strands (IDT, Inc.) were enriched by hybrid capture (Agilent Technologies, Inc.), pooled, and sequenced using paired-end synthesis (NextSeq 500 and/or HiSeq 2500, Illumina, Inc.). Mutations in *HER2, AKT1* and *PTEN* were assessed by a member of the Trial Management Group (TMG), and only mutations considered to be pathogenic were considered for entry into the respective treatment cohort.

For patients who entered prior to prospective Guardant360 sequencing, frozen aliquots of plasma were shipped to Guardant. No results were available in 237 patients as there was insufficient plasma to ship for retrospective testing. Results of retrospective targeted sequencing were not used to assess eligibility for cohort entry.

Determination of cancer fraction and subclonality from targeted sequencing

For the prespecified analysis of *ESR1* mutation subclonality in cohort A, clonally dominant versus sub-clonal mutations were identified in targeted sequencing.

The proportion of total plasma DNA that originates from the cancer (ctDNA) is highly variable, determined in part by tumour bulk and proliferation[3]. In some patients nearly all of the total plasma DNA originates from the cancer, and in other patients only a small minority of the total plasma DNA originates from the cancer. This substantial variation in the 'purity' of ctDNA in plasma DNA, complicates the assessment of subclonality of mutations in ctDNA sequencing. To assess whether mutations were clonally dominant in the cancer (present in all or most cancer cells) or subclonal (present in a minority of cancer cells), it is necessary to correct for this variation in ctDNA purity between patients, by expressing the allele fraction of *ESR1* mutations relative to the maximum allele fraction in the cancer, assuming that the maximum allele fraction is likely to represent a clonal mutation, and that this will adjust for ctDNA purity.

The cancer fraction of *ESR1* mutation was calculated by summing the allele fractions of all the pathogenic *ESR1* mutations identified, and expressing this relative to the maximum allele fraction identified in the sample. *ESR1* mutations with a cancer fraction >=50% were termed clonally dominant and with cancer fraction <50% were termed subclonal. If only a single mutation was identified, or no mutation, or the maximum allele fraction within the sample was <1% (preventing accurate comparison with maximum allele fraction), the clonal dominance was unknown. Samples with an *ESR1* mutation detected by digital PCR, with no *ESR1* mutation identified in targeted sequencing, were classified as subclonal. Criteria for clonal dominance were determined prior to association with clinical outcome, using previously established criteria[4].

Tissue sequencing

Either a fresh recurrent tumour biopsy or an archival formalin fixed biopsy of recurrent tumour was used for tissue sequencing in patients who entered a treatment cohort. Fresh tumour biopsies were obtained with image guidance where necessary, with two cores fixed in neutral buffered formalin and two cores fresh frozen. The site of biopsy was decided by the investigator.

Fresh frozen and FFPE tissue sections were assessed by a pathologist for tumour content. Tissues with a minimum of 20% tumour content had 10um sections cut, slides were then microdissected according to the pathologist's review, and DNA and RNA extracted using Qiagen AllPrep DNA/RNA Mini Kit (Qiagen, Maryland) or QIAamp DNA/RNA FFPE Tissue Kit as per the manufacturer's instructions for fresh frozen and FFPE samples, respectively. Eluted DNA was stored at −20°C until quantification using Qubit.

Sequencing libraries were prepared using a custom targeted amplicon panel, BCPv10.2. Libraries were made following established methods previously described elsewere[5] after which the libraries were quantified and sequenced on an illumina (Illumina, Inc.) MiniSeq using a mid-output cartridge.

The custom-designed targeted amplicon panel, BCPv10.2. Whole gene and genome regions included in the panel are indicated:

Genes	Genome Regions
CDH1	AKT1
GATA3	BRAF
MAP2K4	HER2
MAP3K1	ESR1
NF1	KIT
	KRAS
	PIK3CA
	PIK3R1
	RUNX1
	SF3B1

	TP53

Sequencing data was analysed using an in-house Ampliseq analysis pipeline, VariTAS. Briefly, reads were aligned to the human genome version 37 using BWA[6], coverage calculation and quality control was completed with Picard tools[7], followed by variant calling with Mutect2[8]. An additional post filtering step was carried out to retain only high quality calls for further validation. First overlapping reads were collapsed with maximum stringency using bbmerge[9]. Samtools pileup was used to count the number of reads for each base at each position in the panel and counts were adjusted using a background set of tumour samples[10]. Variants were filtered for a minimum of 5 alternative reads, minimum depth of 100 and a minimum VAF of 0.01 and only retained if there were alternative reads on both strands. All calls were annotated using Annovar[11]. Additionally variant calls were manually curated using IGV[12].

A biopsy was determined to be contemporaneous if there was <60 days between date of tissue biopsy and plasma sampling for ctDNA analysis.

Comparison of digital PCR and targeted sequencing in ctDNA

Binary (gene-level) plasma mutation agreement between dPCR and targeted sequencing was assessed with a kappa score and 95% confidence interval. Mutation allele frequency was compared between gene groups using Kruskal-Wallis test. Mutation allele frequency of positive concordant versus discordant groups was compared using Kruskal-Wallis test.

Supplementary information on cohort design

Design of Cohort A

Cohort A (protocol v1.2 dated 18th August 2016) was originally designed with a Simon Minimax 2-stage design to detect a target response rate of at least 25% and an unacceptable response rate of 10% in a population of patients assumed to have predominantly clonal mutations thus requiring a sample size of 40 patients. Assuming alpha=0.05, beta=0.2, p0=0.1, p1=0.25, if 3 or more patients respond out of 22 patients in the first stage, the cohort would continue to the second stage and a further 18 patients would be recruited. 8 or more responses out of the total of 40 patients would have been required to infer a level of efficacy for fulvestrant in this cohort of patients.

However, as the trial progressed and before formal consideration of stage 1 it became apparent that the ctDNA testing was likely detecting sub-clonal *ESR1* mutations as well, for whom the response rate to fulvestrant would be expected to be lower (assumed 15%). With agreement from the IDMC and TSC the sample size for Cohort A was therefore extended via protocol amendment (protocol v3.0 dated 01st May 2018) to 78 patients in order to account for the presence of sub-clonal and clonal *ESR1* mutations in patient population (sample size justification provided in main paper) with the expectation that each would have a relative frequency of 50%. The clinical rationale for this noted that the treatment delivered in Cohort A utilized a drug which constituted a standard of care option for these patients and that the dose being investigated was in fact higher than that which would usually be prescribed thus that patients entered into Cohort A were not disadvantaged compared with standard of care.

Recruitment into Cohort A was temporarily suspended on 17th February 2018 after the original sample size had been met, pending approval of the protocol amendment to extend the sample size. Recruitment into Cohort A re-opened on 19th June 2018 following approval of protocol v3.0. Cohort A permanently closed on 17th April 2019 once the extended sample size had been met.

Design of Cohort D

Cohort D was a basket cohort allowing entry of patients with both ctDNA testing results and tissue sequencing results. The full list of allowable mutations were; *AKT1* mutations identified via ctDNA screening in patients with ER negative BC; *AKT1* mutations identified in tumour sequencing conducted outside of plasmaMATCH in patients with ER positive or negative BC; *AKT2/3* E17K, *PIK3R1* or *PTEN* mutations or homozygous deletion of *PTEN* in both ER positive and ER negative BC identified in ctDNA screening or in prior tumour sequencing conducted outside of plasmaMATCH.

Supplementary Tables

Table S1 Reasons for not going into a cohort by cohort/actionable mutation

	**Cohort A (*ESR1*)**	**Cohort B (*HER2*)**	**Cohort C (*AKT1* & HR+)**	**Cohort D (*AKT1* & HR- or *PTEN*)**	**Total**
Ineligible	52*	9*	4+	5	67
Patient choice	19+$	0	2+	4$	22
Clinician decision	33*+	2*	4+	3	40
Cohort suspended/closed	64	0	3	3	70
Patient died	5	0	2	1	8
Unknown	11	2	0	1	14

* 1 patient had an *ESR1* and *HER2* mutation
+ 1 patient had an *ESR1* and *AKT1* mutation
$ 2 patients had an *ESR1* and *AKT1*/*PTEN* mutation

Table S2 Reasons for treatment discontinuations by cohort and details on dose reductions and delays

	Cohort A (n=76)		Cohort B (n=17)		Cohort C (n=14)		Cohort D (n=17)	
	**n**	**%**	**n**	**%**	**n**	**%**	**n**	**%**
Disease progression	72	94.7	16	94.1	11	84.6	15	83.3
Adverse or serious adverse event	1[a]	1.3	1[b]	5.9	2[c1]	7.7	3[d]	16.7
Patient choice	2	2.6	0	0.0	0	0.0	0	0.0
Death	1	1.3	0	0.0	0	0.0	0	0.0
Other	0	0.0	0	0.0	1[c2]	7.7	0	0.0

[a]>21 days of grade 3/4 toxicities - raised alkaline phosphatase and GGT

[b] Haematemesis (Grade 4): Shortness of breath (Grade 1): Confusion (Grade 2): Seizure (vacant episode)(Grade 2)

[c1] One patient - treatment stopped due to worsening dyspnoea. This led to respiratory failure and then death. Other patient stopped due to grade 3 vomiting and diarrhoea.

[c2] Stable by RECIST but little normal liver left and little chance of delivering a meaningful dose of capivasertib.

[d] One patient had face swelling and vomiting; one patient had grade 3 rash and fever; one patient had grade 3 rash

Table S3 Treatment emergent adverse effects of extended dose fulvestrant (Cohort A)

Note: AEs reported in the table below are where the AE was reported for ≥10% patients for any grade at baseline to an increase in grade on treatment or where at least one patient has an AE graded 0-2 at baseline but a grade 3+ on treatment.

N=80	Any grade at baseline to at least one grade increase on treatment		G0-2 at baseline to G3+ on treatment	
	n	%	n	%
Adrenal insufficiency	1	1.3	1	1.3
Anaemia	28	35.0	5	6.3
Arthralgia	13	16.3	1	1.3
Back pain	6	7.5	1	1.3
Blood alkaline phosphatase increased	19	23.8	5	6.3
Blood bilirubin increased	6	7.5	1	1.3
Cancer pain	1	1.3	1	1.3
Constipation	12	15.0	0	0.0
Cough	10	12.5	0	0.0
Diarrhoea	11	13.8	0	0.0
Dyspnoea	14	17.5	0	0.0
Fatigue	17	21.3	0	0.0
Femur fracture	1	1.3	1	1.3
Gamma-glutamyltransferase increased	29	36.3	13	16.3
Groin pain	2	2.5	1	1.3
Headache	9	11.3	0	0.0
Hypercalcemia	4	5.0	2	2.5
Hyperglycaemia	9	11.3	0	0.0
Hypertension	21	26.3	10	12.5
Hypoalbuminemia	9	11.3	1	1.3
Hypokalaemia	8	10.0	1	1.3
Hyponatremia	4	5.0	2	2.5
Injection site reaction	12	15.0	0	0.0
Intracranial venous sinus thrombosis	1	1.3	1	1.3
Leukopenia	17	21.3	1	1.3
Lung infection	1	1.3	1	1.3
Lymphopaenia	18	22.5	5	6.3
Muscle spasms	8	10.0	0	0.0
Musculoskeletal pain	12	15.0	0	0.0
Nausea	18	22.5	0	0.0
Neutropenia	14	17.5	3	3.8
Pain in extremity	2	2.5	1	1.3
Pruritus	10	12.5	0	0.0
Pyrexia	2	2.5	1	1.3
Superior sagittal sinus thrombosis	1	1.3	1	1.3
Tachycardia	9	11.3	0	0.0
Thrombocytopenia	8	10.0	0	0.0
Transaminases increased	23	28.8	2	2.5
Vomiting	13	16.3	0	0.0

Table S4 Pharmacokinetic analysis of extended dose fulvestrant in Cohort A

Comparison of extended-dose fulvestrant PK with population PK model for standard schedule fulvestrant (standard popPK). CV coefficient of variation

	n	Fulvestrant mg/dl**	CV%	% increase from standard popPK
Cycle 2 day 1	38	17.1	29.5	12.4
Cycle 3 day 1	32	19.1	21.2	70.8
Cycle 4 day 1	20	21.4	20.5	66.1

Table S5 Treatment emergent adverse effects of neratinib with and without fulvestrant (Cohort B)

Note: AEs reported in the table below are where the AE was reported for ≥10% patients for any grade at baseline to an increase in grade on treatment or where at least one patient has an AE graded 0-2 at baseline but a grade 3+ on treatment.

N=20	Any grade at baseline to at least one grade increase on treatment		G0-2 at baseline to G3+ on treatment	
	n	%	n	%
Abdominal distension	4	20.0	0	0.0
Abdominal pain	2	10.0	0	0.0
Alopecia	2	10.0	0	0.0
Anaemia	6	30.0	1	5.0
Arthralgia	3	15.0	0	0.0
Back pain	5	25.0	0	0.0
Blood alkaline phosphatase increased	5	25.0	0	0.0
Blood bilirubin increased	3	15.0	0	0.0
Cellulitis	1	5.0	1	5.0
Constipation	6	30.0	1	5.0
Cough	4	20.0	0	0.0
Decreased appetite	8	40.0	0	0.0
Device related infection	1	5.0	1	5.0
Diarrhoea	15	75.0	4	20.0
Dizziness	3	15.0	0	0.0
Dry mouth	2	10.0	0	0.0
Dry skin	2	10.0	0	0.0
Dyspepsia	3	15.0	0	0.0
Dyspnoea	3	15.0	0	0.0
Epistaxis	2	10.0	0	0.0
Fatigue	5	25.0	0	0.0
Gamma-glutamyltransferase increased	3	15.0	3	15.0
Haematemesis	1	5.0	1	5.0
Hand fracture	1	5.0	1	5.0
Headache	2	10.0	0	0.0
Hot flush	2	10.0	0	0.0
Hypertension	7	35.0	3	15.0
Hypoalbuminemia	2	10.0	0	0.0
Kidney infection	1	5.0	1	5.0
Leukopenia	3	15.0	0	0.0
Lymphopaenia	4	20.0	0	0.0
Mouth ulceration	3	15.0	0	0.0
Muscle spasms	2	10.0	0	0.0
Muscular weakness	2	10.0	0	0.0
Musculoskeletal pain	3	15.0	0	0.0
Nasopharyngitis	2	10.0	0	0.0
Nausea	7	35.0	1	5.0
Neutropenia	4	20.0	0	0.0
Oedema peripheral	2	10.0	0	0.0
Pleural effusion	2	10.0	1	5.0
Pruritus	3	15.0	0	0.0
Rash	5	25.0	1	5.0
Tachycardia	5	25.0	0	0.0

N=20	Any grade at baseline to at least one grade increase on treatment		G0-2 at baseline to G3+ on treatment	
	n	%	n	%
Transaminases increased	5	25.0	0	0.0
Urinary tract infection	3	15.0	1	5.0
Vomiting	4	20.0	1	5.0

Four serious adverse reactions reported in 3 patients; 1 patient - grade 3 nausea, vomiting and constipation; 1 patient - grade 2 shortness of breath on exertion and hypoxia on exertion followed by haematemesis (grade 4), shortness of breath (grade 1), confusion (grade 2), seizure (vacant episode) (grade 2); 1 patient - grade 3 urinary tract infection.

Table S6 Adverse effects of capivasertib with fulvestrant (Cohort C)

Note: AEs reported in the table below are where the AE was reported for ≥10% patients for any grade at baseline to an increase in grade on treatment or where at least one patient has an AE graded 0-2 at baseline but a grade 3+ on treatment.

N=18	Any grade at baseline to at least one grade increase on treatment		G0-2 at baseline to G3+ on treatment	
	n	%	n	%
Abdominal pain	2	11.1	0	0.0
Anaemia	6	33.3	0	0.0
Arthralgia	2	11.1	0	0.0
Back pain	4	22.2	0	0.0
Bacterial infection	1	5.6	1	5.6
Blood albumin decreased	2	11.1	0	0.0
Blood alkaline phosphatase increased	3	16.7	0	0.0
Constipation	3	16.7	0	0.0
Cough	2	11.1	0	0.0
Decreased appetite	4	22.2	0	0.0
Dehydration	2	11.1	0	0.0
Diarrhoea	13	72.2	2	11.1
Dizziness	3	16.7	0	0.0
Dyspepsia	2	11.1	0	0.0
Dyspnoea	3	16.7	1	5.6
Fatigue	9	50.0	4	22.2
Gamma-glutamyltransferase increased	3	16.7	2	11.1
Headache	3	16.7	0	0.0
Hyperglycaemia	10	55.6	2	11.1
Hypertension	2	11.1	1	5.6
Hypoalbuminemia	4	22.2	0	0.0
Hypoglycaemia	3	16.7	0	0.0
Hypokalaemia	3	16.7	0	0.0
Hyponatremia	1	5.6	1	5.6
Hypotension	1	5.6	1	5.6
Infection	1	5.6	1	5.6
Influenza like illness	2	11.1	0	0.0
Leukopenia	2	11.1	1	5.6
Lymphopaenia	8	44.4	2	11.1
Muscle spasms	2	11.1	0	0.0
Nausea	8	44.4	0	0.0
Neck pain	1	5.6	1	5.6
Neutropenia	1	5.6	1	5.6
Oral pain	2	11.1	0	0.0
Pain in extremity	2	11.1	0	0.0
Pleural effusion	1	5.6	1	5.6
Pneumocystis jirovecii infection	1	5.6	1	5.6
Pruritus	2	11.1	0	0.0
Pulmonary embolism	1	5.6	1	5.6
Rash	6	33.3	3	16.7
Staphylococcal infection	1	5.6	1	5.6
Stomatitis	2	11.1	0	0.0
Transaminases increased	3	16.7	1	5.6

N=18	Any grade at baseline to at least one grade increase on treatment		G0-2 at baseline to G3+ on treatment	
	**n**	**%**	**n**	**%**
Urinary tract infection	5	27.8	0	0.0
Vaginal discharge	2	11.1	0	0.0
Vomiting	7	38.9	1	5.6
Weight decreased	2	11.1	0	0.0

Eight serious adverse reactions reported in 4 patients: 1 patient – grade 3 diarrhoea and vomiting on two separate occasions; 1 patient – grade 4 dyspnoea (treatment-related death); 1 patient – migraine (grade 2), acute kidney injury (grade 1), hyperglycaemia (grade 4), followed by fatigue (grade 3), nausea (grade 2), abdominal bloating (grade 2), then followed by gram positive cocci on peripheral line blood cultures (grade 3); 1 patient – vomiting (grade 2), hyperglycaemia (grade 3) followed by increasing peripheral neuropathy (grade 2), PV loss (grade 2):

Table S7 Adverse effects of capivasertib (Cohort D)

Note: AEs reported in the table below are where the AE was reported for ≥10% patients for any grade at baseline to an increase in grade on treatment or where at least one patient has an AE graded 0-2 at baseline but a grade 3+ on treatment.

N=19	Any grade at baseline to at least one grade increase on treatment		G0-2 at baseline to G3+ on treatment	
	n	%	n	%
Abdominal pain	3	15.8	0	0.0
Anaemia	4	21.1	0	0.0
Arthralgia	2	10.5	0	0.0
Back pain	3	15.8	0	0.0
Cough	2	10.5	0	0.0
Decreased appetite	2	10.5	0	0.0
Diarrhoea	12	63.2	0	0.0
Dyspepsia	2	10.5	0	0.0
Fatigue	5	26.3	0	0.0
Gamma-glutamyltransferase increased	4	21.1	2	10.5
Headache	2	10.5	0	0.0
Hyperglycaemia	9	47.4	1	5.3
Hypertension	3	15.8	2	10.5
Hypokalaemia	4	21.1	1	5.3
Leukopenia	4	21.1	0	0.0
Lymphopaenia	4	21.1	1	5.3
Mucosal inflammation	2	10.5	0	0.0
Nausea	7	36.8	0	0.0
Neutropenia	6	31.6	0	0.0
Pruritus	1	5.3	1	5.3
Pyrexia	3	15.8	0	0.0
Rash	6	31.6	5	26.3
Transaminases increased	2	10.5	2	10.5
Vomiting	6	31.6	2	10.5

Four serious adverse reactions reported in three patients: 1 patient – grade 3 rash, 1 patient - maculopapular rash (grade 3), vomiting (grade 2), diarrhoea (grade 2) followed by rash (grade 2), pyrexia (grade 2), swelling of nose and lips (grade 2); 1 patient – rash (grade 3), fever (grade 2), hypotensive (grade 2).

Table S8 Response rates with 90%CI by cohort

Mutation	Cohort	Confirmed response rate, % (90%CI; n/N)	
		All patients	First 16 evaluable patients
ESR1	A	8.1% (3.6-15.4; 6/74)	-
Clonal		12.2% (4.9-23.9;5/41)	-
Sub-clonal		0% (0-10.5;0/27)	-
HER2	B	25.0% (10.4-45.6; 5/20)	25.0% (9.0-48.4; 4/16)
AKT1	C	22.2% (8.0-43.9; 4/18)	18.8% (5.3-41.7; 3/16)
AKT basket	D	10.5% (1.9-29.6; 2/19)	12.5% (2.3-34.4; 2/16)
AKT1		33.3% (6.3-72.9; 2/6)	-
PTEN		0.0% (0-20.6; 0/13)	-

Supplementary Figures

Figure S1 Clinical ctDNA testing in advanced breast cancer

A. Design of the plasmaMATCH trial

B. Allele fractions of potentially targetable mutations identified in 1025 patients with digital PCR ctDNA testing results.

C. *Left* Agreement between ctDNA digital PCR and targeted sequencing with kappa scores, *right* allele fraction of concordant mutations compared to allele fraction of discordant mutations.

D. Agreement between digital PCR ctDNA testing and advanced disease tissue sequencing, *left* overall and *right* in patients with contemporaneous biopsies taken with 60 days of the screening plasma.

dPCR – digital PCR, TS – targeted sequencing

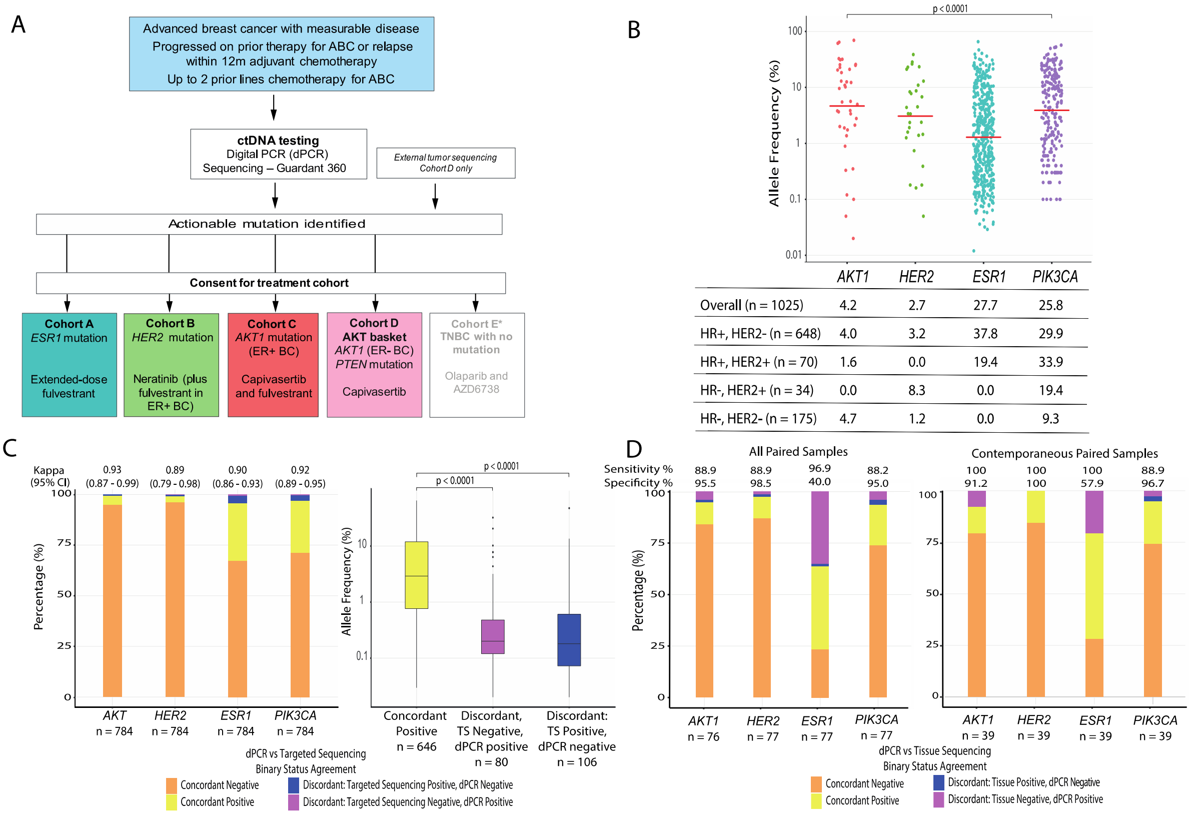

	AKT1	HER2	ESR1	PIK3CA
Overall (n = 1025)	4.2	2.7	27.7	25.8
HR+, HER2- (n = 648)	4.0	3.2	37.8	29.9
HR+, HER2+ (n = 70)	1.6	0.0	19.4	33.9
HR-, HER2+ (n = 34)	0.0	8.3	0.0	19.4
HR-, HER2- (n = 175)	4.7	1.2	0.0	9.3

Figure S2 Mutations detected by digital PCR and by sequencing in ctDNA

A. *Left* Mutation frequencies identified by digital PCR and *right* mutation frequencies identified by sequencing
B. Rate of single versus multiple mutations identified by targeted sequencing

A

Alteration	digital PCR					Targeted sequencing				
	All	HR+ HER2-	HR+ HER2+	HR- HER2+	TNBC	All	HR+ HER2-	HR+ HER2+	HR- HER2+	TNBC
	%	%	%	%	%	%	%	%	%	%
	n = 1025	n = 659	n = 64	n = 36	n = 174	n = 800	n = 515	n = 46	n = 26	n = 138
PIK3CA mutation	25.7	30.0	31.3	19.4	9.2	34.9	39.2	41.3	34.6	14.5
ESR1 mutation	27.7	38.2	12.5	0	0.6	33.1	43.9	17.4	0	1.4
AKT1 mutation	4.2	3.6	1.6	0	5.7	5.0	4.9	2.2	0	5.8
HER2 mutation	2.7	3.0	1.6	8.3	1.1	6.4	6.4	15.2	15.4	2.2

B

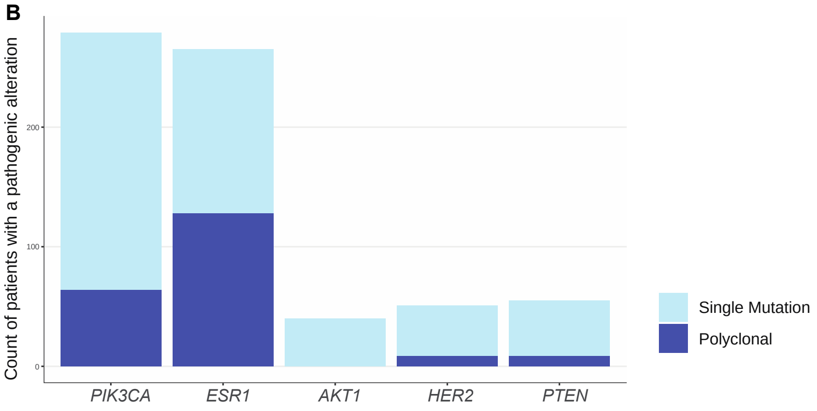

Figure S3 Agreement between digital PCR and sequencing ctDNA analysis

A. Individual mutation agreement for *ESR1*
B. Individual mutation agreement for *HER2*
C. Individual mutation agreement for *AKT1*
D. Individual mutation agreement for *PIK3CA*
E. Proportion of positive calls overall which are concordant or discordant

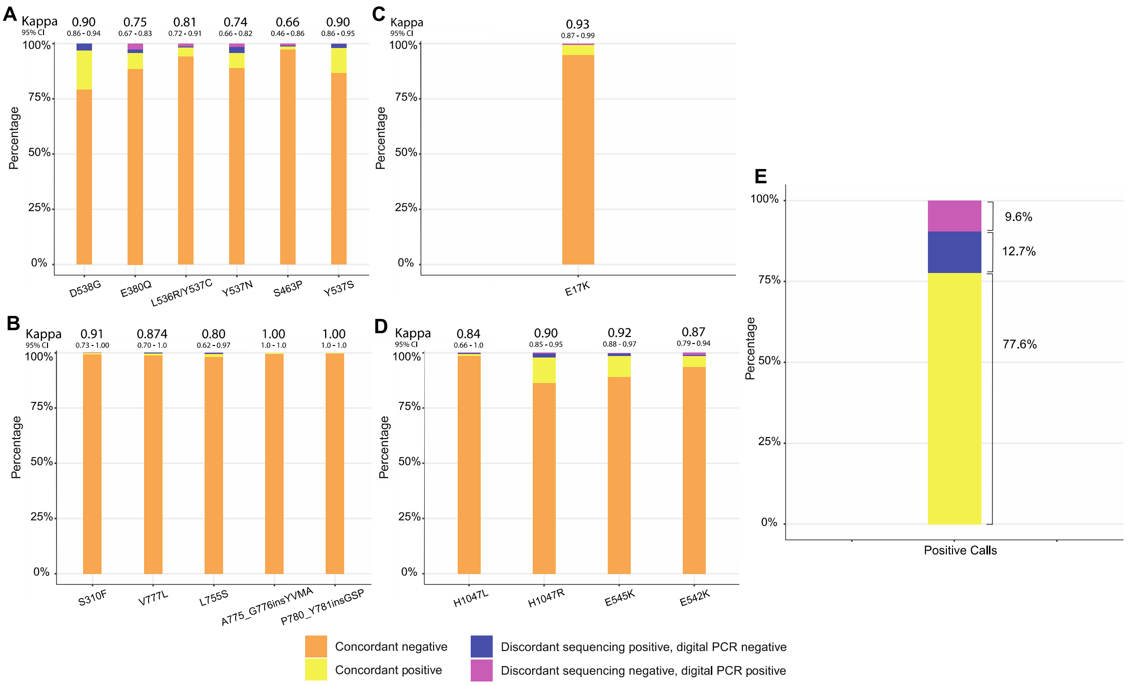

Figure S4 Agreement between digital PCR ctDNA testing and advanced disease tissue sequencing
Agreement in all paired samples (*left*), contemporaneous ctDNA and tissue samples (middle), and time discordant samples where tissue was taken >=60 days prior to ctDNA sample.

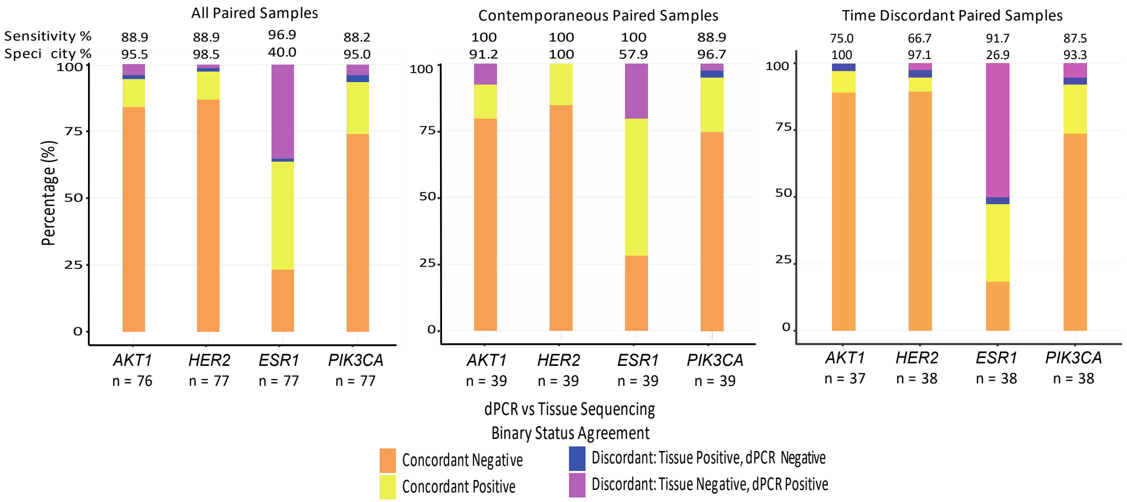

Figure S5 Agreement between tissue sequencing and ctDNA targeted sequencing analysis

All ctDNA targeted sequencing and tissue results

Contemporaneous ctDNA targeted sequencing and tissue results

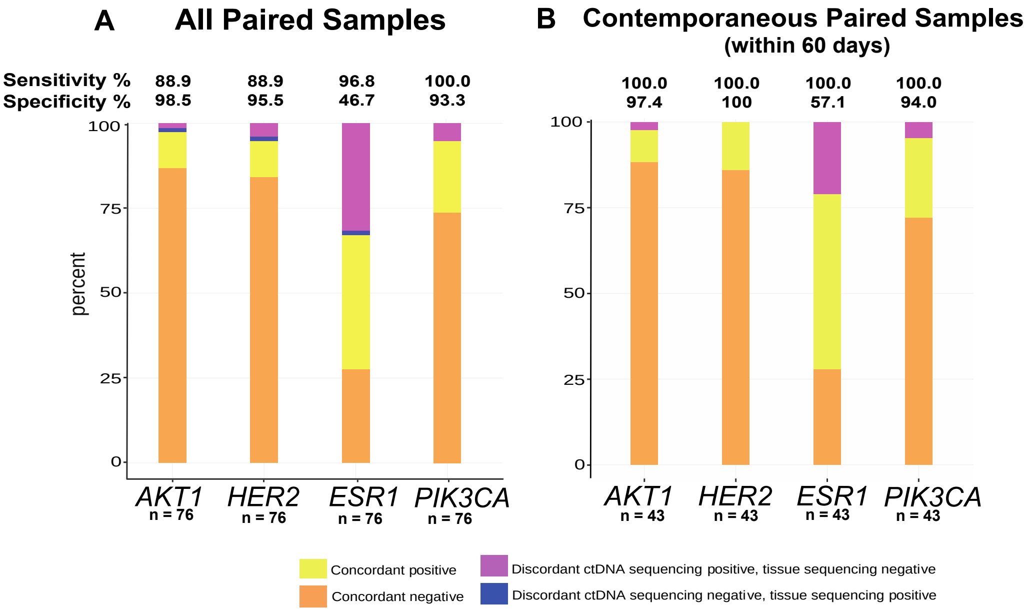

Figure S6: Progression free survival of patients with *ESR1* mutations in ctDNA treated with extended-dose fulvestrant.

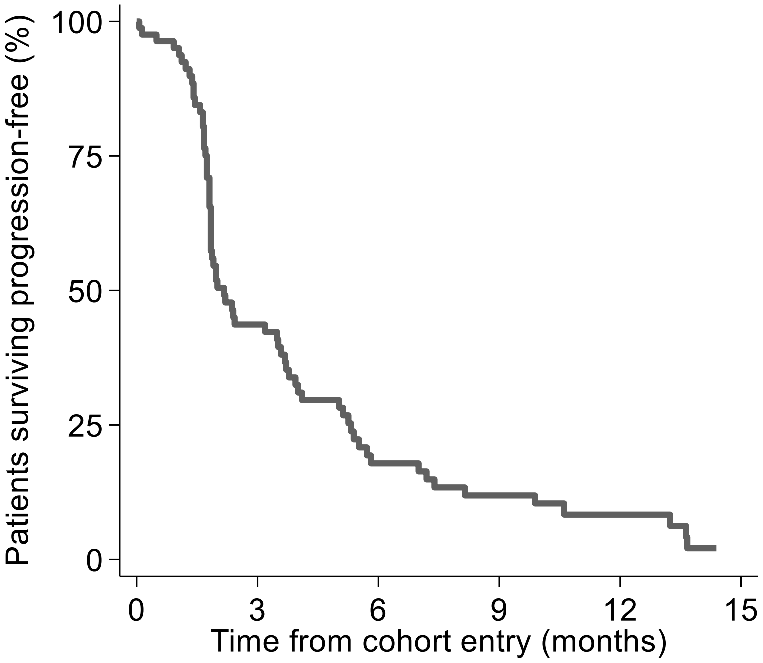

Number at risk (number censored)

84 (10) 32 (12) 12 (12) 8 (14) 4 (15) 0

Figure S7: Progression free survival of patients with *HER2* mutations in ctDNA treated with neratinib with or without fulvestrant.

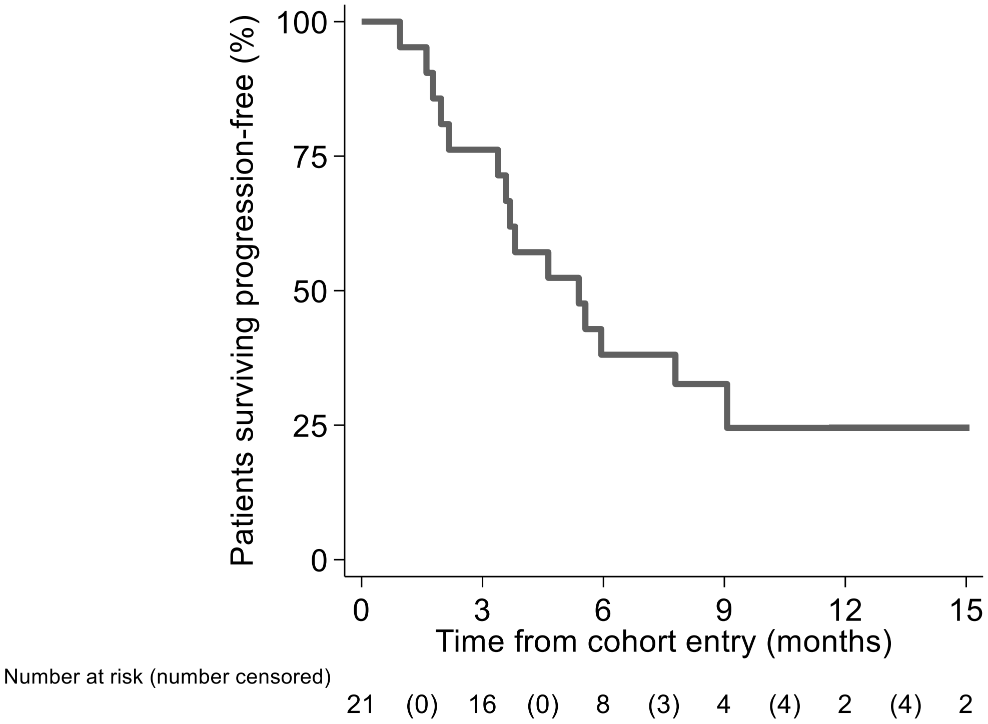

Figure S8 Analysis of subclonality in Cohorts B and C.

Left Cohort B: In exploratory analysis the response rate was 26.7% (4/15, 95%CI,7.8-55.1%) with clonally dominant *HER2* mutations and 33.3% (1/3, 95%CI,0.8-90.6%) with sub-clonal mutations (p=1.00) ; two patients had unknown clonality.

Right Cohort C: In exploratory analysis the response rate as 23.1% (3/13, 95%CI,5.0-53.8%) with clonally dominant *AKT1* mutations and 25.0% (1/4, 95%CI,0.6-80.6%) with sub-clonal mutations (p=1.00); one patient had unknown clonality. These analyses were not pre-planned.

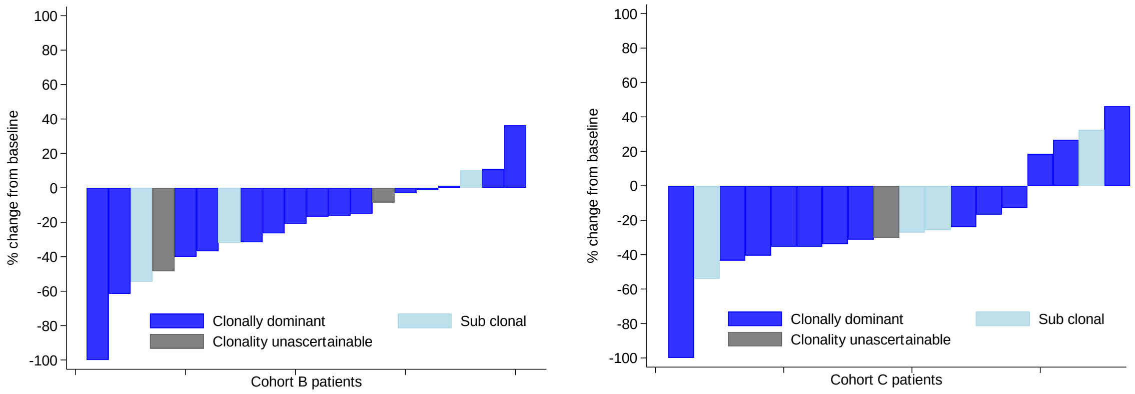

Figure S9: Progression free survival of patients, with HR+ cancer and with *AKT1* mutations in ctDNA, treated with capivasertib plus fulvestrant (Cohort C).

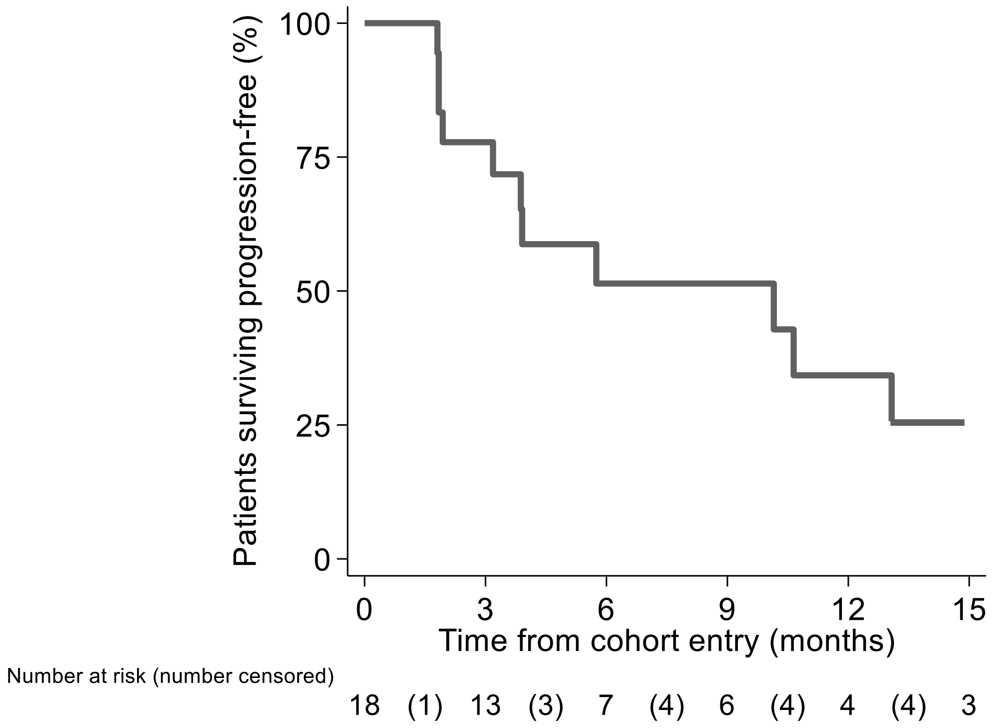

Figure S10: Progression free survival of patients in Cohort D treated with capivasertib.

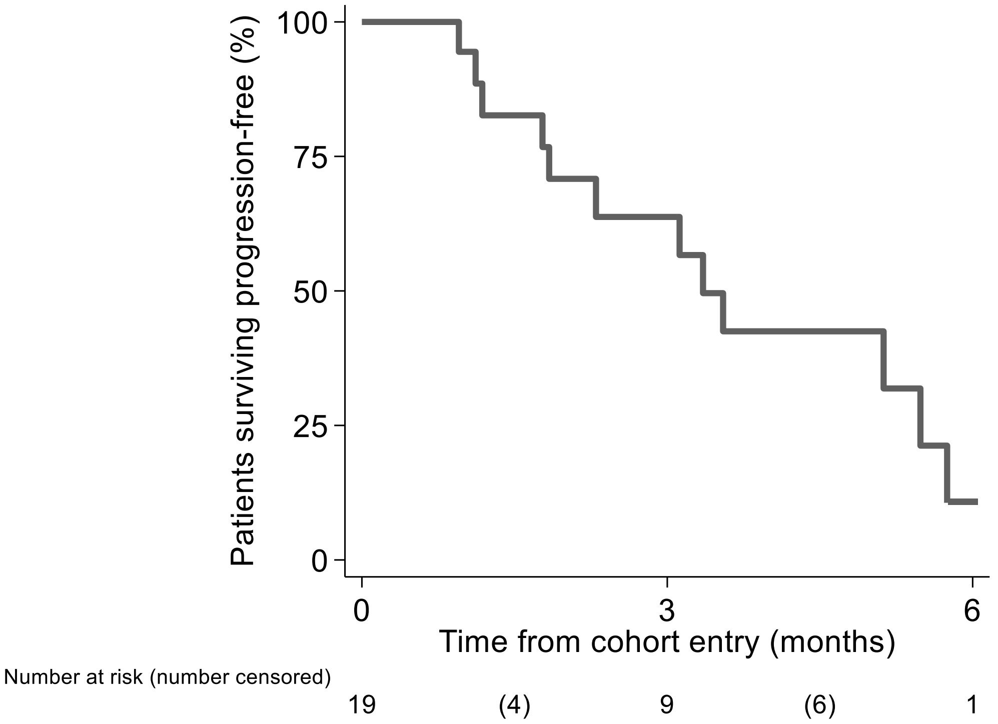

Figure S11 *PIK3CA* mutation status and efficacy of targeted therapy by cohort

Waterfall plot of maximum change in tumour size in patients, split by *PIK3CA* mutation status in baseline ctDNA. Response rates %, (n/N, 95%CI): Cohort A: Mutant 3.8% (1/26, 0.1-19.6), Wild type 7.0% (3/43, 1.5-19.1, p=1.00). Cohort B: Mutant 40.0% (4/10, 12.2-73.8), Wild type 10.0% (1/10, 2.5-44.5, p=0.30). Cohort C: Mutant 66.7% (2/3, 9.4-99.2), Wild type 13.3% (2/15, 1.7-40.5, p=0.11). Cohort D: Mutant 0.0% (0/1, -), Wild type 11.8% (2/17, 1.5-36.4). These analyses were not pre-planned.

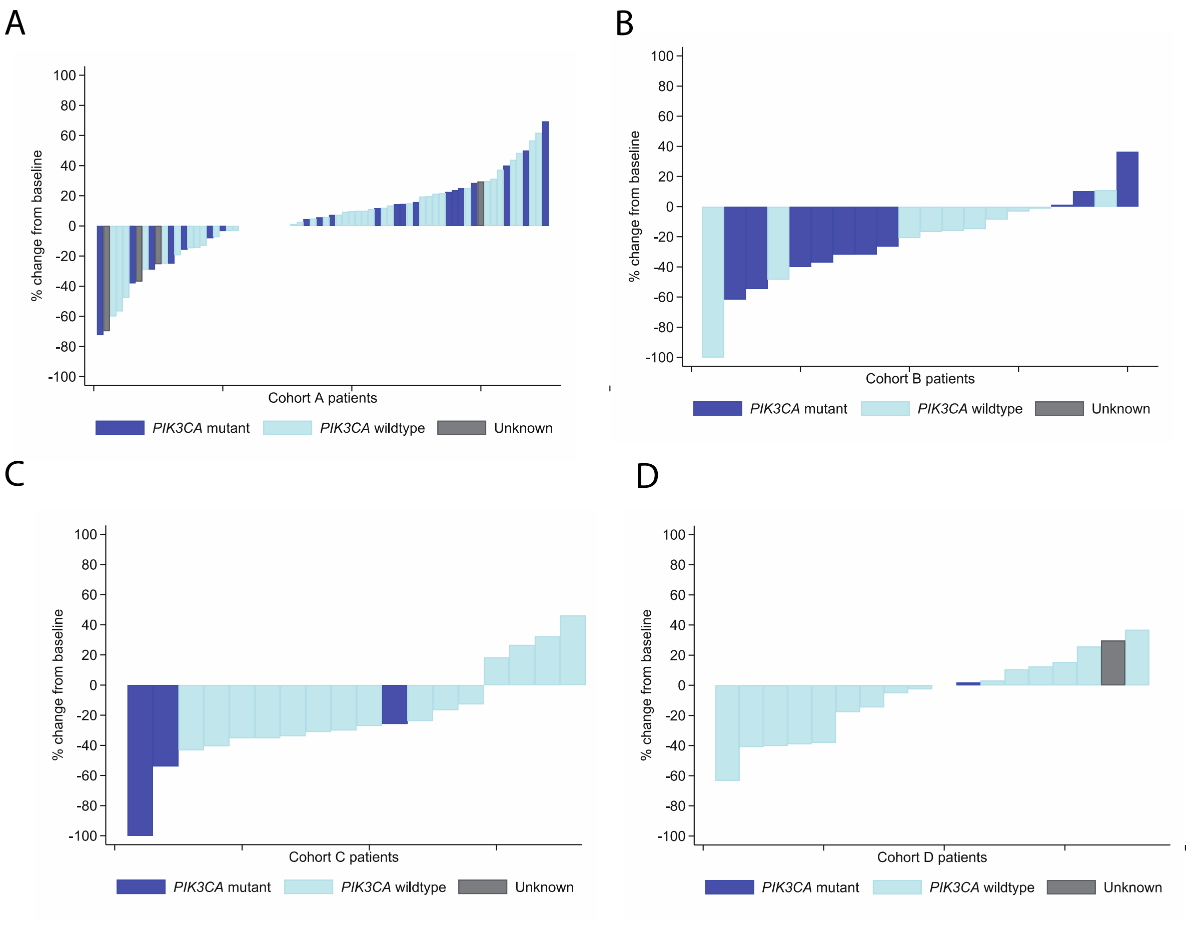

Figure S12 *TP53* mutation status and efficacy of targeted therapy by cohort

Waterfall plot of maximum change in tumour size in patients, split by *TP53* mutation status in baseline ctDNA. Response rates %, (n/N, 95%CI): Cohort A: Mutant 0.0% (0/22, -), Wild type 8.5% (4/47, 2.4-20.4, p=0.30). Cohort B: Mutant 33.3% (3/9, 7.5-70.1), Wild type 18.2% (2/11, 2.3-51.8, p=0.62). Cohort C: Mutant 36.4% (4/11, 10.9-69.2), Wild type 0.0% (0/7, -, p=0.12). Cohort D Mutant 14.3% (2/14, 1.8-42.8), Wild type 0.0% (0/4, -, p=1.00). These analyses were not pre-planned, *TP53* mutations were analysed in the ctDNA targeted sequencing.

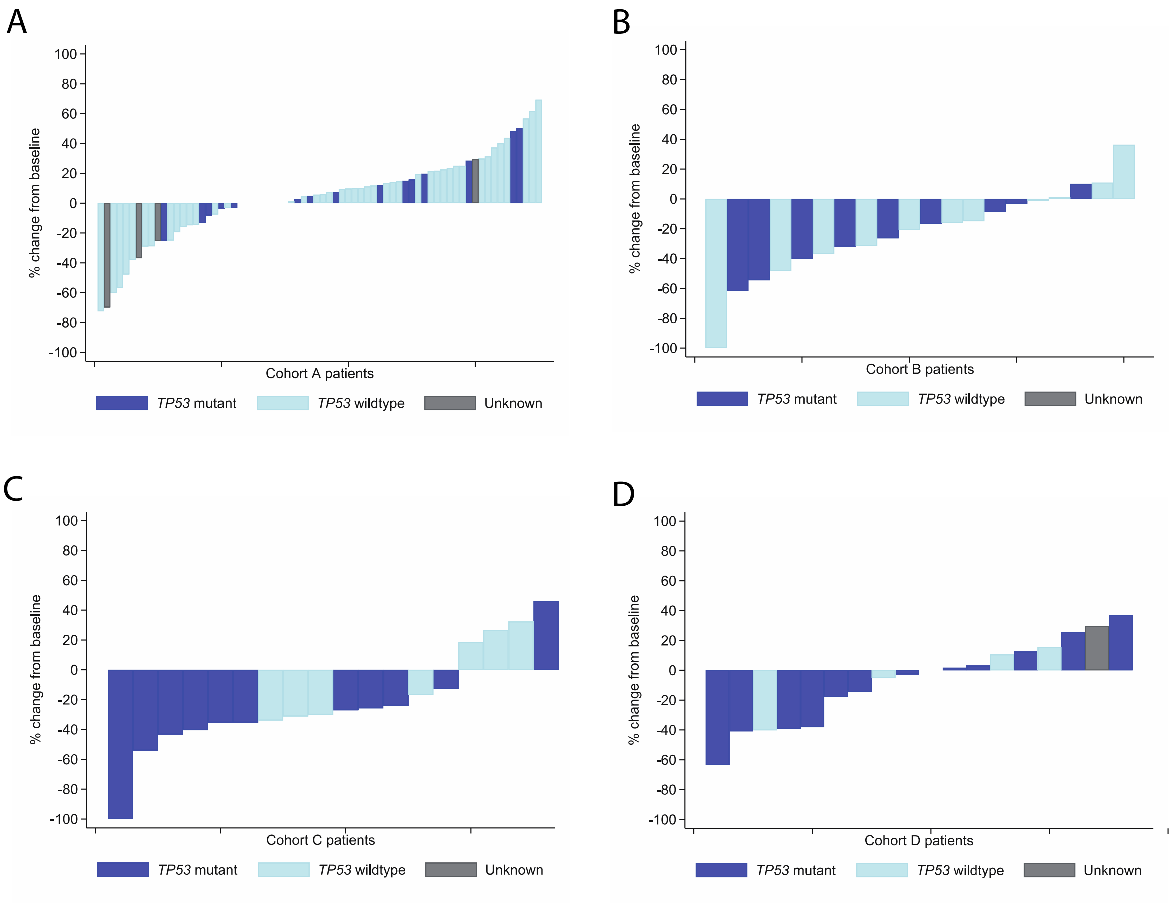

Figure S13 Tissue mutation status and efficacy of extended dose fulvestrant in Cohort A.

Waterfall plot of maximum change in tumour size in Cohort A, split by *ESR1* tissue positive and *ESR1* tissue negative. Response rate tissue positive 12.5% (3/24, 2.7-32.4) and tissue negative 4.5% (1/22, 1.2-22.8, p=0.61).

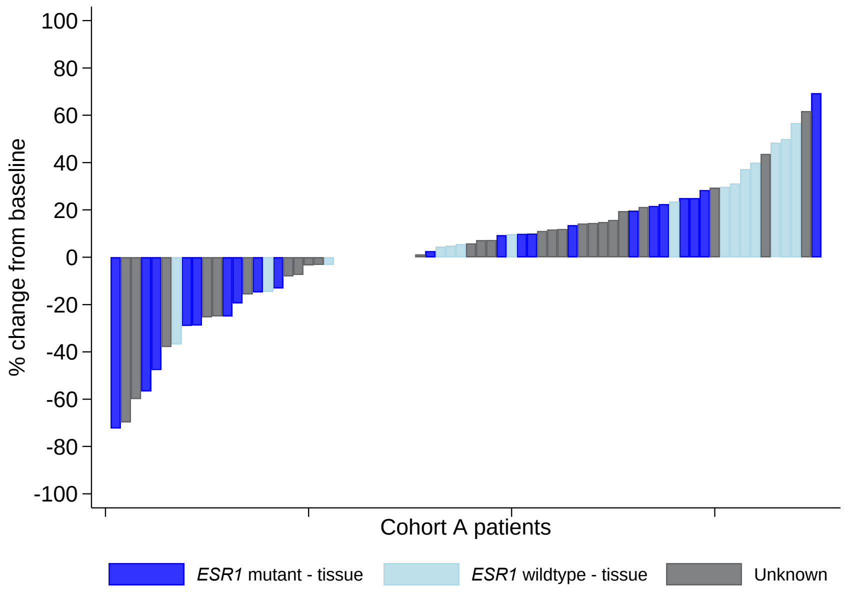

Supplementary References

1. O'Leary B, Hrebien S, Beaney M, et al. Comparison of BEAMing and Droplet Digital PCR for Circulating Tumor DNA Analysis. Clin Chem 2019;65:1405-13.
2. Lanman RB, Mortimer SA, Zill OA, et al. Analytical and Clinical Validation of a Digital Sequencing Panel for Quantitative, Highly Accurate Evaluation of Cell-Free Circulating Tumor DNA. PLoS One 2015;10:e0140712.
3. Merker JD, Oxnard GR, Compton C, et al. Circulating Tumor DNA Analysis in Patients With Cancer: American Society of Clinical Oncology and College of American Pathologists Joint Review. J Clin Oncol 2018;36:1631-41.
4. Zill OA, Banks KC, Fairclough SR, et al. The Landscape of Actionable Genomic Alterations in Cell-Free Circulating Tumor DNA from 21,807 Advanced Cancer Patients. Clin Cancer Res 2018;24:3528-38.
5. O'Leary B, Cutts RJ, Liu Y, et al. The Genetic Landscape and Clonal Evolution of Breast Cancer Resistance to Palbociclib plus Fulvestrant in the PALOMA-3 Trial. Cancer Discov 2018;8:1390-403.
6. Carreira S, Romanel A, Goodall J, et al. Tumor clone dynamics in lethal prostate cancer. Sci Transl Med 2014;6:254ra125.
7. Picard. 2019. (Accessed 21/05/2019, 2019, at https://broadinstitute.github.io/picard/.)
8. de Bruin EC, McGranahan N, Mitter R, et al. Spatial and temporal diversity in genomic instability processes defines lung cancer evolution. Science 2014;346:251-6.
9. Bushnell B, Rood J, Singer E. BBMerge – Accurate paired shotgun read merging via overlap. PLOS ONE 2017;12:e0185056.
10. Newman AM, Lovejoy AF, Klass DM, et al. Integrated digital error suppression for improved detection of circulating tumor DNA. Nature biotechnology 2016;34:547-55.
11. Wang J, Wen S, Symmans WF, Pusztai L, Coombes KR. The bimodality index: a criterion for discovering and ranking bimodal signatures from cancer gene expression profiling data. Cancer Inform 2009;7:199-216.
12. Robinson JT, Thorvaldsdóttir H, Winckler W, et al. Integrative genomics viewer. Nature biotechnology 2011;29:24-6.

The UK plasma based Molecular profiling of Advanced breast cancer to inform Therapeutic Choices (plasmaMATCH) Trial:

A multiple parallel cohort, open-label, multi-centre phase IIa clinical trial aiming to provide proof of principle efficacy for designated targeted therapies in patients with advanced breast cancer where the targetable mutation is identified through ctDNA screening

PROTOCOL

This supplement contains the following item:

- Final approved plasmaMATCH protocol, version 4.0, 25 February 2019*

*Please note that plasmaMATCH protocol, version 4.0, 25 February 2019, contains a summary of all protocol amendments in the 'History of changes' section on page iii.

The UK plasma based Molecular profiling of Advanced breast cancer to inform Therapeutic CHoices (plasmaMATCH) Trial:

A multiple parallel cohort, open-label, multi-centre phase IIa clinical trial aiming to provide proof of principle efficacy for designated targeted therapies in patients with advanced breast cancer where the targetable mutation is identified through ctDNA screening

PROTOCOL

Version: 4.0

Dated: 25 February 2019

Chief Investigator:	Prof Nicholas Turner
Co-sponsors:	The Institute of Cancer Research and The Royal Marsden NHS Foundation Trust
Funders:	Cancer Research UK and Stand Up to Cancer
Coordinating Trials Unit:	The Institute of Cancer Research Clinical Trials and Statistics Unit (ICR-CTSU)

EudraCT Number:	2015-003735-36
Main REC Reference Number:	16/SC/0271
IRAS Project ID:	187103
ICR-CTSU Protocol Number:	ICR-CTSU/2015/10056
Sponsor Number:	CCR4381
ISRCTN:	ISRCTN16945804
ClinicalTrials.gov Identifier:	NCT03182634
CRUK Reference Number:	CRUK/15/010

The plasmaMATCH trial has received funding from Cancer Research UK's Clinical Research Committee (CRC)

The plasmaMATCH trial is part of the National Institute for Health Research Clinical Research Network Trial Portfolio

This protocol is a controlled document and should not be copied, distributed or reproduced without the written permission of the ICR-CTSU

ADMINISTRATION

Scientific Coordination

Chief Investigator
Prof Nicholas Turner
The Institute of Cancer Research and
The Royal Marsden NHS Foundation Trust
Tel: 020 7153 5574
nick.turner@icr.ac.uk

Coordinating Investigator & Cohort E Clinical Lead
Dr Alistair Ring
The Royal Marsden NHS Foundation Trust
Tel: 020 8661 3362
alistair.ring@rmh.nhs.uk

Trials Methodology Lead & ICR-CTSU Scientific Lead
Prof Judith Bliss
Director ICR-CTSU
Tel: 020 8722 4013
judith.bliss@icr.ac.uk

Trial Operations Lead
Claire Snowdon
Deputy Director & Director of Operations ICR-CTSU
Tel: 020 8722 4307
claire.snowdon@icr.ac.uk

Cohort A Clinical Lead
Dr Iain Macpherson
The Beatson West of Scotland Cancer Centre
Tel: 0141 301 7128
iain.macpherson@glasgow.ac.uk

Cohort B Clinical Lead
Prof Andrew Wardley
The Christie NHS Foundation Trust and Division of Cancer Sciences, University of Manchester
Tel: 0161 918 7676/7903
andrew.wardley@christie.nhs.uk

Cohort C Clinical Lead
Dr Rebecca Roylance
University College London Hospitals NHS Foundation Trust
Tel: 020 3447 9072
r.roylance@ucl.ac.uk

Cohort D Clinical Lead
Dr Richard Baird
Cambridge University Hospitals NHS Foundation Trust
Tel: 01223 769480
richard.baird@medschl.cam.ac.uk

Trial Coordination

ICR-CTSU (a UKCRC Registered clinical trials unit (CTU) and member of the NCRI Cancer CTU Group) is responsible for the central coordination of the trial on behalf of the Co-sponsors.

**ICR-CTSU Scientific Lead**<br>Judith Bliss<br>Tel: 020 8722 4013<br>judith.bliss@icr.ac.uk	**ICR-CTSU Clinical Trials Programme Manager**<br>Sarah KernaghanTel: 020 8722 4152<br>sarah.kernaghan@icr.ac.uk		
**ICR-CTSU Senior Statistician**<br>Lucy Kilburn<br>Tel: 020 8722 4080			
**plasmaMATCH Trial Manager**	**plasmaMATCH Data Managers**		
Katie Wilkinson<br>Tel: 020 8722 4754	Sue Martin<br>Tel: 020 8722 4077	Grace Elwood<br>Tel: 020 8722 4091	Sarah Fynn<br>Tel: 020 8722 4288
**Trial Team Email:** plasmamatch-icrctsu@icr.ac.uk<br>**Address:** ICR Clinical Trials & Statistics Unit (ICR-CTSU), Division of Clinical Studies, The Institute of Cancer Research, Sir Richard Doll Building, 15 Cotswold Road, Sutton, Surrey SM2 5NG			

Any questions relating to this protocol should be addressed in the first instance to the plasmaMATCH Trial Managers within ICR-CTSU.

Protocol Development Group

Nicholas Turner	Chief Investigator	The Institute of Cancer Research, London and The Royal Marsden NHS Foundation Trust
Alistair Ring	Coordinating Investigator & Cohort E Clinical Lead	The Royal Marsden NHS Foundation Trust
Judith Bliss	Director	ICR-CTSU, The Institute of Cancer Research, London
Claire Snowdon	Deputy Director & Director of Operations	ICR-CTSU, The Institute of Cancer Research, London
Iain Macpherson	Cohort A Clinical Lead	The Beatson West of Scotland Cancer Centre
Andrew Wardley	Cohort B Clinical Lead	The Christie NHS Foundation Trust and Division of Cancer Sciences, University of Manchester
Rebecca Roylance	Cohort C Clinical Lead	University College London Hospitals NHS Foundation Trust
Richard Baird	Cohort D Clinical Lead	Cambridge University Hospitals NHS Foundation Trust
Daniel Rea	Deputy Clinical Director & Medical Oncologist	University of Birmingham
David Cameron	Clinical Director & Chair of Oncology	Edinburgh Cancer Research Centre and Western General Hospital
Abeer Shaaban	Consultant Pathologist	University Hospital Birmingham NHS Foundation Trust
Bhuey Sharma	Consultant Radiologist	The Royal Marsden NHS Foundation Trust
David Gonazlez de Castro	Head of the Molecular Diagnostics Laboratory	The Institute of Cancer Research, London
Katrina Randle	Patient Representative	
Louise Jones	Professor of Breast Pathology	Barts and the London School of Medicine and Dentistry
Rowland Illing	Consultant Interventional Radiologist	University College London Hospitals NHS Foundation Trust
Charlotte Fribbens	Clinical Research Fellow	The Institute of Cancer Research, London
James Morden	Senior Statistician	ICR-CTSU, The Institute of Cancer Research, London
Laura Moretti	Clinical Trials Programme Manager	ICR-CTSU, The Institute of Cancer Research, London
Sarah Kernaghan	Trial Manager	ICR-CTSU, The Institute of Cancer Research, London

The Trial Management Group (TMG) will be constituted from members of the Protocol Development Group and will include the Chief Investigator (CI), Coordinating Investigator, ICR-CTSU Scientific Lead, Trial Operations Lead, Cohort Clinical Leads, Co-investigators and identified collaborators, key ICR-CTSU staff and a lay representative. Additionally some Principal Investigators (PIs) and key trial personnel will be invited to join the TMG as appropriate to ensure representation from a range of sites and professional groups. A copy of the current membership of the TMG can be obtained from the plasmaMATCH Trial Manager at ICR-CTSU.

Protocol authorised by:

Name & Role	Signature	Date
Prof Nicholas Turner (Chief Investigator)	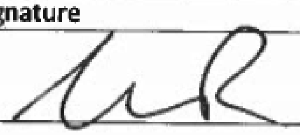	26 Feb 2019

This protocol describes the plasmaMATCH trial and provides information about procedures for entering patients into this trial. The protocol should not be used as a guide for the treatment of patients outside of this trial. Every care was taken in the preparation of this protocol, but corrections or amendments may be necessary. Protocol amendments will be circulated to participating sites as they occur; sites entering patients for the first time are advised to contact ICR-CTSU to confirm they have the most recent version.

HISTORY OF CHANGES

<table>
<tr><th>PROTOCOL VERSION AND DATE</th><th>SUMMARY OF CHANGES</th></tr>
<tr><td>Version 1.2,
18 August 2016</td><td>Not applicable – original approved version</td></tr>
<tr><td>Version 2.0,
07 November 2017</td><td>Eligibility updates
Eligibility criteria updated to limit the number of prior lines of chemotherapy in the advanced setting to <u>a maximum of two prior lines of cytotoxic chemotherapy or chemotherapy antibody-drug conjugate</u>
At ctDNA screening patients must have demonstrated progression of disease by radiological assessment or suspicion of disease progression by clinical assessment <u>following most recent line of therapy</u>
At treatment cohort entry patients must have radiological evidence of disease progression <u>following most recent line of therapy</u>
Additional minor clarifications to the eligibility criteria
Other updates
Clarification of primary objective: To assess the safety and activity profile of targeted therapies (initially including neratinib, AZD5363 and fulvestrant) in <u>patient subgroups</u> identified by ctDNA screening
Volume of blood collected for the ctDNA screening sample increased to <u>40ml</u> to enable next-generation sequencing to be conducted at an external laboratory in the US
SAE reporting section updated to stipulate that any SAE that occurs <u>after the patient has provided written informed consent for entry into a treatment cohort</u> and up to 30 days (or 60 days for Cohort A) following the last dose of trial treatment must be reported
Additional minor clarifications throughout</td></tr>
<tr><td>Version 3.0,
01 May 2018</td><td>Addition of Cohort E
Patients with triple negative breast cancer and an absence of actionable mutations identified in ctDNA screening or actionable mutations identified but not otherwise eligible for Cohorts A-D will have the opportunity to enter Cohort E to receive treatment with olaparib plus AZD6738
Primary objective updated: To assess the safety and activity profile of targeted therapies (initially including neratinib, AZD5363, fulvestrant, <u>olaparib and AZD6738</u>) in patient subgroups identified by ctDNA screening
Extension of sample size for ctDNA screening and Cohort A
Sample size for Cohort A extended to <u>78 patients</u> in order to account for presence of non-clonal and clonal ESR1 mutations in patient population
Sample size for ctDNA screening extended to <u>~1150 patients</u> in order to accommodate extended recruitment into Cohort A and addition of Cohort E
Other updates
SAE reporting section updated to stipulate that any SAEs that occur more than 30 days after the last dose of trial treatment that, in the opinion of the PI, are related to the trial treatment should be reported to ICR-CTSU if the PI becomes aware of them
Update to Cohort D inclusion criteria to clarify that patients with an AKT1 mutation identified in tumour sequencing conducted outside of plasmaMATCH with ER positive or negative breast cancer are eligible
Additional minor clarifications throughout</td></tr>
<tr><td>Version 3.1,
28 June 2018</td><td>Administrative updates
Update to trial contacts and correction of typographical error in Cohort E inclusion criteria</td></tr>
</table>

Version 4.0<br>25 February 2019	**Eligibility update**<br>• Eligibility associated with liver function updated for all cohorts confirming that patients with known Gilbert's syndrome and conjugated bilirubin within normal range are eligible for inclusion<br>• Removal of Inclusion criterion 18 for Cohort E (included in error in previous versions)<br><br>**Change in AZD5363 dose modifications Cohort C**<br>• Second dose reduction changed from 240mg to 200mg BID<br><br>**Capping of the number of patients with known BRCA1/2 germline mutations in Cohort E**<br>• Confirmation that a maximum of 15 patients with known BRCA1/2 germline mutations at the time of entry into Cohort E will be permitted<br><br>**Other updates**<br>• Multiple clarifications relating to the closure of treatment cohorts.<br>• Clarity that sample size for each Cohort (A-D) relates to the number of *evaluable* patients required<br>• Removal of section relating to ctDNA Advisory Group<br>• Confirmation that treatment should be discontinued in patients with unacceptable toxicity in Cohort B (neratinib) and Cohort C (AZD5363).<br>• To correct inconsistency between schedule of assessments and sections relating to on-treatment assessments regarding timing of pregnancy test during treatment for patients in Cohort E. Additional text added for clarity.<br>• Update to trial contacts<br>• Additional minor clarifications throughout

CONTENTS

1. INTRODUCTION 1
1.1. Background 1
1.2. Description of Population 2
1.3. Trial Rationale 2

2. TRIAL OBJECTIVES 4
2.1. Primary Objective 4
2.2. Secondary Objectives 4

3. TRIAL DESIGN 4
3.1. ctDNA Screening Component 5
3.2. Therapeutic Component 5

4. TRIAL ENDPOINTS 7
4.1. Primary Endpoint 7
4.2. Secondary Endpoints 7
4.3. Exploratory Endpoints 8

5. SELECTION OF PATIENTS FOR REGISTRATION 8
5.1. Number of Patients 8
5.2. Source of Patients 8
5.3. Eligibility Criteria for Registration 9
5.4. Obtaining Informed Consent for Registration 11
5.5. Pre-Registration Procedure 11

6. REGISTRATION PROCEDURE 11
7. ctDNA SCREENING PROCEDURE FOLLOWING REGISTRATION 12
7.1. Blood Sample Collection 12
7.2. ctDNA Analysis 12
7.3. ctDNA Screening Results 13

8. SELECTION OF PATIENTS FOR ENTRY INTO TREATMENT COHORTS 13
8.1. Obtaining Informed Consent for Entry into the Treatment Cohorts – Treatment Sites Only 13
8.2. Registered Patients Who Do Not Consent to Treatment Cohort Entry 14

9. TREATMENT COHORT ENTRY PROCEDURE – TREATMENT SITES ONLY 14
10. PATIENTS WITH TUMOUR SEQUENCING RESULTS CONDUCTED OUTSIDE OF plasmaMATCH 16
10.1. Requirements for Entry into Cohort D 16
10.2. Obtaining Informed Consent 16
10.3. Confirmation of Tumour Sequencing Results 16
10.4. Entry into Cohort D 16

11. plasmaMATCH SCREENING LOG 17
12. PHARMACOVIGILANCE 18
12.1. Definitions 18
12.2. Reporting of Adverse Events to ICR-CTSU 19
12.3. Reporting of Serious Adverse Events to ICR-CTSU 20
12.4. Review of Serious Adverse Events 20
12.5. Expedited Reporting of SUSARs 21
12.6. Follow up of Serious Adverse Events 21
12.7. Annual Reporting of Serious Adverse Reactions 21
12.8. Reporting Pregnancies 21

13. STATISTICAL CONSIDERATIONS 23
13.1. Treatment Allocation 23
13.2. Statistical Design and Sample Size Justification 23
13.3. Endpoint Definitions 24
13.4. Statistical Analysis Plan 27
13.5. Interim Analyses and Stopping Rules 27

14. TRIAL MANAGEMENT ... 27
14.1. Trial Management Group ... 27
14.2. Trial Steering Committee ... 27
14.3. Independent Data Monitoring Committee ... 28
14.4. Safety Review Committee ... 28
14.5. ctDNA Advisory Group ... **Error! Bookmark not defined.**

15. RESEARCH GOVERNANCE ... 28
15.1. Co-sponsor Responsibilities ... 28
15.2. Participating Site Responsibilities ... 28
15.3. AstraZeneca Responsibilities ... 28
15.4. Puma Biotechnology Responsibilities ... 28

16. TRIAL ADMINISTRATION AND LOGISTICS ... 29
16.1. plasmaMATCH Participating Sites ... 29
16.2. Site Activation ... 30
16.3. Data Acquisition ... 30
16.4. Central Data Monitoring ... 30
16.5. On-Site Monitoring ... 30
16.6. Completion of the Trial and Definition of Trial End Date ... 31
16.7. Archiving ... 31

17. PATIENT PROTECTION AND ETHICAL CONSIDERATIONS ... 31
17.1. Trial Approvals ... 31
17.2. Trial Conduct ... 31
17.3. Informed Consent ... 31
17.4. Patient Confidentiality ... 31
17.5. Data Protection ... 32
17.6. Insurance and Liability ... 32

18. FINANCIAL MATTERS ... 32
19. PUBLICATION POLICY ... 32
A1. COHORT A BACKGROUND AND RATIONALE ... 34
A2. KNOWN RISKS AND BENEFITS OF FULVESTRANT ... 34
A3. COHORT A SPECIFIC ELIGIBILITY CRITERIA ... 35
A3.1. Inclusion Criteria ... 35
A3.2. Exclusion Criteria ... 36

A4. COHORT A TRIAL ASSESSMENTS ... 38
A4.1. Cohort A Screening Assessments ... 38
A4.2. Cohort A Baseline Assessments Pre-Treatment Cycle 1 Day 1 ... 38
A4.3. Cohort A On-Treatment Assessments ... 39
A4.4. Cohort A End of Treatment Assessments ... 40
A4.5. Cohort A Post-Treatment Follow up ... 41
A4.6. Cohort A Discontinuation from Treatment ... 41
A4.7. Cohort A Discontinuation from Follow up ... 41
A4.8. Cohort A Schedule of Assessments ... 43

A5. COHORT A TRIAL TREATMENT ... 46
A5.1. Cohort A Dose and Schedule ... 46
A5.2. Cohort A Prescription and Dispensing ... 46
A5.3. Cohort A Patient Cards ... 46
A5.4. Cohort A Duration of Treatment ... 46
A5.5. Cohort A Permitted Concomitant Therapy ... 47
A5.6. Cohort A Non-permissible Medications/Therapies ... 47
A5.7. Cohort A Dose Modifications ... 47
A5.8. Cohort A Dose Interruptions ... 48
A5.9. Cohort A Missed Doses ... 48
A5.10. Cohort A Overdoses ... 48
A5.11. Cohort A Discontinuation and Subsequent Therapy ... 48

A5.12. Cohort A Supply and Distribution of Fulvestrant 48
A5.13. Cohort A Formulation, Packaging, Storage Conditions and Labelling 48
A5.14. Cohort A Pharmacy Responsibilities and Drug Accountability 49

A6. COHORT A ASSESSMENT OF TOLERABILITY OF FULVESTRANT 49
B1. COHORT B BACKGROUND AND RATIONALE 52
B2. KNOWN RISKS AND BENEFITS OF NERATINIB 53
B3. COHORT B SPECIFIC ELIGIBILITY CRITERIA 53
B3.1. Inclusion Criteria 53
B3.2. Exclusion Criteria 55

B4. COHORT B TRIAL ASSESSMENTS 56
B4.1. Cohort B Screening Assessments 56
B4.2. Cohort B Baseline Assessments Pre-Treatment Cycle 1 Day 1 56
B4.3. Cohort B On-Treatment Assessments 57
B4.4. Cohort B End of Treatment Assessments 58
B4.5. Cohort B Post-Treatment Follow up 59
B4.6. Cohort B Discontinuation from Treatment 59
B4.7. Cohort B Discontinuation from Follow up 60
B4.8. Cohort B Schedule of Assessments 61

B5. COHORT B TRIAL TREATMENT 64
B5.1. Cohort B Dose and Schedule 64
B5.2. Cohort B Prescription and Dispensing 64
B5.3. Cohort B Patient Cards and Treatment Diary Cards 65
B5.4. Cohort B Duration of Treatment 65
B5.5. Cohort B Required Concomitant Treatment 65
B5.6. Cohort B Permitted Concomitant Therapy 66
B5.7. Cohort B Non-permissible Medications/Therapies 67
B5.8. Cohort B Potential for Drug–Drug Interactions 67
B5.9. Cohort B Dose Modifications 67
B5.10. Cohort B Dose Interruptions 71
B5.11. Cohort B Missed Doses 71
B5.12. Cohort B Overdoses 71
B5.13. Cohort B Discontinuation and Subsequent Therapy 72
B5.14. Cohort B Compliance 72
B5.15. Cohort B Supply and Distribution of Neratinib and Fulvestrant 72
B5.16. Cohort B Formulation, Packaging, Storage Conditions and Labelling 72
B5.17. Cohort B Pharmacy Responsibilities and Drug Accountability 73

C1. COHORT C BACKGROUND AND RATIONALE 75
C2. KNOWN RISKS AND BENEFITS OF AZD5363 AND FULVESTRANT 75
C3. COHORT C SPECIFIC ELIGIBILITY CRITERIA 75
C3.1. Inclusion Criteria 75
C3.2. Exclusion Criteria 77

C4. COHORT C TRIAL ASSESSMENTS 79
C4.1. Cohort C Screening Assessments 79
C4.2. Cohort C Baseline Assessments Pre-Treatment Cycle 1 Day 1 79
C4.3. Cohort C On-Treatment Assessments 80
C4.4. Cohort C End of Treatment Assessments 81
C4.5. Cohort C Post-treatment Follow up 82
C4.6. Cohort C Discontinuation from Treatment 82
C4.7. Cohort C Discontinuation from Follow up 83
C4.8. Cohort C Schedule of Assessments 84

C5. COHORT C TRIAL TREATMENT 87
C5.1. Cohort C Dose and Schedule 87
C5.2. Cohort C Prescription and Dispensing 87
C5.3. Cohort C Patient Cards and Treatment Diary Cards 88

C5.4. Cohort C Duration of Treatment 88
C5.5. Cohort C Permitted Concomitant Therapy 88
C5.6. Cohort C Non-permissible Medications/Therapies 88
C5.7. Cohort C Dose Modifications 89
C5.8. Cohort C Dose Interruptions 92
C5.9. Cohort C Missed Doses 92
C5.10. Cohort C Overdoses 92
C5.11. Cohort C Discontinuation and Subsequent Therapy 92
C5.12. Cohort C Compliance 92
C5.13. Cohort C Supply and Distribution of AZD5363 and Fulvestrant 93
C5.14. Cohort C Formulation, Packaging, Storage Conditions and Labelling 93
C5.15. Cohort C Pharmacy Responsibilities and Drug Accountability 93

D1. COHORT D BACKGROUND AND RATIONALE 95
D2. KNOWN RISKS AND BENEFITS OF AZD5363 95
D3. COHORT D SPECIFIC ELIGIBILITY CRITERIA 96
D3.1. Inclusion Criteria 96
D3.2. Exclusion Criteria 97

D4. COHORT D TRIAL ASSESSMENTS 99
D4.1. Cohort D Screening Assessments 99
D4.2. Cohort D Baseline Assessments Pre-Treatment Cycle 1 Day 1 99
D4.3. Cohort D On-Treatment Assessments 100
D4.4. Cohort D End of Treatment Assessments 101
D4.5. Cohort D Post-Treatment Follow up 102
D4.6. Cohort D Discontinuation from Treatment 102
D4.7. Cohort D Discontinuation from Follow up 103
D4.8. Cohort D Schedule of Assessments 104

D5. COHORT D TRIAL TREATMENT 107
D5.1. Cohort D Dose and Schedule 107
D5.2. Cohort D Prescription and Dispensing 107
D5.3. Cohort D Patient Cards and Treatment Diary Cards 107
D5.4. Cohort D Duration of Treatment 108
D5.5. Cohort D Permitted Concomitant Therapy 108
D5.6. Cohort D Non-permissible Medications/Therapies 108
D5.7. Cohort D Dose Modifications 108
D5.8. Cohort D Dose Interruptions 110
D5.9. Cohort D Missed Doses 111
D5.10. Cohort D Overdoses 111
D5.11. Cohort D Discontinuation and Subsequent Therapy 111
D5.12. Cohort D Compliance 111
D5.13. Cohort D Supply and Distribution of AZD5363 111
D5.14. Cohort D Formulation, Packaging, Storage Conditions and Labelling 111
D5.15. Cohort D Pharmacy Responsibilities and Drug Accountability 112

E1. COHORT E BACKGROUND AND RATIONALE 114
E2. COHORT E DESIGN 115
E3. KNOWN RISKS AND BENEFITS OF OLAPARIB AND AZD6738 116
E4. COHORT E SPECIFIC ELIGIBILITY CRITERIA 118
E4.1. Inclusion Criteria 118
E4.2. Exclusion Criteria 120

E5. COHORT E TRIAL ASSESSMENTS 122
E5.1. Cohort E Screening Assessments 122
E5.2. Cohort E Baseline Assessments Pre-Treatment Cycle 1 Day 1 122
E5.3. Cohort E On-Treatment Assessments 123
E5.4. Cohort E End of Treatment Assessments 125
E5.5. Cohort E Post-Treatment Follow up 125

E5.6. Cohort E Discontinuation from Treatment 125
E5.7. Cohort E Discontinuation from Follow up 126
E5.8. Cohort E Schedule of Assessments 127

E6. COHORT E TRIAL TREATMENT 130
E6.1. Cohort E Dose and Schedule 130
E6.2. Cohort E Prescription and Dispensing 130
E6.3. Cohort E Patient Cards and Treatment Diary Cards 131
E6.4. Cohort E Duration of Treatment 131
E6.5. Cohort E Permitted Concomitant Therapy 131
E6.6. Cohort E Non-permissible Medications/Therapies 131
E6.7. Cohort E Additional Cautions 133
E6.8. Cohort E Dose Modifications 133
E6.9. Cohort E Dose Interruptions 139
E6.10. Cohort E Missed Doses 139
E6.11. Cohort E Overdoses 139
E6.12. Cohort E Discontinuation and Subsequent Therapy 140
E6.13. Cohort E Compliance 140
E6.14. Cohort E Supply and Distribution of Olaparib and AZD6738 140
E6.15. Cohort E Formulation, Packaging, Storage Conditions and Labelling 140
E6.16. Cohort E Pharmacy Responsibilities and Drug Accountability 140

REFERENCES 142
APPENDIX 1: GLOSSARY 146
APPENDIX 2: SAMPLE COLLECTION AND TRANSLATIONAL RESEARCH 148
APPENDIX 3: RESPONSE EVALUATION CRITERIA IN SOLID TUMOURS (RECIST) VERSION 1.1 151
APPENDIX 4: ECOG PERFORMANCE STATUS 155
APPENDIX 5: CREATININE CLEARANCE CALCULATION 156
APPENDIX 6: COHORT B – POTENTIAL FOR DRUG–DRUG INTERACTIONS 157
APPENDIX 7: COHORTS C AND D – AZD5363 CONCOMITANT TREATMENT CAUTIONS AND RESTRICTIONS .. 161
APPENDIX 8: COHORT E – OLAPARIB CONCOMITANT TREATMENT CAUTIONS AND RESTRICTIONS 166
APPENDIX 9: COHORT E – AZD6738 CONCOMITANT TREATMENT CAUTIONS AND RESTRICTIONS 168

LIST OF FIGURES AND TABLES

FIGURES

Figure 1. plasmaMATCH Trial Schema 16
Figure 2. Schematic of plasmaMATCH umbrella clinical trial design 3
Figure 3. Flow diagram for SAE reporting, and action following report 22
Figure E1. Olaparib + AZD6738 combination efficacy in a triple negative breast cancer patient derived xenograft (52) 115
Figure E2. Cohort E two-stage design 116

TABLES

Table 1. SAE reporting – definitions of causality 19
Table B1. Cohort B diarrhoea prophylaxis – loperamide dosing 65
Table B2. Cohort B loperamide dose adjustment guidelines 66
Table B3. Cohort B neratinib dose reduction guidelines 68
Table B4. Cohort B diarrhoea management guidelines 69
Table B5. Cohort B pneumonitis management guidelines 70
Table B6. Cohort B hepatic toxicity management guidelines 70
Table B7. Cohort B LVEF toxicity management guidelines 71
Table C1. Cohort C treatment schedule 87
Table C2. Cohort C AZD5363 dose reduction guidelines 89
Table C3. Cohort C hyperglycaemia management guidelines 90

Table C4. Cohort C diarrhoea management guidelines ... 91
Table C5. Cohort C rash management guidelines ... 91
Table D1. Cohort D AZD5363 dose reduction guidelines ... 108
Table D2. Cohort D hyperglycaemia management guidelines ... 109
Table D3. Cohort D diarrhoea management guidelines ... 110
Table D4. Cohort D rash management guidelines ... 110
Table E1. Cohort E olaparib and AZD6738 dose reductions guidelines ... 134
Table E2. Cohort E haematological toxicity management guidelines ... 134
Table E3. Cohort E non-haematological toxicity management guidelines ... 136
Table E4. Cohort E hepatic toxicity management guidelines ... 139
Appendix 3, Table 1. RECIST v1.1 evaluation of target lesions ... 152
Appendix 3, Table 2. RECIST v1.1 evaluation of non-target lesions ... 153
Appendix 3, Table 3. RECIST v1.1 evaluation of overall response ... 153
Appendix 4, Table 1. ECOG performance status ... 155
Appendix 6, Table 1. Cohort B – Drugs associated with risk of QT/QTc prolongation leading to Torsade de pointes ... 157
Appendix 6, Table 2. Cohort B – Substrates and inhibitors of P-glycoprotein (P-gp) ... 159
Appendix 6, Table 3. Cohort B – Inhibitors and inducers of the cytochrome P450 isoenzymes ... 160
Appendix 7, Table 1. Cohorts C and D – Strong CYP3A4 inhibitors that may increase exposure to AZD5363 more than 5-fold ... 161
Appendix 7, Table 2. Cohorts C and D – Potent Inducers of CYP3A4 that may reduce exposure to AZD5363 by more than 5-fold ... 161
Appendix 7, Table 3. Cohorts C and D – Moderate Inhibitors of CYP3A4 that may increase exposure to AZD5363 ... 162
Appendix 7, Table 4. Cohorts C and D – Exposure, pharmacological action and toxicity that may be increased by inhibition of CYP3A4 by AZD5363 ... 162
Appendix 7, Table 5. Cohorts C and D – Exposure, pharmacological action and toxicity that may be increased by inhibition of CYP3A4 by AZD5363 ... 163
Appendix 7, Table 6. Cohorts C and D – Exposure, pharmacological action and toxicity that may be increased by inhibition of CYP2D6 by AZD5363 ... 163
Appendix 7, Table 7. Cohorts C and D – Exposure, pharmacological action and toxicity that may be increased by inhibition of CYP2D6 by AZD5363 ... 164
Appendix 7, Table 8. Cohorts C and D – Exposure, pharmacological action and toxicity that may be increased by inhibition of CYP3A4 and CYP2D6 by AZD5363 ... 164
Appendix 7, Table 9. Cohorts C and D – Exposure, pharmacological action and toxicity that may be increased by inhibition of CYP2B6, CYP2C9 and CYP2C19 by AZD5363 ... 164
Appendix 9, Table 1. Drugs known to be inhibitors and inducers of CYP3A ... 168
Appendix 9, Table 2. Drugs known to be inhibitors or inducers of P-gp ... 169
Appendix 9, Table 3. Drugs known to be inhibitors or inducers of BCRP ... 169
Appendix 9, Table 4. Drugs known to be metabolised by CYP3A4 and have a narrow therapeutic index ... 170
Appendix 9, Table 5. Drugs known to be metabolised by CYP2B6 and have a narrow therapeutic index ... 170
Drugs known to be metabolised by CYP2B6 and have a narrow therapeutic index ... 170
Appendix 9, Table 6. Drugs known to be substrates of OATP1B1 ... 170
Appendix 9, Table 7. Drugs known to be substrates of BCRP ... 171

plasmaMATCH TRIAL PROTOCOL STRUCTURE

plasmaMATCH is a multiple parallel cohort, open-label, multi-centre phase IIa umbrella clinical trial in patients with advanced breast cancer.

Eligible patients who provide consent for plasmaMATCH screening will be registered for circulating tumour DNA (ctDNA) screening and a sample of their blood will be sent to the central laboratory for processing and mutation analysis.

Eligible patients with a targetable mutation identified at ctDNA screening will be invited to enter Cohorts A to D within the therapeutic component of the trial. Eligible patients with a targetable mutation identified by tumour sequencing conducted outside of plasmaMATCH may enter Cohort D. Patients with triple negative breast cancer (TNBC) on their most recent tumour biopsy who do not have a targetable mutation identified by ctDNA screening or tumour sequencing that would allow entry into Cohorts A to D, or who have an actionable mutation identified but are not otherwise eligible for Cohorts A to D may be eligible to enter Cohort E.

Patients who do not have a targetable mutation identified at ctDNA screening and are not eligible for Cohort E will be treated as per standard of care outside the context of this trial.

For patients with more than one mutation identified at ctDNA screening a hierarchy of the treatment cohorts has been defined as described in Section 3.2.3.

Consenting patients will enter the relevant treatment cohort and commence trial treatment with the applicable targeted therapy.

This protocol is separated into clearly labelled sections:

SECTIONS 1 – 4 provide background information on the plasmaMATCH trial, including the overall trial design, aims and objectives
SECTIONS 5 – 7 describe the procedure for patient registration and ctDNA screening
SECTIONS 8 – 11 describe the procedure for patient entry into a treatment cohort
SECTION 12 provides instructions on safety reporting procedures
SECTIONS 13 – 19 provide information on the statistical considerations, trial management and research governance

Once the patient has entered into a treatment cohort please refer to the relevant cohort section for the schedule of assessments, information on trial treatment and dose modifications:

SECTION A – **Cohort A:** *ESR1* mutation treated with extended-dose fulvestrant
SECTION B – **Cohort B:** *HER2* mutation treated with neratinib plus fulvestrant in estrogen receptor (ER) positive breast cancer or neratinib only in ER negative breast cancer
SECTION C – **Cohort C:** *AKT1* mutation treated with AZD5363 and fulvestrant in ER positive breast cancer
SECTION D – **Cohort D:** AKT activation basket mutations treated with AZD5363
SECTION E – **Cohort E**: Triple negative breast cancer treated with olaparib plus AZD6738

plasmaMATCH TRIAL SUMMARY

**PROTOCOL TITLE**	**The UK plasma based Molecular profiling of Advanced breast cancer to inform Therapeutic CHoices (plasmaMATCH) Trial:** A multiple parallel cohort, open-label, multi-centre phase IIa clinical trial aiming to provide proof of principle efficacy for designated targeted therapies in patients with advanced breast cancer where the targetable mutation is identified through ctDNA screening
**TARGET DISEASE**	Advanced breast cancer
**PRIMARY OBJECTIVE**	To assess the safety and activity profile of targeted therapies (initially including neratinib, AZD5363, fulvestrant, olaparib and AZD6738) in patient subgroups identified by ctDNA screening.
**SECONDARY OBJECTIVES**	**ctDNA screening component:**<br>1. To determine the frequency of targetable genetic mutations in a large population of patients with advanced breast cancer.<br>2. To determine the proportion of patients with targetable mutations identified by ctDNA screening who enter the therapeutic component.<br>3. To assess whether ctDNA is a feasible multi-centre screening tool for detecting aberrations in advanced breast cancer.<br>4. To bank plasma samples to allow future development of ctDNA assays.<br><br>**Therapeutic component:**<br>1. To determine whether ctDNA screening can select populations of patients who will be sensitive to targeted therapies.<br>2. To determine whether serial ctDNA assessment on treatment can be used to monitor response and the development of resistance to targeted therapies.<br>3. To assess the level of agreement between ctDNA screening and recurrent disease biopsies.<br>4. To investigate whether patients with apparently clonal mutations in the tumour derive greater benefit from targeted therapy than patients with subclonal mutations.<br>5. To determine the efficiency of the dynamic trial platform design in providing proof of principle efficacy for designated targeted therapies.
**TRIAL DESIGN**	Multiple parallel cohort, open-label, multi-centre phase IIa umbrella clinical trial, with ctDNA screening.
**TRIAL POPULATION**	Patients with metastatic or recurrent locally advanced breast cancer with prior treatment in the advanced setting.

**RECRUITMENT TARGET**	**ctDNA screening component:**<br>Approximately 1150 patients<br><br>**Therapeutic component:**<br>• Cohort A – 78 patients<br>• Cohort B – 16 patients<br>• Cohort C – 16 patients<br>• Cohort D – 16 patients<br>• Cohort E – max. 69 patients
**ctDNA SCREENING COMPONENT**	Patients meeting the eligibility criteria for registration will be asked to provide a blood sample for central ctDNA screening.<br><br>Patients must have disease progression on radiological grounds or suspicion of disease progression on clinical grounds following the most recent line of therapy to provide consent for ctDNA screening.
**THERAPEUTIC COMPONENT**	If a targetable mutation is identified at ctDNA screening the patient will be invited to enter the relevant treatment cohort and consenting patients will commence treatment with the applicable agent(s) as outlined below:<br><br>**Cohort A:** *ESR1* mutation identified in ctDNA screening treated with extended-dose fulvestrant.<br>• 500mg fulvestrant to be administered intramuscularly (IM) on Cycle 1 Days 1, 8 and 15 and Cycle 2 onwards Days 1 and 15.<br><br>**Cohort B:** *HER2* mutation identified in ctDNA screening in patients with ER positive breast cancer treated with neratinib plus fulvestrant, or in patients with ER negative breast cancer treated with neratinib only.<br>• 240mg neratinib to be administered once daily on a continuous schedule starting on Cycle 1 Day 1.<br>• And in ER positive breast cancer, 500mg fulvestrant to be administered IM on Cycle 1 Days 1 and 15 and Cycle 2 onwards Day 1.<br><br>**Cohort C:** *AKT1* mutation identified in ctDNA screening in patients with ER positive breast cancer treated with AZD5363 and fulvestrant.<br>• 400mg AZD5363 to be administered twice daily on a 7 day schedule of 4 days on treatment followed by 3 days off treatment.<br>• And 500mg fulvestrant IM Cycle 1 Days 1 and 15 and Cycle 2 onwards Day 1.<br><br>**Cohort D:** AKT activation basket with mutations of *AKT1* identified in plasmaMATCH ctDNA screening in patients with ER negative breast cancer, mutations of *AKT1* identified in prior tumour sequencing conducted outside of plasmaMATCH in patients with ER positive or negative breast cancer, or *AKT2/3* E17K*, PIK3R1* or *PTEN* mutations or homozygous deletion of *PTEN* in both ER positive and ER negative breast cancer identified in ctDNA screening or in prior tumour sequencing conducted outside of plasmaMATCH, treated with AZD5363.<br>• 480mg AZD5363 to be administered twice daily on a 7 day schedule of 4 days on treatment followed by 3 days off treatment.

Patients with triple negative breast cancer (TNBC) on their most recent tumour biopsy who do not have a targetable mutation identified by ctDNA screening or tumour sequencing that would allow entry into Cohorts A to D, or who have an actionable mutation identified but are not otherwise eligible for Cohorts A to D will be invited to enter Cohort E and consenting patients will commence treatment as outlined below:

Cohort E: TNBC on most recent tumour biopsy. Patients treated with olaparib plus AZD6738.

- 160mg AZD6738 to be administered once daily on Days 1–7 of each cycle.
- And 300mg olaparib to be administered twice daily on a continuous schedule starting on Cycle 1 Day 1.

For each cohort a cycle consists of 28 days.

Treatment will continue until disease progression according to RECIST v1.1. Patients will be assessed by CT scan every 8 weeks with assessment of response by RECIST v1.1. After 32 weeks patients will be assessed by CT scan every 12 weeks.

PRIMARY ENDPOINT

The primary endpoint will be determined for each cohort separately.

The primary endpoint for Cohorts A to E is confirmed objective response rate as defined by RECIST v1.1 for each cohort separately.

SECONDARY ENDPOINTS

- Clinical benefit rate and progression free survival (PFS) for each cohort separately.
- Safety and tolerability of therapies.
- Duration of response for each cohort.
- Frequency of mutations identified in ctDNA screening and the proportion of patients with a targetable mutation who enter the therapeutic component.
- Agreement between ctDNA mutation status and tissue mutation status for patients entering the therapeutic component.
- Pharmacokinetics in Cohorts A and B.

EXPLORATORY ENDPOINTS

- Comparison of overall response rate between cancers with apparently clonal mutations and those with sub-clonal mutations.
- Heterogeneity in change in tumour size between lesions in ctDNA-driven cohorts in cancers with apparently clonal mutations and those with sub-clonal mutations.
- Association between change in ctDNA abundance on treatment with maximum change in tumour size, overall response rate and PFS in cohorts individually and combined.
- The proportion of cancers with ctDNA detected HER2 amplification that have HER2 amplification on optional subsequent disease biopsy.
- Exploratory assessment of the pharmacodynamic effect of therapies in patients consenting to optional on-treatment biopsies.
- Exploratory assessment of mechanisms of resistance to therapies in optional biopsies taken at disease progression.

- Identification of a ctDNA biomarker that predicts for response to olaparib plus AZD6738.

FOLLOW UP Patients will be followed up every 6 months until death or up to 2 years following the end of trial treatment.

Figure 1. plasmaMATCH Trial Schema

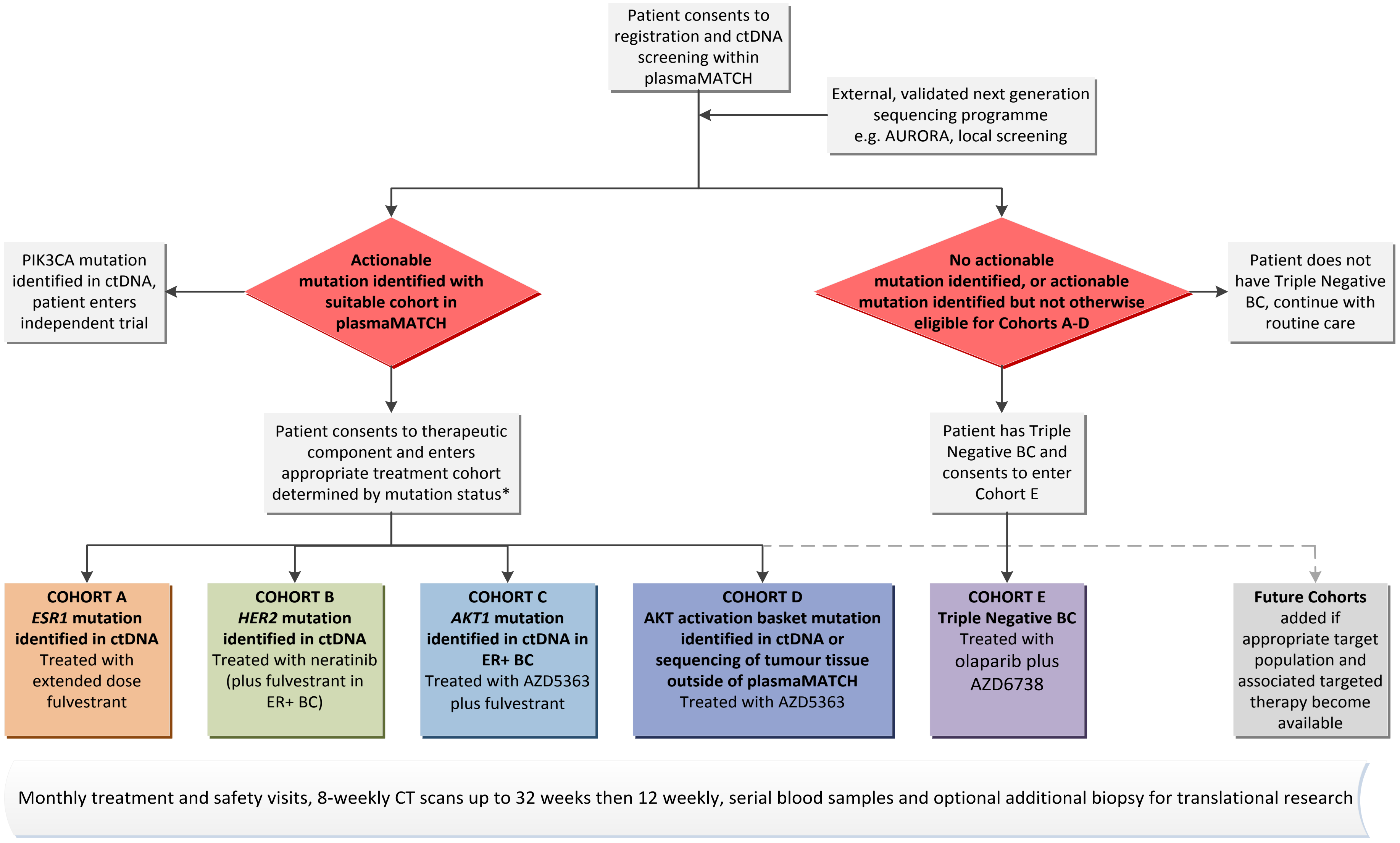

*In the case of patients with more than one actionable mutation identified, a hierarchy of mutations is outlined in Section 3 of the protocol.

1. INTRODUCTION

1.1. Background

Breast cancer is the most common cancer in UK women with ~50,000 cases diagnosed and 11,700 deaths attributed to the disease per year (1). Despite significant advances in the management of metastatic breast cancer (MBC), it remains a cancer of substantial unmet need. Treatment of MBC is non-curative, with breast cancer being the second most common cause of cancer-related death in women, and advanced stages of the disease are associated with considerable morbidity (2). In recent years, breast cancer has been recognised as a heterogeneous disease comprised of distinct molecular subtypes and large collaborative sequencing projects have defined genetic profiles for both early and MBC (3-6). Although some potentially targetable mutations occur at relatively high frequency, such as *PIK3CA* mutation and *HER2* amplification, the majority of currently targetable mutations occur at low frequency. Many of these low frequency genetic events present excellent potential therapeutic targets yet their rarity make the assessment of each in separate clinical trials impractical.

Increasing evidence suggests that genetic events may be acquired in MBC. Mutations in the estrogen receptor gene (*ESR1*) are found in ~20% of patients with recurrent estrogen receptor (ER) positive breast cancer (7), with the mutation not present in the primary cancers but acquired as a mechanism of resistance to prior endocrine therapy. Similarly ~10% of patients with *HER2* amplification may acquire activating mutations in the *HER2* gene as a mechanism of resistance to trastuzumab (8), and *HER2* amplification may be acquired in ~2-5% of MBCs with a HER2 negative primary cancer (9). At present, many of these genetic events would not be identified in routine clinical practice where recurrent breast cancer may not be re-biopsied, meaning treatment for recurrent disease is based on results from the primary tumour. Repeat biopsy can provide contemporaneous molecular assessment of recurrent disease, but is not conducted in approximately half of patients with recurrent breast cancer in routine clinical practice. This is because repeat biopsy can be technically challenging due to accessibility of the disease and confers additional discomfort and inconvenience for the patient. Establishing an alternative, non-invasive method of characterising tumour molecular profiles with circulating tumour DNA (ctDNA) would simplify screening, reduce costs and reduce risk to patients from an invasive procedure. Furthermore, repeated assessments throughout the disease course to identify mutations acquired during prior treatment, such as *ESR1* mutations, is only practically possible in routine clinical practice with the use of non-invasive methods such as analysis of ctDNA.

ctDNA is found in the plasma of over 90% of women with MBC (10, 11), and we have recently shown how tracking tumour specific mutations in plasma can predict early relapse following the treatment of primary breast cancer (12). At the time plasmaMATCH was initiated, results had indicated that assays of ctDNA have concordance as high as 100% with biopsies of metastatic disease, including *PIK3CA* mutations in patients with MBC (13, 14). Metastatic tumour biopsy is limited to samples from a single site of disease which may not be fully representative of all sites of metastasis. ctDNA assessment may circumvent the limitations of metastatic tissue biopsies with respect to heterogeneity of metastatic sites (15, 16) and potentially allow identification of genetic aberrations that are driving a tumour but are not present in the single biopsied sample (15). Screening ctDNA therefore provides a valid approach to characterising the molecular profile of metastatic tissue.

1.2. Description of Population

Patients with metastatic or recurrent locally advanced breast cancer who have received prior systemic treatment in the advanced setting will be invited to participate.

1.3. Trial Rationale

Screening for the presence of genomic abnormalities in ctDNA is more practical than metastatic tumour biopsies, is suitable for all patients (not just those with accessible disease), more economical and more acceptable to patients, lending itself to widespread screening to include a larger proportion of the eligible population. The reduction in invasive procedures with the use of ctDNA screening is key when considering screening for rare genetic events, as otherwise screening would involve a substantial number of patients undergoing biopsies to identify only a few with targetable aberrations. Finally, screening ctDNA provides a current assessment of the genetic profile of recurrent cancer, allowing the identification of genetic events selected by prior treatments. This is a particular issue in the treatment of breast cancer, where intervening adjuvant therapy may drive changes in the genetic profile of recurrent cancer. Large-scale screening with non-invasive methods, such as ctDNA screening, is the only practical strategy to identify relevant populations in large numbers and make clinical trials viable and accessible to the widest patient population.

Potential therapies which are target driven can have low activity when tested in unselected cohorts of patients, with screening to select appropriate populations for treatment becoming an increasingly important part of identifying the therapeutic potential of new agents. The EGFR-targeted tyrosine kinase inhibitor, gefitinib, was initially developed in unselected populations of patients with non-small cell lung cancer, with only limited clinical activity and non-significant phase III trials. Subsequently it became apparent that the efficacy of gefitinib was substantially higher in patients with an EGFR mutation, for example objective response rate in the non-small cell lung cancer IPASS study was 71% in EGFR mutation carriers and 1% in non-mutation carriers (17). This supports the notion that the efficacy of targeted therapies may be considerably improved by appropriate selection of populations, and that the efficacy may be significantly underestimated where the mutation is a rare event in unselected populations (as is the case for the mutations targeted in plasmaMATCH). Furthermore it is now accepted by the European Medicines Agency (EMA) that EGFR-mutation status assessed via ctDNA is a valid means by which to assess mutation status and decide which patients with non-small cell lung cancer should be selected for gefitinib therapy (18, 19).

The umbrella clinical trial design of plasmaMATCH allows biologically different and defined subgroups of patients with advanced breast cancer to be identified and triaged into distinct treatment cohorts in order to receive a targeted therapy which is hypothesised to be of benefit to that specific patient subgroup as shown in Figure 2. Whilst this is expected to be via a pairing of detectable actionable mutations with a designated therapeutic the design also allows patients to be selected, within broader molecular phenotypes, by the absence of mutations to address the unmet need in patients who otherwise do not have targeted treatments available.

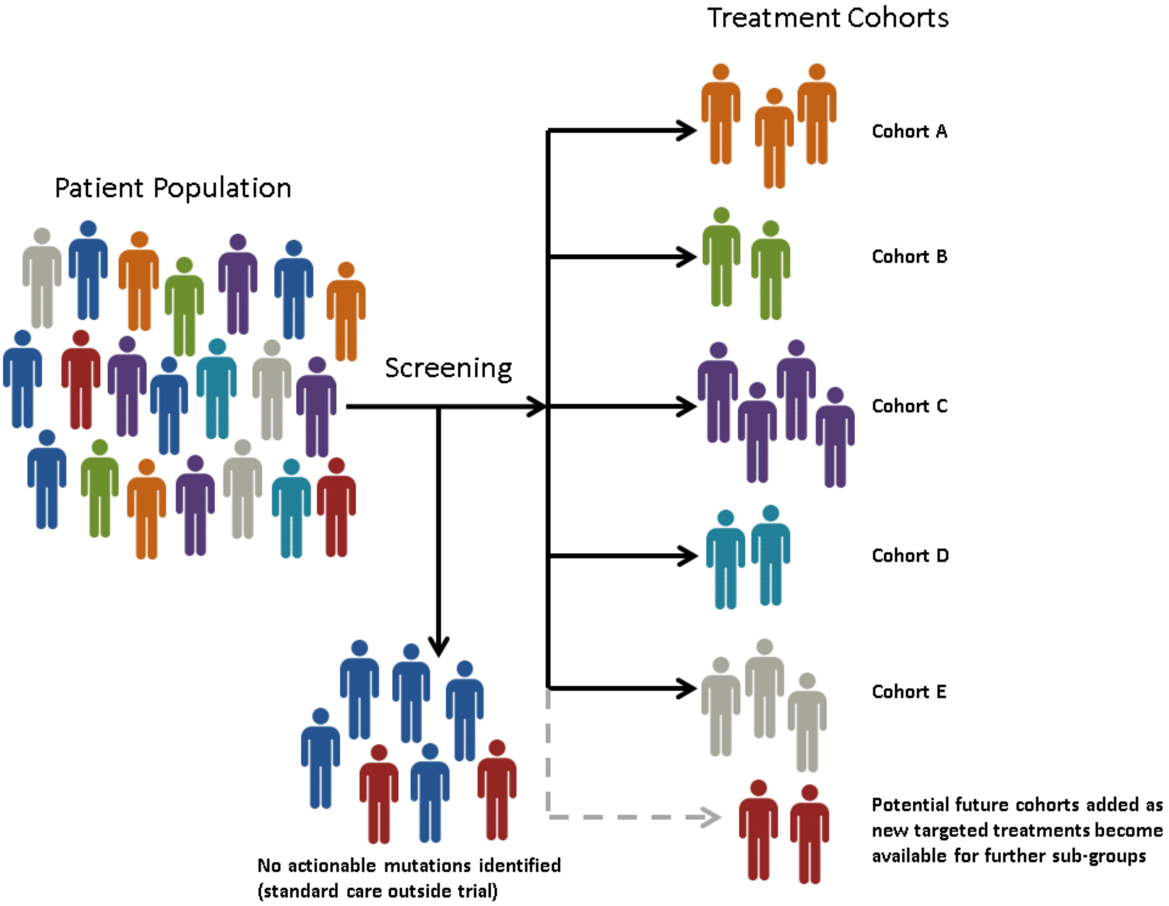

Figure 2. Schematic of plasmaMATCH umbrella clinical trial design

The ctDNA screening component of plasmaMATCH aims to assess whether screening for multiple rare and acquired genetic aberrations in advanced breast cancer purely with ctDNA assays presents a viable strategy to identify patients who will respond to specific matched targeted therapies. In addition, plasmaMATCH will seek to demonstrate the feasibility of ctDNA as a screening tool with the potential for future integration into routine NHS practice. This study will seek to address the major limitation of tissue biopsy based molecular screening for patients with advanced breast cancer. For many patients biopsy of recurrent cancer is not possible without an unacceptable risk of complications due to the site of the recurrence. Although a minority of patients do have a tissue biopsy performed at initial diagnosis of recurrence, a biopsy is very rarely repeated later in the treatment course. As tumour mutations may change through treatment, identifying which genetic aberrations are present in the tumour after prior treatment is important to guide treatment optimally. ctDNA assays present a way to identify the mutations currently driving the tumour, whereas it would not be possible or practical to biopsy tumours to direct therapy.

The therapeutic component of plasmaMATCH will include patient subgroups identified via ctDNA screening (or via tumour sequencing conducted outside the plasmaMATCH trial in the case of Cohort D) including subgroups characterised by the absence of a targetable mutation at ctDNA screening (initially olaparib plus AZD6738 in TNBC), and comprises of multiple parallel treatment cohorts defined according to the patient subgroup. Suitably matched patient subgroups and targeted treatment pairs have been chosen according to the likely relevance to the biology of breast cancer

and the availability of a therapeutic agent to target the relevant pathway. This will provide proof of principle efficacy for multiple potential therapeutic targets in advanced breast cancer and complement local and international clinical sequencing programmes based on tumour biopsy sequencing.

plasmaMATCH will allow appraisal of the overall approach of targeting genetically defined subgroups of advanced breast cancer through assessment of response, PFS and patterns of relapse, assessing the safety and tolerability of the agents under study and exploring mechanisms of resistance to therapies targeting these subgroups. The trial also has the potential to explore the impact of intra-tumoural genetic heterogeneity on response to targeted therapy, and will assess heterogeneity in change in tumour size between lesions in the ctDNA-driven cohorts in cancers with apparently clonal mutations and those with sub-clonal mutations.

2. TRIAL OBJECTIVES

2.1. Primary Objective

To assess the safety and activity profile of targeted therapies (initially including neratinib, AZD5363, fulvestrant, olaparib and AZD6738) in patient subgroups identified by ctDNA screening.

2.2. Secondary Objectives

2.2.1. ctDNA Screening Component

- To determine the frequency of targetable genetic mutations in a large population of patients with advanced breast cancer.
- To determine the proportion of patients with targetable mutations identified by ctDNA screening who enter the therapeutic component.
- To assess whether ctDNA is a feasible multi-centre screening tool for detecting aberrations in advanced breast cancer.
- To bank plasma samples to allow future development of ctDNA assays.

2.2.2. Therapeutic Component

- To determine whether ctDNA screening can select populations of patients who will be sensitive to targeted therapies.
- To determine whether serial ctDNA assessment on treatment can be used to monitor response and the development of resistance to targeted therapies.
- To assess the level of agreement between ctDNA screening and recurrent disease biopsies.
- To investigate whether patients with apparently clonal mutations in the tumour derive greater benefit from targeted therapy than patients with subclonal mutations.
- To determine the efficiency of the dynamic trial platform design in providing proof of principle efficacy for designated targeted therapies.

3. TRIAL DESIGN

The plasmaMATCH trial comprises a multiple parallel cohort, open-label, multi-centre phase IIa umbrella clinical trial and consists of a ctDNA screening component and a therapeutic component.

Both components are dynamic in design, such that new genetic aberrations or molecular subtypes paired with a targeted therapy may be added in the future.

3.1. ctDNA Screening Component

Approximately 50 UK Screening Sites will consent patients for plasmaMATCH ctDNA screening. Blood samples will be sent to the central laboratory for processing and ctDNA mutation analysis. Initially, up to 1150 patients will undergo ctDNA screening for activating mutations in PIK3CA, AKT1, ESR1 and HER2 and for HER2 copy number.

Patients who do not have a targetable mutation identified at ctDNA screening for entry into Cohorts A to D (or tumour sequencing for entry into Cohort D) will be treated as per standard of care outside the context of this trial, with the exception of patients with TNBC who may be eligible to enter Cohort E.

3.2. Therapeutic Component

Approximately 25 of the UK Screening Sites will also be designated as Treatment Sites and will enter patients (n=126 for Cohorts A to D, n = max. 69 for Cohort E) into the therapeutic component of plasmaMATCH, which incorporates multiple parallel cohorts defined by molecular target and therapy. Each cohort consists of either a conventional phase II single target group, driven by ctDNA screening (Cohorts A to C), a multi-target 'basket' driven by mutations identified either through plasmaMATCH ctDNA screening or sequencing of tumour tissue conducted outside of plasmaMATCH (Cohort D) and a TNBC cohort for those patients with TNBC and no targetable mutations identified for entry into Cohorts A to D or actionable mutations identified but not otherwise eligible for Cohorts A to D (Cohort E).

3.2.1. ctDNA-Driven Cohorts

- **Cohort A:** *ESR1* mutation identified in ctDNA screening treated with extended-dose fulvestrant.
- **Cohort B:** *HER2* mutation identified in ctDNA screening in patients with ER positive breast cancer treated with neratinib plus fulvestrant or in patients with ER negative breast cancer treated with neratinib only.
- **Cohort C:** *AKT1* mutation identified in ctDNA screening in patients with ER positive breast cancer treated with AZD5363 and fulvestrant.
- **Cohort E:** Absence of targetable mutations identified at ctDNA screening for entry into Cohorts A to D, or actionable mutations identified but not otherwise eligible for Cohorts A to D, and TNBC on most recent tumour biopsy treated with olaparib plus AZD6738.

3.2.2. Multi-Target 'Basket' Cohort

- **Cohort D:** AKT activation basket with mutations of *AKT1* identified in ctDNA screening in patients with ER negative breast cancer, mutations of *AKT1* identified in tumour sequencing conducted outside of plasmaMATCH in patients with ER positive or negative breast cancer, or *AKT2/3* E17K, *PIK3R1 or PTEN* mutations or homozygous deletion of *PTEN* in both ER positive and ER negative breast cancer identified in ctDNA screening or in prior tumour sequencing conducted outside of plasmaMATCH, treated with AZD5363.

Entry into all treatment cohorts will require provision of a mandatory baseline recurrent tumour biopsy (or archival recurrent tumour biopsy where deemed unsafe by the local Investigator) to complement the ctDNA screening samples and to enable analyses of heterogeneity. Archival primary tumour biopsies will also be collected on all patients at the time of entry into a treatment cohort.

Each cohort will utilise a primary endpoint of confirmed objective response rate according to RECIST v1.1 and will be analysed and reported independently from the other cohorts.

3.2.3. Hierarchy of Treatment Cohorts

If more than one mutation is identified at ctDNA screening, it is likely that more than one driver mutation has been identified in the tumour. The patient will be eligible to enter the treatment cohort in the order of priority defined below, provided that they meet all eligibility criteria for that treatment cohort and recruitment to the cohort remains open:

1. *AKT1* mutation in ER positive breast cancer (Cohort C) or *HER2* mutation (Cohort B)
2. AKT activation basket mutations (Cohort D)
3. *ESR1* mutation (Cohort A)

The hierarchy of mutations is determined by the frequency of each mutation, with priority given to the rarest mutations. In addition preliminary clinical trial data has identified a high response rate to AZD5363 in AKT1 mutant cancer, and neratinib in HER2 mutant cancer, justifying their inclusion in hierarchy level 1.

In the unlikely event that a patient has both an *AKT1* and *HER2* mutation, the local Principal Investigator (PI) will decide on the first cohort the patient will enter provided all other eligibility criteria are met.

Upon trial treatment discontinuation due to disease progression, patients with more than one actionable mutation identified by ctDNA screening may participate in a second cohort after the defined washout period specified in the inclusion criteria and provided they meet all other cohort specific eligibility criteria at the time of entry into the second cohort. For patients who have discontinued trial treatment due to unacceptable toxicity, upon subsequent disease progression those with more than one actionable mutation identified by ctDNA screening may participate in a second cohort.

3.2.4. Additional Actionable Mutations/Subtypes

plasmaMATCH ctDNA screening will also report *PIK3CA* mutation status to facilitate entry into trials outside of the context of plasmaMATCH. If a patient has a *PIK3CA* mutation identified at ctDNA screening but no other actionable mutation making them eligible for entry into a treatment cohort they should be considered for an alternative trial outside of plasmaMATCH.

Patients with *HER2* amplification identified in ctDNA screening who were not previously known to be HER2 positive should be advised by their treating clinician to consider tumour biopsy or testing of HER2 status on previously collected and stored tumour tissue, with the aim of confirmation of *HER2* amplification status followed by appropriate treatment off-trial. In such cases, follow up data including details of treatment, response and progression will be collected. Following a review of

HER2 amplification data by the TMG on 19 February 2018 it was decided that HER2 amplification recorded on ctDNA samples was insufficiently sensitive, and that reporting would cease. Patients with discordant results prior to cessation of reporting should be handled as documented above.

Additional phase II cohorts may be added by substantial amendment in the case of new diagnostic/therapeutic targets as the trial progresses, providing such an addition would not compromise the completion of recruitment to existing cohorts. Additional cohorts will be added based on: a) the feasibility and validity of testing for the defined molecular subtype or the relevant mutation based on ctDNA analysis; b) the pre-clinical data suggesting the biological relevance of the mutation/subtype; and c) the availability of an agent which targets the mutation or molecular subtype with sufficient phase I safety data to justify its inclusion.

3.2.5. Treatment Duration and Follow Up

Treatment on the trial will continue until disease progression according to RECIST v1.1, unacceptable toxicity or withdrawal of the patient's consent for any reason. Patients who discontinue trial treatment in the absence of disease progression should continue to have CT scans to assess disease status according to the trial assessment schedule, provided that the patient has not withdrawn their consent to further trial assessments.

Beyond the end of trial treatment, all patients should be followed up every 6 months until death or up to 2 years, including those who prematurely withdraw from trial treatment (unless the patient specifically withdraws consent for follow up). Patients will be asked to consent for future linkage with routinely collected health data via national registries such as the National Cancer Registration and Analysis Service (NCRAS), NHS Digital or NHS Care Records Service (NHS CRS) to trace their eventual vital status and assess subsequent unexpected co-morbidities.

4. TRIAL ENDPOINTS

4.1. Primary Endpoint

The primary endpoint will be determined for each cohort separately.

The primary end point for Cohorts A to E is confirmed objective response rate as defined by RECIST v1.1 for each cohort separately.

4.2. Secondary Endpoints

- Clinical benefit rate and PFS for each cohort separately.
- Safety and tolerability of therapies.
- Duration of response for each cohort.
- Frequency of mutations identified in ctDNA screening and the proportion of patients with a targetable mutation who enter the therapeutic component.
- Agreement between ctDNA mutation status and tissue mutation status for patients entering the therapeutic component.
- Pharmacokinetics in Cohorts A and B.

4.3. Exploratory Endpoints

- Comparison of overall response rate between cancers with apparently clonal mutations and those with sub-clonal mutations.
- Heterogeneity in change in tumour size between lesions in ctDNA-driven cohorts in cancers with apparently clonal mutations and those with sub-clonal mutations.
- Association between change in ctDNA abundance on treatment with maximum change in tumour size, overall response rate and PFS in cohorts individually and combined.
- The proportion of cancers with ctDNA detected HER2 amplification that have HER2 amplification on optional subsequent disease biopsy.
- Exploratory assessment of the pharmacodynamic effect of therapies in patients consenting to optional on-treatment biopsies.
- Exploratory assessment of mechanisms of resistance to therapies in optional biopsies taken at disease progression.
- Identification of a ctDNA biomarker that predicts for response to olaparib plus AZD6738.

5. SELECTION OF PATIENTS FOR REGISTRATION

5.1. Number of Patients

plasmaMATCH aims to recruit approximately 126 patients into Cohorts A to D as follows:
Cohort A – 78 patients
Cohort B – 16 patients
Cohort C – 16 patients
Cohort D – 16 patients

And up to a maximum of 69 patients into Cohort E:
Cohort E – max. 69 patients

In order to do so, it is expected that approximately 1150 patients will be required to be registered for ctDNA screening. Should no external factors (e.g. drug supply) determine closure of cohorts, entry into Cohort A will close once a total of 78 evaluable patients have been confirmed. For Cohorts B, C and D, a minimum of 16 evaluable patients will be required.

5.2. Source of Patients

Patients will be registered for ctDNA screening from approximately 50 UK Screening Sites. Potential participants will be identified in oncology clinics and discussed at multi-disciplinary team (MDT) meetings. Patients identified as having an actionable mutation via ctDNA screening (Cohorts A to D), or via tumour sequencing conducted outside of plasmaMATCH (Cohort D), or patients with TNBC with no actionable mutation identified at ctDNA screening or with an actionable mutation identified but not otherwise eligible for Cohorts A to D (Cohort E) will be invited to enter the therapeutic component of plasmaMATCH via a network of approximately 25 of the Screening Sites designated as Treatment Sites.

5.3. Eligibility Criteria for Registration

Patients will be considered eligible for registration into plasmaMATCH if they fulfil all eligibility criteria listed below and if a suitable cohort remains open to recruitment. Additional cohort specific eligibility criteria apply for entry into each treatment cohort. Full eligibility criteria for entry into each specific treatment cohort are presented in the relevant cohort sections.

Patients who fulfil the eligibility criteria will be given the opportunity to participate in plasmaMATCH if they have participated in other clinical trials prior to recruitment.

5.3.1. Inclusion Criteria for Registration

1. Signed Informed Consent Form for Registration and ctDNA Screening.
2. Female.
3. Aged ≥18 years old.
4. Histologically confirmed invasive breast carcinoma.
5. Metastatic or recurrent locally advanced breast cancer that is not suitable for treatment with radical or curative intent.
6. Demonstrated progression of disease by radiological assessment or suspicion of disease progression by clinical assessment following most recent line of therapy. *Patients may still be taking maintenance endocrine therapy and targeted therapy provided they have evidence of objective disease progression.*
7. Measurable disease by RECIST v1.1 or anticipation that the patient will have measurable disease on the next imaging. *If it is anticipated that the patient will have measurable disease on the next imaging this should be documented in the patient's notes by the PI or delegated Co-investigator.* Patients with bone only disease may be eligible if they have a soft tissue component that is measurable by RECIST v1.1. Any measurable lesion(s) that have previously been irradiated must have documented progression since the radiotherapy to be assessable.
8. Patients must have completed at least one prior line of treatment (chemotherapy, endocrine therapy or targeted therapy) for advanced breast cancer and/or relapse within 12 months of completing (neo)adjuvant chemotherapy. *Patients with HER2 positive breast cancer must have been treated with at least two lines of HER2 targeted therapy in the advanced setting (or one line if no further lines of HER2 targeted therapy are available locally).*
9. A maximum of two prior lines of cytotoxic chemotherapy, chemotherapy antibody-drug conjugate or immunotherapy alone in the advanced setting is permitted.
10. Patients must either be suitable for a baseline biopsy of recurrent disease or have an archival biopsy of recurrent disease available. *Patients who are approached for entry into a treatment cohort are requested to consent to a baseline biopsy but if deemed unsafe by the Investigator, an archival biopsy of recurrent disease can be used instead. If it is deemed unsafe to proceed with baseline biopsy, and no archival recurrent disease biopsy is available, the patient will not be eligible for entry into the treatment cohort.*
11. ECOG performance status ≤2.
12. Life expectancy >3 months.
13. Patients must be a) surgically sterile; b) have a sterilised sole partner; or c) be postmenopausal; or d) must agree to practice true abstinence; or e) use effective contraception during the period of trial treatment and be willing to do so for 6 months

following the end of trial treatment. *True abstinence must be in line with the preferred and usual lifestyle of the patient. (Periodic abstinence, such as calendar, ovulation, symptothermal, post-ovulation methods, and withdrawal are not acceptable methods of contraception). Effective contraception is defined as double barrier contraception (e.g. condom plus spermicide in combination with a diaphragm, cervical cap or intrauterine device). Ovarian suppression with a luteinizing hormone-releasing hormone (LHRH) agonist is not a method of contraception. Postmenopausal is defined by at least one of the following criteria:*

- Age >60 years;
- Age <60 years and cessation of regular menses for at least 12 consecutive months with no alternative pathological or physiological cause; and serum estradiol and follicle stimulating hormone (FSH) level within the laboratory's reference range for postmenopausal females;
- Documented bilateral oophorectomy.

5.3.2. Exclusion Criteria for Registration

1. Uncontrolled CNS disease (brain metastases or leptomeningeal disease). Patients with prior diagnosis of CNS metastases must be stable by clinical assessment having ceased steroids after prior treatment.
2. History of clinically significant or uncontrolled cardiac disease, including congestive heart failure, angina, myocardial infarction within the last 6 months or ventricular arrhythmia. *Patients with a history of any of the above listed cardiac conditions judged not to be clinically significant by the PI or delegated Co-investigator must be notified to the trial team at the ICR-CTSU for approval by the Chief Investigator (CI) and/or Cohort Lead prior to registration.*
3. Ongoing toxic manifestations of previous treatments Grade ≥1. Exceptions to this are alopecia or toxicities which in the opinion of the Investigator should not exclude the patient. *Such cases should be clearly documented in the patient's notes by the PI or delegated Co-investigator.*
4. Pregnant or breastfeeding.
5. Any condition that according to the treating physician may compromise the patient's safety or the conduct of the trial.
6. Current malignancies of other types, with the exception of adequately treated in situ carcinoma of the cervix and basal or squamous cell carcinoma of the skin. *Cancer survivors who have undergone potentially curative therapy for a prior malignancy and have no evidence of the disease for 3 years or more are eligible for the trial.*

Please note, the following exclusion criteria will apply for entry into a treatment cohort. These criteria should be considered at the time of registration for ctDNA screening with the expectation that the patient would not be excluded from entry into a treatment cohort based on these points:

1. Prior treatment with radiotherapy (except for palliative reasons), endocrine therapy, immunotherapy, chemotherapy or investigational medicinal products (IMPs) during the previous 4 weeks (6 weeks for nitrosoureas, Mitomycin-C) before trial treatment, except for hormonal therapy with LHRH analogues, which are permitted, and bisphosphonates or RANK ligand antibodies that are permitted for the management of bone metastases.

2. Major surgery (excluding minor procedures, e.g. placement of vascular access) within 4 weeks of the first dose of trial treatment.

5.4. Obtaining Informed Consent for Registration

The PI (or delegated individual) must ensure that each trial patient is fully informed about the nature and objectives of the trial and possible risks associated with participation.

Patients should be given the current ethics approved **plasmaMATCH Patient Information Sheet for Registration and ctDNA Screening** for their consideration. Patients should only be asked to provide consent for registration and ctDNA screening after they have had sufficient time to consider their participation and had the opportunity to ask any further questions.

No protocol required assessments, other than those required as part of standard patient care, should be conducted until the **plasmaMATCH Informed Consent Form for Registration and ctDNA Screening** has been signed and dated by both the patient and the Investigator.

Confirmation that the patient meets all eligibility criteria should be documented in the patient's medical notes by the PI or delegated Co-investigator, along with confirmation of the patient's consent for registration and ctDNA screening. One copy of the signed consent form should be provided to the patient, one copy should be filed in the patient's medical records and the original should be retained in the Site Investigator File, which should be available for verification by the trial team at the ICR-CTSU or for regulatory inspection at any time.

5.5. Pre-Registration Procedure

The following should be conducted within 14 days prior to registration:

- Informed consent for registration and ctDNA screening
- Complete medical history to confirm suitability for registration and ctDNA screening
- ECOG performance status

6. REGISTRATION PROCEDURE

The **ctDNA Screening Eligibility Checklist** (signed by the PI or delegated Co-investigator) must be completed prior to completing the **Registration Form**, and both must be completed prior to registration. Written confirmation that eligibility has been checked by an Investigator should also be documented in the patient's medical records.

Patients should be registered centrally with the ICR-CTSU before ctDNA screening blood samples are collected from patients and before entry into any treatment cohort.

Patients should be registered by telephoning ICR-CTSU on:

020 8643 7150

09.00-17.00 Monday to Friday

The following information will be required at registration:

- Name of hospital, Investigator and person registering patient

- Confirmation that patient has given written informed consent for trial registration and ctDNA screening
- Confirmation that the patient is eligible for trial registration by completion of the ctDNA screening eligibility checklist
- Patient’s full name, hospital number, date of birth, postcode and NHS/CHI number

The caller will be given the patient’s unique Registration Number and fax confirmation will be sent to the trial contact (Research Nurse/Trial Coordinator).

7. ctDNA SCREENING PROCEDURE FOLLOWING REGISTRATION

The blood samples for ctDNA screening will be sent to the central laboratory (the Molecular Diagnostics Laboratory at the Centre for Molecular Pathology, The Royal Marsden NHS Foundation Trust and The Institute of Cancer Research) for digital droplet PCR (ddPCR) analysis of the ctDNA screening blood samples within plasmaMATCH, and to an external laboratory based within the US who will conduct ctDNA next-generation sequencing with a CLIA certified assay within plasmaMATCH.

The procedures for collection, processing, storage, shipping and tracking of ctDNA screening blood samples are detailed in the plasmaMATCH Investigator Laboratory Manual.

7.1. Blood Sample Collection

The ctDNA screening blood sample should be collected and shipped in accordance with the instructions in the plasmaMATCH Investigator Laboratory Manual as soon as possible following registration of the patient.

40ml blood should be taken in the preservative tubes (as detailed in the Laboratory Manual) provided by the ICR-CTSU for plasma extraction and collection of buffy coat. All samples must be labelled with the unique patient Registration Number and date of birth to enable cross referencing.

Samples should be sent as per the instructions in the Laboratory Manual on the same day that they are taken unless otherwise specified in the Laboratory Manual. Please refer to the Laboratory Manual for further details.

Haemolysis should be avoided. Blood samples that are overtly haemolysed or under filled will be rejected and a further blood sample will be required in order to perform ctDNA screening for that patient. In some cases, for example if a sample fails testing, an additional sample may be required in order to complete ctDNA screening. Sites will be notified by the ICR-CTSU if a further blood sample is required.

7.2. ctDNA Analysis

The blood samples taken for ctDNA screening will be analysed centrally using appropriately validated assays. The central laboratory, the Clinical Pathology Accreditation (CPA) accredited Molecular Diagnostics Laboratory at the Centre for Molecular Pathology, The Royal Marsden NHS Foundation Trust and The Institute of Cancer Research, will extract the DNA from the plasma samples and

mutations and copy number events will be analysed by ddPCR. A blood sample will also be sent to an external laboratory based within the US who will conduct ctDNA next-generation sequencing.

7.3. ctDNA Screening Results

Results from the ctDNA analysis performed at ctDNA screening will be provided to the site by the ICR-CTSU. Patients will be offered entry into the open treatment cohorts based on eligibility.

If a patient has more than one actionable mutation identified at ctDNA screening please refer to the treatment cohort hierarchy in Section 3.2.3.

Direct entry into Cohort E is permitted for patients with TNBC with actionable mutations identified if the corresponding treatment Cohort is closed to recruitment.

8. SELECTION OF PATIENTS FOR ENTRY INTO TREATMENT COHORTS

Patients with actionable mutations identified at ctDNA screening (Cohorts A to D), or patients with TNBC with no actionable mutation identified at ctDNA screening or with an actionable mutation identified but not otherwise eligible for Cohorts A to D (Cohort E), will be offered entry into a treatment cohort at a Treatment Site. Patients identified at a Screening Only Site should be referred to a Treatment Site. Please refer to Section 16.1 for further details on Screening Only and Treatment Sites and the referral process. In order to proceed to entry into a treatment cohort at the Treatment Site the patient must fulfil the cohort specific eligibility criteria as presented in the relevant cohort section of the protocol.

In exceptional circumstances (for example if there is a delay in receipt of the ctDNA screening result) patients are permitted to have one intervening line of therapy (chemotherapy, endocrine therapy or trial treatment on a different cohort within plasmaMATCH or on another trial) between ctDNA screening and entry into a treatment cohort. Patients receiving an intervening line of therapy must demonstrate disease progression prior to entry into a treatment cohort. If a patient has more than one intervening line of therapy between ctDNA screening and entry into a treatment cohort, the actionable mutation must be re-confirmed by repeat ctDNA screening in order for the patient to be eligible.

8.1. Obtaining Informed Consent for Entry into the Treatment Cohorts – Treatment Sites Only

Consent for entry into the therapeutic component of plasmaMATCH must be obtained by the Treatment Site following receipt of the ctDNA screening result confirming the presence of an actionable mutation before the patient can be entered into a specific treatment cohort at the Treatment Site.

A separate Patient Information Sheet (PIS) and Informed Consent Form (ICF) exists for each treatment cohort with information relevant for entry into that specific cohort.

Please note: If a patient from a Screening Only Site has an actionable mutation identified at ctDNA screening (Cohorts A to D) or has TNBC and no actionable mutation identified at ctDNA screening or

has an actionable mutation identified but not otherwise eligible for Cohorts A to D (Cohort E), the site should initiate the consent process by providing the cohort specific PIS to confirm that the patient is willing to be referred for consent at the Treatment Site.

The PI (or delegated Co-investigator) must ensure that each trial patient is fully informed about the nature and objectives of the trial and possible risks associated with participation within an individual treatment cohort.

Patients should be given the current ethics approved **plasmaMATCH PIS for entry into the relevant treatment cohort** for their consideration. Patients should only be asked to consent to the entry into the treatment cohort after they have had sufficient time to consider their participation, and had the opportunity to ask any further questions.

No further protocol required assessments, other than those required as part of standard patient care, should be conducted until the **plasmaMATCH ICF for entry into the relevant treatment cohort** has been signed and dated by both the patient and the Investigator at the Treatment Site.

Confirmation that the patient meets all eligibility criteria must be documented in the patient's medical notes by the PI or delegated Co-investigator at the Treatment Site, along with confirmation of the patient's consent and the informed consent process for entry into the treatment cohort. One copy of the signed consent form should be provided to the patient, one copy should be filed in the patient's medical records and the original should be retained in the Site Investigator File, which must be available for verification by the trial team at the ICR-CTSU or for regulatory inspection at any time.

8.2. Registered Patients Who Do Not Consent to Treatment Cohort Entry

Patients may be registered for plasmaMATCH ctDNA screening but then decide not to consent for entry into a treatment cohort even though they have an actionable mutation identified at ctDNA screening, or have TNBC and no actionable mutation identified at ctDNA screening. The plasmaMATCH Trial Management Group (TMG) are interested in finding out the reasons for non-consent to the therapeutic component of the trial, therefore this information will be collected on the Patient Treatment Plan Form in the clinical trial database. Follow up data on the status of these patients will also be collected, patients will be asked to provide consent to this in the plasmaMATCH informed consent for registration and ctDNA screening.

9. TREATMENT COHORT ENTRY PROCEDURE – TREATMENT SITES ONLY

The **Cohort Specific Eligibility Checklist** (signed by the PI or delegated Co-investigator) must be completed prior to completing the **Treatment Cohort Entry Form**, and both must be completed prior to entry at the Treatment Site. Written confirmation that eligibility has been checked by an Investigator should also be documented in the patient's medical records.

Patients must be entered into the treatment cohort centrally with the ICR-CTSU by a Treatment Site before protocol trial treatment can commence.

Patients should be entered by telephoning ICR-CTSU on:

020 8643 7150

09.00-17.00 Monday to Friday

Entry into the treatment cohort should take place as close to the planned start date of trial treatment as possible.

The following information will be required at entry into a treatment cohort:

- Patient's unique Registration Number
- Patient's hospital number, initials, date of birth, postcode and NHS/CHI number for cross check with registration data and confirmation of patient details
- Name of hospital, Investigator and person entering patient
- Confirmation of the treatment cohort the patient is entering
- Confirmation that patient has given written informed consent for entry into the treatment cohort
- Confirmation that patient is eligible for entry into the treatment cohort by completion of the cohort specific eligibility checklist

The ICR-CTSU staff will give the caller the patient's unique trial identification number (Trial ID), and fax confirmation will be sent to the trial contact (Research Nurse/Trial Coordinator) and pharmacist to enable dispensing of the trial treatment.

FOLLOWING PATIENT ENTRY INTO THE TREATMENT COHORT PLEASE REFER TO THE APPROPRIATE TREATMENT COHORT SECTION AT THE BACK OF THIS PROTOCOL FOR FURTHER INSTRUCTION ON THE COHORT SPECIFIC TRIAL PROCEDURES.

10. PATIENTS WITH TUMOUR SEQUENCING RESULTS CONDUCTED OUTSIDE OF plasmaMATCH

10.1. Requirements for Entry into Cohort D

Patients with actionable mutations identified by tumour sequencing of a metastatic biopsy conducted outside of the plasmaMATCH trial may be eligible for entry into Cohort D without the requirement to undergo plasmaMATCH ctDNA screening, provided that:

- The sequencing has been carried out in a laboratory that operates to Good Clinical Laboratory Practice or appropriate clinical laboratory standards, such as Clinical Laboratory Improvement Amendments (CLIA), CPA or UKAS ISO15189:2012 Accreditation.
- Sequencing conducted outside the Royal Marsden NHS Foundation Trust must be with an assay CE-marked for the intended purpose.
- A copy of the report stating the actionable mutation identified by tumour sequencing has been reviewed and approved by the plasmaMATCH TMG.
- The patient meets all eligibility criteria for entry into Cohort D as described in Section D.

The patient will not be eligible to enter Cohort D if the tumour sequencing has not been carried out to the required laboratory standards specified above. In this case, the patient must be registered for ctDNA screening within plasmaMATCH (and the mutation identified in ctDNA) or the result must be confirmed in a laboratory that operates to the required standards in order to confirm eligibility prior to entry.

10.2. Obtaining Informed Consent

Consent for entry into Cohort D must be obtained as described in Section 8.1. Consent should be obtained from the patient before a copy of the tumour sequencing report is sent to the ICR-CTSU for TMG approval.

Please note: If the patient is identified at a Screening Only Site, the site should initiate the consent process by providing the Cohort D PIS to confirm that the patient is willing to be referred for consent at the Treatment Site.

10.3. Confirmation of Tumour Sequencing Results

To enter a patient into Cohort D based on a prior tumour sequencing result, the Treatment Site will be required to provide evidence of the actionable mutations identified by tumour sequencing and laboratory accreditation to the trial team at the ICR-CTSU in order to obtain approval from the plasmaMATCH TMG. Evidence of this approval must be documented in the patient's medical records.

10.4. Entry into Cohort D

Following receipt of approval of the tumour sequencing result from the trial team at the ICR-CTSU, and provided the patient meets all treatment Cohort D eligibility criteria, the patient can be entered into Cohort D. The Treatment Site should complete the **Registration Form for Entry into Cohort D**, the **Cohort D Eligibility Checklist** (signed by the PI or delegated Co-investigator) and the **Treatment**

Cohort Entry Form. Written confirmation that eligibility has been checked by an Investigator should also be documented in the patient's medical records.

Patients must be registered and entered into Cohort D centrally with the ICR-CTSU before protocol trial treatment can commence. Registration and entry into Cohort D should take place as close to the planned start date of trial treatment as possible.

Patients should be registered and entered by telephoning ICR-CTSU on:
020 8643 7150
09.00-17.00 Monday to Friday

Please inform the member of staff at the ICR-CTSU that the patient will be registered and entered into treatment Cohort D during the same phone call.

The Treatment Site will be required to complete the registration procedure first by providing the details as described in Section 6. The ICR-CTSU staff will give the caller the patient's unique Registration Number. The Treatment Site will then be required to complete the treatment cohort entry procedure by providing the details as described in Section 9.

The ICR-CTSU staff will give the caller the patient's Trial ID, and fax confirmation will be sent to the trial contact (Research Nurse/Trial Coordinator) and pharmacist to enable dispensing of the trial treatment.

FOLLOWING PATIENT ENTRY INTO COHORT D PLEASE REFER TO SECTION D OF THIS PROTOCOL FOR FURTHER INSTRUCTION ON THE COHORT SPECIFIC TRIAL PROCEDURES.

11. plasmaMATCH SCREENING LOG

All participating sites will be required to keep a log of all patients with advanced breast cancer who are identified at the MDT meeting to be potential candidates for this trial. The information collected on the log will include:

- Date patient identified at MDT
- Registration screening outcome (patient approached/accepted participation/declined participation)
- Reasons for not approaching/declining participation (if available)
- Date of registration and unique Registration Number (if patient goes on to be registered)

This information will be used to monitor recruitment activity. No patient identifiable data will be collected on the screening log.

12. PHARMACOVIGILANCE

12.1. Definitions

Adverse Event (AE)

An AE is any untoward medical occurrence in a patient or clinical trial subject; the event does not necessarily have a causal relationship with trial treatment or usage.

Serious Adverse Event (SAE)

An SAE is any untoward medical occurrence that occurs after the patient has provided written informed consent for entry into a treatment cohort and within 30 days of the last administration and:

- results in death;
- is life-threatening;
- requires hospitalisation or prolongation of existing inpatients' hospitalisation;
- results in persistent or significant disability or incapacity;
- or is a congenital anomaly or birth defect.

Important AEs that are not immediately life-threatening or do not result in death or hospitalisation but may jeopardise the patient or may require intervention to prevent one of the other outcomes listed in the definition above, may also be considered serious.

According to NCI CTCAE v4.0, Grade 4 events are life threatening events. Any Grade 4 event should therefore be considered an SAE.

Progression of the indicated disease and death due to progression of the indicated disease are not considered SAEs.

Pregnancy whilst participating in a trial is not itself considered an SAE but should be reported to the ICR-CTSU using the pregnancy reporting form and followed up for congenital anomalies or birth defects (See Section 12.8 for further information).

Serious Adverse Reaction (SAR)

A SAR is an SAE that is suspected as having a causal relationship to the IMP, as assessed by the Investigator responsible for the care of the patient. A suspected causal relationship is defined as possibly, probably or definitely related (see definitions of causality table).

Table 1. SAE reporting – definitions of causality

Relationship	Description
Unrelated	There is no evidence of any causal relationship with the trial drug.
Unlikely	There is little evidence to suggest there is a causal relationship (e.g. the event did not occur within a reasonable time after administration of the trial medication). There is another reasonable explanation for the event (e.g. the patient's clinical condition, other concomitant treatment).
Possible	There is some evidence to suggest a causal relationship (e.g. because the event occurs within a reasonable time after administration of the trial medication). However, the influence of other factors may have contributed to the event (e.g. the patient's clinical condition, other concomitant treatments).
Probable	There is evidence to suggest a causal relationship, and the influence of other factors is unlikely.
Definitely	There is clear evidence to suggest a causal relationship, and other possible contributing factors can be ruled out.
Not assessable	There is insufficient or incomplete evidence to make a clinical judgement of the causal relationship.

Suspected Unexpected Serious Adverse Reaction (SUSAR)

A SUSAR is a SAR, the nature or severity of which is not consistent with the safety information provided in the applicable Investigator Brochure or Summary of Product Characteristics (SmPC), and is assessed as unexpected by the CI.

Fatal reactions at least possibly related to the study drug have to be considered unexpected and reported as SUSARs.

Adverse Events of Special Interest in Cohort E

In addition, selected adverse events experienced by any patient who has provided written informed consent for entry into treatment Cohort E meeting any of the criteria listed below (Adverse Events of Special Interest (AESI)), are reportable to the ICR-CTSU in the same timeframe as SAEs as detailed in Section 12.3. Therefore for the purposes of pharmacovigilance reporting the below events should be considered serious:

- Any event of pneumonitis
- Any event of myelodysplastic syndromes (MDS)/acute myeloid leukaemia (AML)
- Any new primary malignancy

12.2. Reporting of Adverse Events to ICR-CTSU

Any toxicity, sign or symptom that occurs after the patient has provided written informed consent for entry into a treatment cohort and within 30 days (or 60 days for Cohort A) of the last administration of trial treatment, which is not unequivocally due to progression of disease, should be considered an AE.

All AEs must be reported on the relevant electronic case report form (eCRF) and submitted to ICR-CTSU.

The severity of AEs should be graded according to the National Cancer Institute Common Terminology Criteria for Adverse Events (NCI CTCAE) v4.0. For each AE, the highest grade observed since the last visit should be reported.

Abnormal laboratory findings that meet the criteria for Grade 2 or above toxicity as defined by NCI CTCAE v4.0 are considered to be clinically significant and should be reported as AEs.

Whenever one or more toxicity/sign/symptom corresponds to a disease or a well-defined syndrome only the main disease/syndrome should be reported.

12.3. Reporting of Serious Adverse Events to ICR-CTSU

Any SAE that occurs after the patient has provided written informed consent for entry into a treatment cohort and up to 30 days (or 60 days for Cohort A) following the last dose of trial treatment must be reported.

Any SAEs that occur more than 30 days after the last dose of trial treatment that, in the opinion of the PI, are related to the trial treatment should be reported to ICR-CTSU if the PI becomes aware of them.

All SAEs should be reported to ICR-CTSU within 24 hours of the PI (or delegated Co-investigator) becoming aware of the event, by completing the plasmaMATCH SAE form and faxing to:

The ICR-CTSU safety desk
Fax: 020 8722 4368
For the attention of the plasmaMATCH Trial Team

As much information as possible, including the Investigator's assessment of causality, must be reported to ICR-CTSU in the first instance. Additional follow up information including final diagnosis and outcome should be reported as soon as it is available.

All SAE forms must be completed, signed and dated by the PI or delegated Co-investigator.

All reported SAEs and follow up information for patients entered into a treatment cohort will be forwarded to the relevant pharmaceutical partner(s) upon receipt at ICR-CTSU.

12.4. Review of Serious Adverse Events

The CI (or designated representative) will assess all reported SAEs for causality. SAEs assessed as having a causal relationship to trial treatment will be evaluated for expectedness based on the reference safety information contained in the relevant Investigator Brochure (and SmPC if appropriate). (NB. The CI cannot downgrade the PI's assessment of causality).

SAEs assessed as having a causal relationship to trial treatment and as being unexpected (SUSARs) will undergo expedited reporting to the relevant authorities and all other interested parties by ICR-CTSU (see Section 12.5).

Sites should respond to requests from the CI or designated representative (via ICR-CTSU) for further information that may be required for final assessment of an SAE as soon as possible.

12.5. Expedited Reporting of SUSARs

If an SAE is identified as being a SUSAR by the CI, and is fatal or life threatening, it will be reported by ICR-CTSU to the MHRA, the main Research Ethics Committee (REC), the Co-sponsors, relevant pharmaceutical partner(s) and all other interested parties within 7 days of being notified of the event.

If an SAE is identified as a SUSAR by the CI, and is not fatal or life threatening, it will be reported by ICR-CTSU to the MHRA, the main REC, the Co-sponsors and relevant pharmaceutical partner(s) within 15 days of ICR-CTSU being notified of the event.

ICR-CTSU will report any additional relevant information to the MHRA, main REC, the Co-sponsors and pharmaceutical partner(s) as soon as possible, or within 8 days of the initial report of a fatal/life threatening SUSAR.

The PIs at all actively recruiting sites will be informed of any SUSARs occurring within the trial at appropriate intervals.

12.6. Follow up of Serious Adverse Events

SAEs should be followed up until clinical recovery is complete or until disease has stabilised. SAE outcomes should be reported to ICR-CTSU using the relevant section of the SAE form as soon as the PI or delegated Co-investigator becomes aware of the outcome.

12.7. Annual Reporting of Serious Adverse Reactions

An annual report will be provided to the MHRA and the main REC by ICR-CTSU and copied to the Co-sponsors at the end of the reporting year.

12.8. Reporting Pregnancies

If any trial patient becomes pregnant while receiving trial treatment or up to 90 days after receiving trial drug, this should be reported to ICR-CTSU using the pregnancy reporting form. Patients who become pregnant should discontinue trial treatment immediately. Pregnancies should be followed up until conclusion and all follow-up information should be reported to ICR-CTSU. If the outcome of the pregnancy meets the definition of serious (i.e. congenital abnormality) this should be reported to ICR-CTSU following the SAE reporting procedures described above.

Figure 3. Flow diagram for SAE reporting, and action following report

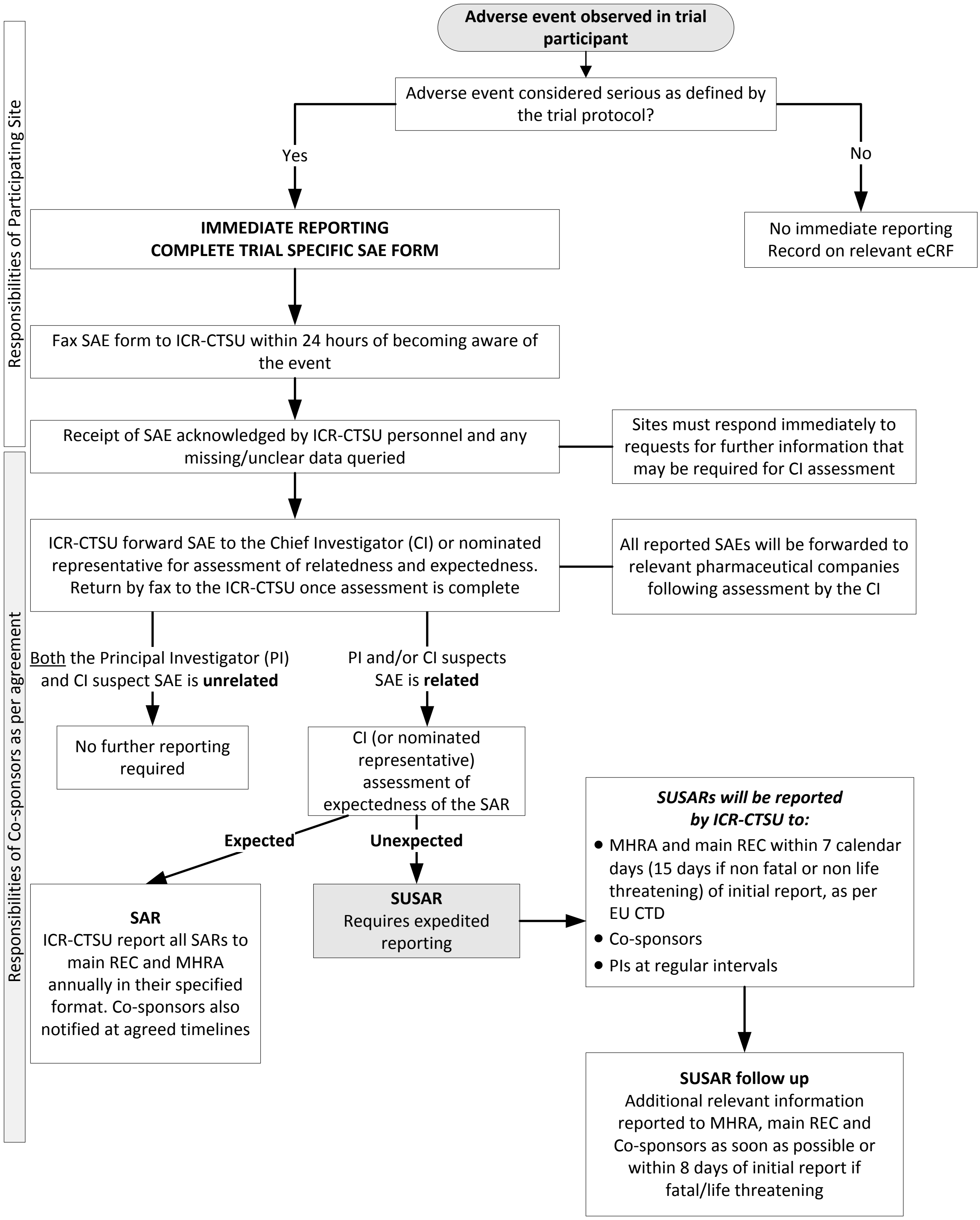

NB. All SAEs should continue to be followed up as specified above

13. STATISTICAL CONSIDERATIONS

13.1. Treatment Allocation

plasmaMATCH is a phase IIa umbrella clinical trial; all patients entered into a treatment cohort will receive the therapeutic agent(s) of interest for that cohort.

13.2. Statistical Design and Sample Size Justification

13.2.1. Screening Component

The aim is to recruit approximately 1150 patients into the screening component of the trial. This gives 85% probability of identifying 25 patients for a mutation with prevalence of 3% for each of Cohorts B, C and D individually, allowing for 36% attrition between ctDNA screening and the therapeutic component. For the *ESR1* mutation (Cohort A), assuming 20% prevalence there will be 80% probability of identifying 195 patients, allowing for 60% attrition between ctDNA screening and the therapeutic component (based on plasmaMATCH data to date). For the TNBC patients (Cohort E), assuming 25% prevalence there will be >99% probability of identifying 99 patients, allowing for 30% attrition between ctDNA screening and the therapeutic component (attrition rate expected to be lower as TNBC patients are less likely to have had a preceding treatment).

13.2.2. Therapeutic Component

The therapeutic component of plasmaMATCH is a phase IIa umbrella clinical trial comprising multiple parallel cohorts. Each cohort will recruit and be analysed independently; as such, no adjustment for multiple testing (across the cohorts) is included.

13.2.3. Cohort A

Effective targeting of mutant ER should manifest as objective responses to treatment; hormone therapy in endocrine naïve cancer has a 35% response rate (23). The response rate of fulvestrant in unselected patients is 10%, and a response rate of 20% would indicate clear evidence of increased activity in ESR1 mutant cancer, provide evidence that fulvestrant is an alternative to regimens with greater systemic toxicity, and be sufficient to justify development of further, randomised, studies. For Cohort A, a single-stage A'Hern design will be used with a target response rate of 20% and unacceptable response rate of 10% (response rate in unselected but less pre-treated populations). Assuming alpha=0.05, beta=0.2, p0=0.1, p1=0.20, 13 responses out of 78 evaluable patients will be required to infer a level of efficacy for fulvestrant in this cohort of patients. The original sample size of 40 patients for Cohort A (protocol v1.2 dated 18 August 2016) was estimated on the basis of detecting a response rate of at least 25% in a population of patients assumed to have predominantly clonal mutations. However, as the trial has progressed it is apparent that the ctDNA screening may be detecting sub-clonal ESR1 mutations as well, for whom the response rate to fulvestrant would be expected to be lower (assumed 15%). Assuming a 50% prevalence of clonal versus sub-clonal mutations (20) , the revised target response rate for the whole Cohort A is therefore 20% ((25% + 15%)/2). In addition to the primary endpoint analysis of Cohort A, a retrospective exploratory analysis will be conducted of response rate in patients designated as having clonal or sub-clonal mutations, and these sub-populations will be analysed in separate subgroup analyses as described in section 13.3.3. It is recognised that the statistical power for the analysis of the sub-clonal mutations alone will be low (around 30%), due to the lower expected response rate in this subgroup.

13.2.4. Cohorts B, C and D

A single stage A'Hern design will be used for Cohorts B, C and D. For each of these cohorts a target response rate of p1=0.25 with an unacceptable response rate of p0=0.05 will be used. With alpha=0.05 and beta=0.2, 3 responses out of 16 evaluable patients will be required to infer a level of efficacy of the agent of interest in that cohort of patients.

13.2.5. Cohort E

Cohort E will recruit patients with TNBC who do not have an actionable mutation identified at ctDNA screening for entry into treatment Cohorts A to D, or who have an actionable mutation identified but are not otherwise eligible for Cohorts A to D, to receive the combination of olaparib plus AZD6738. Cohort E will use a two-stage design (see Figure E2 in the Cohort E section of this protocol) based on H_0: p_0=0.10; H_a; p_1=0.25; α=0.02 and β=0.10. It will recruit a maximum of 69 patients (37 in Stage 1 and 32 in Stage 2) with an initial efficacy level of 25% and an inefficacy level of 10%. An upper efficacy bound of 50% will be used, above which broad activity will be concluded, with no more patients entered and a subsequent trial will be developed in an unselected TNBC population.

Stage 1

37 patients will be entered into the first stage of Cohort E. When all 37 patients have been recruited an interim analysis will take place to evaluate the response rate:

- If there are 4 or fewer responses observed, given the efficacy level of 25% and inefficacy level of 10%, inactivity will be concluded. Cohort E will be terminated and no further patients will be entered into the Cohort.
- If there are 19 or more responses, broad activity will be concluded and a subsequent randomised Phase III trial will be developed in an unselected TNBC patient population.
- If the number of responses observed is between 5 to 18 inclusive, patient accrual will continue into Cohort E Stage 2 as described below.

Stage 2

- If Cohort E progresses to Stage 2, a further 32 patients will be recruited, giving a total of 69 patients. If 12 or fewer responses are observed, the null hypothesis will be accepted.
- If 35 or more responses are observed, broad activity will be concluded and a subsequent randomised Phase III trial will be developed in unselected TNBC patients.
- If the number of responses observed is between 13 and 34 inclusive, an investigation into the identification of potential biomarkers of response will be carried out. If a potential biomarker subgroup is found, a future study to prospectively validate the potential biomarker will be developed.

Cohort E will include a maximum of 15 patients with known BRCA1/2 germline mutations at the time of entry into Cohort E.

13.3. Endpoint Definitions

13.3.1. Primary Endpoint

The primary endpoint will be determined for each cohort separately.

Primary end point for Cohorts A to E: confirmed objective response rate as defined by RECIST v1.1 for each cohort separately

A patient will be said to have had an objective response if they have a complete/partial response at any point during trial treatment. The proportion with objective response will be presented along with the corresponding exact two-sided 95% confidence interval separately for each cohort.

13.3.2. Secondary Endpoints (for Each Cohort Separately)

Clinical benefit rate and progression free survival

A patient will be defined as having clinical benefit if they have either a complete/partial response or stable disease as defined by RECIST v1.1 lasting at least 24 weeks. The duration of clinical benefit is measured from the first documentation of RECIST complete/partial response or stable disease (whichever status is recorded first) until the first date that recurrence or progressive disease is objectively documented, taking as reference for progressive disease the smallest measurements recorded since the treatment started. The proportion with clinical benefit will be presented along with the corresponding exact two-sided 95% confidence interval separately for each cohort.

PFS will be measured from the date of entry into the treatment cohort until first date of either confirmed progressive disease according to RECIST criteria or death. Patients who remain alive and have not progressed will be censored at time of last follow up. A Kaplan Meier graph and median survival time will be presented separately for each cohort. The proportion of patients alive and progression-free at 6 months will also be presented with associated 95% confidence interval.

Safety and tolerability of therapies

Safety will be assessed throughout the treatment period using the NCI CTCAE v4.0 and summarised in tabular format. Reported toxicities will be coded using MedDRA (current version). For each agent, the proportion of patients reporting a dose reduction/delay during trial treatment will be presented.

Duration of response

The duration of response is measured from the time of first documentation of RECIST complete/partial response (whichever status is recorded first) until the first date that recurrence or progressive disease is objectively documented, taking as reference for progressive disease the smallest measurements recorded since the treatment started. Median duration of response and interquartile range will be presented along with its 95% confidence interval.

Frequency of mutations identified in ctDNA screening and the proportion of patients with a targetable mutation who enter the treatment cohorts

The proportion of patients undergoing ctDNA screening who have each targetable mutation of interest will be presented. The proportion of patients with a targetable mutation who enter the relevant therapeutic cohort will also be presented. Proportions will be presented with corresponding exact two-sided 95% confidence interval.

Agreement between ctDNA mutation status and tissue mutation status for patients entering the therapeutic component

The proportion of cancers with a ctDNA detected mutation that have a matching mutation on subsequent tissue biopsy will be presented with associated exact two-sided 95% confidence interval.

Pharmacokinetics Cohorts A and B

Changes in pharmacokinetic measures during the treatment period will be displayed graphically per patient. Values at specific time points will be summarised across all patients using the mean, standard deviation and range. Analyses will be performed separately for patients in Cohorts A and B.

13.3.3. Exploratory Endpoints

Comparison of overall response rate between cancers with apparently clonal mutations and those with sub-clonal mutations

Objective response rate will be calculated as for the primary endpoint and presented separately for patients with clonal and sub-clonal mutations within each cohort.

Heterogeneity in change in tumour size between lesions in ctDNA-driven cohorts in cancers with apparently clonal mutations and those with sub-clonal mutations

Waterfall plots will be used to display the percentage change in the sum of target marker lesions between baseline and 24 weeks (or time of progression if sooner), with shading used to indicate whether the mutation was clonal or sub-clonal.

Association between change in ctDNA abundance on treatment with maximum change in tumour size, overall response rate and progression free survival

Scatter plots will be used to display the relationship between ctDNA abundance and maximum reduction in the sum of target marker lesions. Box plots will be used to summarise ctDNA abundance in patients with and without objective response. To investigate the relationship between ctDNA abundance and PFS, a Cox proportional hazard model will be fitted with ctDNA as a continuous variable. Analyses will primarily be performed using data from all cohorts combined, with descriptive techniques also used to describe the associations within in each cohort separately. Consideration will be given to log or other transformation of ctDNA abundance data as appropriate.

The proportion of cancers with ctDNA detected *HER2* amplification that have *HER2* amplification on optional subsequent disease biopsy

The proportion of cancers with ctDNA detected *HER2* amplification that have *HER2* amplification on subsequent disease biopsy will be presented with associated exact two-sided 95% confidence interval.

Exploratory assessment of the pharmacodynamic effect of therapies in patients consenting to optional on-treatment biopsies

Analysis methods for additional exploratory endpoints investigating pharmacodynamic effects of therapy in relation to the additional optional biopsy will be defined in the statistical analysis plan.

Exploratory assessment of mechanisms of resistance to therapies in optional biopsies taken at disease progression

Analysis methods for additional exploratory endpoints investigating mechanism of resistance in relation to the additional optional biopsy will be defined in the statistical analysis plan.

Identification of a ctDNA biomarker that predicts for response to olaparib plus AZD6738
Provided that Cohort E is not halted for inactivity, upon Cohort E completion potential biomarkers of response will be analysed to identify a biomarker in ctDNA that could subsequently be validated in a future cohort.

The aim will be to develop a ctDNA classifier that identifies a subgroup with a response rate of 40% or more. The biomarker defined subgroup satisfying this criteria will be selected for validation in a subsequent future cohort that will prospectively recruit the specific biomarker subgroup.

13.4. Statistical Analysis Plan

Further details of analysis methods will be specified in a statistical analysis plan in accordance with ICR-CTSU Standard Operating Procedures.

13.5. Interim Analyses and Stopping Rules

Cohorts A, B, C and D utilise a single stage design and as such no formal stopping rule is incorporated. Cohort E utilises a two-stage design and will recruit a maximum of 69 patients with various stopping rules as described in Section 13.2.5. An Independent Data Monitoring Committee (IDMC) will meet regularly to review emerging safety and efficacy data from all cohorts. In addition, a Safety Review Committee (SRC) consisting of key members of the trial team will meet to perform an initial review of the safety and tolerability of extended-dose fulvestrant in Cohort A. The SRC may also review emerging safety data from other cohorts if deemed appropriate by the IDMC.

14. TRIAL MANAGEMENT

14.1. Trial Management Group

A TMG will be established and will include the CI, Coordinating Investigator, ICR-CTSU Scientific Lead, Cohort Clinical Leads, Co-investigators and identified collaborators, the Trial Operations Lead, Trial Statistician, Senior Trials Manager and Trial Manager. PIs, Investigators and key trial personnel will be invited to join the TMG as appropriate to ensure representation from a range of sites and professional groups. The TMG will meet at regular intervals, and at least annually. Notwithstanding the legal obligations of the Co-sponsors and CI, the TMG have operational responsibility for the conduct of the trial. The Group’s terms of reference, roles and responsibilities will be defined in a charter issued by ICR-CTSU.

14.2. Trial Steering Committee

The trial will be monitored by the generic ICR-CTSU Breast Systemic Therapy Trials Steering Committee (TSC). The TSC will meet at regular intervals, and at least annually. The TSC will provide expert independent oversight of the trial on behalf of the Co-sponsors and funder. The Committee’s terms of reference, roles and responsibilities will be defined in a charter issued by ICR-CTSU and based on MRC Good Clinical Practice (MRC GCP).

14.3. Independent Data Monitoring Committee

An IDMC will be set up for the plasmaMATCH trial and will comprise a Chairman and at least two further members with clinical or statistical expertise (at least one member must be a statistician). Membership of the IDMC will be proposed by the TMG and approved by the TSC. The IDMC will meet in confidence at regular intervals, and at least annually. A summary of findings and any recommendations will be produced following each meeting. This summary will be submitted to the TMG and TSC, and if required, the main REC and the MHRA. The IDMC will reserve the right to release any data on outcomes or side effects through the TSC to the TMG (and if appropriate to patients) if the IDMC determines at any stage that the combined evidence from this and other studies justifies the release of data. The Committee's terms of reference, roles and responsibilities will be defined in a charter issued by ICR-CTSU.

14.4. Safety Review Committee

A SRC will be set up that will include key members of the trial team. The Committee will meet to review the initial safety and tolerability data from patients on extended-dose fulvestrant in Cohort A after 3 patients have received 2 cycles of trial treatment and then again after 6 patients have received 2 cycles of trial treatment, with a further review after 40 patients have received 2 cycles of trial treatment (the target sample size for Cohort A in protocol v1.2). The SRC will specifically review 60 day toxicity data in the first 3 patients stopping Cohort A treatment to identify any residual toxicity. The SRC may also review emerging safety data from other treatment cohorts if deemed appropriate by the IDMC. The Committee's terms of reference, roles and responsibilities will be defined in a charter issued by ICR-CTSU.

15. RESEARCH GOVERNANCE

15.1. Co-sponsor Responsibilities

The Co-sponsors of the plasmaMATCH trial are The Institute of Cancer Research (ICR) and The Royal Marsden NHS Foundation Trust. Sponsor responsibilities, as defined by The Medicines for Human Use (Clinical Trials) Regulations 2004 as amended, are allocated between the Co-Sponsors as set out in a co-sponsorship agreement between ICR and The Royal Marsden NHS Foundation Trust.

15.2. Participating Site Responsibilities

Responsibilities delegated to participating sites are defined in an agreement between the Co-sponsors and the individual site.

15.3. AstraZeneca Responsibilities

AstraZeneca is responsible on behalf of the Co-sponsors for the manufacture, packaging, labelling and distribution of fulvestrant, AZD5363, olaparib and AZD6738 to participating sites in accordance with Good Manufacturing Practice and all applicable local legislation. Responsibilities are defined in an agreement between AstraZeneca and the Co-sponsors.

15.4. Puma Biotechnology Responsibilities

Puma Biotechnology is responsible on behalf of the Co-sponsors for the manufacture, packaging, labelling and distribution of neratinib to participating sites in accordance with Good Manufacturing

Practice and all applicable local legislation. Responsibilities are defined in an agreement between Puma Biotechnology and the Co-sponsors.

16. TRIAL ADMINISTRATION AND LOGISTICS

16.1. plasmaMATCH Participating Sites

plasmaMATCH will be opened across a network of approximately 50 UK Screening Sites, of which approximately 25 sites will also be designated as Treatment Sites. It is the expectation that the Screening Only Sites will refer patients with actionable mutations identified (Cohorts A to D) or TNBC with no actionable mutation identified at ctDNA screening or with an actionable mutation identified but not otherwise eligible for Cohorts A to D (Cohort E) to the Treatment Sites for entry into a treatment cohort and trial treatment administration. After completion of trial treatment the patient will be transferred back to the Screening Only Site for follow up.

16.1.1. Screening Only Sites

Screening Only Site selection will be based on proximity to and natural referral into an open Treatment Site. The pairing of Screening Only and Treatment Sites will be determined at the time of site activation of the Screening Only Site.

Screening Only Centres will perform the following tasks:

- Approach patients and obtain consent for registration and ctDNA screening
- Register patients for ctDNA screening
- Collect the ctDNA screening blood sample from the patient, send to the central laboratory for analysis and receive results from the ICR-CTSU
- Initiate the consent process for entry into the relevant treatment cohort by providing the cohort specific PIS to potentially eligible patients in order to confirm that the patient is willing to be referred for consent at the Treatment Site
- Referral of patients to the Treatment Site for consent and entry into the relevant treatment cohort as per local practice
- Follow up once patient care is transferred back from the Treatment Site, which can occur once the patient has completed trial treatment and has been followed up for a period of 30 days (or 60 days for Cohort A)

Please note: Screening Only Sites should not consent patients for entry into a treatment cohort. This task should **only** be performed by the Treatment Site who will be responsible for entry of the patient into the treatment cohort and administration of trial treatment.

16.1.2. Treatment Sites

Treatment Site selection will be based on experience in the delivery of early phase breast cancer trials and the ability to receive referrals from the Screening Only Sites.

Treatment Sites will perform the following tasks:

- Approach patients and obtain consent for registration and ctDNA screening
- Register patients for ctDNA screening

- Collect the ctDNA screening blood sample from the patient, send to the central laboratory for analysis and receive results from the ICR-CTSU
- Obtain consent from potentially eligible patients for entry into the relevant treatment cohort, including those patients referred from a Screening Only Site
- Entry of consenting patients into the relevant treatment cohort
- Trial treatment administration
- Follow up of all patients entered into a treatment cohort until the end of trial treatment visit 30 days (or 60 days for Cohort A) after the last administration of trial treatment, after which point follow up should be continued either at the Treatment Site or at the original referring Screening Only Site if applicable

16.2. Site Activation

Before recruitment can commence at a site, the site agreement must have been signed by all required signatories, the required trial documentation (as specified by ICR-CTSU) must be in place and a site initiation must have taken place. Site initiation may be by teleconference or by on-site visit if requested by the PI or if deemed appropriate by ICR-CTSU. ICR-CTSU will provide the final confirmation that recruitment can commence at a site. This confirmation should be filed in the Site Investigator File.

16.3. Data Acquisition

eCRFs will be used for the collection of trial data from sites and data should be entered into the clinical trial database in a timely manner. ICR-CTSU will provide guidance to sites to aid the completion of the eCRFs. The TMG reserves the right to amend or add to the eCRF template as appropriate. Such changes do not constitute a protocol amendment, and revised or additional forms should be used by sites in accordance with the guidelines provided by ICR-CTSU.

16.4. Central Data Monitoring

Once data has been entered on the eCRF by the site personnel, ICR-CTSU will review it for protocol compliance and for inconsistent or missing data. Should any missing data or data anomalies be found, queries will be raised for resolution by the site. Any systematic inconsistencies identified through central data monitoring may trigger an on-site monitoring visit.

16.5. On-Site Monitoring

If a monitoring visit is required, ICR-CTSU will contact the site to arrange the visit. Once a date for the visit has been confirmed, the site should ensure that full medical records of patients selected for source data verification are available for monitoring.

ICR-CTSU staff conducting on-site monitoring will review essential documentation and carry out source data verification to confirm compliance with the protocol. If any problems are detected during the course of the monitoring visit, ICR-CTSU will work with the PI and/or delegated individual to resolve issues and determine appropriate action.

16.6. Completion of the Trial and Definition of Trial End Date

The trial end date is deemed to be the date of last data capture.

16.7. Archiving

Essential trial documents should be retained according to local policy and for a sufficient period for possible inspection by the regulatory authorities (at least 5 years after the date of last data capture). Documents should be securely stored and access restricted to authorised personnel.

17. PATIENT PROTECTION AND ETHICAL CONSIDERATIONS

17.1. Trial Approvals

This trial has been formally assessed for risk by ICR-CTSU.

ICR-CTSU, on behalf of the Co-sponsors, will ensure that the trial has received ethics approval from a REC for multi-centre trials, regulatory approval from the MHRA and the relevant NHS permissions. Before recruiting patients, the PI at each site is responsible for obtaining local approvals.

17.2. Trial Conduct

This trial will be conducted according to the approved protocol and its amendments, supplementary guidance and manuals supplied by the Co-sponsors and in accordance with The Medicines for Human Use (Clinical Trials) Regulations 2004 as amended, the Research Governance Framework for Health and Social Care and the principles of GCP.

17.3. Informed Consent

Patients should be asked to provide consent for a trial in a two-step process. Patients should be asked to sign the current ethics approved **plasmaMATCH ICF for Registration and ctDNA Screening** prior to registration and ctDNA screening. Patients with actionable mutations identified at ctDNA screening should be asked to sign the current ethics approved **plasmaMATCH ICF for Entry into the Specific Treatment Cohort** prior to trial entry. Consent should only be taken after the patient has received both verbal and written information about the trial and had sufficient time to consider this information. All consent forms must be countersigned and dated by the PI or a designated individual. A signature log of delegated responsibilities, listing the designated individuals and the circumstances under which they may countersign consent forms, must be maintained at the participating site. This log, together with original copies of all signed patient consent forms, should be retained in the Site Investigator File and must be available for inspection. The current ethics approved plasmaMATCH PIS should be provided in addition to any standard PIS that is provided by the site and used in routine practice.

17.4. Patient Confidentiality

Patients will be asked to consent to their full name being collected at trial entry in addition to their date of birth, hospital number, postcode and NHS number or equivalent to allow linkage with routinely collected NHS data.

Each Investigator should keep a separate log of all patients' Registration Numbers, Trial IDs, names, addresses and hospital numbers. The Investigator must retain trial documents (e.g. patients' written consent forms) in strict confidence. The Investigator must ensure the patients' confidentiality is maintained at all times.

Representatives of ICR-CTSU and the regulatory authorities will require access to patients' hospital notes for quality assurance purposes. ICR-CTSU will maintain the confidentiality of patients at all times and will not reproduce or disclose any information by which patients could be identified.

17.5. Data Protection

ICR-CTSU will comply with all applicable data protection laws.

17.6. Insurance and Liability

Indemnity to meet the potential legal liability of Investigators participating in this trial is provided by the usual NHS indemnity arrangements.

18. FINANCIAL MATTERS

This trial is Investigator designed and led and has been approved and funded by the Clinical Research Committee (CRC) of Cancer Research UK.

ICR has received funding from Cancer Research UK for the central coordination of the trial. The trial meets the criteria for R&D support as outlined in the Statement of Partnership on Non-Commercial R&D in the NHS in England. The trial is part of the National Institute for Health Research Clinical Research Network (NIHR CRN). NIHR CRN resources should therefore be made available for the trial to cover UK specific research costs.

The Co-sponsors have received Investigator initiated research grants from both AstraZeneca and Puma Biotechnology to conduct the plasmaMATCH trial.

19. PUBLICATION POLICY

The main trial results for each treatment cohort will be published either separately or together in a peer-reviewed journal, on behalf of all collaborators. The manuscript will be prepared by a writing group, consisting of members of the TMG. Participating clinicians may be selected to join the writing group on the basis of intellectual and time input. All participating clinicians will be acknowledged in the publication. The results of the screening component, individual cohorts and translational research may be published together or individually.

Any presentations and publications relating to the trial must be authorised by the TMG. Authorship of any secondary publications, e.g. those relating to sub-studies, will reflect intellectual and time input into these studies.

No Investigator or other collaborator may present or attempt to publish data relating to the plasmaMATCH trial without prior permission from the TMG.

COHORT A: *ESR1* MUTATION TREATED WITH EXTENDED-DOSE FULVESTRANT

A1. COHORT A BACKGROUND AND RATIONALE

Mutations in *ESR1*, the gene encoding the ER, occur in up to 20% of endocrine resistant ER positive breast cancers, with ligand-binding domain mutations in *ESR1* resulting in ligand-independent activation of the ER (7, 21, 22). Fulvestrant is a selective estrogen receptor degrader (SERD), with mutated-ER cells remaining sensitive to high concentrations of fulvestrant *in vitro,* but being relatively resistant to the lower concentrations of fulvestrant (7). Standard dose fulvestrant may not achieve the concentration required to inhibit mutant ER, with phase III trials suggestive of dose-dependent response (23-25). The plasmaMATCH trial will assess the activity of extended-dose fulvestrant (double the current standard dose achieved by doubling the frequency of administration) in *ESR1* mutant breast cancer.

Cancers with acquired ligand-binding domain *ESR1* mutations are likely to be highly dependent on ER. Effective targeting of mutant ER should manifest as increased response rate. Hormone therapy in endocrine naïve cancer has a 35% response rate (26). An improvement in response from 10% to an observed response rate of 20% would indicate clear evidence of increased activity in *ESR1* mutant cancer, provide evidence that fulvestrant is an alternative to regimens with greater systemic toxicity, and be sufficient to launch further randomised studies. The original sample size of 40 patients for Cohort A was estimated on the basis of detecting a response rate of at least 25% in a population of patients assumed to have predominantly clonal mutations. However, as the trial has progressed it is apparent that the ctDNA screening may be detecting sub-clonal *ESR1* mutations as well, for whom the response rate to fulvestrant would be expected to be lower (assumed 15%). Assuming a 50% prevalence of clonal versus sub-clonal mutations (20), the revised target response rate for the whole Cohort A is therefore 20% ((25% + 15%)/2). In addition to the primary endpoint analysis of Cohort A, a retrospective exploratory analysis will be conducted of response rate in patients designated as having clonal or sub-clonal mutations.

A2. KNOWN RISKS AND BENEFITS OF FULVESTRANT

Fulvestrant is an intramuscularly administered SERD which binds competitively to the ER leading to its degradation (27). Fulvestrant has been the subject of a number of phase III trials where at a dose of 250mg it demonstrated similar efficacy to aromatase inhibitors in women with advanced disease who had progressed on prior endocrine therapy (23, 24). The subsequent CONFIRM trial in a similar population demonstrated a small but statistically significant survival advantage for fulvestrant 500mg versus 250mg with median overall survival improved to 26.4 months compared to 22.3 months (HR 0.81, 95% CI 0.69-0.96, p=0.02) (25). In all of these trials at both dose levels fulvestrant has been very well tolerated with minimal side effects. Fulvestrant has not been tested in a specific population of patients with *ESR1* mutations, but *in vitro* data suggest a potential dose response (7). Fulvestrant is not given at the maximum tolerated dose and this trial will assess a more frequent administration of fulvestrant with the aim of achieving greater drug exposure. The Cohort A treatment regimen is estimated to give an exposure double that achieved by fulvestrant at the UK licensed standard dose, which will be confirmed by steady state PK assessment. Cancers with *ESR1* mutations are predicted to be sensitive to fulvestrant, but with a dose response that implies that a dose of fulvestrant higher than that given as part of standard of care may increase response.

A3. COHORT A SPECIFIC ELIGIBILITY CRITERIA

Patients will be considered eligible for entry into treatment Cohort A if they fulfil all eligibility criteria presented below.

A3.1. Inclusion Criteria

1. *ESR1* mutation identified in plasmaMATCH ctDNA screening. *If a patient has more than one intervening line of therapy between ctDNA screening and entry into the treatment cohort, the actionable mutation must be re-confirmed by repeat ctDNA screening prior to entry.*
2. Signed Informed Consent Form for Entry into Treatment Cohort A.
3. Female.
4. Aged ≥18 years old.
5. Histologically confirmed invasive breast carcinoma.
6. Metastatic or recurrent locally advanced breast cancer that is not suitable for treatment with radical or curative intent.
7. Radiological evidence of disease progression following most recent line of therapy.
8. Measurable disease by RECIST v1.1 assessed by CT and/or MRI. Patients with bone only disease may be eligible if they have a soft tissue component that is measurable by RECIST v1.1. Any measurable lesion(s) that have previously been irradiated must have documented progression since the radiotherapy to be assessable.
9. Patients must have completed at least one prior line of treatment (chemotherapy, endocrine therapy or targeted therapy) for advanced breast cancer and/or relapse within 12 months of completing (neo)adjuvant chemotherapy. *Patients with HER2 positive breast cancer must have been treated with at least two lines of HER2 targeted therapy in the advanced setting (or one line if no further lines of HER2 targeted therapy are available locally).*
10. A maximum of two prior lines of cytotoxic chemotherapy, chemotherapy antibody-drug conjugate or immunotherapy alone in the advanced setting is permitted.
11. Patient must either be suitable for a baseline biopsy of recurrent disease or have an archival biopsy of recurrent disease available. *Patients are requested to consent to a baseline biopsy but if deemed unsafe by the Investigator, an archival biopsy of recurrent disease can be used instead. If it is deemed unsafe to proceed with baseline biopsy, and no archival recurrent disease biopsy is available, the patient will not be eligible for entry into the treatment cohort.*
12. ECOG performance status ≤2.
13. Life expectancy >3 months.
14. EITHER postmenopausal, as defined by at least one of the following criteria:
    - Age >60 years;
    - Age <60 years and cessation of regular menses for at least 12 consecutive months with no alternative pathological or physiological cause; and serum estradiol and follicle stimulating hormone (FSH) level within the laboratory's reference range for postmenopausal females;
    - Documented bilateral oophorectomy.

    OR Pre-/peri-menopausal (i.e. not meeting the criteria for being postmenopausal) if being treated with an LHRH agonist that was commenced at least 4 weeks prior to Cycle 1 Day 1, and continues on the LHRH agonist throughout the trial period.

15. Patients must be a) surgically sterile; b) have a sterilised sole partner; or c) be postmenopausal; or d) must agree to practice true abstinence; or e) use effective contraception during the period of trial treatment and be willing to do so for 6 months following the end of trial treatment. *True abstinence must be in line with the preferred and usual lifestyle of the patient. (Periodic abstinence, such as calendar, ovulation, symptothermal, post-ovulation methods, and withdrawal are not acceptable methods of contraception). Effective contraception is defined as double barrier contraception (e.g. condom plus spermicide in combination with a diaphragm, cervical cap or intrauterine device). Ovarian suppression with an LHRH agonist is not a method of contraception.*
16. Patients of childbearing potential should have a negative serum or urine pregnancy test within 14 days prior to initiation of trial treatment.
17. At least 4 weeks washout period after the end of trial treatment on a different cohort within plasmaMATCH.
18. Adequate haematological, renal and hepatic function as defined by:
    - Haematology:
        - Absolute neutrophil count (ANC) ≥1000/mm3 (≥1.0 x 109/L)
        - Platelet count ≥100,000/mm3 (≥100 x 109/L)
        - Haemoglobin ≥9g/dL (≥90g/L)
    - Renal function:
        - Serum creatinine ≤1.5 x upper limit of normal (ULN)
        - Calculated creatinine clearance more than 30ml/min using the Cockcroft-Gault equation (please refer to Appendix 5)
    - Liver function tests:
        - Total bilirubin ≤1.5 ULN*
        - Alanine aminotransferase (ALT) ≤3 ULN. In the presence of liver metastases ALT ≤5 ULN

* Patients with known Gilbert's syndrome (normal conjugated bilirubin and otherwise normal liver function) are eligible, assuming conjugated bilirubin is within normal range.

A3.2. Exclusion Criteria

1. Prior treatment with radiotherapy (except for palliative reasons), endocrine therapy, immunotherapy, chemotherapy or IMPs during the previous 4 weeks (6 weeks for nitrosoureas, Mitomycin-C) before trial treatment, except for hormonal therapy with LHRH analogues, which are permitted, and bisphosphonates or RANK ligand antibodies that are permitted for the management of bone metastases.
2. Uncontrolled CNS disease (brain metastases or leptomeningeal disease). Patients with prior diagnosis of CNS metastases must be stable by clinical assessment having ceased steroids after prior treatment.
3. History of clinically significant or uncontrolled cardiac disease, including congestive heart failure, angina, myocardial infarction within the last 6 months or ventricular arrhythmia. *Patients with a history of any of the above listed cardiac conditions judged not to be clinically significant by the local Investigator must be notified to the trial team at the ICR-CTSU for approval by the CI and/or Cohort Lead.*

4. Ongoing toxic manifestations of previous treatments Grade ≥1. Exceptions to this are alopecia or toxicities which in the opinion of the Investigator should not exclude the patient. Such cases should be clearly documented in the patient's notes by the Investigator.
5. Major surgery (excluding minor procedures, e.g. placement of vascular access) within 4 weeks of the first dose of trial treatment.
6. Pregnant or breastfeeding.
7. Any condition that according to the treating physician may compromise the patient's safety or the conduct of the trial.
8. Current malignancies of other types, with the exception of adequately treated in situ carcinoma of the cervix and basal or squamous cell carcinoma of the skin. *Cancer survivors, who have undergone potentially curative therapy for a prior malignancy and have no evidence of the disease for 3 years or more are eligible for the trial.*
9. Known abnormalities in coagulation such as bleeding diathesis, or treatment with anticoagulants. *Low molecular weight heparin (LMWH), low dose aspirin and clopidogrel are permitted.*
10. Prior exposure to a SERD. Fulvestrant at 250mg or lower is allowed. Patients with prior treatment with fulvestrant at 500mg are excluded.
11. Unwilling to receive an IM injection.
12. Known hypersensitivity to fulvestrant and its excipients.

A4. COHORT A TRIAL ASSESSMENTS

The Cohort A Schedule of Assessments (Section A.4.8) shows all required trial assessments in table form. All blood and tissue samples should be collected according to the instructions provided in the plasmaMATCH Investigator Laboratory Manual.

A4.1. Cohort A Screening Assessments

The following assessments should be conducted following identification of an *ESR1* mutation in plasmaMATCH ctDNA screening required for entry into treatment Cohort A. Only those procedures required as part of standard patient care should be conducted prior to obtaining written informed consent from the patient for entry into the treatment cohort as detailed in Section 8.1.

The following assessments should be performed within 28 days prior to Cycle 1 Day 1:

- Medical history
- Physical examination and vital signs
- ECOG performance status
- Assessment of symptoms
- Review of concomitant medication
- Safety bloods: Haematology – full blood count, white cell count with differential and ANC, prothrombin time and international normalised ratio (INR); Biochemistry – sodium, potassium, calcium, magnesium, ALT, gamma-glutamyl transferase (GGT), bilirubin, albumin, creatinine, alkaline phosphatase, glucose, urea
- Bone scan
- CT and/or MRI scan to establish RECIST v1.1 baseline
- Mandatory baseline recurrent tumour biopsy and/or provision of archival recurrent disease biopsy where it is deemed unsafe by the Investigator to take a baseline biopsy from the patient
- Provision of archival primary tumour sample. If an archival primary tumour sample is not available but another previously obtained sample is available, this sample should be provided instead

The following assessments should be conducted within 14 days prior to Cycle 1 Day 1:

- Electrocardiogram (ECG)
- Calculated creatinine clearance (see Appendix 5 for creatinine clearance calculation)
- Pregnancy test and contraceptive counselling for patients of childbearing potential

A4.2. Cohort A Baseline Assessments Pre-Treatment Cycle 1 Day 1

The following assessments should be performed within 7 days (including Cycle 1 Day 1) prior to commencing trial treatment:

- Physical examination and vital signs
- Assessment of symptoms
- Review of concomitant medications
- Safety bloods: Haematology – full blood count, white cell count with differential and ANC, prothrombin time and INR; Biochemistry – sodium, potassium, calcium, magnesium, ALT, GGT, bilirubin, albumin, creatinine, alkaline phosphatase, glucose, urea

- Research blood samples:
  - 20ml blood sample collected in EDTA tube for plasma ctDNA analysis, to be processed within 1 hour at site
  - 10ml blood sample collected in serum tube for biomarker bloods
  - 3ml blood sample collected in EDTA tube for germline DNA

A4.3. Cohort A On-Treatment Assessments

A4.3.1. Cycle 1, Day 15 Pre-Treatment

- Physical examination (symptom directed) and vital signs
- Assessment of symptoms including clinical assessment of disease progression
- Dosing compliance
- Review of concomitant medications
- Review of AEs
- Safety bloods to be taken within 72 hours prior to treatment: Haematology – full blood count, white cell count with differential and ANC, prothrombin time and INR; Biochemistry – sodium, potassium, calcium, magnesium, ALT, GGT, bilirubin, albumin, creatinine, alkaline phosphatase, glucose, urea
- Research blood samples:
  - 20ml blood sample collected in EDTA tube for plasma ctDNA analysis, to be processed within 1 hour at site
  - 10ml blood sample collected in serum tube for biomarker bloods
- Research tissue sample:
  - Optional recurrent tumour biopsy (can be performed before or after trial treatment administration)

A4.3.2. Cycle 2, Day 1 Pre-Treatment

The following assessments should be performed as close to as possible and within -/+ 3 days of the due date of the visit:

- Physical examination and vital signs
- Assessment of symptoms including clinical assessment of disease progression
- Dosing compliance
- Review of concomitant medications
- Review of AEs
- Safety bloods to be taken within 72 hours prior to the start of cycle 2: Haematology – full blood count, white cell count with differential and ANC, prothrombin time and INR; Biochemistry – sodium, potassium, calcium, magnesium, ALT, GGT, bilirubin, albumin, creatinine, alkaline phosphatase, glucose, urea
- Research blood samples:
  - 20ml blood sample collected in EDTA tube for plasma ctDNA analysis, to be processed within 1 hour at site
  - 10ml blood sample collected in serum tube for biomarker bloods
  - 3ml blood sample collected in tube provided for PK analysis

A4.3.3. Cycle 3 Onwards, Day 1 Pre-Treatment

The following assessments should be performed as close to as possible and within -/+ 7 days of the due date of the visit:

- Physical examination and vital signs
- Assessment of symptoms including clinical assessment of disease progression
- Dosing compliance
- Review of concomitant medications
- Review of AEs
- Safety bloods to be taken within 72 hours prior to the start of each cycle: Haematology – full blood count, white cell count with differential and ANC, prothrombin time and INR; Biochemistry – sodium, potassium, calcium, magnesium, ALT, GGT, bilirubin, albumin, creatinine, alkaline phosphatase, glucose, urea
- CT and/or MRI scan every 8 weeks from Cycle 1 Day 1 until 32 weeks, then every 12 weeks thereafter
- RECIST v1.1 assessment every 8 weeks from Cycle 1 Day 1 until 32 weeks, then every 12 weeks thereafter
- Bone scan only if clinically indicated
- Research blood samples:
    - 20ml blood sample collected in EDTA tube for plasma ctDNA analysis, to be processed within 1 hour at site
    - 3ml blood sample collected in tubes provided for PK analysis on Cycle 3 Day 1 and Cycle 4 Day 1 only

A4.4. Cohort A End of Treatment Assessments

A4.4.1. Treatment Discontinuation Visit

The following assessments should be performed at the time of discontinuation of trial treatment for any reason:

- ECOG performance status
- Physical examination and vital signs
- Assessment of symptoms including clinical assessment of disease progression
- Dosing compliance
- Review of concomitant medications
- Review of AEs
- Safety bloods: Haematology – full blood count, white cell count with differential and ANC, prothrombin time and INR; Biochemistry – sodium, potassium, calcium, magnesium, ALT, GGT, bilirubin, albumin, creatinine, alkaline phosphatase, glucose, urea
- Research blood sample:
    - 20ml blood sample collected in EDTA tube for plasma ctDNA analysis, to be processed within 1 hour at site

A4.4.2. End of Treatment Visits 30 and 60 days after the Last Administration of Trial Treatment

- ECOG performance status
- Physical examination and vital signs
- Assessment of symptoms including clinical assessment of disease progression
- Review of concomitant medications
- Review of AEs
- Safety bloods: Haematology – full blood count, white cell count with differential and ANC, prothrombin time and INR; Biochemistry – sodium, potassium, calcium, magnesium, ALT, GGT, bilirubin, albumin, creatinine, alkaline phosphatase, glucose, urea
- Research tissue sample:
    - Optional recurrent tumour biopsy (Day 30 only)

A4.5. Cohort A Post-Treatment Follow up

All patients should be followed up at 6 monthly intervals from the end of trial treatment until death or up to 2 years; assessment should be in line with standard practice and should include:

- Survival
- Further treatment

A4.6. Cohort A Discontinuation from Treatment

Patients may discontinue from trial treatment at any time at their own request or they may be discontinued at the discretion of the PI. Protocol-specified reasons for discontinuation include:

- Disease progression or recurrence as defined by RECIST v1.1
- Unacceptable toxicity
- Pregnancy
- Withdrawal of consent
- Serious non-attendance and/or persistent non-compliance with procedures defined in the trial protocol

Patients who discontinue trial treatment in the absence of disease progression should continue to have CT and/or MRI scans to assess disease status according to the trial assessment schedule. All patients who discontinue treatment should continue to be followed up. Patients should be asked for consent for future linkage with routinely collected health data (via national registries) to trace their eventual vital status and assess subsequent unexpected co-morbidities.

A4.7. Cohort A Discontinuation from Follow up

If a patient wishes to withdraw from further follow up a Patient Withdrawal Form should be submitted to ICR-CTSU within the clinical trial database stating whether the patient has withdrawn consent for further information to be sent to the ICR-CTSU or whether they simply no longer wish to attend trial follow up visits.

In the very rare event that a patient requests that their data is removed from the trial entirely, the implications of this should be discussed with the patient first to ensure that this is their intent and, if

confirmed, ICR-CTSU should be notified in writing. *The patient should be made aware that any information about them that has already been published or submitted to the authorities for safety monitoring purposes cannot be withdrawn.*

Should a patient withdraw consent for their samples to be used in plasmaMATCH, following receipt of written confirmation from the site to ICR-CTSU, blood samples will be destroyed and biopsy blocks will be returned to the site for archiving where requested or otherwise destroyed.

A4.8. Cohort A Schedule of Assessments

**Procedures and assessments**	**Screening for entry into treatment cohort**		**Cycle 1 (Baseline and Week 2)**		**Cycle 2 (Week 4)**	**Cycle 3 (Week 8)**	**Cycle 4 onwards (Week 12, and 4 weekly thereafter until progression)**	**End of treatment visits**			**Follow up**
	**Day -28 to Day -1**	**Day -14 to Day -1**	**Day 1[1]**	**Day 15**	**Day 1[2]**	**Day 1[3]**	**Day 1[3]**	**Treatment discontinuation**	**Day 30 after last dose of drug**	**Day 60 after last dose of drug**	**6 monthly from end of treatment until death or up to 2 years**
Fulvestrant			X<br>Days 1, 8 and 15		X<br>Days 1 and 15	X<br>Days 1 and 15	X<br>Days 1 and 15				
Informed consent	X										
Medical history	X										
ECOG performance status	X							X	X	X	
Obtain archival primary tissue[4]	X										
Recurrent tumour biopsy[5,6]	X										
**PLEASE NOTE, ASSESSMENTS SHOULD BE COMPELTED PRE-TREATMENT UNLESS OTHERWISE STATED**											
Recurrent tumour biopsy (optional)[6]				X					X		
Physical examination and vital signs	X		X	X	X	X	X	X	X	X	
Assessment of symptoms (including clinical assessment of disease progression)	X		X	X	X	X	X	X	X	X	
Dosing compliance				X	X	X	X	X			
Concomitant medications	X		X		X	X	X	X	X	X	
Adverse events				X	X	X	X	X	X[7]	X[7]	

Procedures and assessments	Screening for entry into treatment cohort		Cycle 1 (Baseline and Week 2)		Cycle 2 (Week 4)	Cycle 3 (Week 8)	Cycle 4 onwards (Week 12, and 4 weekly thereafter until progression)	End of treatment visits			Follow up
	Day -28 to Day -1	Day -14 to Day -1	Day 1[1]	Day 15	Day 1[2]	Day 1[3]	Day 1[3]	Treatment discontinuation	Day 30 after last dose of drug	Day 60 after last dose of drug	6 monthly from end of treatment until death or up to 2 years
12 Lead ECG		X									
Pregnancy test[8]		X									
Safety bloods[9]	X		X[9]	X[9]	X[9]	X[9]	X[9]	X	X	X	
Calculated creatinine clearance		X									
Biomarker bloods			X	X	X						
Plasma for ctDNA			X	X	X	X	X	X			
Whole blood for germline DNA			X								
PK bloods					X	X	X Cycle 4 Day 1 only				
Bone scan	X					(X) if clinically indicated	(X) if clinically indicated				
CT and/or MRI scan	X					X	X 8 weekly from Cycle 1 Day 1 until 32 weeks then every 12 weeks[10] or if clinically indicated				

Continued overleaf

**Procedures and assessments**	**Screening for entry into treatment cohort**		**Cycle 1 (Baseline and Week 2)**		**Cycle 2 (Week 4)**	**Cycle 3 (Week 8)**	**Cycle 4 onwards (Week 12, and 4 weekly thereafter until progression)**	**End of treatment visits**			**Follow up**
	**Day -28 to Day -1**	**Day -14 to Day -1**	**Day 1[1]**	**Day 15**	**Day 1[2]**	**Day 1[3]**	**Day 1[3]**	**Treatment discontinuation**	**Day 30 after last dose of drug**	**Day 60 after last dose of drug**	**6 monthly from end of treatment until death or up to 2 years**
Disease progression assessment (according to RECIST v1.1)	X					X	X 8 weekly from Cycle 1 Day 1 until 32 weeks then every 12 weeks				
Survival and further treatment follow up											X

[1] Cycle 1 Day 1: Baseline assessments should be performed within 7 days (including Cycle 1 Day 1) prior to commencing trial treatment.

[2] Cycle 2 Day 1: Assessments should be performed as close to as possible and within -/+ 3 days of the due date of the visit; trial treatment should be administered as per the above schedule, however if not possible due to unavoidable circumstances treatment may be administered 1 day prior to or delayed for up to 3 days after the due date.

[3] Cycle 3 Day 1 onwards: Assessments should be performed as close to as possible and within -/+ 7 days of the due date of the visit; trial treatment should be administered as per the above schedule, however if not possible due to unavoidable circumstances treatment may be administered 1 day prior to or delayed for up to 7 days after the due date.

[4] Provision of archival primary tumour sample. If an archival primary tumour sample is not available but another previously obtained sample is available, this sample should be provided instead.

[5] Patients are requested to consent to a baseline recurrent disease biopsy, however if deemed unsafe by the Investigator, an archival biopsy of recurrent disease can be used instead.

[6] Recurrent tumour core biopsies can be ultrasound assisted or CT scan guided dependent on metastatic site and according to local practice.

[7] SAEs should be followed up until resolution.

[8] Patients of childbearing potential should have a negative serum or urine pregnancy test within 14 days prior to initiation of trial treatment.

[9] Safety bloods should be taken within 7 days prior to Cycle 1 Day 1 and then within 72 hours prior to Cycle 1 Day 15 and the start of each cycle from Cycle 2 onwards: Haematology – full blood count, white cell count with differential and ANC, prothrombin time and INR; Biochemistry – sodium, potassium, calcium, magnesium, ALT, GGT, bilirubin, albumin, creatinine, alkaline phosphatase, glucose, urea.

[10] Patients who experience treatment cycle delays should continue to have CT and/or MRI scans every 8 weeks from Cycle 1 Day 1 until 32 weeks, and then every 12 weeks thereafter.

Please note, all blood and tissue samples should be collected according to the instructions provided in the plasmaMATCH Investigator Laboratory Manual.

A5. COHORT A TRIAL TREATMENT

Fulvestrant is an IMP within plasmaMATCH Cohort A.

A5.1. Cohort A Dose and Schedule

Fulvestrant should be given 500mg intramuscularly on Cycle 1 Days 1, 8 and 15, and Cycle 2 onwards, Days 1 and 15. Each treatment cycle consists of 28 days. Sites should refer to the SmPC for fulvestrant for instructions for drug preparation and administration.

Fulvestrant 500mg should be administered intramuscularly into the buttocks slowly (1–2 minutes per injection) as two 5ml injections, one in each buttock. Drug preparation and administration should be performed at the site by a physician, registered nurse or other qualified healthcare provider.

Fulvestrant should not be administered if the platelet count is <50,000/mm^3 (<50 x 10^9/L).

Trial treatment should be administered as per the above schedule, however if this is not possible due to unavoidable circumstances (i.e. bank holidays) from Cycle 2 onwards treatment may be administered 1 day prior to or delayed for up to 3 days after the due date within Cycle 2, or 1 day prior to or delayed for up to 7 days after the due date within Cycle 3 onwards.

For further guidance on trial treatment delay due to toxicity please refer to Sections A5.7 and A5.8.

A5.2. Cohort A Prescription and Dispensing

Fulvestrant should be prescribed by the PI or delegated Co-investigator and dispensed by the hospital pharmacy from trial stock supplied specifically for use within the plasmaMATCH trial only.

A5.3. Cohort A Patient Cards

A small wallet sized patient card template will be provided by ICR-CTSU for completion by the participating site. Each card will state:

- The name of the participating site
- That the patient is participating in the plasmaMATCH trial
- That the patient is taking fulvestrant
- An emergency site contact number

Patients should be advised to keep their patient card in their possession at all times.

A5.4. Cohort A Duration of Treatment

Patients should remain on trial treatment until progression as defined by RECIST v1.1. Patients may withdraw from trial treatment early if they experience unacceptable toxicity or if the treating clinician believes further treatment is no longer appropriate or at the patient’s request.

A5.5. Cohort A Permitted Concomitant Therapy

All medication considered necessary for the patients' welfare and which is not expected to interfere with the evaluation of the trial drugs may be given at the discretion of the Investigator. All concomitant medications must be recorded in the patient's notes, as well as the appropriate section of the plasmaMATCH eCRF.

There are no known drug–drug interactions requiring dose adjustment with fulvestrant.

Bisphosphonates, RANK ligand antagonists and blood transfusions should be given as required at the discretion of the Investigator.

Palliative radiotherapy is acceptable if given for bony metastases providing these are not indicative of disease progression.

Patients already on low molecular weight heparin (LMWH) prior to trial entry and those who need to begin anti-coagulant therapy while receiving trial treatment may be treated with LMWH. The LMWH should be temporarily discontinued 12–24 hours prior to each fulvestrant injection and then resumed 12–24 hours later (depending on the particular LMWH used). There is an increased risk of haemorrhage in patients on LMWH and the Investigator should decide whether that risk is outweighed by the possible benefits of continued trial treatment. It is advised to apply direct pressure to the injection site in these patients.

A5.6. Cohort A Non-permissible Medications/Therapies

Investigational agents and other anticancer agents should not be given while the patient is on trial treatment (except for LHRH agonists which are permitted).

Anticoagulation is not permitted with the exception of LMWH (see Section A5.5).

A5.7. Cohort A Dose Modifications

Every effort should be made to administer fulvestrant at the planned dose and schedule. However, patients experiencing toxicities related to the trial treatment may have their dose modified as outlined in this section.

Fulvestrant should not be administered if the platelet count is <50,000/mm^3 (<50 x 10^9/L).

Patients having unacceptable AEs as a result of fulvestrant may dose reduce to the licensed schedule of 500mg intramuscularly on Day 1 only of each subsequent cycle. Each treatment cycle consists of 28 days. No further dose reductions are permitted. Patients who continue to have unacceptable toxicity attributed to fulvestrant at the licensed schedule should permanently discontinue treatment with fulvestrant.

Caution should be used in patients with a creatinine clearance less than 30ml/min as safety and efficacy have not been evaluated in this population.

Caution should be used with fulvestrant in patients with hepatic impairment, as clearance may be reduced. The use of fulvestrant has not been evaluated in patients with Child-Pugh C hepatic impairment.

A5.8. Cohort A Dose Interruptions

If at the time of the scheduled start of the next cycle a patient has unresolved toxicities Grade ≥3, the start of the next cycle should be delayed for up to 21 days until these toxicities have resolved to Grade ≤1 or returned to baseline.

If a patient remains off trial treatment for >3 weeks trial treatment should be permanently discontinued.

A5.9. Cohort A Missed Doses

If a dose of fulvestrant is missed trial treatment should be resumed at the next scheduled dose. Missed doses should not be made up.

A5.10. Cohort A Overdoses

There is no human experience of over dosage. Animal studies suggest that no effects other than those related directly or indirectly to anti-estrogenic activity were evident with higher doses of fulvestrant. If overdose occurs, this should be managed symptomatically. Please contact the ICR-CTSU for advice.

A5.11. Cohort A Discontinuation and Subsequent Therapy

Treatment should continue until disease progression, unacceptable toxicity or withdrawal of patient consent. Patients who discontinue trial treatment in the absence of disease progression should continue to have CT and/or MRI scans to assess disease status according to the trial assessment schedule, provided that the patient has not withdrawn their consent to further trial assessments.

A5.12. Cohort A Supply and Distribution of Fulvestrant

Fulvestrant is manufactured and provided free of charge by AstraZeneca to participating sites.

No drug will be distributed to participating sites unless ICR-CTSU is satisfied that the required approvals and agreements and initiation procedures are complete.

A5.13. Cohort A Formulation, Packaging, Storage Conditions and Labelling

Fulvestrant 50mg/ml solution will be supplied by AstraZeneca for use in the plasmaMATCH trial only. Complete information about fulvestrant formulation can be found in the SmPC for fulvestrant. Fulvestrant is supplied as two 5ml clear neutral glass (Type 1) barrels, each containing 250mg/5ml of fulvestrant solution for injection and fitted with a tamper evident closure. The syringes are presented in a tray with polystyrene plunger rod and safety needles (SafetyGlide™) for connection to the barrel. Fulvestrant should be stored at 2°C to 8°C (in a refrigerator) in the original packaging.

The drug distribution company is responsible for labelling fulvestrant in accordance with the MHRA approved plasmaMATCH label. Pharmacies may add their own hospital dispensing label to the trial drug but should not obscure the existing label on the drug packaging.

A5.14. Cohort A Pharmacy Responsibilities and Drug Accountability

Fulvestrant supplied for the plasmaMATCH trial must not be used outside the context of the plasmaMATCH protocol. Records must be kept of all deliveries, dispensing and destruction in accordance with the plasmaMATCH Pharmacy Guidance Notes. These records may be requested by ICR-CTSU during the trial to monitor supply and usage of stock. Account must be given of any discrepancies and certificates of delivery and destruction must be signed and dated.

A6. COHORT A ASSESSMENT OF TOLERABILITY OF FULVESTRANT

If 2 out of the first 6 patients treated with the starting dose of fulvestrant experience a dose limiting toxicity (DLT) within the first 2 cycles of treatment, the starting dose of fulvestrant will be declared non-tolerable. If the starting dose is declared non-tolerable then the dose of fulvestrant will be decreased to the licensed dose of fulvestrant 500mg IM on Cycle 1 Days 1 and 15, and Cycle 2 onwards on Day 1.

The tolerability of extended-dose fulvestrant will be assessed by the SRC after the first 3 evaluable patients have completed 2 cycles and after 6 patients have completed 2 cycles. A further review will be carried out by the SRC after the first 40 patients have completed 2 cycles (target sample size of Cohort A in protocol v1.2) to assess whether it is safe to extend recruitment to the revised sample size of 78 patients. An evaluable patient is one who has had at least 75% of fulvestrant injections.

A DLT is defined using NCI CTCAE v4.0. A DLT is defined as a highly probable or probable **drug-related** toxicity occurring during the first 2 cycles:

- Grade 3 or 4 neutropenia
- Febrile neutropenia (fever of unknown origin without clinically or microbiologically documented infection) with Grade 3 or 4 neutropenia (ANC <1.0 x 10^9/L and fever ≥38.5°C)
- Infection (documented clinically or microbiologically) with Grade 3 or 4 neutropenia (ANC <1.0 x 10^9/L)
- Grade 3 or 4 thrombocytopenia
- Grade 3 or 4 diarrhoea despite maximal medical intervention
- Any other toxicity that is greater than that at baseline, is clinically significant and/or unacceptable, does not respond to supportive care and results in a disruption of dosing schedule of more than 14 days
- Any event, including significant dose reductions or omissions, judged to be a DLT by the SRC
- Death (excluding death from disease progression)

Excluding:

- Grade 3 fatigue, unless there is an increase by at least two grades from baseline
- Alopecia of any grade

- Isolated laboratory changes of any grade without clinical sequelae or clinical significance

COHORT B: *HER2* MUTATION TREATED WITH NERATINIB PLUS FULVESTRANT IN ER POSITIVE BREAST CANCER OR NERATINIB ALONE IN ER NEGATIVE BREAST CANCER

B1. COHORT B BACKGROUND AND RATIONALE

Mutation of *HER2* occurs in approximately 2% of *HER2* non-amplified cancers and approximately 10% of trastuzumab-resistant *HER2* amplified breast cancers (8, 9). In breast cancers, the most common *HER2* mutations include missense substitutions and indels within the tyrosine kinase domain which result in increased cell signalling, oncogenic transformation, and enhanced tumour growth in preclinical models (28).

Neratinib, an irreversible *EGFR*, *ERBB2* and *ERBB4* tyrosine kinase inhibitor, has been demonstrated to potently inhibit growth of both *HER2* amplified and mutant breast tumours *in vitro* and *in vivo* (28, 29). In a phase II open-label trial of neratinib in women with HER2 positive MBC with an average of two prior chemotherapy regimens, neratinib resulted in a 26% (95% CI 16-39%) response rate in women with prior trastuzumab treatment (30). Neratinib has demonstrated improved disease-free survival in the adjuvant setting with *HER2* amplified breast cancers in the phase III ExteNET study (31).

Neratinib is also under investigation in several phase II studies in advanced breast cancer patients whose tumours harbour *HER2* mutations (NCT01953926 and NCT01670877). In an interim analysis from an ongoing phase II multi-histology ‘basket’ study, neratinib was demonstrated to have encouraging signs of clinical activity as a single agent or in combination with fulvestrant, a selective endocrine receptor degrader, in a cohort of *HER2* mutant HER2 non-amplified hormone receptor positive breast cancers (32). Case reports describing confirmed partial responses in HER2 non-amplified *HER2* mutant metastatic breast cancers with neratinib have also been recently reported (33, 34). This provides a strong rationale for investigation of neratinib for *ERBB2* (*HER2*) mutated breast cancers.

On the basis of first in human studies, neratinib 320mg was determined to be the maximum tolerated dose (35). In the phase II study (without prophylactic diarrhoea management, NCT00266877), the high frequency of diarrhoea and gastrointestinal toxicities resulted in dose reductions and therefore the recommended dose for this and the subsequent phase II and III solid tumour studies was 240mg, both for single agent and studies in which neratinib is co-administered with other therapeutic agents including fulvestrant (NCT01953926 and NCT01670877).

Cross-talk between the estrogen and HER2 receptors results in reduced ER signalling (36). HER2 downstream activation can lead to ER activation and endocrine therapy resistance, and conversely estrogen receptor signalling may limit the sensitivity of therapies targeting HER2. Blockade of HER2 signalling leads to increased transcriptional activity of ER-related genes (37, 38). Therapies aimed at inhibiting the ‘cross-talk’ between ER and HER2 using combinations of endocrine and HER2-directed therapies have been shown to improve anti-tumour efficacy compared to single agent treatments alone in preclinical studies (39, 40). For example in *HER2* amplified breast cancer xenograft models, the combination of neratinib plus fulvestrant resulted in significant inhibition of tumour growth compared to single agent treatments (Puma, data on file).

Clinical studies have explored the combination of fulvestrant with pan-ERBB tyrosine kinase inhibitors such as neratinib in the clinical setting. Lapatinib was combined with fulvestrant in a randomised phase III trial in patients with aromatase inhibitor resistant metastatic ER positive and

HER2 positive breast cancer (41). The combination of fulvestrant plus lapatinib exhibited an overall response rate of 20% compared to 9% in the fulvestrant plus placebo arm. The combination regimen did not appear to decrease the tolerability of either agent alone. In the phase III ExteNET study (31), in a pre-specified subgroup analysis in the hormone receptor positive cohort in which >93% received concurrent hormone therapy, an enhanced neratinib benefit in improving disease free survival was observed. In a phase II neratinib basket study (NCT01953926), at the time of writing three *HER2* mutant breast cancer patients had been enrolled and treated with neratinib plus fulvestrant for greater than 1 cycle (>28 days) of therapy (Puma, data on file, (32)). No new safety signals from this combination had been observed at the time of writing.

B2. KNOWN RISKS AND BENEFITS OF NERATINIB

In a phase II study of single agent neratinib in 136 patients, the most common toxicity noted was diarrhoea, experienced to some degree by nearly all patients. In light of this, prophylactic loperamide will be administered in Cohort B of the plasmaMATCH trial. Also prevalent in 26-40% of patients were vomiting, fatigue and nausea (30). Agents that inhibit ErbB2 are associated with cardiac toxicity (decreased left ventricular ejection fraction (LVEF)), therefore there is a possible risk of cardiotoxicity with use of neratinib and cardiac assessments (MUGA/echocardiogram (ECHO)) will be conducted in all patients receiving this agent. Of note, in the phase III ExteNET study, there were no recorded instances of Grade 3 or 4 cardiotoxicity (31).

B3. COHORT B SPECIFIC ELIGIBILITY CRITERIA

Patients will be considered eligible for entry into treatment Cohort B if they fulfil all eligibility criteria presented below.

B3.1. Inclusion Criteria

1. Somatic HER2 activating mutation identified in plasmaMATCH ctDNA screening. *If a patient has more than one intervening line of therapy between ctDNA screening and entry into the treatment cohort, the actionable mutation must be re-confirmed by repeat ctDNA screening prior to entry.*
2. Signed Informed Consent Form for Entry into Treatment Cohort B (ER positive or ER negative).
3. Female.
4. Aged ≥18 years old.
5. Histologically confirmed invasive breast carcinoma.
6. Metastatic or recurrent locally advanced breast cancer that is not suitable for treatment with radical or curative intent.
7. Radiological evidence of disease progression following most recent line of therapy.
8. Measurable disease by RECIST v1.1 assessed by CT and/or MRI. Patients with bone only disease may be eligible if they have a soft tissue component that is measurable by RECIST v1.1. Any measurable lesion(s) that have previously been irradiated must have documented progression since the radiotherapy to be assessable.
9. Patients must have completed at least one prior line of treatment (chemotherapy, endocrine therapy or targeted therapy) for advanced breast cancer and/or relapse within 12 months of completing (neo)adjuvant chemotherapy. *Patients with HER2 positive breast cancer must*

have been treated with at least two lines of HER2 targeted therapy in the advanced setting (or one line if no further lines of HER2 targeted therapy are available locally).

10. A maximum of two prior lines of cytotoxic chemotherapy, chemotherapy antibody-drug conjugate or immunotherapy alone in the advanced setting is permitted.
11. Patient must either be suitable for a baseline biopsy of recurrent disease or have an archival biopsy of recurrent disease available. *Patients are requested to consent to a baseline biopsy but if deemed unsafe by the Investigator, an archival biopsy of recurrent disease can be used instead. If it is deemed unsafe to proceed with baseline biopsy, and no archival recurrent disease biopsy is available, the patient will not be eligible for entry into the treatment cohort.*
12. ECOG performance status ≤2.
13. Patients must be able to swallow and retain oral medication.
14. Life expectancy >3 months.
15. If ER positive on most recent tumour biopsy (determined locally using appropriate standard criteria) the patient must be:

    EITHER postmenopausal, as defined by at least one of the following criteria:
    - Age >60 years;
    - Age <60 years and cessation of regular menses for at least 12 consecutive months with no alternative pathological or physiological cause; and serum estradiol and FSH level within the laboratory's reference range for postmenopausal females;
    - Documented bilateral oophorectomy.

    OR Pre/peri-menopausal (i.e. not meeting the criteria for being postmenopausal) if being treated with an LHRH agonist that was commenced at least 4 weeks prior to Cycle 1 Day 1, and continues on the LHRH agonist throughout the trial period.
16. Patients must be a) surgically sterile; b) have a sterilised sole partner; or c) be postmenopausal; or d) must agree to practice true abstinence; or e) use effective contraception during the period of trial treatment and be willing to do so for 6 months following the end of trial treatment. *True abstinence must be in line with the preferred and usual lifestyle of the patient. (Periodic abstinence, such as calendar, ovulation, symptothermal, post-ovulation methods, and withdrawal are not acceptable methods of contraception). Effective contraception is defined as double barrier contraception (e.g. condom plus spermicide in combination with a diaphragm, cervical cap or intrauterine device). Ovarian suppression with an LHRH agonist is not a method of contraception.*
17. Patients of childbearing potential should have a negative serum or urine pregnancy test within 14 days prior to initiation of trial treatment.
18. At least 4 weeks washout period after the end of trial treatment on a different cohort within plasmaMATCH.
19. Adequate haematological, renal and hepatic function as defined by:
    - Haematology:
        - ANC ≥1000/mm^3 (≥1.0 x 10^9/L)
        - Platelet count ≥100,000/mm^3 (≥100 x 10^9/L)
        - Haemoglobin ≥9g/dL (≥90g/L)
    - Renal function:
        - Serum creatinine ≤1.5 ULN
    - Liver function tests:
        - Total bilirubin ≤1.5 ULN*

- ALT and AST ≤3 ULN. In the presence of liver metastases, AST and ALT ≤5 ULN

20. Normal LVEF by an ECHO or MUGA assessed using local criteria.
21. Prior exposure to fulvestrant is allowed.

* Patients with known Gilbert's syndrome (normal conjugated bilirubin and otherwise normal liver function) are eligible, assuming conjugated bilirubin is within normal range.

B3.2. Exclusion Criteria

1. Prior treatment with radiotherapy (except for palliative reasons), endocrine therapy, immunotherapy, chemotherapy or IMPs during the previous 4 weeks (6 weeks for nitrosoureas, Mitomycin-C) before trial treatment, except for hormonal therapy with fulvestrant or LHRH analogues, which are permitted, and bisphosphonates or RANK ligand antibodies that are permitted for the management of bone metastases.
2. Uncontrolled CNS disease (brain metastases or leptomeningeal disease). Patients with prior diagnosis of CNS metastases must be stable by clinical assessment having ceased steroids after prior treatment.
3. History of clinically significant or uncontrolled cardiac disease, including congestive heart failure, angina, myocardial infarction within the last 6 months or ventricular arrhythmia. *Patients with a history of any of the above listed cardiac conditions judged not to be clinically significant by the local Investigator must be notified to the trial team at the ICR-CTSU for approval by the CI and/or Cohort Lead.*
4. Ongoing toxic manifestations of previous treatments Grade ≥1. Exceptions to this are alopecia or toxicities which in the opinion of the Investigator should not exclude the patient. Such cases should be clearly documented in the patient's notes by the Investigator.
5. Major surgery (excluding minor procedures, e.g. placement of vascular access) within 4 weeks of the first dose of trial treatment.
6. Pregnant or breastfeeding.
7. Any condition that according to the treating physician may compromise the patient's safety or the conduct of the trial.
8. Current malignancies of other types, with the exception of adequately treated in situ carcinoma of the cervix and basal or squamous cell carcinoma of the skin. *Cancer survivors, who have undergone potentially curative therapy for a prior malignancy and have no evidence of the disease for 3 years or more are eligible for the trial.*
9. Prior exposure to anthracyclines or mitoxantrone with cumulative exposure in excess of 360mg/m² for doxorubicin, 720mg/m² for epirubicin or 72mg/m² for mitoxantrone.
10. Prior exposure to other HER2 kinase inhibitors is not allowed.
11. QTc interval >470ms, or known history of congenital QT-prolongation or Torsade de Pointes.
12. If ER positive on most recent tumour biopsy, known abnormalities in coagulation such as bleeding diathesis, or treatment with anticoagulants. *LMWH, low dose aspirin and clopidogrel are permitted.*
13. If ER positive on most recent tumour biopsy, unwilling to receive an IM injection.
14. History of malabsorption syndrome, Grade 2 or higher diarrhoea or other gastrointestinal condition where diarrhoea is a prevalent symptom, or other condition that would interfere with enteral absorption. *For example active intestine inflammation (e.g. Crohn's disease or ulcerative colitis) requiring immunosuppressive therapy.*

15. If ER positive on most recent tumour biopsy, known hypersensitivity to neratinib, fulvestrant or their excipients.

B4. COHORT B TRIAL ASSESSMENTS

The Cohort B Schedule of Assessments (Section B4.8) shows all required trial assessments in table form. All blood and tissue samples should be collected according to the instructions provided in the plasmaMATCH Investigator Laboratory Manual.

B4.1. Cohort B Screening Assessments

The following assessments should be conducted following identification of a *HER2* mutation in plasmaMATCH ctDNA screening required for entry into treatment Cohort B. Only those procedures required as part of standard patient care should be conducted prior to obtaining written informed consent from the patient for entry into the treatment cohort as detailed in Section 8.1.

The following assessments should be performed within 28 days prior to Cycle 1 Day 1:

- Medical history
- Physical examination and vital signs
- ECOG performance status
- Assessment of symptoms
- Review of concomitant medication
- Safety bloods: Haematology – full blood count, white cell count with differential and ANC, prothrombin time and INR; Biochemistry – sodium, potassium, calcium, magnesium, ALT, AST, GGT, bilirubin, albumin, creatinine, alkaline phosphatase, glucose, urea
- Bone scan
- CT and/or MRI scan to establish RECIST v1.1 baseline
- ECHO or MUGA
- Mandatory baseline recurrent tumour biopsy and/or provision of archival recurrent disease biopsy where it is deemed unsafe by the Investigator to take a baseline biopsy from the patient
- Provision of archival primary tumour sample. If an archival primary tumour sample is not available but another previously obtained sample is available, this sample should be provided instead

The following assessments should be conducted within 14 days prior to Cycle 1 Day 1:

- ECG
- Pregnancy test and contraceptive counselling for patients of childbearing potential

B4.2. Cohort B Baseline Assessments Pre-Treatment Cycle 1 Day 1

The following assessments should be performed within 7 days (including Cycle 1 Day 1) prior to commencing trial treatment:

- Physical examination and vital signs
- Assessment of symptoms including diarrhoea. Patients should start diarrhoea prophylaxis (see Section B5.5.1)
- Review of concomitant medications

- Safety bloods to be taken within 7 days prior to commencing trial treatment: Haematology – full blood count, white cell count with differential and ANC, prothrombin time and INR; Biochemistry – sodium, potassium, calcium, magnesium, ALT, AST, GGT, bilirubin, albumin, creatinine, alkaline phosphatase, glucose
- Research blood samples:
  - 20ml blood sample collected in EDTA tube for plasma ctDNA analysis, to be processed within 1 hour at site
  - 10ml blood sample collected in serum tube for biomarker bloods
  - 3ml blood sample collected in EDTA tube for germline DNA
  - 3ml blood sample collected in tube provided for PK analysis

B4.3. Cohort B On-Treatment Assessments

B4.3.1. Cycle 1 Day 15 Pre-Treatment

- Physical examination (symptom directed) and vital signs
- Assessment of symptoms including clinical assessment of disease progression and diarrhoea
- Dosing compliance
- Review of concomitant medications
- Review of AEs
- Safety bloods to be taken within 72 hours prior to Cycle 1 Day 15: Haematology – full blood count, white cell count with differential and ANC, prothrombin time and INR; Biochemistry – sodium, potassium, calcium, magnesium, ALT, AST, GGT, bilirubin, albumin, creatinine, alkaline phosphatase, glucose, urea
- Research blood samples:
  - 20ml blood sample collected in EDTA tube for plasma ctDNA analysis, to be processed within 1 hour at site
  - 10ml blood sample collected in serum tube for biomarker bloods
- Research tissue sample:
  - Optional recurrent tumour biopsy (can be performed before or after trial treatment administration)

B4.3.2. Cycle 2, Day 1 Pre-Treatment

The following assessments should be performed as close to as possible and within -/+ 3 days of the due date of the visit:

- Physical examination and vital signs
- Assessment of symptoms including clinical assessment of disease progression and diarrhoea
- Dosing compliance
- Review of concomitant medications
- Review of AEs
- Safety bloods to be taken within 72 hours prior to the start of Cycle 2: Haematology – full blood count, white cell count with differential and ANC, prothrombin time and INR; Biochemistry – sodium, potassium, calcium, magnesium, ALT, AST, GGT, bilirubin, albumin, creatinine, alkaline phosphatase, glucose, urea
- ECG

- Research blood samples:
    - 20ml blood sample collected in EDTA tube for plasma ctDNA analysis, to be processed within 1 hour at site
    - 10ml blood sample collected in serum tube for biomarker bloods
    - 3ml blood sample collected in tube provided for PK analysis

B4.3.3. Cycle 3 Onwards, Day 1 Pre-Treatment

The following assessments should be performed as close to as possible and within -/+ 7 days of the due date of the visit:

- Physical examination and vital signs
- Assessment of symptoms including clinical assessment of disease progression and diarrhoea
- Dosing compliance
- Review of concomitant medications
- Review of AEs
- Safety bloods to be taken within 72 hours prior the start of each cycle: Haematology – full blood count, white cell count with differential and ANC, prothrombin time and INR; Biochemistry – sodium, potassium, calcium, magnesium, ALT, AST, GGT, bilirubin, albumin, creatinine, alkaline phosphatase, glucose, urea
- ECG at Cycle 3 then every 12 weeks thereafter
- ECHO or MUGA at Cycle 3 then every 12 weeks thereafter
- CT and/or MRI scan every 8 weeks from Cycle 1 Day 1 until 32 weeks, then every 12 weeks thereafter
- RECIST v1.1 assessment every 8 weeks from Cycle 1 Day 1 until 32 weeks, then every 12 weeks thereafter
- Bone scan only if clinically indicated
- Research blood samples:
    - 20ml blood sample collected in EDTA tube for plasma ctDNA analysis, to be processed within 1 hour at site
    - 3ml blood sample collected in tubes provided for PK analysis on Cycle 3 Day 1 and Cycle 4 Day 1 only

B4.4. Cohort B End of Treatment Assessments

B4.4.1. Treatment Discontinuation Visit

The following assessments should be performed at the time of discontinuation of trial treatment for any reason:

- ECOG performance status
- Physical examination and vital signs
- Assessment of symptoms including clinical assessment of disease progression and diarrhoea
- Dosing compliance
- Review of concomitant medications
- Review of AEs
- Safety bloods: Haematology – full blood count, white cell count with differential and ANC, prothrombin time and INR; Biochemistry – sodium, potassium, calcium, magnesium, ALT, AST, GGT, bilirubin, albumin, creatinine, alkaline phosphatase, glucose, urea

- ECG
- Repeat LVEF by ECHO or MUGA unless performed within the last 6 weeks
- Research blood sample:
    - 20ml blood sample collected in EDTA tube for plasma ctDNA analysis, to be processed within 1 hour at site

B4.4.2. End of Treatment Visit 30 days after the Last Administration of Trial Treatment

The following assessments should be performed at the end of trial treatment visit, 30 days after the last administration of trial treatment:

- ECOG performance status
- Physical examination and vital signs
- Assessment of symptoms including clinical assessment of disease progression and diarrhoea
- Review of concomitant medications
- Review of AEs
- Safety bloods: Haematology – full blood count, white cell count with differential and ANC, prothrombin time and INR; Biochemistry – sodium, potassium, calcium, magnesium, ALT, AST, GGT, bilirubin, albumin, creatinine, alkaline phosphatase, glucose, urea
- ECG
- Repeat LVEF by ECHO or MUGA unless performed within the last 6 weeks
- Research tissue sample:
    - Optional recurrent tumour biopsy

B4.5. Cohort B Post-Treatment Follow up

All patients should be followed up at 6 monthly intervals from the end of trial treatment until death or up to 2 years; assessment should be in line with standard practice and should include:

- Survival
- Further treatment

B4.6. Cohort B Discontinuation from Treatment

Participants may discontinue from trial treatment at any time at their own request, or they may be discontinued at the discretion of the PI. Protocol-specified reasons for discontinuation will include:

- Disease progression or recurrence
- Unacceptable toxicity
- Pregnancy
- Withdrawal of consent
- Serious non-attendance and/or persistent non-compliance with procedures defined in the trial protocol

Patients who discontinue trial treatment in the absence of disease progression should continue to have CT and/or MRI scans to assess disease status according to the trial assessment schedule. All patients who discontinue treatment should continue to be followed up. Patients will be asked for consent for future linkage with routinely collected health data (via national registries) to trace their eventual vital status and assess subsequent unexpected co-morbidities.

B4.7. Cohort B Discontinuation from Follow up

If a patient wishes to withdraw from further follow up, a Patient Withdrawal Form should be submitted to ICR-CTSU within the clinical trial database stating whether the patient has withdrawn consent for further information to be sent to the ICR-CTSU or whether they simply no longer wish to attend trial follow up visits.

In the very rare event that a patient requests that their data is removed from the trial entirely, the implications of this should be discussed with the patient first to ensure that this is their intent and, if confirmed, ICR-CTSU should be notified in writing. *The patient should be made aware that any information about them that has already been published or submitted to the authorities for safety monitoring purposes cannot be withdrawn.*

Should a patient withdraw consent for their samples to be used in plasmaMATCH, following receipt of written confirmation from the site to ICR-CTSU, blood samples will be destroyed and biopsy blocks returned to the site for archiving where requested or otherwise destroyed.

B4.8. Cohort B Schedule of Assessments

**Procedures and Assessments**	**Screening for entry into treatment cohort**		**Cycle 1 (Baseline and Week 2)**		**Cycle 2 (Week 4)**	**Cycle 3 (Week 8)**	**Cycle 4 onwards (Week 12, and 4 weekly thereafter until progression)**	**End of treatment visits**		**Follow up**
	**Day -28 to Day -1**	**Day -14 to Day -1**	**Day 1[1]**	**Day 15**	**Day 1[2]**	**Day 1[3]**	**Day 1[3]**	**Treatment discontinuation**	**Day 30 after last dose of drug**	**6 monthly from end of treatment until death or up to 2 years**
Neratinib			Neratinib taken continuously for each 28 day treatment cycle							
Patients with ER positive breast cancer only: Fulvestrant			X	X	X Day 1 only	X Day 1 only	X Day 1 only			
Informed consent	X									
Medical history	X									
ECOG performance status	X							X	X	
Obtain archival primary tissue[4]	X									
Recurrent tumour biopsy[5,6]	X									
**PLEASE NOTE, ASSESSMENTS SHOULD BE COMPELTED PRE-TREATMENT UNLESS OTHERWISE STATED**										
Recurrent tumour biopsy (optional)[6]				X					X	
Physical examination and vital signs	X		X	X	X	X	X	X	X	
Assessment of symptoms (including clinical assessment of disease progression)	X		X	X	X	X	X	X	X	
Assessment of diarrhoea			X	X	X	X	X	X	X	
Dosing compliance				X	X	X	X	X		
Concomitant medications	X		X	X	X	X	X	X	X	

Continued overleaf

**Procedures and Assessments**	**Screening for entry into treatment cohort**		**Cycle 1 (Baseline and Week 2)**		**Cycle 2 (Week 4)**	**Cycle 3 (Week 8)**	**Cycle 4 onwards (Week 12, and 4 weekly thereafter until progression)**	**End of treatment visits**		**Follow up**
	**Day -28 to Day -1**	**Day -14 to Day -1**	**Day 1[1]**	**Day 15**	**Day 1[2]**	**Day 1[3]**	**Day 1[3]**	**Treatment discontinuation**	**Day 30 after last dose of drug**	**6 monthly from end of treatment until death or up to 2 years**
Adverse events				X	X	X	X	X	X[7]	
12 Lead ECG		X			X	X Cycle 3 Day 1 then 12 weekly		X	X	
Pregnancy test[8]		X								
Safety bloods[9]	X		X[9]	X[9]	X[9]	X[9]	X[9]	X	X	
Biomarker bloods			X	X	X					
Plasma for ctDNA			X	X	X	X	X	X		
Whole blood for germline DNA			X							
PK bloods			X		X	X	X Cycle 4 Day 1 only			
Bone scan	X					(X) if clinically indicated	(X) if clinically indicated			
ECHO or MUGA	X					X Cycle 3 Day 1 then 12 weekly		X unless performed within last 6 weeks	X unless performed within last 6 weeks	
CT and/or MRI scan	X					X	X 8 weekly from Cycle 1 Day 1 until 32 weeks then every 12 weeks[10] or if clinically indicated			

Continued overleaf

**Procedures and Assessments**	**Screening for entry into treatment cohort**		**Cycle 1 (Baseline and Week 2)**		**Cycle 2 (Week 4)**	**Cycle 3 (Week 8)**	**Cycle 4 onwards (Week 12, and 4 weekly thereafter until progression)**	**End of treatment visits**		**Follow up**
	**Day -28 to Day -1**	**Day -14 to Day -1**	**Day 1[1]**	**Day 15**	**Day 1[2]**	**Day 1[3]**	**Day 1[3]**	**Treatment discontinuation**	**Day 30 after last dose of drug**	**6 monthly from end of treatment until death or up to 2 years**
Disease progression assessment (according to RECIST v1.1)	X					X	X 8 weekly from Cycle 1 Day 1 until 32 weeks then every 12 weeks			
Survival and further treatment follow up										X

[1] Cycle 1 Day 1: Baseline assessments (including safety bloods) should be performed within 7 days (including Cycle 1 Day 1) prior to commencing trial treatment.
[2] Cycle 2 Day 1: Assessments should be performed as close to as possible and within -/+ 3 days of the due date of the visit; trial treatment should be administered as per the above schedule, however if not possible due to unavoidable circumstances treatment may be administered 1 day prior to or delayed for up to 3 days after the due date.
[3] Cycle 3 Day 1 onwards: Assessments should be performed as close to as possible and within -/+ 7 days of the due date of the visit; trial treatment should be administered as per the above schedule, however if not possible due to unavoidable circumstances treatment may be administered 1 day prior to or delayed for up to 7 days after the due date.
[4] Provision of archival primary tumour sample. If an archival primary tumour sample is not available but another previously obtained sample is available, this sample should be provided instead.
[5] Patients are requested to consent to a baseline biopsy, however if deemed unsafe by the Investigator, an archival biopsy of recurrent disease can be used instead.
[6] Recurrent tumour core biopsies can be ultrasound assisted or CT scan guided dependent on metastatic site and according to local practice.
[7] SAEs should be followed up until resolution.
[8] Patients of childbearing potential should have a negative serum or urine pregnancy test within 14 days prior to initiation of trial treatment.
[9] Safety bloods should be taken within 7 days prior to Cycle 1 Day 1 and then within 72 hours prior to Cycle 1 Day 15 and the start of each cycle from Cycle 2 onwards: Haematology – full blood count, white cell count with differential and ANC, prothrombin time and INR; Biochemistry – sodium, potassium, calcium, magnesium, ALT, AST, GGT, bilirubin, albumin, creatinine, alkaline phosphatase, glucose, urea.
[10] Patients who experience treatment cycle delays should continue to have CT and/or MRI scans every 8 weeks from Cycle 1 Day 1 until 32 weeks, and then every 12 weeks thereafter.
Please note, all blood and tissue samples should be collected in accordance with the instructions provided in the plasmaMATCH Investigator Laboratory Manual.

B5. COHORT B TRIAL TREATMENT

Neratinib and fulvestrant are IMPs within plasmaMATCH Cohort B.

B5.1. Cohort B Dose and Schedule

B5.1.1. Patients with ER Positive Breast Cancer

Neratinib 240mg once daily should be administered orally. Neratinib tablets should be taken with food and must be swallowed intact. Trial treatment will be taken continuously for each 28 day treatment cycle.

Fulvestrant 500mg should be given intramuscularly on Cycle 1 Days 1 and 15, and Cycle 2 onwards, Day 1. Each treatment cycle consists of 28 days. Sites should refer to the SmPC for fulvestrant for instructions for drug preparation and administration.

Fulvestrant 500mg will be administered intramuscularly into the buttocks slowly (1–2 minutes per injection) as two 5ml injections, one in each buttock. Drug preparation and administration will be performed at the site by a physician, registered nurse or other qualified healthcare provider.

Fulvestrant should not be administered if the platelet count is <50,000/mm^3 (<50 x 10^9/L).

Trial treatment should be administered as per the above schedule, however if this is not possible due to unavoidable circumstances (i.e. bank holidays) from Cycle 2 onwards Day 1 of trial treatment may be administered 1 day prior to or delayed for up to 3 days after the due date for Cycle 2, or 1 day prior to or delayed for up to 7 days after the due date for Cycle 3 onwards.

For further guidance on trial treatment delay due to toxicity please refer to Sections B5.9 and B5.10.

B5.1.2. Patients with ER Negative Breast Cancer

Neratinib 240mg once daily should be administered orally and should be taken with food, and preferably in the morning. Trial treatment should be taken continuously for each 28 day treatment cycle.

B5.2. Cohort B Prescription and Dispensing

Neratinib tablets will be provided in non-patient-specific bottles. The patient's Trial ID should be recorded on the bottle label prior to dispensing. Patients should be instructed to keep their medication in the bottles provided and not transfer it to any other container.

All efforts should be made to ensure that patients clearly understand the directions for self-medication. Patients should be given a sufficient supply and unused drug and/or empty bottles should be returned at the appropriate time points. Returned unused medication must not be re-dispensed to any patient and should only be destroyed with prior approval from ICR-CTSU and according to local destruction policy.

Neratinib and fulvestrant should be prescribed by the PI or Co-investigator and dispensed by hospital pharmacy from trial stock supplied specifically for use within the plasmaMATCH trial only.

B5.3. Cohort B Patient Cards and Treatment Diary Cards

A small wallet sized patient card template will be provided by ICR-CTSU for completion by the participating site. Each card will state:

- The name of the participating site
- That the patient is participating in the plasmaMATCH trial
- That the patient is taking neratinib (if ER negative breast cancer) or neratinib and fulvestrant (if ER positive breast cancer)
- An emergency site contact number

Patients should be advised to keep their patient card in their possession at all times.

A treatment diary card will be provided by ICR-CTSU for completion by the patient, in order to record the number of tablets taken on each day of the treatment cycle.

B5.4. Cohort B Duration of Treatment

Patients will remain on trial treatment until progression. Patients may withdraw from trial treatment early if they experience unacceptable toxicity or if the treating clinician believes further treatment is no longer appropriate or at the patient's request.

B5.5. Cohort B Required Concomitant Treatment

B5.5.1. Diarrhoea prophylaxis

Diarrhoea is the major dose limiting toxicity of neratinib. Onset is usually in the first few weeks. Primary prophylactic use of anti-diarrhoeal medication is mandatory for all patients entered into Cohort B. Loperamide is recommended, and should be dispensed with neratinib on Cycle 1 Day 1.

Table B1. Cohort B diarrhoea prophylaxis – loperamide dosing

Cycle/Day	Dosing of loperamide
Cycle 1<br>Days 1–14	Loperamide 4mg self-administered orally 3 times a day (total 12mg a day). The initial dose of loperamide 4mg will be self-administered orally with the first dose of neratinib.
Cycle 1 Day<br>15–28	Loperamide 4mg self-administered orally twice a day (total 8mg a day).
Cycle 2 Day 1<br>onwards	Loperamide administered as needed (not to exceed 16mg per day). The goal for loperamide dosing is to titrate to 1–2 bowel movements a day.

B5.5.2. Loperamide Dose Adjustment

Patients are expected to take loperamide prophylaxis as directed. However, patients may require individualisation of loperamide prophylaxis dose (up to a maximum of 16mg per day) with the goal of titrating to 1–2 bowel movements a day. Patients with significant diarrhoea or unresponsive to medical treatment may need a dose interruption or reduction. Guidelines for loperamide dose modifications are detailed in Table B2 below, and separate guidelines for neratinib dose modifications are detailed in Section B5.9.2.

Table B2. Cohort B loperamide dose adjustment guidelines

<table>
<tr><th rowspan="2">Event</th><th colspan="2">Action</th></tr>
<tr><th>Cycles 1–2</th><th>Cycle 3 onwards</th></tr>
<tr><td>Grade 1 or 2 diarrhoea</td><td>For patients who develop diarrhoea during Cycles 1–2, loperamide should be increased to a maximum of 16mg a day.</td><td>Loperamide 4mg should be administered with the first bout of diarrhoea then 2mg every 4 hours or after every unformed stool (maximum 16mg per day) and continued until the patient is diarrhoea free for 12 hours.</td></tr>
<tr><td>Persistent Grade 1 diarrhoea</td><td colspan="2">For patients with persistent Grade 1 diarrhoea on loperamide, co-phenotrope 2.5mg/0.025mg tablets every 6 to 8 hours may be added. Octreotide and intraveous fluids should be administered as appropriate. Prophylactic antibiotics should be considered, and stool cultures taken at the Investigator's discretion.</td></tr>
<tr><td>Grade 3 or 4 diarrhoea</td><td colspan="2">Dietetic measures as above should be followed. Loperamide 4mg should be administered with the first bout of diarrhoea followed by 2mg every 4 hours or after every unformed stool (maximum 16mg per day) and continued until the patient is diarrhoea free for 12 hours.</td></tr>
<tr><td>Symptomatic constipation</td><td colspan="2">If a patient is unable to tolerate loperamide due to symptomatic constipation, loperamide should be held until after the first bowel movement and then resumed at a dose reduced by one level.

<table>
<tr><th>Dose level</th><th>Loperamide</th></tr>
<tr><td>Starting dose</td><td>4mg TID (6 tablets/capsules per day)</td></tr>
<tr><td>First dose reduction</td><td>4mg BID (4 tablets/capsules per day)</td></tr>
<tr><td>Second dose reduction</td><td>2mg TID (3 tablets/capsules per day)</td></tr>
<tr><td>Third dose reduction</td><td>2mg BID (2 tablets/capsules per day)</td></tr>
<tr><td>Fourth dose reduction</td><td>2mg OD (1 tablet/capsule per day)</td></tr>
</table>

For recurrent symptomatic constipation events, loperamide should be held until after the first bowel movement and then resumed at a dose reduced to the next lower dose level. If a patient is unable to tolerate once daily loperamide due to constipation, loperamide should be held. Sites should inform the ICR-CTSU who will discuss subsequent loperamide dosing with the Chief Investigator. Neratinib dosing should continue if loperamide is held.</td></tr>
</table>

B5.6. Cohort B Permitted Concomitant Therapy

All medication considered necessary for the patient's welfare and which is not expected to interfere with the evaluation of the trial drugs may be given at the discretion of the Investigator. All concomitant medications must be recorded in the patient's notes, as well as the appropriate section of the plasmaMATCH eCRF.

Bisphosphonates, RANK ligand antagonists, blood transfusions and secondary prophylactic use of growth factors should be given as required at the discretion of the Investigator.

Patients already on LMWH prior to trial entry and those who need to begin anti-coagulant therapy while receiving trial treatment may be treated with LMWH. For patients receiving neratinib and fulvestrant, the LMWH should be temporarily discontinued 12–24 hours prior to each fulvestrant injection and then resumed 12–24 hours later (depending on the particular LMWH used). There is an increased risk of haemorrhage in these patients and the Investigator should decide whether that risk is outweighed by the possible benefits of continued trial treatment. It is advised to apply direct pressure to the injection site in these patients.

Palliative radiotherapy is acceptable if given for bony metastases as long as these are not indicative of disease progression. Neratinib must be stopped 3 days before radiotherapy and restarted within 21 days.

B5.7. Cohort B Non-permissible Medications/Therapies

Investigational agents and other anticancer agents must not be given while the patient is on trial treatment.

For those patients on neratinib plus fulvestrant, anticoagulation is not permitted with the exception of LMWH (see Section B5.6)

B5.8. Cohort B Potential for Drug–Drug Interactions

Patients using drugs known to cause QT/QTc prolongation should be monitored closely with serial ECGs at the Investigator's discretion. Please refer to Appendix 6 for a summary of drugs known to have a risk of causing QT/QTc prolongation, potentially causing Torsades de Pointes.

Patients taking digoxin, a P-glycoprotein (P-gp) substrate with a narrow therapeutic window, should be monitored closely. The digoxin dose should be adjusted as needed, as neratinib is an inhibitor of P-gp. Administration of neratinib with digoxin could result in increased digoxin levels and associated digoxin toxicity. Please refer to Appendix 6 for a list of substrates and inhibitors of P-gp.

Cytochrome P450 (CYP) 3A4 inhibitors and inducers affect neratinib metabolism. Patients should avoid agents known to be strong CYP3A4 inducers or inhibitors. Please refer to Appendix 6 for further details.

Proton pump inhibitors, H2-receptor antagonists and antacids may lower the solubility of neratinib. Proton pump inhibitors should be avoided, if possible, and exchanged for either H2-receptor antagonists or antacids. If an H2-receptor antagonist such as ranitidine is required, neratinib should be taken 10 hours after the H2-receptor antagonist dosing and at least 2 hours before the next dose of the H2-receptor antagonist. If antacids are necessary, the antacid dose and the neratinib dose should be separated by 2 to 4 hours.

B5.9. Cohort B Dose Modifications

Every effort should be made to administer neratinib and fulvestrant at the planned dose and schedule. However, patients experiencing toxicities related to the trial treatment may have their dose modified as outlined in this section.

B5.9.1. Fulvestrant dose modifications

No dose reductions of fulvestrant are permitted within Cohort B. Patients who have unacceptable toxicity attributed to fulvestrant should permanently discontinue treatment with fulvestrant but should continue treatment with neratinib subject to the dose modification guidance provided in Section B5.9.2.

Fulvestrant should not be administered if the platelet count is $<50{,}000/mm^3$ ($<50 \times 10^9/L$).

Caution should be used in patients with a creatinine clearance less than 30ml/min as safety and efficacy have not been evaluated in this population.

Caution should be used with fulvestrant in patients with hepatic impairment, as clearance may be reduced. The use of fulvestrant has not been evaluated in patients with Child-Pugh C hepatic impairment.

B5.9.2. Neratinib Dose Modifications

A dose reduction of neratinib is recommended if the patient has a Grade 2 toxicity (not including alopecia) lasting >3 weeks despite appropriate supportive treatment or for any Grade ≥3 toxicity (based on NCI CTCAE v4.0). The following dose reductions should be applied to subsequent cycles unless a further dose reduction is required. Once a reduction is made the patient should not increase back to a higher dose level.

Table B3. Cohort B neratinib dose reduction guidelines

Dose level	Neratinib
Starting dose	240mg OD
First dose reduction	160mg OD
Second dose reduction	120mg OD
Third dose reduction	Discontinue trial treatment

Patients who have unacceptable toxicity attributed to neratinib should permanently discontinue neratinib. Patients with ER positive breast cancer should continue treatment with Fulvestrant according to the dose provided in B5.1.1.

B5.9.2.1. Specific Toxicities Requiring Neratinib Dose Modifications

Gastrointestinal Toxicity

Table B4. Cohort B diarrhoea management guidelines

Event	Action
• Grade 1 diarrhoea<br>OR<br>• Grade 2 lasting <5 days<br>OR<br>• Grade 3 lasting <2 days	• Adjust anti-diarrhoeal treatment, as per Section B5.5.2 at the first onset of diarrhoea<br>• Continue neratinib at full dose<br>• Instruct patient to follow dietetic recommendations – stop all lactose containing products, drink 8–10 glasses of water per day, and eat frequent small meals on a low fat regimen<br>• Fluid intake of ~2L should be maintained to avoid dehydration<br>• Once the event resolves to Grade ≤1 or baseline, start loperamide 4mg with each subsequent neratinib administration
• Persisting and intolerable Grade 2 diarrhoea lasting >5 days despite being treated with optimal medical therapy, or associated with fever, dehydration, or Grade 3–4 neutropenia<br>OR<br>• Grade 3 diarrhoea lasting >2 days despite being treated with optimal medical therapy, or associated with fever, dehydration, or Grade 3–4 neutropenia<br>OR<br>• Any Grade 4 diarrhoea	• Adjust anti-diarrhoeal treatment, as per Section B5.5.2 at the first onset of diarrhoea<br>• Hold neratinib until recovery to Grade ≤1 or baseline<br>• Instruct patient to follow dietetic recommendations – stop all lactose containing products, drink 8–10 glasses of water per day, and eat frequent small meals on a low fat regimen<br>• Fluid intake of ~2L should be maintained, intravenously if needed<br>• If recovery occurs:<br>o ≤1 week after withholding treatment, resume at the current dose level of neratinib<br>o Within 1-3 weeks after withholding treatment, reduce neratinib dose to the next lower dose level<br>• If event occurs a second time and the neratinib dose has not already been decreased, reduce neratinib dose to the next lower dose level<br>• A Grade 4 event should be reported as an SAE

Pulmonary toxicity

Patients should be monitored for acute onset of pulmonary symptoms and treated appropriately.

Table B5. Cohort B pneumonitis management guidelines

Event	Actions
• Grade 2 pneumonitis	• Hold neratinib until recovery to Grade ≤1 or baseline<br>• After recovery restart neratinib at the next lower dose level or discontinue at Investigator's discretion
• Grade 3 or 4 pneumonitis	• Discontinue neratinib permanently<br>• A Grade 4 event should be reported as an SAE

Hepatic toxicity

Table B6. Cohort B hepatic toxicity management guidelines

Event	Actions
• Grade 3 ALT/AST or Grade 3 bilirubin	• Hold neratinib until recovery to Grade ≤1, or Grade ≤2 in patients with Grade 2 ALT/AST at baseline<br>• Look for alternative causes<br>• Resume neratinib at the next lower dose level
• Grade 4 ALT/AST or Grade 4 bilirubin	• Permanently discontinue neratinib<br>• Evaluate alternative cause<br>• A Grade 4 event should be reported as an SAE
• ALT/AST >3 x ULN<br>AND<br>• Total bilirubin >2 x ULN<br>AND<br>• Alkaline phosphatase <2 x ULN	• Hold neratinib<br>• Evaluate the patient as soon as possible (within 48 hours if possible). All cases confirmed on repeat testing with no alternative explanation for abnormal liver function should be considered potential Hy's law cases<br>• If criteria for Hy's law are met, permanently discontinue treatment<br>• If criteria for Hy's law are not met, follow the guidance above for Grade 3/4 ALT/AST or bilirubin<br>• Report as SAE

LVEF toxicity

Table B7. Cohort B LVEF toxicity management guidelines

Event	Action
• Asymptomatic absolute decline of LVEF >15% from baseline<br>OR<br>• Absolute decline of LVEF >10% and below lower limit of normal	• **A. If LVEF below 40%:** Hold neratinib and seek cardiology input<br>Initiate monthly monitoring of LVEF:<br>o If while monitoring monthly LVEF remains <40%: reconsider neratinib at the next lower dose level only if appropriate and after cardiology consult<br>o If while monitoring monthly LVEF increases to ≥40%: continue neratinib at the next lower dose level, monitor LVEF every 12 weeks and consider cardiac support with input from cardiologist<br>• **B. If LVEF between 40% to 50%:** continue neratinib at the next lower dose level with caution and surveillance<br>Initiate monthly monitoring of LVEF:<br>o If while monitoring monthly LVEF falls to <40%: follow point A instructions above<br>o If while monitoring monthly LVEF remains ≥40%: continue neratinib, at the current dose level monitor LVEF every 12 weeks and consider cardiac support with input from cardiology<br>• ACE inhibitors should be considered according to local guidelines from other HER2 targeted therapies
• Symptomatic cardiac failure	• Discontinue neratinib and refer to cardiologist

B5.10. Cohort B Dose Interruptions

In addition to the specific toxicity management guidelines within Section B5.9, neratinib treatment should be interrupted if patients experience any Grade ≥3 toxicity.

Following an interruption for Grade ≥3 toxicity, trial treatment should be delayed for up to 21 days until these toxicities have resolved to Grade ≤1 or returned to baseline. Treatment with neratinib should be resumed at the next lower dose level. Please see Section B5.9.2.1 for guidance on restarting neratinib after stopping due to diarrhoea, pneumonitis or LVEF decline.

If a patient remains off trial treatment for >3 weeks trial treatment should be permanently discontinued.

B5.11. Cohort B Missed Doses

If a dose of neratinib or fulvestrant is missed trial treatment should be resumed at the next scheduled dose. Missed doses should not be made up. If the patient vomits after taking a dose of neratinib they should be advised to resume treatment at next scheduled dose.

B5.12. Cohort B Overdoses

No specific antidotes exist for the treatment of neratinib overdoses, and the benefit of haemodialysis in the treatment of neratinib overdose is unknown. General supportive care is recommended. There is no human experience of over dosage of fulvestrant. Animal studies suggest that no effects other than those related directly or indirectly to anti-estrogenic activity were evident with higher doses of fulvestrant. If overdose occurs, this should be managed symptomatically. Please contact the ICR-CTSU for advice.

B5.13. Cohort B Discontinuation and Subsequent Therapy

Treatment should continue until disease progression, unacceptable toxicity or withdrawal of patient consent. Patients who discontinue trial treatment in the absence of disease progression should continue to have CT and/or MRI scans to assess disease status according to the trial assessment schedule, provided that the patient has not withdrawn their consent to further trial assessments.

On disease progression, patients with more than one actionable mutation identified at ctDNA screening may participate in a second cohort according to the hierarchy of treatment cohorts specified in Section 3.2.3 after the defined washout period specified in the second cohort specific eligibility criteria and provided they meet all other cohort specific eligibility criteria at the time of entry into the second cohort.

B5.14. Cohort B Compliance

Patients should be asked to bring all their trial medication every time they attend the clinic for the purposes of treatment compliance assessment and drug accountability. Every effort should be made to encourage patients to return the unused medication and empty bottles. The unused tablets should be collected by the Investigator/Research Nurse and counted to ascertain patient compliance; medication will then be returned to pharmacy for drug accountability. Drug destruction should only be carried out with prior approval from ICR-CTSU and according to local destruction policy.

B5.15. Cohort B Supply and Distribution of Neratinib and Fulvestrant

Neratinib is manufactured and provided free of charge by Puma Biotechnology to participating sites.

Fulvestrant is manufactured and provided free of charge by AstraZeneca to participating sites.

No drug will be distributed to participating sites unless ICR-CTSU is satisfied that the required approvals and agreements and initiation procedures are complete.

B5.16. Cohort B Formulation, Packaging, Storage Conditions and Labelling

Neratinib will be supplied as film coated tablets packaged in bottles with desiccant. Neratinib should be stored at 25°C (77°F) or below with desiccant. Excursions are permitted to 30°C (86°F). Patients should be instructed to store neratinib in a safe place at room temperature.

Fulvestrant 50mg/ml solution will be supplied by AstraZeneca for use in the plasmaMATCH trial only. Complete information about fulvestrant formulation can be found in the SmPC for fulvestrant. Fulvestrant is supplied as two 5ml clear neutral glass (Type 1) barrels, each containing 250mg/5ml of fulvestrant solution for injection and fitted with a tamper evident closure. The syringes are presented in a tray with polystyrene plunger rod and safety needles (SafetyGlide™) for connection to the barrel. Fulvestrant should be stored at 2°C to 8°C (in a refrigerator) in the original packaging.
The drug distribution companies are responsible for labelling neratinib and fulvestrant in accordance with the MHRA approved plasmaMATCH labels. Pharmacies may add their own hospital dispensing label to the trial drug but should not obscure the existing label on the drug packaging.

B5.17. Cohort B Pharmacy Responsibilities and Drug Accountability

Neratinib and fulvestrant supplied for the plasmaMATCH trial must not be used outside the context of the plasmaMATCH protocol. Records must be kept of all deliveries, dispensing and destruction in accordance with the plasmaMATCH Pharmacy Guidance Notes. These records may be requested by ICR-CTSU during the trial to monitor supply and usage of stock. Account must be given of any discrepancies, and certificates of delivery and destruction must be signed and dated.

COHORT C: *AKT1* MUTATION TREATED WITH AZD5363 AND FULVESTRANT IN ER POSTIVE BREAST CANCER

C1. COHORT C BACKGROUND AND RATIONALE

AKT1 mutation occurs at an approximate prevalence of 3% in breast cancer (42). The *AKT1* E17K mutation results in constitutive activation of AKT1 (43), and is associated with sensitivity to catalytic kinase inhibitors *in vitro* such as AZD5363. In a phase I study with AZD5363 the single patient with *AKT1* E17K mutant ovarian cancer responded to AZD5363 (44).

AZD5363 is an oral, potent and selective ATP-competitive inhibitor of the serine/threonine kinase AKT with preclinical data that showed greatest potency in breast cancer cell lines (45). Increased AKT activity has been associated with endocrine resistance and so combined inhibition of the ER with fulvestrant and the AKT pathway with AZD5363 may be an effective strategy.

C2. KNOWN RISKS AND BENEFITS OF AZD5363 AND FULVESTRANT

As of 10 July 2014, 194 patients had been recruited into phase I and II studies of AZD5363. Recognised toxicities include diarrhoea, rash, hypersensitivity and hyperglycaemia (45). Therefore in Cohort C of the plasmaMATCH trial, glucose will be monitored pre-dose and at 2 and 4 hours post drug administration on Day 1 of each cycle. Asymptomatic increases in glucose have appeared to be mitigated by the use of metformin in the majority of patients. Hypersensitivity reactions have also been seen in four patients, three on monotherapy and one in combination with paclitaxel. A rash was seen in combination with one or more associated clinical features, such as pruritus, urticaria, throat itchiness, pyrexia, facial and/or lip oedema. All cases resolved with drug discontinuation and treatment with antihistamines and steroids.

FAKTION, a phase Ib/II randomised placebo controlled trial of fulvestrant +/- AZD5363 is an ongoing clinical study (CRUK reference CRUK/12/044; EudraCT reference 2013-000898-68; sponsored by Velindre NHS Trust, UK). FAKTION established a combination maximum tolerated dose for fulvestrant and AZD5363. Dosing fulvestrant at its licenced dose of 500mg IM on Cycle 1 Days 1 and 15, Cycle 2 onwards, Day 1 of a 28 day cycle, there were no dose limiting toxicities with AZD5363 given at 400mg BID po in the 4 days on, 3 days off schedule. The AE profile observed in the phase Ib part of FAKTION was consistent with the individual AE profiles of each drug and with no additional observations in relation to safety and tolerability. The randomised phase II component of FAKTION commenced in March 2015 using the 400mg BID 4 days on, 3 days off AZD5363 dose. This is the dose and schedule to be used in plasmaMATCH.

C3. COHORT C SPECIFIC ELIGIBILITY CRITERIA

Patients will be considered eligible for entry into treatment Cohort C if they fulfil all eligibility criteria presented below.

C3.1. Inclusion Criteria

1. *AKT1* mutation (any) identified in plasmaMATCH ctDNA screening. *If a patient has more than one intervening line of therapy between ctDNA screening and entry into the treatment cohort, the actionable mutation must be re-confirmed by repeat ctDNA screening prior to entry.*
2. ER positive on most recent tumour biopsy determined locally using appropriate standard criteria.

3. Signed Informed Consent Form for entry into Treatment Cohort C.
4. Female.
5. Aged ≥18 years old.
6. Histologically confirmed invasive breast carcinoma.
7. Metastatic or recurrent locally advanced breast cancer that is not suitable for treatment with radical or curative intent.
8. Radiological evidence of disease progression following most recent line of therapy.
9. Measurable disease by RECIST v1.1 assessed by CT and/or MRI. Patients with bone only disease may be eligible if they have a soft tissue component that is measurable by RECIST v1.1. Any measurable lesion(s) that have previously been irradiated must have documented progression since the radiotherapy to be assessable.
10. Patients must have completed at least one prior line of treatment (chemotherapy, endocrine therapy or targeted therapy) for advanced breast cancer and/or relapse within 12 months of completing (neo)adjuvant chemotherapy. *Patients with HER2 positive breast cancer must have been treated with at least two lines of HER2 targeted therapy in the advanced setting (or one line if no further lines of HER2 targeted therapy are available locally).*
11. A maximum of two prior lines of cytotoxic chemotherapy, chemotherapy antibody-drug conjugate or immunotherapy alone in the advanced setting is permitted.
12. Patient must either be suitable for a baseline biopsy of recurrent disease or have an archival biopsy of recurrent disease available. *Patients are requested to consent to a baseline biopsy but if deemed unsafe by the Investigator, an archival biopsy of recurrent disease can be used instead. If it is deemed unsafe to proceed with baseline biopsy, and no archival recurrent disease biopsy is available, the patient will not be eligible for entry into the treatment cohort.*
13. ECOG performance status ≤2.
14. Patients must be able to swallow and retain oral medication.
15. Life expectancy >3 months.
16. EITHER postmenopausal, as defined by at least one of the following criteria:
    - Age >60 years;
    - Age <60 years and cessation of regular menses for at least 12 consecutive months with no alternative pathological or physiological cause; and serum estradiol and FSH level within the laboratory's reference range for postmenopausal females;
    - Documented bilateral oophorectomy.

    OR Pre/peri-menopausal (i.e. not meeting the criteria for being postmenopausal) if being treated with an LHRH agonist that was commenced at least 4 weeks prior to Cycle 1 Day 1, and continues on the LHRH agonist throughout the trial period.
17. Patients must be surgically sterile or have a sterilised sole partner, be postmenopausal or must agree to practice true abstinence or use effective contraception during the period of trial treatment and be willing to do so for 6 months following the end of trial treatment. *True abstinence must be in line with the preferred and usual lifestyle of the patient. (Periodic abstinence, such as calendar, ovulation, symptothermal, post-ovulation methods, and withdrawal are not acceptable methods of contraception). Effective contraception is defined as double barrier contraception (e.g. condom plus spermicide in combination with a diaphragm, cervical cap or intrauterine device). Ovarian suppression with an LHRH agonist is not a method of contraception.*

18. Patients of childbearing potential should have a negative serum or urine pregnancy test within 14 days prior to initiation of trial treatment.
19. At least 4 weeks washout period after the end of trial treatment on a different cohort within plasmaMATCH.
20. Adequate haematological, renal and hepatic function as defined by:
    - Haematology:
        - ANC ≥1000/mm^3 (≥1.0 x 10^9/L)
        - Platelet count ≥100,000/mm^3 (≥100 x 10^9/L)
        - Haemoglobin ≥9g/dL (≥90g/L)
    - Renal function:
        - Serum creatinine ≤1.5 ULN
    - Liver function tests:
        - Total bilirubin ≤1.5 ULN*
        - ALT and AST ≤3 ULN. In the presence of liver metastases, AST and ALT ≤5 ULN
21. Normal LVEF by an ECHO or MUGA assessed using local criteria.
22. Prior exposure to fulvestrant and everolimus is allowed.

* Patients with known Gilbert's syndrome (normal conjugated bilirubin and otherwise normal liver function) are eligible, assuming conjugated bilirubin is within normal range.

C3.2. Exclusion Criteria

1. Prior treatment with radiotherapy (except for palliative reasons), endocrine therapy, immunotherapy, chemotherapy or IMPs during the previous 4 weeks (6 weeks for nitrosoureas, Mitomycin-C) before trial treatment, except for hormonal therapy with fulvestrant or LHRH analogues, which are permitted, and bisphosphonates or RANK ligand antibodies that are permitted for the management of bone metastases.
2. Uncontrolled CNS disease (brain metastases or leptomeningeal disease). Patients with prior diagnosis of CNS metastases must be stable by clinical assessment having ceased steroids after prior treatment.
3. History of clinically significant or uncontrolled cardiac disease, including congestive heart failure, angina, myocardial infarction within the last 6 months or ventricular arrhythmia. *Patients with a history of any of the above listed cardiac conditions judged not to be clinically significant by the local Investigator must be notified to the trial team at the ICR-CTSU for approval by the CI and/or Cohort Lead.*
4. Ongoing toxic manifestations of previous treatments Grade ≥1. Exceptions to this are alopecia or toxicities which in the opinion of the Investigator should not exclude the patient. Such cases should be clearly documented in the patient's notes by the Investigator.
5. Major surgery (excluding minor procedures, e.g. placement of vascular access) within 4 weeks of the first dose of trial treatment.
6. Pregnant or breastfeeding.
7. Any condition that according to the treating physician may compromise the patient's safety or the conduct of the trial.
8. Current malignancies of other types, with the exception of adequately treated in situ carcinoma of the cervix and basal or squamous cell carcinoma of the skin. *Cancer survivors,*

who have undergone potentially curative therapy for a prior malignancy and have no evidence of the disease for 3 years or more are eligible for the trial.

9. Glucose criteria: glycosylated haemoglobin (HbA1c) ≥8.0% (64mmol/mol) or fasting plasma glucose ≥7.0mmol/L (126mg/dL). Patients with well controlled diabetes mellitus may enter the trial unless any of the following exclusion criteria are fulfilled at baseline:
    - HbA1c >7.5% (58.5mmol/mol)
    - Baseline fasting glucose >8.4mmol/L or 152mg/dL (fasting is defined as no calorific intake for at least 8 hours)
    - Insulin required for routine diabetic management and control
    - More than 2 oral hypoglycaemic medications required for routine diabetic management and control
10. Known abnormalities in coagulation such as bleeding diathesis, or treatment with anticoagulants. *LMWH, low dose aspirin and clopidogrel are permitted.*
11. Prior exposure to agents with primary pharmacological activity of inhibition of AKT, including prior dosing of AZD5363.
12. Unwilling to receive an IM injection.
13. History of malabsorption syndrome or other condition that would interfere with enteral absorption of AZD5363. For example active intestine inflammation (e.g. Crohn's disease or ulcerative colitis) requiring immunosuppressive therapy.
14. Known hypersensitivity to fulvestrant and its excipients.
15. Patients requiring concurrent administration of CYP3A4 inhibitors and inducers.
16. Known hypersensitivity to AZD5363 and its excipients.

C4. COHORT C TRIAL ASSESSMENTS

The Cohort C Schedule of Assessments (Section C4.8) shows all required trial assessments in table form. All blood and tissue samples should be collected according to the instructions provided in the plasmaMATCH Investigator Laboratory Manual.

C4.1. Cohort C Screening Assessments

The following assessments should be conducted following identification of an *AKT1* mutation in plasmaMATCH ctDNA screening required for entry into treatment Cohort C. Only those procedures required as part of standard patient care should be conducted prior to obtaining written informed consent from the patient for entry into the treatment cohort as detailed in Section 8.1.

The following assessments should be performed within 28 days prior to Cycle 1 Day 1:

- Medical history
- Physical examination and vital signs
- ECOG performance status
- Assessment of symptoms
- Review of concomitant medication
- Safety bloods: Haematology – full blood count, white cell count with differential and ANC, prothrombin time and INR; Biochemistry – sodium, potassium, calcium, magnesium, ALT, AST, GGT, bilirubin, albumin, creatinine, alkaline phosphatase, urea
- HbA1c
- Fasting lipids (high-density lipoprotein (HDL), low-density lipoprotein (LDL), triglycerides, cholesterol)
- Bone scan
- CT and/or MRI scan to establish RECIST v1.1 baseline
- ECHO or MUGA
- Mandatory baseline recurrent tumour biopsy and/or provision of archival recurrent disease biopsy where it is deemed unsafe by the Investigator to take a baseline biopsy from the patient
- Provision of archival primary tumour sample. If an archival primary tumour sample is not available but another previously obtained sample is available, this sample should be provided instead

The following assessments should be conducted within 14 days prior to Cycle 1 Day 1:

- ECG
- Fasting plasma glucose
- Pregnancy test and contraceptive counselling for patients of childbearing potential

C4.2. Cohort C Baseline Assessments Pre-Treatment Cycle 1 Day 1

The following assessments should be performed within 7 days (including Cycle 1 Day 1) prior to commencing trial treatment:

- Physical examination and vital signs
- Assessment of symptoms
- Review of concomitant medications

- Safety bloods: Haematology – full blood count, white cell count with differential and ANC, prothrombin time and INR; Biochemistry – sodium, potassium, calcium, magnesium, ALT, AST, GGT, bilirubin, albumin, creatinine, alkaline phosphatase, urea
- Non-fasting glucose: pre-treatment **and** 2 and 4 hours post AZD5363. NB. The pre-treatment non-fasting glucose test must be done prior to dosing on the day of treatment.
- Research blood samples:
    - 20ml blood sample collected in EDTA tube for plasma ctDNA analysis, to be processed within 1 hour at site
    - 10ml blood sample collected in serum tube for biomarker bloods
    - 3ml blood sample collected in EDTA tube for germline DNA

C4.3. Cohort C On-Treatment Assessments

C4.3.1. Cycle 1, Day 15 Pre-Treatment

- Physical examination (symptom directed) and vital signs
- Assessment of symptoms including clinical assessment of disease progression
- Dosing compliance
- Review of concomitant medications
- Review of AEs
- Safety bloods to be taken within 72 hours prior to Cycle 1 Day 15: Haematology – full blood count, white cell count with differential and ANC, prothrombin time and INR; Biochemistry – sodium, potassium, calcium, magnesium, ALT, AST, GGT, bilirubin, albumin, creatinine, alkaline phosphatase, urea
- Non-fasting glucose: pre-dose **and** 2 and 4 hours post AZD5363. NB. The pre-treatment non-fasting glucose test must be done prior to dosing on the day of treatment.
- Research blood samples:
    - 20ml blood sample collected in EDTA tube for plasma ctDNA analysis, to be processed within 1 hour at site
    - 10ml blood sample collected in serum tube for biomarker bloods
- Research tissue sample:
    - Optional recurrent tumour biopsy (can be performed before or after trial treatment administration)

C4.3.2. Cycle 2, Day 1 Pre-Treatment

The following assessments should be performed as close to as possible and within -/+ 3 days of the due date of the visit:

- Physical examination and vital signs
- Assessment of symptoms including clinical assessment of disease progression
- Dosing compliance
- Review of concomitant medications
- Review of AEs
- Safety bloods to be taken within 72 hours prior to the start of Cycle 2: Haematology – full blood count, white cell count with differential and ANC, prothrombin time and INR; Biochemistry – sodium, potassium, calcium, magnesium, ALT, AST, GGT, bilirubin, albumin, creatinine, alkaline phosphatase, urea

- Non-fasting glucose: pre-dose **and** 2 and 4 hours post AZD5363. NB. The pre-treatment non-fasting glucose test must be done prior to dosing on the day of treatment.
- ECG
- Research blood samples:
  - 20ml blood sample collected in EDTA tube for plasma ctDNA analysis, to be processed within 1 hour at site
  - 10ml blood sample collected in serum tube for biomarker bloods

C4.3.3. Cycle 3 Onwards, Day 1 Pre-Treatment

The following assessments should be performed as close to as possible and within -/+ 7 days of the due date of the visit:

- Physical examination and vital signs
- Assessment of symptoms including clinical assessment of disease progression
- Dosing compliance
- Review of concomitant medications
- Review of AEs
- Safety bloods to be taken within 72 hours prior to the start of each cycle: Haematology – full blood count, white cell count with differential and ANC, prothrombin time and INR; Biochemistry – sodium, potassium, calcium, magnesium, ALT, AST, GGT, bilirubin, albumin, creatinine, alkaline phosphatase, urea
- Non-fasting glucose: pre-dose **and** 2 and 4 hours post AZD5363. NB. The pre-treatment non-fasting glucose test must be done prior to dosing on the day of treatment.
- ECG
- CT and/or MRI scan every 8 weeks from Cycle 1 Day 1 until 32 weeks, then every 12 weeks thereafter
- RECIST v1.1 assessment every 8 weeks from Cycle 1 Day 1 until 32 weeks, then every 12 weeks thereafter
- Bone scan only if clinically indicated
- Fasting lipids, HbA1c and fasting glucose at Cycle 4, then every 12 weeks thereafter
- Research blood samples:
  - 20ml blood sample collected in EDTA tube for plasma ctDNA analysis, to be processed within 1 hour at site

C4.4. Cohort C End of Treatment Assessments

C4.4.1. Treatment Discontinuation Visit

The following assessments should be performed at the time of discontinuation of trial treatment for any reason:

- ECOG performance status
- Physical examination and vital signs
- Assessment of symptoms including clinical assessment of disease progression
- Dosing compliance
- Review of concomitant medications
- Review of AEs

- Safety bloods: Haematology – full blood count, white cell count with differential and ANC, prothrombin time and INR; Biochemistry – sodium, potassium, calcium, magnesium, ALT, AST, GGT, bilirubin, albumin, creatinine, alkaline phosphatase, urea
- Non-fasting glucose
- ECG
- Research blood sample:
    - 20ml blood sample collected in EDTA tube for plasma ctDNA analysis, to be processed within 1 hour at site

C4.4.2. End of Treatment Visit 30 days after the last Administration of Trial Treatment

The following assessments should be performed at the end of trial treatment visit, 30 days after the last administration of trial treatment:

- ECOG performance status
- Physical examination and vital signs
- Assessment of symptoms including clinical assessment of disease progression
- Review of concomitant medications
- Review of AEs
- Safety bloods: Haematology – full blood count, white cell count with differential and ANC, prothrombin time and INR; Biochemistry – sodium, potassium, calcium, magnesium, ALT, AST, GGT, bilirubin, albumin, creatinine, alkaline phosphatase, urea
- Non-fasting glucose
- ECG
- Research tissue sample:
    - Optional recurrent tumour biopsy

C4.5. Cohort C Post-treatment Follow up

All patients should be followed up at 6 monthly intervals from the end of trial treatment until death or up to 2 years; assessment should be in line with standard practice and should include:

- Survival
- Further treatment

C4.6. Cohort C Discontinuation from Treatment

Patients may discontinue from trial treatment at any time at their own request, or they may be discontinued at the discretion of the PI. Protocol-specified reasons for discontinuation will include:

- Disease progression or recurrence
- Unacceptable toxicity
- Pregnancy
- Withdrawal of consent
- Serious non-attendance and/or persistent non-compliance with procedures defined in the trial protocol

Patients who discontinue trial treatment in the absence of disease progression should continue to have CT and/or MRI scans to assess disease status according to the trial assessment schedule. All patients who discontinue treatment should continue to be followed up. Patients will be asked for

consent for future linkage with routinely collected health data (via national registries) to trace their eventual vital status and assess subsequent unexpected co-morbidities.

C4.7. Cohort C Discontinuation from Follow up

If a patient wishes to withdraw from further follow up, a Patient Withdrawal Form should be submitted to ICR-CTSU within the clinical trial database stating whether the patient has withdrawn consent for further information to be sent to the ICR-CTSU or whether they simply no longer wish to attend trial follow up visits.

In the very rare event that a patient requests that their data is removed from the trial entirely, the implications of this should be discussed with the patient first to ensure that this is their intent and, if confirmed, ICR-CTSU should be notified in writing. *The patient should be made aware that any information about them that has already been published or submitted to the authorities for safety monitoring purposes cannot be withdrawn.*

Should a patient withdraw consent for their samples to be used in plasmaMATCH, following receipt of written confirmation from the site to ICR-CTSU, blood samples will be destroyed and biopsy blocks returned to the site for archiving where requested or otherwise destroyed.

C4.8. Cohort C Schedule of Assessments

Procedures and assessments	Screening for entry into treatment cohort		Cycle 1 (Baseline and Week 2)		Cycle 2 (Week 4)	Cycle 3 (Week 8)	Cycle 4 onwards (Week 12, and 4 weekly thereafter until progression)	End of treatment visits		Follow up
	Day -28 to Day -1	Day -14 to Day -1	Day 1[1]	Day 15	Day 1[2]	Day 1[3]	Day 1[3]	Treatment discontinuation	Day 30 after last dose of drug	6 monthly from end of treatment until death or up to 2 years
AZD5363			AZD5363 taken on a 4 days on – 3 days off schedule continuously for each 28 day treatment cycle							
Fulvestrant			X	X	X Day 1 only	X Day 1 only	X Day 1 only			
Informed consent	X									
Medical history	X									
ECOG performance status	X							X	X	
Obtain archival primary tissue[4]	X									
Recurrent tumour biopsy[5,6]	X									
**PLEASE NOTE, ASSESSMENTS SHOULD BE COMPELTED PRE-TREATMENT UNLESS OTHERWISE STATED**										
Recurrent tumour biopsy (optional)[6]				X					X	
Physical examination and vital signs	X		X	X	X	X	X	X	X	
Assessment of symptoms (including clinical assessment of disease progression)	X		X	X	X	X	X	X	X	
Dosing compliance				X	X	X	X	X		
Concomitant medications	X		X	X	X	X	X	X	X	
Adverse events				X	X	X	X	X	X[7]	
12 Lead ECG		X			X	X	X	X	X	

Continued overleaf

**Procedures and assessments**	**Screening for entry into treatment cohort**		**Cycle 1 (Baseline and Week 2)**		**Cycle 2 (Week 4)**	**Cycle 3 (Week 8)**	**Cycle 4 onwards (Week 12, and 4 weekly thereafter until progression)**	**End of treatment visits**		**Follow up**
	**Day -28 to Day -1**	**Day -14 to Day -1**	**Day 1[1]**	**Day 15**	**Day 1[2]**	**Day 1[3]**	**Day 1[3]**	**Treatment discontinuation**	**Day 30 after last dose of drug**	**6 monthly from end of treatment until death or up to 2 years**
Pregnancy test[8]		X								
HbA1c and fasting[9] lipid profile[10]	X						X Cycle 4 then 12 weekly			
Fasting[9] glucose		X					X Cycle 4 then 12 weekly			
Non-fasting glucose[11]			X pre-dose[11], 2 & 4 hours post-dose	X pre-dose[11], 2 & 4 hours post-dose	X pre-dose[11], 2 & 4 hours post-dose	X pre-dose[11], 2 & 4 hours post-dose	X pre-dose[11], 2 & 4 hours post-dose	X	X	
Safety bloods[12]	X		X[12]	X[12]	X[12]	X[12]	X[12]	X	X	
Biomarker bloods			X	X	X					
Plasma for ctDNA			X	X	X	X	X	X		
Whole blood for germline DNA			X							
Bone scan	X					(X) if clinically indicated	(X) if clinically indicated			
ECHO or MUGA	X									
CT and/or MRI scan	X					X	X 8 weekly from Cycle 1 Day 1 until 32 weeks then every 12 weeks[13] or if clinically indicated			

Continued overleaf

**Procedures and assessments**	**Screening for entry into treatment cohort**		**Cycle 1 (Baseline and Week 2)**		**Cycle 2 (Week 4)**	**Cycle 3 (Week 8)**	**Cycle 4 onwards (Week 12, and 4 weekly thereafter until progression)**	**End of treatment visits**		**Follow up**
	**Day -28 to Day -1**	**Day -14 to Day -1**	**Day 1[1]**	**Day 15**	**Day 1[2]**	**Day 1[3]**	**Day 1[3]**	**Treatment discontinuation**	**Day 30 after last dose of drug**	**6 monthly from end of treatment until death or up to 2 years**
Disease progression assessment (according to RECIST v1.1)	X					X	X 8 weekly from Cycle 1 Day 1 until 32 weeks then every 12 weeks			
Survival and further treatment follow up										X

[1] Cycle 1 Day 1: Baseline assessments should be performed within 7 days (including Cycle 1 Day 1) prior to commencing trial treatment.
[2] Cycle 2 Day 1: Assessments should be performed as close to as possible and within -/+ 3 days of the due date of the visit; trial treatment should be administered as per the above schedule, however if not possible due to unavoidable circumstances treatment may be administered 1 day prior to or delayed for up to 3 days after the due date.
[3] Cycle 3 Day 1 onwards: Assessments should be performed as close to as possible and within -/+ 7 days of the due date of the visit; trial treatment should be administered as per the above schedule, however if not possible due to unavoidable circumstances treatment may be administered 1 day prior to or delayed for up to 7 days after the due date.
[4] Provision of archival primary tumour sample. If an archival primary tumour sample is not available but another previously obtained sample is available, this sample should be provided instead.
[5] Patients are requested to consent to a baseline biopsy, however if deemed unsafe by the Investigator, an archival biopsy of recurrent disease can be used instead.
[6] Recurrent tumour core biopsies can be ultrasound assisted or CT scan guided dependent on metastatic site and according to local practice.
[7] SAEs should be followed up until resolution.
[8] Patients of childbearing potential should have a negative serum or urine pregnancy test within 14 days prior to initiation of trial treatment.
[9] For the purposes of the fasting lipid profile and fasting glucose assessment fasting is defined as no calorific intake for at least 8 hours.
[10] Triglycerides, HDL, LDL, cholesterol.
[11] The pre-treatment non-fasting glucose test must be done prior to dosing on the day of treatment.
[12] Safety bloods should be taken within 7 days prior to Cycle 1 Day 1 and then within 72 hours prior to Cycle 1 Day 15 and the start of each cycle from Cycle 2 onwards: Haematology – full blood count, white cell count with differential and ANC, prothrombin time and INR; Biochemistry – sodium, potassium, calcium, magnesium, ALT, AST, GGT, bilirubin, albumin, creatinine, alkaline phosphatase, urea.
[13] Patients who experience treatment cycle delays should continue to have CT and/or MRI scans every 8 weeks from Cycle 1 Day 1 until 32 weeks, and then every 12 weeks thereafter.
Please note, all blood and tissue samples should be collected according to the instructions provided in the plasmaMATCH Investigator Laboratory Manual.

C5. COHORT C TRIAL TREATMENT

AZD5363 and fulvestrant are IMPs within plasmaMATCH Cohort C.

C5.1. Cohort C Dose and Schedule

AZD5363 400mg BID should be administered orally on a 4 days on – 3 days off schedule. Trial treatment cycles are defined in 28 day periods. Patients must fast for 2 hours before and 1 hour after taking medication and AZD5363 tablets must be swallowed intact.

Fulvestrant 500mg should be given intramuscularly on Cycle 1 Days 1 and 15, Cycle 2 onwards, Day 1. Each treatment cycle consists of 28 days. Sites should refer to the SmPC for fulvestrant for instructions for drug preparation and administration.

Fulvestrant 500mg will be administered intramuscularly into the buttocks slowly (1–2 minutes per injection) as two 5ml injections, one in each buttock. Drug preparation and administration will be performed at the site by a physician, registered nurse or other qualified healthcare provider.

Fulvestrant should not be administered if the platelet count is $<50{,}000/mm^3$ ($<50 \times 10^9/L$).

Trial treatment should be administered as per the above schedule, however if this is not possible due to unavoidable circumstances (i.e. bank holidays) from Cycle 2 onwards Day 1 of trial treatment may be delayed for up to 3 days after the due date for Cycle 2, or delayed for up to 7 days after the due date for Cycle 3 onwards.

For further guidance on trial treatment delay due to toxicity please refer to Sections C5.7 and C5.8.

Table C1. Cohort C treatment schedule

	Cycle 1				Cycle 2 onwards			
	Week 1	Week 2	Week 3	Week 4	Week 1	Week 2	Week 3	Week 4
Fulvestrant	Day 1		Day 1		Day 1			
AZD5363	Days 1–4	Days 1–4	Days 1–4	Days 1–4	Days 1–4	Days 1–4	Days 1–4	Days 1–4

C5.2. Cohort C Prescription and Dispensing

AZD5363 will be provided in non-patient-specific bottles. The patient's Trial ID should be recorded on the bottle label prior to dispensing. Patients should be instructed to keep their medication in the bottles provided and not transfer it to any other container.

All efforts should be made to ensure that patients clearly understand the directions for self-medication. Patients should be given a sufficient supply and unused drug and/or empty bottles should be returned at the appropriate time points. Returned unused medication must not be re-dispensed to any patient and should only be destroyed with prior approval from ICR-CTSU according to local destruction policy.

Fulvestrant and AZD5363 should be prescribed by the PI or Co-investigator and dispensed by the hospital pharmacy from trial stock supplied specifically for use within plasmaMATCH only.

C5.3. Cohort C Patient Cards and Treatment Diary Cards

A small wallet sized patient card template will be provided by ICR-CTSU for completion by the participating site. Each card will state:

- The name of the participating site
- That the patient is participating in the plasmaMATCH trial
- That the patient is taking AZD5363 and fulvestrant
- An emergency site contact number

Patients should be advised to keep their patient card in their possession at all times.

A treatment diary card will be provided by ICR-CTSU for completion by the patient, in order to record the number of tablets taken on each day of the treatment cycle.

C5.4. Cohort C Duration of Treatment

Patients will remain on treatment until progression as defined by RECIST v1.1. Patients may withdraw from trial treatment early if they experience unacceptable toxicity or if the treating clinician believes further treatment is no longer appropriate or at the patient's request.

C5.5. Cohort C Permitted Concomitant Therapy

All medication considered necessary for the patients' welfare and which is not expected to interfere with the evaluation of the trial drugs may be given at the discretion of the Investigator. All concomitant medications must be recorded in the patient's notes, as well as the appropriate section of the plasmaMATCH eCRF.

Bisphosphonates, RANK ligand antagonists and blood transfusions should be given as required at the discretion of the Investigator. Patients may take corticosteroids but increased vigilance is recommended on electrolyte and glucose levels.

Palliative radiotherapy is acceptable if given for bony metastases as long as these are not indicative of disease progression. AZD5363 must be stopped 3 days before radiotherapy and restarted within 21 days.

Patients already on LMWH prior to trial entry and those who need to begin anti-coagulant therapy while receiving trial treatment may be treated with LMWH. The LMWH should be temporarily discontinued 12–24 hours prior to each fulvestrant injection and then resumed 12–24 hours later (depending on the particular LMWH used). There is an increased risk of haemorrhage in these patients and the Investigator should decide whether that risk is outweighed by the possible benefits of continued trial treatment. It is advised to apply direct pressure to the injection site in these patients.

C5.6. Cohort C Non-permissible Medications/Therapies

Investigational agents and other anticancer agents must not be given while the patient is on trial treatment.

Inhibitors or inducers of CYP3A4, or substrates of CYP3A4, CYP2B6, CYP2C9, CYP2C19 and CYP2D6, may affect the metabolism of AZD5363. Please see Appendix 7 for further details.

Anticoagulation is not permitted with the exception of LMWH (see Section C5.5).

C5.7. Cohort C Dose Modifications

Every effort should be made to administer AZD5363 and fulvestrant at the planned dose and schedule. However, patients experiencing toxicities related to the trial treatment may have their dose modified as outlined in this section.

C5.7.1. Fulvestrant Dose Modifications

No dose reductions of fulvestrant are permitted. Patients who have unacceptable toxicity attributed to fulvestrant should permanently discontinue fulvestrant but should continue treatment with AZD5363 subject to the dose modification guidance provided in Section C5.7.2.

Fulvestrant should not be administered if the platelet count is <50,000/mm^3 (<50 x 10^9/L).

Caution should be used in patients with a creatinine clearance less than 30ml/min as safety and efficacy have not been evaluated in this population.

Caution should be used with fulvestrant in patients with hepatic impairment, as clearance may be reduced. The use of fulvestrant has not been evaluated in patients with Child-Pugh C hepatic impairment.

C5.7.2. AZD5363 Dose Modifications

A dose reduction of AZD5363 is recommended if the patient has a Grade 2 toxicity (not including alopecia) lasting >3 weeks despite appropriate supportive treatment or for any Grade ≥3 toxicity (based on NCI CTCAE v4.0). The following dose reductions should be applied to subsequent cycles unless a further dose reduction is required. Once a reduction is made the patient should not increase back to a higher dose level.

Table C2. Cohort C AZD5363 dose reduction guidelines

Dose level	AZD5363
Starting dose	400mg BID, 4 days on – 3 days off
First dose reduction	320mg BID, 4 days on – 3 days off
Second dose reduction	200mg BID, 4 days on – 3 days off
Third dose reduction	Discontinue trial treatment

Patients who have unacceptable toxicity attributed to AZD5363 should permanently discontinue AZD5363 but should continue treatment with Fulvestrant according to the dose provided in C5.1.

C5.7.2.1. Specific Toxicities Requiring AZD5363 Dose Modifications

Hyperglycaemia

Table C3. Cohort C hyperglycaemia management guidelines

Event	Action
Grade ≤1 hyperglycaemia Glucose <8.9mmol/L	• Continue AZD5363 at the current dose level
Grade 2 hyperglycaemia Glucose ≥8.9–13.9mmol/L	• Continue AZD5363 at the current dose level, monitor glucose (as per local guidelines)
Grade 3 hyperglycaemia Glucose >13.9–27.8mmol/L	• If asymptomatic:<br>o Stop AZD5363 (for up to 21 days)<br>o Hold until blood glucose 13.9mmol/L or lower (recovery to Grade ≤2)<br>o Add oral metformin on the days when AZD5363 is re-administered at the current dose level.<br>o Dose reduce AZD5363 to the next lower dose level if recurs despite metformin<br>• If symptoms or signs of hyperglycaemia:<br>o Stop AZD5363 (for up to 21 days), add oral metformin and titrate dose according to blood glucose, consider admitting to hospital and appropriate medical management as per local guidelines<br>o Hold until blood glucose <8.9mmol/L or lower (recovery to Grade 1)<br>o Restart AZD5363 at the next lower dose level
Grade 4 hyperglycaemia Glucose >27.8mmol/L	• Discontinue permanently and treat with insulin sliding scale as per local guidelines

Use of metformin

Metformin is currently recommended for the management of hyperglycaemia occurring in patients participating in studies of AZD5363. Investigators should exercise caution in the dosing and management of patients receiving the metformin/AZD5363 combination and must be vigilant for signs of renal impairment and metformin toxicity, such as lactic acidosis and hypoglycaemia: lethargy, hypotension, poor urine output, drowsiness, irritation, tachypnoea, sweating, diarrhoea and vomiting.

Metformin should only be given on the days when AZD5363 is also administered (the half-life of AZD5363 is approximately 8–15 hours), and should be withdrawn when treatment with AZD5363 is withdrawn, unless otherwise clinically indicated. Metformin should be started at an initial dose of 500mg od.

Due to the potential interaction of metformin and AZD5363 as a result of inhibition of OCT2, when taking both AZD5363 and metformin concurrently patients should attend the clinic for monitoring of serum creatinine at least once per week for the first 3 weeks after initiation of metformin, then every 4 weeks thereafter.

Please contact ICR-CTSU with any queries on this issue.

Diarrhoea

Table C4. Cohort C diarrhoea management guidelines

Event	AZD5363 modification	Management of diarrhoea
Uncomplicated Grade ≤2 diarrhoea	None	• Dietary measures: patients should stop all lactose-containing products, drink 2L of clear liquids per day, and eat frequent small meals on a low fat regimen<br>• Loperamide: an initial dose of 4mg should be administered, followed by 2mg every 4 hours or after every unformed stool. Consider continuation of loperamide until patient is diarrhoea free for 12 hours
Diarrhoea Grade ≥3 or any grade with complications (dehydration, fever and/or Grade ≥3 neutropenia)	• If toxicity does not improve to Grade ≤2 withhold AZD5363 for up to 21 days<br>• If toxicity improves to Grade ≤2 reinstate AZD5363 at the next lower dose level maintaining treatment for toxicity as necessary	• Dietary measures: as per Grade ≤2 diarrhoea<br>• Loperamide: as per Grade ≤2 diarrhoea; if dehydration is severe, octreotide and intravenous fluids should be administered as appropriate.<br>• Prophylactic antibiotics should be considered if diarrhoea is persistent beyond 24h or there is a fever or Grade 3 or 4 neutropenia<br>• A Grade 4 event should be reported as an SAE

Rash

Table C5. Cohort C rash management guidelines

Event	AZD5363 modification	Management of maculopapular rash
Grade 1	• Start dermatological treatment and continue AZD5363 at current dose level	• Topical steroid moderate strength<br>• Oral antihistamine (if symptomatic)
Grade 2	• Start dermatological treatment and continue AZD5363 at current dose level	• Topical steroid moderate strength BID<br>• Oral antihistamine (if symptomatic)
Grade ≥3	• For any skin reaction Grade ≥3 withhold dose for up to 21 days until improvement of toxicity. Restart treatment as below:<br>• If toxicity improves to Grade ≤1 within 21 days restart AZD5363 at the current dose level<br>• If toxicity improves to Grade 2 within 21 days reinstate AZD5363 at the next lower dose level<br>• <u>If recurrence</u> of Grade ≥3 discontinue AZD5363	• Oral steroid for up to 2 weeks<br>• A Grade 4 event should be reported as an SAE

C5.8. Cohort C Dose Interruptions

In addition to the specific toxicity management guidelines within Section C5.7, AZD5363 treatment should be interrupted if patients experience any Grade ≥3 toxicity.

Following an interruption for Grade ≥3 toxicity, trial treatment should be delayed for up to 21 days until these toxicities have resolved to Grade ≤1 or returned to baseline. Treatment with AZD5363 should be resumed at the next lower dose level. Please see Section C5.7 for guidance on restarting AZD5363 after stopping due to hyperglycaemia, diarrhoea or rash.

If a patient remains off trial treatment for >3 weeks trial treatment should be permanently discontinued.

C5.9. Cohort C Missed Doses

If a dose of AZD5363 or fulvestrant is missed trial treatment should be resumed at the next scheduled dose. Missed doses should not be made up. If the patient vomits after taking a dose of AZD5363 they should be advised to resume treatment at the next scheduled dose.

C5.10. Cohort C Overdoses

There is currently no specific treatment in the event of an overdose with AZD5363 and possible symptoms of overdose are not established. If overdose occurs, this should be managed symptomatically. Please contact the ICR-CTSU for advice.

There is no human experience of over dosage of fulvestrant. Animal studies suggest that no effects other than those related directly or indirectly to anti-estrogenic activity were evident with higher doses of fulvestrant. If overdose occurs, this should be managed symptomatically. Please contact the ICR-CTSU for advice.

C5.11. Cohort C Discontinuation and Subsequent Therapy

Treatment should continue until disease progression, unacceptable toxicity or withdrawal of patient consent. Patients who discontinue trial treatment in the absence of disease progression should continue to have CT and/or MRI scans to assess disease status according to the trial assessment schedule, provided that the patient has not withdrawn their consent to further trial assessments.

On disease progression, patients with more than one actionable mutation identified at ctDNA screening may participate in a second cohort according to the hierarchy of treatment cohorts specified in Section 3.2.3 after the defined washout period specified in the second cohort specific eligibility criteria and provided they meet all other cohort specific eligibility criteria at the time of entry into the second cohort.

C5.12. Cohort C Compliance

Patients must be asked to bring all their trial medication every time they attend the clinic for the purposes of treatment compliance assessment and drug accountability. Every effort should be made to encourage patients to return the unused medication and empty bottles. The unused tablets

should be collected by the Investigator/Research Nurse and counted to ascertain patient compliance, medication will then be returned to pharmacy for drug accountability. Drug destruction should only be carried out with prior approval from ICR-CTSU and according to local destruction policy.

C5.13. Cohort C Supply and Distribution of AZD5363 and Fulvestrant

AZD5363 and fulvestrant are manufactured and provided free of charge by AstraZeneca to participating sites..

No drug will be distributed to participating sites unless ICR-CTSU is satisfied that the required approvals and agreements and initiation procedures are complete.

C5.14. Cohort C Formulation, Packaging, Storage Conditions and Labelling

AZD5363 will be supplied as beige film-coated tablets. AZD5363 tablets are packed in high-density polyethylene (HDPE) bottles. Bottles are secured with a child-resistant closure; induction-sealed membranes provide tamper evidence. Tablets should be stored in the bottles provided and taken according to the instructions on the label. AZD5363 should be stored below 30°C (86°F). Patients should be instructed to store AZD5363 in a safe place at room temperature.

Fulvestrant 50mg/ml solution will be supplied by AstraZeneca for use in the plasmaMATCH trial only. Complete information about fulvestrant formulation can be found in the SmPC for fulvestrant. Fulvestrant is supplied as two 5ml clear neutral glass (Type 1) barrels, each containing 250mg/5ml of fulvestrant solution for injection and fitted with a tamper evident closure. The syringes are presented in a tray with polystyrene plunger rod and safety needles (SafetyGlide™) for connection to the barrel. Fulvestrant should be stored at 2°C to 8°C (in a refrigerator) in the original packaging.

The drug distribution company is responsible for labelling AZD5363 and fulvestrant in accordance with the MHRA approved plasmaMATCH labels. Pharmacies may add their own hospital dispensing label to the trial drug but should not obscure the existing label on the drug packaging.

C5.15. Cohort C Pharmacy Responsibilities and Drug Accountability

AZD5363 and fulvestrant supplied for the plasmaMATCH trial must not be used outside the context of the plasmaMATCH protocol. Records must be kept of all deliveries, dispensing and destruction in accordance with the plasmaMATCH Pharmacy Guidance Notes. These records may be requested by ICR-CTSU during the trial to monitor supply and usage of stock. Account must be given of any discrepancies, and certificates of delivery and destruction must be signed and dated.

COHORT D:
AKT ACTIVATION BASKET MUTATIONS
TREATED WITH AZD5363

D1. COHORT D BACKGROUND AND RATIONALE

Genetic events other than *AKT1* mutation are likely to promote AKT activation, for example inactivating mutation or homozygous deletion of *PTEN* occurs in approximately 2% of breast cancers overall, and is observed in approximately 10% of triple negative breast cancers (5). Genetic loss of *PTEN* is associated with strong activation of AKT in breast cancers (42), and is associated with selective sensitivity to AKT inhibition *in vitro* (46). Mutation in *PIK3R1* activates AKT *in vitro* and may identify a further group of cancers sensitive to AKT kinase inhibitors (47). Mutations in *PIK3R1* occur at a low frequency (~1%) and both *PTEN* and *PIK3R1* will be included in plasmaMATCH Cohort D, a 'basket' cohort for treatment with AZD5363.

AZD5363 is a potent catalytic inhibitor of AKT1, AKT2 and AKT3 with evidence of activity both *in vivo* (45) and in the clinic (43), and will be used to target genetic events that activate AKT.

D2. KNOWN RISKS AND BENEFITS OF AZD5363

As of 10 July 2014, 194 patients had been recruited into phase I and II studies of AZD5363. Recognised toxicities include diarrhoea, rash, hyperglycaemia. Therefore in Cohort D of the plasmaMATCH trial, glucose will be monitored pre-dose and at 2 and 4 hours post drug administration on Day 1 of each cycle. Asymptomatic increases in glucose have appeared to be mitigated by the use of metformin in the majority of patients. Hypersensitivity reactions have also been seen in four patients, three on monotherapy and one in combination with paclitaxel. A rash was seen in combination with one or more associated clinical features, such as pruritus, urticaria, throat itchiness, pyrexia, facial and/or lip oedema. All cases resolved with drug discontinuation and treatment with antihistamines and steroids.

In a phase I trial (NCT01226316), patients received escalating doses of AZD5363, either continuous or intermittent and from this a dose of 480mg BID, 4 days on, 3 days off schedule was recommended. In a study of Japanese patients with advanced solid tumours (48) dose limiting toxicities were only found with continuous dosing. The 480mg BID 4 days on, 3 days off schedule was selected for further investigation and is currently proposed as the recommended phase II dose for monotherapy intermittent dosing. This is the schedule used in plasmaMATCH.

D3. COHORT D SPECIFIC ELIGIBILITY CRITERIA

Patients will be considered eligible for entry into treatment Cohort D if they fulfil all eligibility criteria presented below.

D3.1. Inclusion Criteria

1. Mutations of:
    - AKT1 identified in plasmaMATCH ctDNA screening in patients with ER negative breast cancer
    - OR *AKT1* identified in tumour sequencing conducted outside of the plasmaMATCH trial in an appropriately accredited laboratory in patients with ER positive or negative breast cancer
    - OR *AKT2/3* E17K*, PIK3R1* or *PTEN* or homozygous deletion of *PTEN* in both ER positive and ER negative breast cancer identified in plasmaMATCH ctDNA screening or in tumour sequencing conducted outside of the plasmaMATCH trial in an appropriately accredited laboratory (please refer to Section 10).
    If a patient has more than one intervening line of therapy between ctDNA screening and entry into the treatment cohort, the actionable mutation must be re-confirmed by repeat ctDNA screening prior to entry.
2. Signed Informed Consent Form for Entry into Treatment Cohort D.
3. Female.
4. Aged ≥18 years old.
5. Histologically confirmed invasive breast carcinoma.
6. Metastatic or recurrent locally advanced breast cancer that is not suitable for treatment with radical or curative intent.
7. Radiological evidence of disease progression following most recent line of therapy.
8. Measurable disease by RECIST v1.1 assessed by CT and/or MRI. Patients with bone only disease may be eligible if they have a soft tissue component that is measurable by RECIST v1.1. Any measurable lesion(s) that have previously been irradiated must have documented progression since the radiotherapy to be assessable.
9. Patients must have completed at least one prior line of treatment (chemotherapy, endocrine therapy or targeted therapy) for advanced breast cancer and/or relapse within 12 months of completing (neo)adjuvant chemotherapy. *Patients with HER2 positive breast cancer must have been treated with at least two lines of HER2 targeted therapy in the advanced setting (or one line if no further lines of HER2 targeted therapy are available locally).*
10. A maximum of two prior lines of cytotoxic chemotherapy, chemotherapy antibody-drug conjugate or immunotherapy alone in the advanced setting is permitted.
11. Patient must either be suitable for a baseline biopsy of recurrent disease or have an archival biopsy of recurrent disease available. *Patients are requested to consent to a baseline biopsy but if deemed unsafe by the Investigator, an archival biopsy of recurrent disease can be used instead. If it is deemed unsafe to proceed with baseline biopsy, and no archival recurrent disease biopsy is available, the patient will not be eligible for entry into the treatment cohort.*
12. ECOG performance status ≤2.
13. Patients must be able to swallow and retain oral medication.
14. Life expectancy >3 months.

15. Patients must be a) surgically sterile; b) have a sterilised sole partner; or c) be postmenopausal; or d) must agree to practice true abstinence; or e) use effective contraception during the period of trial treatment and be willing to do so for 6 months following the end of trial treatment. *True abstinence must be in line with the preferred and usual lifestyle of the patient. (Periodic abstinence, such as calendar, ovulation, symptothermal, post-ovulation methods, and withdrawal are not acceptable methods of contraception). Effective contraception is defined as double barrier contraception (e.g. condom plus spermicide in combination with a diaphragm, cervical cap or intrauterine device). Ovarian suppression with an LHRH agonist is not a method of contraception. Postmenopausal is defined by at least one of the following criteria:*
    - Age >60 years;
    - Age <60 years and cessation of regular menses for at least 12 consecutive months with no alternative pathological or physiological cause; and serum estradiol and follicle stimulating hormone (FSH) level within the laboratory's reference range for postmenopausal females;
    - Documented bilateral oophorectomy.
16. Patients of childbearing potential should have a negative serum or urine pregnancy test within 14 days prior to initiation of trial treatment.
17. At least 4 weeks washout period after the end of trial treatment on a different cohort within plasmaMATCH.
18. Adequate haematological, renal and hepatic function as defined by:
    - Haematology:
        - ANC ≥1000/mm^3 (≥1.0 x 10^9/L)
        - Platelet count ≥100,000/mm^3 (≥100 x 10^9/L)
        - Haemoglobin ≥9g/dL (≥90g/L)
    - Renal function:
        - Serum creatinine ≤1.5 ULN;
    - Liver function tests:
        - Total bilirubin ≤1.5 ULN*
        - ALT and AST ≤3 ULN. In the presence of liver metastases, AST and ALT ≤5 ULN.
19. Normal LVEF by an ECHO or MUGA assessed using local criteria.
20. Prior exposure to everolimus is allowed.

* Patients with known Gilbert's syndrome (normal conjugated bilirubin and otherwise normal liver function) are eligible, assuming conjugated bilirubin is within normal range.

D3.2. Exclusion Criteria

1. Prior treatment with radiotherapy (except for palliative reasons), endocrine therapy, immunotherapy, chemotherapy or IMPs during the previous 4 weeks (6 weeks for nitrosoureas, Mitomycin-C) before trial treatment, except for hormonal therapy with LHRH analogues, which are permitted, and bisphosphonates or RANK ligand antibodies that are permitted for the management of bone metastases.
2. Uncontrolled CNS disease (brain metastases or leptomeningeal disease). Patients with prior diagnosis of CNS metastases must be stable by clinical assessment having ceased steroids after prior treatment.

3. History of clinically significant or uncontrolled cardiac disease, including congestive heart failure, angina, myocardial infarction within the last 6 months or ventricular arrhythmia. *Patients with a history of any of the above listed cardiac conditions judged not to be clinically significant by the local Investigator must be notified to the trial team at the ICR-CTSU for approval by the CI and/or Cohort Lead.*
4. Ongoing toxic manifestations of previous treatments Grade ≥1. Exceptions to this are alopecia or toxicities which in the opinion of the Investigator should not exclude the patient. Such cases should be clearly documented in the patient's notes by the Investigator.
5. Major surgery (excluding minor procedures, e.g. placement of vascular access) within 4 weeks of the first dose of trial treatment.
6. Pregnant or breastfeeding.
7. Any condition that according to the treating physician may compromise the patient's safety or the conduct of the trial.
8. Current malignancies of other types, with the exception of adequately treated in situ carcinoma of the cervix and basal or squamous cell carcinoma of the skin. *Cancer survivors, who have undergone potentially curative therapy for a prior malignancy and have no evidence of the disease for 3 years or more are eligible for the trial.*
9. Glucose criteria: HbA1c ≥8.0% (64mmol/mol) at trial entry; fasting plasma glucose ≥7.0mmol/L (126mg/dL). Patients with diabetes mellitus may enter the trial unless any of the following exclusion criteria are fulfilled:
    - HbA1c >7.5% (58.5mmol/mol)
    - Baseline fasting glucose >8.4mmol/L or 152mg/dL (fasting is defined as no calorific intake for at least 8 hours)
    - Insulin required for routine diabetic management and control
    - More than 2 oral hypoglycaemic medications required for routine diabetic management and control
10. Prior exposure to agents with primary pharmacological activity of inhibition of AKT, including prior dosing of AZD5363.
11. History of malabsorption syndrome or other condition that would interfere with enteral absorption. For example active intestine inflammation (e.g. Crohn's disease or ulcerative colitis) requiring immunosuppressive therapy.
12. Patients requiring concurrent administration of CYP3A4 inhibitors and inducers.
13. Known hypersensitivity to AZD5363 and its excipients.

D4. COHORT D TRIAL ASSESSMENTS

The Cohort D Schedule of Assessments (Section D4.8) shows all required trial assessments in table form. All blood and tissue samples should be collected according to the instructions provided in the plasmaMATCH Investigator Laboratory Manual.

D4.1. Cohort D Screening Assessments

The following assessments should be conducted following identification of an AKT activation basket mutation, either in plasmaMATCH ctDNA screening or tumour sequencing performed outside of the plasmaMATCH trial, required for entry into the treatment Cohort D. Only those procedures required as part of standard patient care should be conducted prior to obtaining written informed consent from the patient for entry into the treatment cohort as detailed in Section 8.1.

The following assessments should be performed within 28 days prior to Cycle 1 Day 1:

- Medical history
- Physical examination and vital signs
- ECOG performance status
- Assessment of symptoms
- Review of concomitant medication
- Safety bloods: Haematology – full blood count, white cell count with differential and ANC, prothrombin time and INR; Biochemistry – sodium, potassium, calcium, magnesium, ALT, AST, GGT, bilirubin, albumin, creatinine, alkaline phosphatase
- HbA1c
- Fasting lipids (high-density lipoprotein (HDL), low-density lipoprotein (LDL), triglycerides, cholesterol)
- Bone scan
- CT and/or MRI scan to establish RECIST v1.1 baseline
- ECHO or MUGA
- Mandatory baseline recurrent tumour biopsy and/or provision of archival recurrent disease biopsy where it is deemed unsafe by the Investigator to take a baseline biopsy from the patient
- Provision of archival primary tumour sample. If an archival primary tumour sample is not available but another previously obtained sample is available, this sample should be provided instead

The following assessments should be conducted within 14 days prior to Cycle 1 Day 1:

- ECG
- Fasting plasma glucose
- Pregnancy test and contraceptive counselling for patients of childbearing potential

D4.2. Cohort D Baseline Assessments Pre-Treatment Cycle 1 Day 1

The following assessments should be performed within 7 days (including Cycle 1 Day 1) prior to commencing trial treatment:

- Physical examination and vital signs
- Assessment of symptoms

- Review of concomitant medications
- Safety bloods: Haematology – full blood count, white cell count with differential and ANC, prothrombin time and INR; Biochemistry – sodium, potassium, calcium, magnesium, ALT, AST, GGT, bilirubin, albumin, creatinine, alkaline phosphatase
- Non-fasting glucose: pre-treatment **and** 2 and 4 hours post AZD5363. NB. The pre-treatment non-fasting glucose test must be done prior to dosing on the day of treatment.
- Research blood samples:
    - 20ml blood sample collected in EDTA tube for plasma ctDNA analysis, to be processed within 1 hour at site
    - 10ml blood sample collected in serum tube for biomarker bloods
    - 3ml blood sample collected in EDTA tube for germline DNA

D4.3. Cohort D On-Treatment Assessments

D4.3.1. Cycle 1 Day 15 Pre-Treatment

- Physical examination (symptom directed) and vital signs
- Assessment of symptoms including clinical assessment of disease progression
- Dosing compliance
- Review of concomitant medications
- Review of AEs
- Safety bloods to be taken within 72 hours prior to Cycle 1 Day 15: Haematology – full blood count, white cell count with differential and ANC, prothrombin time and INR; Biochemistry – sodium, potassium, calcium, magnesium, ALT, AST, GGT, bilirubin, albumin, creatinine, alkaline phosphatase
- Non-fasting glucose: pre-dose **and** 2 and 4 hours post AZD5363. NB. The pre-treatment non-fasting glucose test must be done prior to dosing on the day of treatment.
- Research blood samples:
    - 20ml blood sample collected in EDTA tube for plasma ctDNA analysis, to be processed within 1 hour at site
    - 10ml blood sample collected in serum tube for biomarker bloods
- Research tissue sample:
    - Optional recurrent tumour biopsy (can be performed before or after trial treatment administration)

D4.3.2. Cycle 2, Day 1 Pre-Treatment

The following assessments should be performed as close to as possible and within -/+ 3 days of the due date of the visit:

- Physical examination and vital signs
- Assessment of symptoms including clinical assessment of disease progression
- Dosing compliance
- Review of concomitant medications
- Review of AEs
- Safety bloods to be taken within 72 hours prior to the start of Cycle 2: Haematology – full blood count, white cell count with differential and ANC, prothrombin time and INR; Biochemistry –

sodium, potassium, calcium, magnesium, ALT, AST, GGT, bilirubin, albumin, creatinine, alkaline phosphatase, urea
- Non-fasting glucose: pre-dose **and** 2 and 4 hours post AZD5363. NB. The pre-treatment non-fasting glucose test must be done prior to dosing on the day of treatment.
- ECG
- Research blood samples:
    - 20ml blood sample collected in EDTA tube for plasma ctDNA analysis, to be processed within 1 hour at site
    - 10ml blood sample collected in serum tube for biomarker bloods

D4.3.3. Cycle 3 Onwards, Day 1 Pre-Treatment

The following assessments should be performed as close to as possible and within -/+ 7 days of the due date of the visit:

- Physical examination and vital signs
- Assessment of symptoms including clinical assessment of disease progression
- Dosing compliance
- Review of concomitant medications
- Review of AEs
- Safety bloods to be taken within 72 hours prior to the start of each cycle: Haematology – full blood count, white cell count with differential and ANC, prothrombin time and INR; Biochemistry – sodium, potassium, calcium, magnesium, ALT, AST, GGT, bilirubin, albumin, creatinine, alkaline phosphatase, urea
- Non-fasting glucose: pre-dose **and** 2 and 4 hours post AZD5363. NB. The pre-treatment non-fasting glucose test must be done prior to dosing on the day of treatment.
- ECG
- CT and/or MRI scan every 8 weeks from Cycle 1 Day 1 until 32 weeks, then every 12 weeks thereafter
- RECIST v1.1 assessment every 8 weeks from Cycle 1 Day 1 until 32 weeks, then every 12 weeks thereafter
- Bone scan only if clinically indicated
- Fasting lipids, HbA1c and fasting glucose at Cycle 4, then every 12 weeks thereafter
- Research blood sample:
    - 20ml blood sample collected in EDTA tube for plasma ctDNA analysis, to be processed within 1 hour at site

D4.4. Cohort D End of Treatment Assessments

D4.4.1. Treatment Discontinuation Visit

The following assessments should be performed at the time of discontinuation of trial treatment for any reason:

- ECOG performance status
- Physical examination and vital signs
- Assessment of symptoms including clinical assessment of disease progression
- Dosing compliance
- Review of concomitant medications

- Review of AEs
- Safety bloods: Haematology – full blood count, white cell count with differential and ANC, prothrombin time and INR; Biochemistry – sodium, potassium, calcium, magnesium, ALT, AST, GGT, bilirubin, albumin, creatinine, alkaline phosphatase, urea
- Non-fasting glucose
- ECG
- Research blood sample:
    - 20ml blood sample collected in EDTA tube for plasma ctDNA analysis, to be processed within 1 hour at site

D4.4.2. End of Trial Treatment Visit 30 days after the Last Administration of Trial Treatment

The following assessments should be performed at the end of trial treatment visit, 30 days after the last administration of trial treatment:

- ECOG performance status
- Physical examination and vital signs
- Assessment of symptoms including clinical assessment of disease progression
- Review of concomitant medications
- Review of AEs
- Safety bloods: Haematology – full blood count, white cell count with differential and ANC, prothrombin time and INR; Biochemistry – sodium, potassium, calcium, magnesium, ALT, AST, GGT, bilirubin, albumin, creatinine, alkaline phosphatase, urea
- Non-fasting glucose
- ECG
- Research tissue sample:
    - Optional recurrent tumour biopsy

D4.5. Cohort D Post-Treatment Follow up

All patients should be followed up at 6 monthly intervals from the end of trial treatment until death or up to 2 years; assessment should be in line with standard practice and should include:

- Survival
- Further treatment

D4.6. Cohort D Discontinuation from Treatment

Patients may discontinue from trial treatment at any time at their own request, or they may be discontinued at the discretion of the PI. Protocol-specified reasons for discontinuation will include:

- Disease progression or recurrence
- Unacceptable toxicity
- Pregnancy
- Withdrawal of consent
- Serious non-attendance and/or persistent non-compliance with procedures defined in the trial protocol

Patients who discontinue trial treatment in the absence of disease progression should continue to have CT and/or MRI scans to assess disease status according to the trial assessment schedule. All patients who discontinue treatment should continue to be followed up. Patients will be asked for consent for future linkage with routinely collected health data (via national registries) to trace their eventual vital status and assess subsequent unexpected co-morbidities.

D4.7. Cohort D Discontinuation from Follow up

If a patient wishes to withdraw from further follow up, a Patient Withdrawal Form should be submitted to ICR-CTSU within the clinical trial database stating whether the patient has withdrawn consent for further information to be sent to the ICR-CTSU or whether they simply no longer wish to attend trial follow up visits.

In the very rare event that a patient requests that their data is removed from the trial entirely, the implications of this should be discussed with the patient first to ensure that this is their intent and, if confirmed, ICR-CTSU should be notified in writing. *The patient should be made aware that any information about them that has already been published or submitted to the authorities for safety monitoring purposes cannot be withdrawn.*

Should a patient withdraw consent for their samples to be used in plasmaMATCH, following receipt of written confirmation from the site to ICR-CTSU, blood samples will be destroyed and biopsy blocks returned to the site for archiving where requested or otherwise destroyed.

D4.8. Cohort D Schedule of Assessments

Procedures and assessments	Screening for entry into treatment cohort		Cycle 1 (Baseline and Week 2)		Cycle 2 (Week 4)	Cycle 3 (Week 8)	Cycle 4 onwards (Week 12, and 4 weekly thereafter until progression)	End of treatment visits		Follow up
	Day -28 to Day -1	Day -14 to Day -1	Day 1[1]	Day 15	Day 1[2]	Day 1[3]	Day 1[3]	Treatment discontinuation	Day 30 after last dose of drug	6 monthly from end of treatment until death or up to 2 years
AZD5363			AZD5363 taken on a 4 days on – 3 days off schedule continuously for each 28 day treatment cycle							
Informed consent	X									
Medical history	X									
ECOG performance status	X							X	X	
Obtain archival primary tissue[4]	X									
Recurrent tumour biopsy[5,6]	X									
**PLEASE NOTE, ASSESSMENTS SHOULD BE COMPELTED PRE-TREATMENT UNLESS OTHERWISE STATED**										
Recurrent tumour biopsy (optional)[6]				X					X	
Physical examination and vital signs	X		X	X	X	X	X	X	X	
Assessment of symptoms (including clinical evidence of disease progression)	X		X		X	X	X	X	X	
Dosing compliance				X	X	X	X	X		
Concomitant medications	X		X	X	X	X	X	X	X	
Adverse events				X	X	X	X	X	X[7]	
12 Lead ECG		X			X	X	X	X	X	
Pregnancy test[8]		X								

Continued overleaf

Procedures and assessments	Screening for entry into treatment cohort		Cycle 1 (Baseline and Week 2)		Cycle 2 (Week 4)	Cycle 3 (Week 8)	Cycle 4 onwards (Week 12, and 4 weekly thereafter until progression)	End of treatment visits		Follow up
	Day -28 to Day -1	Day -14 to Day -1	Day 1[1]	Day 15	Day 1[2]	Day 1[3]	Day 1[3]	Treatment discontinuation	Day 30 after last dose of drug	6 monthly from end of treatment until death or up to 2 years
HbA1c and fasting[9] lipid profile[10]	X						X Cycle 4 then 12 weekly			
Fasting[9] glucose		X					X Cycle 4 then 12 weekly			
Non-fasting glucose[11]			X pre-dose[11], 2 & 4 hours post-dose	X pre-dose[11], 2 & 4 hours post-dose	X pre-dose[11], 2 & 4 hours post-dose	X pre-dose[11], 2 & 4 hours post-dose	X pre-dose[11], 2 & 4 hours post-dose	X	X	
Safety bloods[12]	X		X[12]	X[12]	X[12]	X[12]	X[12]	X	X	
Biomarker bloods			X	X	X					
Plasma for ctDNA			X	X	X	X	X	X		
Whole blood for germline DNA			X							
Bone scan	X					(X) if clinically indicated	(X) if clinically indicated			
ECHO or MUGA	X									
CT and/or MRI scan	X					X	X 8 weekly from Cycle 1 Day 1 until 32 weeks then every 12 weeks[13] or if clinically indicated			

Continued overleaf

Procedures and assessments	Screening for entry into treatment cohort		Cycle 1 (Baseline and Week 2)		Cycle 2 (Week 4)	Cycle 3 (Week 8)	Cycle 4 onwards (Week 12, and 4 weekly thereafter until progression)	End of treatment visits		Follow up
	Day -28 to Day -1	Day -14 to Day -1	Day 1[1]	Day 15	Day 1[2]	Day 1[3]	Day 1[3]	Treatment discontinuation	Day 30 after last dose of drug	6 monthly from end of treatment until death or up to 2 years
Disease progression assessment (according to RECIST v1.1)	X					X	X 8 weekly from Cycle 1 Day 1 until 32 weeks then every 12 weeks			
Survival and further treatment										X

[1] Cycle 1 Day 1: Baseline assessments should be performed within 7 days (including Cycle 1 Day 1) prior to commencing trial treatment.

[2] Cycle 2 Day 1: Assessments should be performed as close to as possible and within -/+ 3 days of the due date of the visit; trial treatment should be administered as per the above schedule, however if not possible due to unavoidable circumstances treatment may be administered 1 day prior to or delayed for up to 3 days after the due date.

[3] Cycle 3 Day 1 onwards: Assessments should be performed as close to as possible and within -/+ 7 days of the due date of the visit; trial treatment should be administered as per the above schedule, however if not possible due to unavoidable circumstances treatment may be administered 1 day prior to or delayed for up to 7 days after the due date.

[4] Provision of archival primary tumour sample. If an archival primary tumour sample is not available but another previously obtained sample is available, this sample should be provided instead.

[5] Patients are requested to consent to a baseline biopsy, however if deemed unsafe by the Investigator, an archival biopsy of recurrent disease can be used instead.

[6] Recurrent tumour core biopsies can be ultrasound assisted or CT scan guided dependent on metastatic site and according to local practice.

[7] SAEs should be followed up until resolution.

[8] Patients of childbearing potential should have a negative serum or urine pregnancy test within 14 days prior to initiation of trial treatment.

[9] For the purposing of the fasting lipid profile and fasting glucose assessment fasting is defined as no calorific intake for at least 8 hours.

[10] Triglycerides, HDL, LDL, cholesterol.

[11] The pre-treatment non-fasting glucose test must be done prior to dosing on the day of treatment.

[12] Safety bloods should be taken within 7 days prior to Cycle 1 Day 1 and then within 72 hours prior to Cycle 1 Day 15 and the start of each cycle from Cycle 2 onwards: Haematology – full blood count, white cell count with differential and ANC, prothrombin time and INR; Biochemistry – sodium, potassium, calcium, magnesium, ALT, AST, bilirubin, GGT, albumin, creatinine, alkaline phosphatase, urea.

[13] Patients who experience treatment cycle delays should continue to have CT and/or MRI scans every 8 weeks from Cycle 1 Day 1 until 32 weeks, and then every 12 weeks thereafter.

Please note, all blood and tissue samples should be collected according to the instructions provided in the plasmaMATCH Investigator Laboratory Manual.

D5. COHORT D TRIAL TREATMENT

AZD5363 is an IMP within plasmaMATCH Cohort D.

D5.1. Cohort D Dose and Schedule

AZD5363 480mg BID should be administered orally on a 4 days on and 3 days off schedule. Trial treatment cycles are defined in 28 day periods. Patients must fast for 2 hours before and 1 hour after taking medication, and AZD5363 tablets must be swallowed intact.

Trial treatment should be administered as per the above schedule, however if this is not possible due to unavoidable circumstances (i.e. bank holidays) from Cycle 2 onwards Day 1 of trial treatment may be delayed for up to 3 days after the due date for Cycle 2, delayed for up to 7 days after the due date for Cycle 3 onwards.

For further guidance on trial treatment delay due to toxicity please refer to Sections D5.7 and D5.8.

D5.2. Cohort D Prescription and Dispensing

AZD5363 will be provided in non-patient-specific bottles. The patient's Trial ID should be recorded on the bottle label prior to dispensing. Patients should be instructed to keep their medication in the bottles provided and not transfer it to any other container.

All efforts should be made to ensure that patients clearly understand the directions for self-medication. Patients should be given a sufficient supply and unused drug and/or empty bottles should be returned at the appropriate time points. Returned unused medication must not be re-dispensed to any patient and should only be destroyed with prior approval from ICR-CTSU according to local destruction policy.

AZD5363 should be prescribed by the PI or Co-investigator and dispensed by the hospital pharmacy from trial stock supplied specifically for use within plasmaMATCH only.

D5.3. Cohort D Patient Cards and Treatment Diary Cards

A small wallet sized patient card template will be provided by ICR-CTSU for completion by the participating site. Each card will state:

- The name of the participating site
- That the patient is participating in the plasmaMATCH trial
- That the patient is taking AZD5363
- An emergency site contact number

Patients should be advised to keep their patient card in their possession at all times.

A treatment diary card will be provided by ICR-CTSU for completion by the patient, in order to record the number of tablets taken on each day of the treatment cycle.

D5.4. Cohort D Duration of Treatment

Patients will remain on treatment until progression. Patients may withdraw from trial treatment early if they experience unacceptable toxicity or if the treating clinician believes further treatment is no longer appropriate or at the patient's request.

D5.5. Cohort D Permitted Concomitant Therapy

All medication considered necessary for the patients' welfare and which is not expected to interfere with the evaluation of the trial drugs may be given at the discretion of the Investigator. All concomitant medications must be recorded in the patient's notes, as well as the appropriate section of the plasmaMATCH eCRF.

Bisphosphonates, RANK ligand antagonists and blood transfusions should be given as required at the discretion of the Investigator.

Palliative radiotherapy is acceptable if given for bony metastases as long as these are not indicative of disease progression. AZD5363 must be stopped 3 days before radiotherapy and restarted within 21 days.

Patients may take corticosteroids but increased vigilance is recommended on electrolyte and glucose levels.

D5.6. Cohort D Non-permissible Medications/Therapies

Investigational agents and other anticancer agents must not be given while the patient is on trial treatment.

Inhibitors or inducers of CYP3A4, or substrates of CYP3A4, CYP2B6, CYP2C9, CYP2C19 and CYP2D6 may affect the metabolism of AZD5363. Please see Appendix 7 for further details.

D5.7. Cohort D Dose Modifications

Every effort should be made to administer AZD5363 at the planned dose and schedule. However, patients experiencing toxicities related to the trial treatment may have their dose modified as outlined in this section.

A dose reduction of AZD5363 is recommended if the patient has a Grade 2 toxicity (not including alopecia) lasting >3 weeks despite appropriate supportive treatment or for any Grade ≥3 toxicity (based on NCI CTCAE v4.0). These dose reductions should be applied to subsequent cycles unless a further dose reduction is required. Once a reduction is made the patient should not increase back to a higher dose level.

Table D1. Cohort D AZD5363 dose reduction guidelines

Dose level	AZD5363
Starting dose	480mg BID, 4 days on – 3 days off
First dose reduction	400mg BID, 4 days on – 3 days off
Second dose reduction	320mg BID, 4 days on – 3 days off
Third dose reduction	Discontinue trial treatment

D5.7.1.1. Specific Toxicities Requiring AZD5363 Dose Modifications

Hyperglycaemia

Table D2. Cohort D hyperglycaemia management guidelines

Event	Action
Grade ≤1 hyperglycaemia<br>Glucose <8.9mmol/L	• Continue AZD5363 at the current dose level
Grade 2 hyperglycaemia<br>Glucose ≥8.9–13.9mmol/L	• Continue AZD5363 at the current dose level, monitor glucose (as per local guidelines)
Grade 3 hyperglycaemia<br>Glucose >13.9–27.8mmol/L	• If asymptomatic:<br>o Stop AZD5363 (for up to 21 days)<br>o Hold until blood glucose 13.9mmol/L or lower (recovery to Grade ≤2)<br>o Add oral metformin on the days when AZD5363 is re-administered at the current dose level<br>o Dose reduce AZD5363 to the next lower dose level if recurs despite metformin<br>• If symptoms or signs of hyperglycaemia:<br>o Stop AZD5363 (for up to 21 days), add oral metformin and titrate dose according to blood glucose, consider admitting to hospital and appropriate medical management as per local guidelines<br>o Hold until blood glucose <8.9mmol/L or lower (recovery to Grade 1)<br>o Restart AZD5363 at the next lower dose level
Grade 4 hyperglycaemia<br>Glucose >27.8mmol/L	• Discontinue permanently and treat with insulin sliding scale as per local guidelines

Use of metformin

Metformin is currently recommended for the management of hyperglycaemia occurring in patients participating in studies of AZD5363. Investigators should exercise caution in the dosing and management of patients receiving the metformin/AZD5363 combination and must be vigilant for signs of renal impairment and metformin toxicity, such as lactic acidosis and hypoglycaemia: lethargy, hypotension, poor urine output, drowsiness, irritation, tachypnoea, sweating, diarrhoea and vomiting.

Metformin should only be given on the days when AZD5363 is also administered (the half-life of AZD5363 is approximately 8–15 hours), and should be withdrawn when treatment with AZD5363 is withdrawn, unless otherwise clinically indicated. Metformin should be started at an initial dose of 500mg od.

Due to the potential interaction of metformin and AZD5363 as a result of inhibition of OCT2, when taking both AZD5363 and metformin concurrently patients should attend the clinic for monitoring of serum creatinine at least once per week for the first 3 weeks after initiation of metformin, then every 4 weeks thereafter.

Please contact ICR-CTSU with any queries on this issue.

Diarrhoea

Table D3. Cohort D diarrhoea management guidelines

Event	AZD5363 modification	Management of diarrhoea
Uncomplicated Grade ≤2 diarrhoea	None	• Dietary measures: patients should stop all lactose-containing products, drink 2L of clear liquids per day, and eat frequent small meals on a low fat regimen<br>• Loperamide: an initial dose of 4mg should be administered, followed by 2mg every 4 hours or after every unformed stool. Consider continuation of loperamide until patient is diarrhoea free for 12 hours
Diarrhoea Grade ≥3 or any grade with complications (dehydration, fever and/or Grade ≥3 neutropenia)	• If toxicity does not improve to Grade ≤2 withhold AZD5363 for up to 21 days<br>• If toxicity improves to Grade ≤2 reinstate AZD5363 at the next lower dose level maintaining treatment for toxicity as necessary	• Dietary measures: as per Grade ≤2 diarrhoea<br>• Loperamide: as per Grade ≤2 diarrhoea; if dehydration is severe, octreotide and intravenous fluids should be administered as appropriate.<br>• Prophylactic antibiotics should be considered if diarrhoea is persistent beyond 24h or there is a fever or Grade 3 or 4 neutropenia<br>• A Grade 4 event should be reported as an SAE

Rash

Table D4. Cohort D rash management guidelines

Event	AZD5363 modification	Management of maculopapular rash
Grade 1	• Start dermatological treatment and continue AZD5363 at current dose level	• Topical steroid moderate strength<br>• Oral antihistamine (if symptomatic)
Grade 2	• Start dermatological treatment and continue AZD5363 at current dose level	• Topical steroid moderate strength BID<br>• Oral antihistamine (if symptomatic)
Grade ≥3	• For any skin reaction Grade ≥3 withhold dose for up to 21 days until improvement of toxicity. Restart treatment as below:<br>• If toxicity improves to Grade ≤1 within 21 days restart AZD5363 at the current dose level<br>• If toxicity improves to Grade 2 within 21 days reinstate AZD5363 at the next lower dose level<br>• If recurrence of Grade ≥3 discontinue AZD5363	• Oral steroid for up to 2 weeks<br>• A Grade 4 event should be reported as an SAE

D5.8. Cohort D Dose Interruptions

In addition to the specific toxicity management guidelines within Section D5.7, AZD5363 treatment should be interrupted if patients experience any Grade ≥3 toxicity.

Following an interruption for Grade ≥3 toxicity, trial treatment should be delayed for up to 21 days until these toxicities have resolved to Grade ≤1 or returned to baseline. Treatment with AZD5363 should be resumed at the next lower dose level. Please see Section D5.7 for guidance on restarting AZD5363 after stopping due to hyperglycaemia, diarrhoea, or rash.

If a patient remains off trial treatment for >3 weeks trial treatment should be permanently discontinued.

D5.9. Cohort D Missed Doses

If a dose of AZD5363 is missed trial treatment should be resumed at the next scheduled dose. Missed doses should not be made up. If the patient vomits after taking a dose of AZD5363 they should be advised to resume treatment at next scheduled dose.

D5.10. Cohort D Overdoses

There is currently no specific treatment in the event of an overdose with AZD5363 and possible symptoms of overdose are not established. If overdose occurs, this should be managed symptomatically. Please contact the ICR-CTSU for advice.

D5.11. Cohort D Discontinuation and Subsequent Therapy

Treatment should continue until disease progression, unacceptable toxicity or withdrawal of patient consent. Patients who discontinue trial treatment in the absence of disease progression should continue to have CT and/or MRI scans to assess disease status according to the trial assessment schedule, provided that the patient has not withdrawn their consent to further trial assessments.

On disease progression, patients with more than one actionable mutation identified at ctDNA screening may participate in a second cohort according to the hierarchy of treatment cohorts specified in Section 3.2.3 after the defined washout period specified in the second cohort specific eligibility criteria and provided they meet all other cohort specific eligibility criteria at the time of entry into the second cohort.

D5.12. Cohort D Compliance

Patients must be asked to bring all their trial medication every time they attend the clinic for the purposes of treatment compliance assessment and drug accountability. Every effort should be made to encourage patients to return the unused medication and empty bottles. The unused tablets should be collected by the Investigator/Research Nurse and counted to ascertain patient compliance, medication will then be returned to pharmacy for drug accountability. Drug destruction should only be carried out with prior approval from ICR-CTSU and according to local destruction policy.

D5.13. Cohort D Supply and Distribution of AZD5363

AZD5363 is manufactured and provided free of charge by AstraZeneca to participating sites.

No drug will be distributed to participating sites unless ICR-CTSU is satisfied that the required approvals and agreements and initiation procedures are complete.

D5.14. Cohort D Formulation, Packaging, Storage Conditions and Labelling

AZD5363 will be supplied as beige film-coated tablets. AZD5363 tablets are packed in HDPE bottles. Bottles are secured with a child-resistant closure; induction-sealed membranes provide tamper

evidence. Tablets should be stored in the bottles provided and taken according to the instructions on the label. AZD5363 should be stored below 30°C (86°F). Patients should be instructed to store AZD5363 in a safe place at room temperature.

AstraZeneca are responsible for labelling AZD5363 in accordance with the MHRA approved plasmaMATCH label. Pharmacies may add their own hospital dispensing label to the trial drug but should not obscure the existing label on the drug packaging.

D5.15. Cohort D Pharmacy Responsibilities and Drug Accountability

AZD5363 supplied for the plasmaMATCH trial must not be used outside the context of the plasmaMATCH protocol. Records must be kept of all deliveries, dispensing and destruction in accordance with the plasmaMATCH Pharmacy Guidance Notes. These records may be requested by ICR-CTSU during the trial to monitor supply and usage of stock. Account must be given of any discrepancies, and certificates of delivery and destruction must be signed and dated.

COHORT E:
TRIPLE NEGATIVE BREAST CANCER TREATED WITH OLAPARIB AND AZD6738

E1. COHORT E BACKGROUND AND RATIONALE

10-15% of breast cancers do not express hormone receptors (ER or progesterone receptor (PgR)) nor are HER2 amplified. This triple negative breast cancer (TNBC) subgroup represents approximately 6,000 women per year in the UK. TNBC is highly proliferative and aggressive, and identification of novel therapeutic strategies for TNBC is vital. Patients with TNBC who do not test positive for any of the actionable mutations within plasmaMATCH are currently lacking therapeutic options. Cohort E aims to explore the efficacy of the combination of olaparib with AZD6738 in patients with TNBC who are not eligible for Cohorts A to D, and to establish biomarkers of sensitivity to the combination.

Olaparib (AZD2281, KU-0059436) is a potent inhibitor of polyadenosine 5'diphosphoribose polymerase (PARP) developed as a monotherapy as well as for combination with other anti-cancer agents. PARP inhibitors have high activity in cancers with defective homologous recombination (HR) DNA repair. The PARP enzyme is required for single strand break DNA repair, and cancer cell lines with defective HR are unable to tolerate the DNA damage that results from PARP inhibition, resulting in cell cycle arrest and apoptosis. Both BRCA1 and BRCA2 are needed for HR and consequently cancer cell lines deficient in BRCA1 or BRCA2 are highly sensitive to PARP inhibitors (49). Approximately 10-15% of TNBCs have a deleterious mutation in BRCA1 and BRCA2, either germline or sporadic mutation. In addition a proportion of sporadic TNBCs may have reduced BRCA1 expression or BRCA1 promoter methylation that results in a loss of BRCA1 expression. It is estimated that as a result, up to 40-60% of sporadic TNBCs also have evidence of defective HR and therefore would benefit from PARP inhibitor treatment (50).

AZD6738 is a potent, selective inhibitor of the serine/threonine-specific protein kinase, ataxia telangiectasia and Rad3-related protein (ATR). ATR is an apical kinase in one of the DNA-damage induced checkpoint pathways. During normal DNA replication ATR is recruited at stalled replication forks, which can progress to double strand breaks if left unrepaired. Recruitment and activation of ATR leads to cell cycle arrest in the S phase while the DNA is repaired, the stalled replication fork resolved, or there is nuclear fragmentation and apoptosis. Loss of ATR function leads to the inability to resolve stalled replication forks, resulting in the accumulation of DNA damage and cell death.

Increasing the exogenous replication stress in combination with PARP inhibitors such as olaparib could increase the sensitivity of ATR inhibitors and have improved efficacy in the tumour with manageable toxicity for the normal tissues. The combination of olaparib with an ATR inhibitor is therefore hypothesised to be active in TNBC for multiple reasons: through targeting the underlying defect in HR that may be present in 40-60% of tumours; and targeting the high degree of replication stress that is observed in TNBC.

This hypothesis is supported by pre-clinical models which have shown that TNBC may be highly sensitive to the combination of PARP inhibitors and DNA damage response kinase inhibitors such as ATR inhibitors. Cell line studies using early in vitro probe ATR inhibitor NU6027 in combination with PARP inhibitors showed significantly greater anti-tumour activity than either agent alone (51). In olaparib combination studies against primary explant tumour models in vivo, AZD6738 showed synergistic anti-tumour efficacy with ~100% tumour growth control when dosed using a 5-day on/2-day off intermittent, weekly schedule (Figure E1). The combination efficacy was significantly greater than that achieved by either olaparib or monotherapy treatments alone. The combination is also

well tolerated using this intermittent, weekly dosing schedule with no significant body weight loss noted.

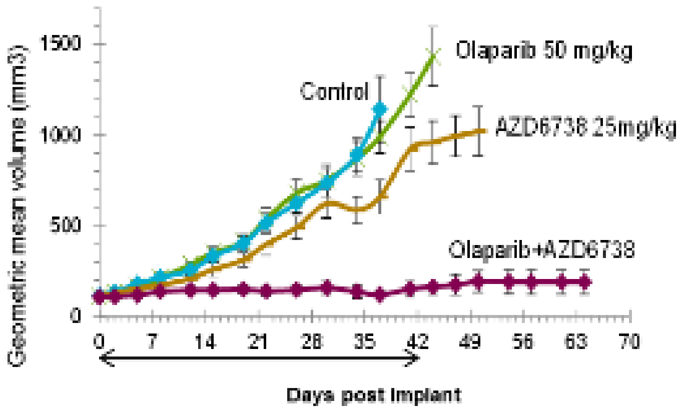

Figure E1. Olaparib + AZD6738 combination efficacy in a triple negative breast cancer patient derived xenograft (52)

E2. COHORT E DESIGN

Cohort E will recruit patients with TNBC who do not have an actionable mutation identified at ctDNA screening for entry into treatment Cohorts A to D (or via tumour sequencing for entry into Cohort D). Patients will receive the combination of olaparib and AZD6738. Biomarkers that identify sensitivity to the combination have not yet been validated and the aim of Cohort E is to establish biomarkers for the combination, principally using ctDNA assays, which can be validated in subsequent potential future cohorts.

Cohort E will use a two-stage design with confirmed objective response as the primary endpoint. 37 patients will be entered into Cohort E Stage 1. When all 37 patients have been followed up for a minimum of 4 months an interim analysis will take place to evaluate the best confirmed response rate. If between 5 and 18 responses are observed a further 32 patients will be recruited into Cohort E Stage 2 otherwise recruitment into Cohort E will stop (see Figure E2). After recruiting a total of 69 patients into Cohort E, potential biomarkers of response will be analysed to identify a biomarker in ctDNA that could subsequently be validated in a future cohort. (For further details on the statistical considerations for the design of Cohort E please refer to Section 13.2.5).

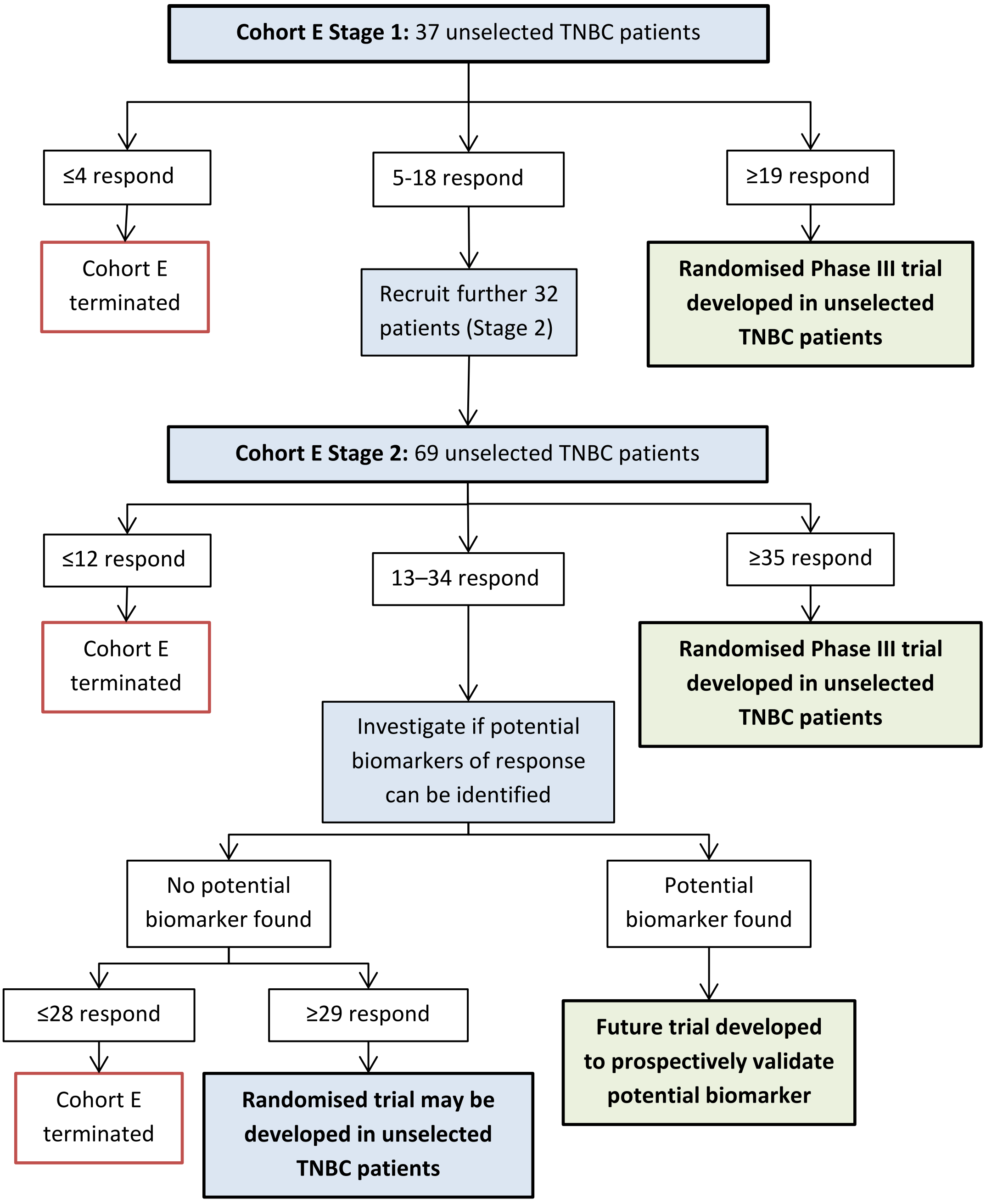

Figure E2. Cohort E two-stage design

E3. KNOWN RISKS AND BENEFITS OF OLAPARIB AND AZD6738

As of 15 December 2016, 6558 patients with ovarian, breast, pancreatic, gastric and a variety of other solid tumours have received treatment with olaparib in clinical studies as either monotherapy or in combination with other chemotherapy/anti-cancer agents (53). Olaparib monotherapy is generally well tolerated at monotherapy doses up to 400mg twice daily (capsule formulation) and 300mg twice daily (tablet formulation) in patients with solid tumours. Adverse event reports considered to be associated with administration of olaparib are generally mild or moderate (CTCAE Grade 1 or 2) haematological effects (anaemia, neutropenia, lymphopenia, thrombocytopenia, MCV elevation), decreased appetite, nausea and vomiting, diarrhoea, rash, hypersensitivity, dyspepsia, stomatitis, upper abdominal pain, dysgeusia, fatigue (including asthenia), increase in blood

creatinine, headache and dizziness. In a small number of patients, pneumonitis, myelodysplastic syndromes (MDS)/acute myeloid leukaemia (AML) and new primary malignancies have been reported, however data from the development programme does not support a conclusion that there is a causal relationship between olaparib and these events. These important potential risks for olaparib are being kept under close pharmacosurveillance.

There are currently five ongoing AZ sponsored clinical trials involving AZD6738. D5330C0004 (NCT02264678) is a Phase I modular clinical trial in which patients are receiving AZD6738 in combination therapy. As of 01 June 2017, 116 patients with advanced malignancies have been recruited into this trial and have received combinations of AZD6738 with carboplatin (Module 1), AZD6738 with olaparib (Module 2) or AZD6738 with durvalumab (Module 3). Preliminary findings from this trial were presented by Yap et al at the EORTC-AACR-NCI annual meeting in November 2016 (52). D5330C00007 is a biomarker study in squamous head and neck cancer; ACE-CL-110 is a study of AZD6738 monotherapy and combination of AZD6738 plus acalabrutinib in relapsed and refractory high risk chronic lymphocytic leukaemia, and at the time of this writing has recruited a single patient. D5336C00001 is a randomized Phase 2 study that will assess the efficacy and safety of olaparib monotherapy versus olaparib in combination with AZD6738, and olaparib monotherapy versus olaparib in combination with an inhibitor of WEE1 (AZD1775) in second or third line patients with TNBC, prospectively stratified by presence/absence of qualifying tumour mutations in genes involved in homologous recombination repair. D5336C00001 is open to recruitment but at this time has not dosed any patients. D6185C00001 is an umbrella study in non-small cell lung cancer in patients who have progressed on prior PD-1/PD-L1 therapy, and includes the combination of AZD6738 plus durvalumab. In addition, there are two open externally sponsored trials in which AZD6738 is being evaluated as monotherapy or in combination with radiotherapy (Study D5330C00002/PATRIOT study) or in combination with paclitaxel (VIKTORY study). A further two trials will explore the combination of AZD6738 plus olaparib in recurrent ovarian cancer (CAPRI) and small cell lung cancer (SUKSES-N2).

As of 01 December 2017, 92 patients have received treatment with the combination of AZD6738 with olaparib in Module 2 of D5330C0004. The recommended Phase II dose has been established at AZD6738 160mg once daily administered on Days 1–7 in combination with olaparib 300mg twice daily administered continuously in a 28-day cycle. This dosing schedule for AZD6738 is supported by the PK-PD model of thrombocytopenia, predicting a period of 21 days free of drug to achieve a full platelet recovery. The recommended dose of AZD6738 160mg once daily is predicted to maintain AZD6738 mean steady state concentrations above the estimated IC_{90} threshold (based on ATR enzyme inhibition assay in LoVo cells) and the GI_{90} threshold (based on the cellular growth inhibition activity in LoVo cells) across the full dosing interval i.e. 24 hours. In addition, this daily dose level was associated with a decrease in peripheral monocytes in the majority of patients and the preliminary blood cell count data from D5330C00004 and D5330C00002 trials suggests this decrease to be AZD6738 specific and dose dependant (monocyte decreases were not observed with either single agent olaparib or durvalumab). Monocytes have been characterised as being deficient in DNA base excision repair and PARP1 expression, suggesting an on-target synthetic lethal effect of AZD6738 mediated ATR inhibition in this cell type. Utilizing the monocyte decrease as a quantitative measure of AZD6738 pharmacological activity, the recommended Phase II dose of AZD6738 160mg once daily on Days 1–7 was driven by maintaining maximally active exposure consistent with manageable

safety. Four expansion cohorts with the same dose and schedule are recruiting patients with ataxia telangiectasia mutated (ATM) deficient (Part B1) and ATM proficient (Part B2) advanced gastric cancer, and HER2 negative, BRCA1 or BRCA2 mutated breast cancer in the second or third line setting (Part B3) and patients with TNBC with no known mutations in BRCA1 or BRCA2 but enriched for mutations in other homologous recombination repair genes in the second or third lines setting (Part B4).

Dose-limiting toxicities of AZD6738 and olaparib observed in the escalation phase included three events of Grade 4 thrombocytopenia associated with bleeding (one each of haematuria, epistaxis, and bleeding from a chest wall tumour) and a prolonged Grade 4 neutropenia. Common adverse events considered possibly related by Investigator assignment of causality include anaemia, thrombocytopenia, neutropenia, leucopenia, fatigue, anorexia, nausea and vomiting, diarrhoea and asthenia. Effects on bone marrow are anticipated in the clinic and may occur in the second or third week of dosing but may also arise after the first cycle resulting in dosing delays. These events are deemed schedule limiting, rather than dose limiting toxicities, as the main issue is a delayed recovery of the platelets. Myelosuppression has been successfully managed with dose interruptions, dose reductions (dose and schedule) and supportive measures such as blood transfusions. Haematology and biochemistry blood counts will be monitored at each cycle in the clinic and patients will be excluded from entry into Cohort E if they have a history of gastrointestinal bleeding or peptic ulceration.

The emerging data from the clinical development programme has identified risks which were predicted from the pre-clinical data and the mode of action of the drug. These risks have been assessed and addressed accordingly by incorporation of dose modification guidelines into the protocol. These risks are considered manageable within clinical practice and AZ support the administration of AZD6738 in combination with olaparib to patients with advanced TNBC according to the protocol.

E4. COHORT E SPECIFIC ELIGIBILITY CRITERIA

Patients will be considered eligible for entry into treatment Cohort E if they fulfil all eligibility criteria presented below.

E4.1. Inclusion Criteria

1. No actionable mutations identified at ctDNA screening or patient has an actionable mutation identified at ctDNA screening but does not meet the relevant cohort specific eligibility criteria for Cohorts A to D **or the relevant cohort is closed to recruitment**.
2. Triple negative breast cancer (TNBC) on the most recent tumour biopsy. *TNBC defined as ER negative, PgR negative (ER and PgR negative as defined by Allred score 0/8 or 2/8 or stain in <1% of cancer cells) or PgR unavailable, and HER2 negative (immunohistochemistry 0/1+ or negative in situ hybridization) as determined by local laboratory.*
3. Signed Informed Consent Form for Entry into Treatment Cohort E.
4. Female.
5. Aged ≥18 years old.
6. Histologically confirmed invasive breast carcinoma.

7. Metastatic or recurrent locally advanced breast cancer that is not suitable for treatment with radical or curative intent.
8. Radiological evidence of disease progression following most recent line of therapy.
9. Measurable disease by RECIST v1.1 assessed by CT and/or MRI. Patients with bone only disease may be eligible if they have a soft tissue component that is measurable by RECIST v1.1. Any measurable lesion(s) that have previously been irradiated must have documented progression since the radiotherapy to be assessable.
10. Patients must have completed at least one prior line of treatment (chemotherapy or targeted therapy) for advanced breast cancer and/or relapse within 12 months of completing (neo)adjuvant chemotherapy.
11. A maximum of two prior lines of cytotoxic chemotherapy, chemotherapy antibody-drug conjugate or immunotherapy alone in the advanced setting is permitted.
12. Patient must either be suitable for a baseline biopsy of recurrent disease or have an archival biopsy of recurrent disease available. *Patients are requested to consent to a baseline biopsy but if deemed unsafe by the Investigator, an archival biopsy of recurrent disease can be used instead. If it is deemed unsafe to proceed with baseline biopsy, and no archival recurrent disease biopsy is available, the patient will not be eligible for entry into the treatment cohort.*
13. ECOG performance status ≤2.
14. Patients must be able to swallow and retain oral medication.
15. Life expectancy >16 weeks.
16. Patients must be a) surgically sterile; b) have a sterilised sole partner; or c) be postmenopausal; or d) must agree to practice true abstinence; or e) use effective contraception during the period of trial treatment and be willing to do so for 6 months following the end of trial treatment. *True abstinence must be in line with the preferred and usual lifestyle of the patient. (Periodic abstinence, such as calendar, ovulation, symptothermal, post-ovulation methods, and withdrawal are not acceptable methods of contraception). Effective contraception is defined as double barrier contraception (e.g. condom plus spermicide in combination with a diaphragm, cervical cap or intrauterine device). Ovarian suppression with an LHRH agonist is not a method of contraception. Postmenopausal is defined by at least one of the following criteria:*
    - Age >60 years;
    - Age <60 years and cessation of regular menses for at least 12 consecutive months with no alternative pathological or physiological cause; and serum estradiol and follicle stimulating hormone (FSH) level within the laboratory's reference range for postmenopausal females;
    - Documented bilateral oophorectomy.
17. Patients of childbearing potential should have a negative serum or urine pregnancy test within 14 days prior to initiation of trial treatment.
18. Adequate haematological, renal and hepatic function as defined by:
    - Haematology:
        - ANC ≥1500/mm^3 (≥1.5 x 10^9/L)
        - Platelet count ≥100,000/mm^3 (≥100 x 10^9/L) with no platelet transfusions in the 28 days prior to screening
        - Haemoglobin ≥10g/dL (≥100g/L) with no blood transfusion or erythropoietin in the 28 days prior to screening

    - INR <1.5
  - Renal function:
    - Serum creatinine ≤1.5 ULN
    - Calculated creatinine clearance ≥51 mL/min using the Cockcroft-Gault equation (please refer to Appendix 5)
  - Liver function tests:
    - Total bilirubin ≤1.5 ULN*
    - ALT and AST ≤2.5 ULN. In the presence of liver metastases, AST and ALT ≤5 ULN.
- * Patients with known Gilbert's syndrome (normal conjugated bilirubin and otherwise normal liver function) are eligible, assuming conjugated bilirubin is within normal range.

E4.2. Exclusion Criteria

1. Prior treatment with radiotherapy (except for palliative reasons which must have been completed 3 weeks or more before Cycle 1 Day 1), endocrine therapy, chemotherapy or IMPs during the previous 3 weeks (6 weeks for nitrosoureas, Mitomycin-C), or immunotherapy during the previous 6 weeks before trial treatment. Bisphosphonates or RANK ligand antibodies that are permitted for the management of bone metastases should have been started at least 5 days prior to study treatment.
2. Known uncontrolled CNS disease (brain metastases or leptomeningeal disease); a scan to confirm the absence of brain metastases is not required. Patients with prior diagnosis of CNS metastases may be included if they meet the following criteria: disease outside the CNS is present, no evidence of progression since completion of CNS-directed therapy, minimum of 3 weeks between completion of radiotherapy and Cycle 1 Day 1 and recovery from significant (Grade ≥3) acute toxicity, ceased steroids after prior treatment and clinically stable.
3. History of clinically significant or uncontrolled cardiac disease currently or within the last 6 months defined by NYHA ≥ Class 2 including: congestive heart failure, unstable angina, myocardial infarction, or conduction abnormalities not controlled with a pacemaker or medication (patients with chronic rate-controlled atrial fibrillation in the absence of other cardiac abnormalities are eligible). *Patients with a history of any of the above listed cardiac conditions judged not to be clinically significant by the local Investigator must be notified to the trial team at the ICR-CTSU for approval by the CI and/or Cohort Lead.*
4. Ongoing toxic manifestations of previous treatments Grade ≥1. Exceptions to this are alopecia or toxicities which in the opinion of the Investigator should not exclude the patient. Such cases should be clearly documented in the patient's notes by the Investigator.
5. Major surgery (excluding minor procedures, e.g. placement of vascular access) within 4 weeks of the first dose of trial treatment (patients must have recovered from any effects of major surgery).
6. Patients receiving formal anti-coagulation treatment (including warfarin, novel oral anti-coagulants and LMWH).
7. Pregnant or breastfeeding.
8. Immunocompromised patients e.g. patients known to be serologically positive for HIV, or with active hepatitis.
9. Previous allogenic bone marrow transplant or double umbilical cord blood transplantation (dUCBT).

10. Whole blood transfusions in the last 4 months prior to entry to the study (packed red blood cells and platelet transfusions are acceptable outside of 28 days prior to treatment).
11. Any condition that according to the treating physician may compromise the patient's safety or the conduct of the trial.
12. Current malignancies of other types, with the exception of adequately treated in situ carcinoma of the cervix and basal or squamous cell carcinoma of the skin. *Cancer survivors, who have undergone potentially curative therapy for a prior malignancy and have no evidence of the disease for 3 years or more are eligible for the trial.*
13. Prior exposure to a PARP inhibitor (including olaparib), ATR inhibitor, or CHK1 inhibitor.
14. History of malabsorption syndrome or other condition that would interfere with enteral absorption. For example, refractory nausea and vomiting, active intestinal inflammation (e.g. Crohn's disease or ulcerative colitis) requiring immunosuppressive therapy.
15. Known hypersensitivity to olaparib and its excipients.
16. Known hypersensitivity to AZD6738 and its excipients.
17. Patients requiring concurrent administration of strong or moderate CYP34A inducers or inhibitors, or medications known to be sensitive CYP4A4 substrates or CYP3A4 substrates with a narrow therapeutic window. *The required washout period prior to starting study treatment is 2 weeks for inhibitors, 3 weeks for inducers, and 5 weeks for enzalutamide or phenobarbital*. *Patients should stop using herbal medication 7 days prior to the first dose of study treatment and for the duration of study treatment.*
18. Mean resting corrected QT interval (QTc) >470ms obtained on triplicate screening ECG performed 2-5 mins apart using Fredericia formula or known personal or family history of congenital QT-prolongation or Torsade de Pointes
19. Patients with myelodysplastic syndrome (MDS)/acute myeloid leukaemia (AML) or with features suggestive of MDS/AML.
20. Previous GI haemorrhage or peptic ulceration.
21. If patients have received prior platinum-containing therapy for metastatic disease patients should have achieved a PR/CR or SD and not have progressed during or within 8 weeks of receipt of last dose of platinum.

E5. COHORT E TRIAL ASSESSMENTS

The Cohort E Schedule of Assessments (Section E5.8) shows all required trial assessments in table form. All blood and tissue samples should be collected according to the instructions provided in the plasmaMATCH Investigator Laboratory Manual.

E5.1. Cohort E Screening Assessments

The following assessments should be conducted following consent to Cohort E. Only those procedures required as part of standard patient care should be conducted prior to obtaining written informed consent from the patient for entry into the treatment cohort as detailed in Section 8.1.

The following assessments should be performed within 28 days prior to Cycle 1 Day 1:

- Medical history
- Physical examination and vital signs
- ECOG performance status
- Assessment of symptoms
- Review of concomitant medication
- Safety bloods: Haematology – full blood count, white cell count with differential and ANC, prothrombin time and INR; Biochemistry – sodium, potassium, calcium, magnesium, ALT, AST, GGT, bilirubin, albumin, creatinine, alkaline phosphatase, glucose, urea, lactate dehydrogenase (LDH)
- Urinalysis by dipstick (blood, albumin, glucose)
- Bone scan
- CT and/or MRI scan to establish RECIST v1.1 baseline
- Mandatory baseline recurrent tumour biopsy and/or provision of archival recurrent disease biopsy where it is deemed unsafe by the Investigator to take a baseline biopsy from the patient
- Provision of archival primary tumour sample. If an archival primary tumour sample is not available but another previously obtained sample is available, this sample should be provided instead

The following assessments should be conducted within 14 days prior to Cycle 1 Day 1:

- Triplicate ECG taken 2-5 minutes apart
- Pregnancy test and contraceptive counselling for patients of childbearing potential
- Calculated creatinine clearance using Cockroft and Gault equation (see Appendix 5)

E5.2. Cohort E Baseline Assessments Pre-Treatment Cycle 1 Day 1

The following assessments should be performed within 7 days (including Cycle 1 Day 1) prior to commencing trial treatment:

- Physical examination and vital signs
- Assessment of symptoms
- Review of concomitant medications
- Safety bloods: Haematology – full blood count, white cell count with differential and ANC, prothrombin time and INR; Biochemistry – sodium, potassium, calcium, magnesium, ALT, AST, GGT, bilirubin, albumin, creatinine, alkaline phosphatase, glucose, urea, LDH

- Urinalysis by dipstick (blood, albumin, glucose) should be performed at screening, baseline and then only if clinically indicated. *Microscopic analysis should be performed by the hospital's local laboratory if required.*
- Triplicate ECG
- Research blood samples:
  - 20ml blood sample collected in EDTA tube for plasma ctDNA analysis, to be processed within 1 hour at site
  - 10ml blood sample collected in serum tube for biomarker bloods
  - 3ml blood sample collected in EDTA tube for germline DNA

The following assessment should be performed on Cycle 1 Day 1 prior to commencing trial treatment:

- Pregnancy test for patients of childbearing potential

E5.3. Cohort E On-Treatment Assessments

E5.3.1. Cycle 1 Day 8 Pre-Treatment

- Safety bloods to be taken as close to as possible and within -/+ 1 day of Cycle 1 Day 8: Haematology – full blood count, white cell count with differential and ANC, prothrombin time and INR; Biochemistry – sodium, potassium, calcium, magnesium, ALT, AST, GGT, bilirubin, albumin, creatinine, alkaline phosphatase, glucose, urea, LDH

E5.3.2. Cycle 1 Day 15 Pre-Treatment

- Physical examination (symptom directed) and vital signs
- Assessment of symptoms including clinical assessment of disease progression
- Dosing compliance
- Review of concomitant medications
- Review of AEs
- Safety bloods to be taken within 72 hours prior to Cycle 1 Day 15: Haematology – full blood count, white cell count with differential and ANC, prothrombin time and INR; Biochemistry – sodium, potassium, calcium, magnesium, ALT, AST, GGT, bilirubin, albumin, creatinine, alkaline phosphatase, glucose, urea, LDH
- Triplicate ECG
- Research blood samples:
  - 20ml blood sample collected in EDTA tube for plasma ctDNA analysis, to be processed within 1 hour at site
  - 10ml blood sample collected in serum tube for biomarker bloods
- Research tissue sample:
  - Optional recurrent tumour biopsy (can be performed before or after trial treatment administration)

E5.3.3. Cycle 2, Day 1 Pre-Treatment

The following assessments should be performed as close to as possible and within -/+ 3 days of the due date of the visit:

- Physical examination and vital signs

- Assessment of symptoms including clinical assessment of disease progression
- Dosing compliance
- Review of concomitant medications
- Review of AEs
- Safety bloods to be taken within 72 hours prior to the start of Cycle 2: Haematology – full blood count, white cell count with differential and ANC, prothrombin time and INR; Biochemistry – sodium, potassium, calcium, magnesium, ALT, AST, GGT, bilirubin, albumin, creatinine, alkaline phosphatase, glucose, urea, LDH
- Triplicate ECG
- Pregnancy test for patients of childbearing potential performed within 72 hours prior to the start of Cycle 2
- Research blood samples:
  - 20ml blood sample collected in EDTA tube for plasma ctDNA analysis, to be processed within 1 hour at site
  - 10ml blood sample collected in serum tube for biomarker bloods
  -

E5.3.4. Cycle 3 Onwards, Day 1 Pre-Treatment

The following assessments should be performed as close to as possible and within -/+ 7 days of the due date of the visit:

- Physical examination and vital signs125
- Assessment of symptoms including clinical assessment of disease progression
- Dosing compliance
- Review of concomitant medications
- Review of AEs
- Safety bloods to be taken within 72 hours prior to the start of each cycle: Haematology – full blood count, white cell count with differential and ANC, prothrombin time and INR; Biochemistry – sodium, potassium, calcium, magnesium, ALT, AST, GGT, bilirubin, albumin, creatinine, alkaline phosphatase, glucose, urea, LDH
- Triplicate ECG
- Pregnancy test for patients of childbearing potential performed within 7 days prior to the start of each cycle
- CT and/or MRI scan every 8 weeks from Cycle 1 Day 1 until 32 weeks, then every 12 weeks thereafter
- RECIST v1.1 assessment every 8 weeks from Cycle 1 Day 1 until 32 weeks, then every 12 weeks thereafter
- Bone scan only if clinically indicated
- Research blood sample:
  - 20ml blood sample collected in EDTA tube for plasma ctDNA analysis, to be processed within 1 hour at site
  -

E5.4. Cohort E End of Treatment Assessments

E5.4.1. Treatment Discontinuation Visit

The following assessments should be performed at the time of discontinuation of trial treatment for any reason:

- ECOG performance status
- Physical examination and vital signs
- Assessment of symptoms including clinical assessment of disease progression
- Dosing compliance
- Review of concomitant medications
- Review of AEs
- Safety bloods: Haematology – full blood count, white cell count with differential and ANC, prothrombin time and INR; Biochemistry – sodium, potassium, calcium, magnesium, ALT, AST, GGT, bilirubin, albumin, creatinine, alkaline phosphatase, glucose, urea, LDH
- Triplicate ECG
- Research blood sample:
    - 20ml blood sample collected in EDTA tube for plasma ctDNA analysis, to be processed within 1 hour at site

E5.4.2. End of Trial Treatment Visit 30 days after the Last Administration of Trial Treatment

The following assessments should be performed at the end of trial treatment visit, 30 days after the last administration of trial treatment:

- ECOG performance status
- Physical examination and vital signs
- Assessment of symptoms including clinical assessment of disease progression
- Review of concomitant medications
- Review of AEs
- Safety bloods: Haematology – full blood count, white cell count with differential and ANC, prothrombin time and INR; Biochemistry – sodium, potassium, calcium, magnesium, ALT, AST, GGT, bilirubin, albumin, creatinine, alkaline phosphatase, glucose, urea, LDH
- Triplicate ECG
- Pregnancy test for patients of childbearing potential
- Research tissue sample:
    - Optional recurrent tumour biopsy

E5.5. Cohort E Post-Treatment Follow up

All patients should be followed up at 6 monthly intervals from the end of trial treatment until death or up to 2 years; assessment should be in line with standard practice and should include:

- Survival
- Further treatment

E5.6. Cohort E Discontinuation from Treatment

Patients may discontinue from trial treatment at any time at their own request, or they may be discontinued at the discretion of the PI. Discontinuation of trial treatment for any reason requires

discontinuation of both olaparib and AZD6738. Protocol-specified reasons for discontinuation will include:

- Disease progression or recurrence
- Unacceptable toxicity
- Bone marrow findings consistent with myelodysplastic syndrome (MDS)/acute myeloid leukaemia (AML)
- Pregnancy
- Withdrawal of consent
- Serious non-attendance and/or persistent non-compliance with procedures defined in the trial protocol

Patients who discontinue trial treatment in the absence of disease progression should continue to have CT and/or MRI scans to assess disease status according to the trial assessment schedule. All patients who discontinue treatment should continue to be followed up. Patients will be asked for consent for future linkage with routinely collected health data (via national registries) to trace their eventual vital status and assess subsequent unexpected co-morbidities.

E5.7. Cohort E Discontinuation from Follow up

If a patient wishes to withdraw from further follow up, a Patient Withdrawal Form should be submitted to ICR-CTSU within the clinical trial database stating whether the patient has withdrawn consent for further information to be sent to the ICR-CTSU or whether they simply no longer wish to attend trial follow up visits.

In the very rare event that a patient requests that their data is removed from the trial entirely, the implications of this should be discussed with the patient first to ensure that this is their intent and, if confirmed, ICR-CTSU should be notified in writing. *The patient should be made aware that any information about them that has already been published or submitted to the authorities for safety monitoring purposes cannot be withdrawn.*

Should a patient withdraw consent for their samples to be used in plasmaMATCH, following receipt of written confirmation from the site to ICR-CTSU, blood samples will be destroyed and biopsy blocks returned to the site for archiving where requested or otherwise destroyed.

E5.8. Cohort E Schedule of Assessments

**Procedures and assessments**	**Screening for entry into treatment cohort**		**Cycle 1 (Baseline and Week 2)**			**Cycle 2 (Week 4)**	**Cycle 3 (Week 8)**	**Cycle 4 onwards (Week 12, and 4 weekly thereafter until progression)**	**End of treatment visits**		**Follow up**
	**Day -28 to Day -1**	**Day -14 to Day -1**	**Day 1[1]**	**Day 8[2]**	**Day 15**	**Day 1[3]**	**Day 1[4]**	**Day 1[4]**	**Treatment discontinuation**	**Day 30 after last dose of drug**	**6 monthly from end of treatment until death or up to 2 years**
Olaparib			Olaparib taken continuously for each 28 day treatment cycle								
AZD6738			AZD6738 taken Days 1-7 of each 28 day treatment cycle								
Informed consent	X										
Medical history	X										
ECOG performance status	X								X	X	
Obtain archival primary tissue[5]	X										
Recurrent tumour biopsy[6,7]	X										
**PLEASE NOTE, ASSESSMENTS SHOULD BE COMPELTED PRE-TREATMENT UNLESS OTHERWISE STATED**											
Recurrent tumour biopsy (optional)[7]					X					X	
Physical examination and vital signs	X		X		X	X	X	X	X	X	
Assessment of symptoms (including clinical evidence of disease progression)	X		X		X	X	X	X	X	X	
Dosing compliance					X	X	X	X	X		
Concomitant medications	X		X		X	X	X	X	X	X	
Adverse events					X	X	X	X	X	X[8]	
12 Lead ECG Triplicate		X	X		X	X	X	X	X	X	
Pregnancy test[9]		X	X			X	X	X		X	
Safety bloods[10]	X		X[10]	X[10]	X[10]	X[10]	X[10]	X[10]	X	X	

Continued overleaf

**Procedures and assessments**	**Screening for entry into treatment cohort**		**Cycle 1 (Baseline and Week 2)**			**Cycle 2 (Week 4)**	**Cycle 3 (Week 8)**	**Cycle 4 onwards (Week 12, and 4 weekly thereafter until progression)**	**End of treatment visits**		**Follow up**
	**Day -28 to Day -1**	**Day -14 to Day -1**	**Day 1[1]**	**Day 8[2]**	**Day 15**	**Day 1[3]**	**Day 1[4]**	**Day 1[4]**	**Treatment discontinuation**	**Day 30 after last dose of drug**	**6 monthly from end of treatment until death or up to 2 years**
Urinalysis by dipstick[11]	X		X			(X) if clinically indicated	(X) if clinically indicated	(X) if clinically indicated			
Calculated creatinine clearance using Cockroft and Gault equation (Appendix 5)		X	X[12]	X[12]	X[12]	X[12]	X[12]	X[12]			
Biomarker bloods			X		X	X					
Plasma for ctDNA			X		X	X	X	X	X		
Whole blood for germline DNA			X								
Bone scan	X						(X) if clinically indicated	(X) if clinically indicated			
CT and/or MRI scan	X						X	X 8 weekly from Cycle 1 Day 1 until 32 weeks then every 12 weeks[13] or if clinically indicated			
Disease progression assessment (according to RECIST v1.1)	X						X	X 8 weekly from Cycle 1 Day 1 until 32 weeks then every 12 weeks			
Survival and further treatment											X

[1] Cycle 1 Day 1: Baseline assessments should be performed within 7 days (including Cycle 1 Day 1) prior to commencing trial treatment with the exception of the pregnancy test in patients of childbearing potential, which must be performed on Cycle 1 Day 1 prior to commencing trial treatment.

[2] Cycle 1 Day 8: Safety bloods should be taken -1/+1 day of Cycle 1 Day 8.

[3] Cycle 2 Day 1: Assessments should be performed as close to as possible and within -/+ 3 days of the due date of the visit; trial treatment should be administered as per the above schedule, however if not possible due to unavoidable circumstances treatment may be administered 1 day prior to or delayed for up to 3 days after the due date.

[4] Cycle 3 Day 1 onwards: Assessments should be performed as close to as possible and within -/+ 7 days of the due date of the visit; trial treatment should be administered as per the above schedule, however if not possible due to unavoidable circumstances treatment may be administered 1 day prior to or delayed for up to 7 days after the due date.

[5] Provision of archival primary tumour sample. If an archival primary tumour sample is not available but another previously obtained sample is available, this sample should be provided instead.

[6] Patients are requested to consent to a baseline biopsy, however if deemed unsafe by the Investigator, an archival biopsy of recurrent disease can be used instead.

[7] Recurrent tumour core biopsies can be ultrasound assisted or CT scan guided dependent on metastatic site and according to local practice.

[8] SAEs should be followed up until resolution.

[9] Patients of childbearing potential should have a negative serum or urine pregnancy test within 14 days prior to initiation of trial treatment, on Day 1 of treatment Cycle 1, and then within 72 hours prior to Cycle 2 Day 1 and within 7 days prior to the start of each cycle from Cycle 3 onwards, and at Day 30 after last dose of trial treatment.

[10] Safety bloods should be taken within 7 days prior to Cycle 1 Day 1, -1/+1 day of Cycle 1 Day 8 and then within 72 hours prior to Cycle 1 Day 15 and the start of each cycle from Cycle 2 onwards: Haematology – full blood count, white cell count with differential and ANC, prothrombin time and INR; Biochemistry – sodium, potassium, calcium, magnesium, ALT, AST, bilirubin, GGT, albumin, creatinine, alkaline phosphatase, glucose, urea, LDH.

[11] Urinalysis (blood, albumin, glucose) by dipstick should be performed at screening, baseline and then only if clinically indicated. *Microscopic analysis should be performed by the hospital's local laboratory if required.*

[12] If creatinine increases to Grade ≥2 then calculated creatinine clearance should be performed.

[13] Patients who experience treatment cycle delays should continue to have CT and/or MRI scans every 8 weeks from Cycle 1 Day 1 until 32 weeks, and then every 12 weeks thereafter.

Please note, all blood and tissue samples should be collected according to the instructions provided in the plasmaMATCH Investigator Laboratory Manual.

E6. COHORT E TRIAL TREATMENT

Olaparib and AZD6738 are IMPs within plasmaMATCH Cohort E.

E6.1. Cohort E Dose and Schedule

Olaparib 300mg twice daily should be administered orally on each day of the treatment cycle and AZD6738 160mg once daily should be administered orally on Days 1–7 of each 28 day treatment cycle.

Olaparib should be taken at the same time each day, approximately 12 hours apart with one glass of water. When AZD6738 is administered in combination with olaparib, patients must fast for at least 2 hours prior to taking trial treatment and for at least 1 hour after taking trial treatment. Trial treatment should be swallowed whole and not chewed, crushed, dissolved or divided. The scheduled dose of trial treatment can be taken up to 2 hours after the scheduled dose time. If greater than 2 hours has passed, the missed dose should not be taken and patient should continue with next dose at allotted time.

When olaparib is given on its own, olaparib can be taken without regard to food.

It is recommended that patients avoid the consumption of grapefruit juice grapefruit hybrids, pummelos, star-fruit, Seville oranges or products containing the juice of each (such as marmalade) during the entire study and preferably 7 days before the first dose of study medication.

Trial treatment should be administered as per the above schedule, however if this is not possible due to unavoidable circumstances (i.e. bank holidays) from Cycle 2 onwards Day 1 of trial treatment may be delayed for up to 3 days after the due date for Cycle 2, delayed for up to 7 days after the due date for Cycle 3 onwards.

For further guidance on trial treatment delay due to toxicity please refer to Sections E6.8 and E6.9.

E6.2. Cohort E Prescription and Dispensing

Olaparib and AZD6738 will be provided in non-patient-specific bottles. The patient's Trial ID should be recorded on the bottle label prior to dispensing. Patients should be instructed to keep their medication in the bottles provided and not transfer it to any other container.

All efforts should be made to ensure that patients clearly understand the directions for self-medication. Patients should be given a sufficient supply and unused drug and/or empty bottles should be returned at the appropriate time points. Returned unused medication must not be re-dispensed to any patient and should only be destroyed with prior approval from ICR-CTSU according to local destruction policy.

Olaparib and AZD6738 should be prescribed by the PI or Co-investigator and dispensed by the hospital pharmacy from trial stock supplied specifically for use within plasmaMATCH only.

Patients should be instructed not to consume grapefruit juice while receiving olaparib.

E6.3. Cohort E Patient Cards and Treatment Diary Cards

A small wallet sized patient card template will be provided by ICR-CTSU for completion by the participating site. Each card will state:

- The name of the participating site
- That the patient is participating in the plasmaMATCH trial
- That the patient is taking olaparib and AZD6738
- An emergency site contact number

Patients should be advised to keep their patient card in their possession at all times.

A treatment diary card will be provided by ICR-CTSU for completion by the patient, in order to record the number of tablets taken on each day of the treatment cycle.

E6.4. Cohort E Duration of Treatment

Patients will remain on treatment until progression. Patients may withdraw from trial treatment early if they experience unacceptable toxicity or if the treating clinician believes further treatment is no longer appropriate or at the patient's request.

E6.5. Cohort E Permitted Concomitant Therapy

All medication considered necessary for the patients' welfare and which is not expected to interfere with the evaluation of the trial drugs may be given at the discretion of the Investigator. All concomitant medications must be recorded in the patient's notes, as well as the appropriate section of the plasmaMATCH eCRF.

Bisphosphonates, RANK ligand antagonists and blood transfusions should be given as required at the discretion of the Investigator.

Palliative radiotherapy is acceptable if given for bony metastases as long as these are not indicative of disease progression. Olaparib and AZD6378 must be stopped at least 3 days before radiotherapy and should be restarted within 28 days provided any bone marrow toxicity has recovered. Olaparib and AZD6738 should be stopped at least 3 days prior to planned surgery. After surgery trial treatment should be restarted within 28 days provided the wound has healed. No interruption to trial treatment is required for any needle biopsy procedure.

Patients may take corticosteroids but increased vigilance is recommended on electrolyte and glucose levels.

Patients who require anti-coagulation during trial treatment should start on LMWH.

E6.6. Cohort E Non-permissible Medications/Therapies

Investigational agents and other anticancer agents must not be given while the patient is on trial treatment. Live virus and live bacterial vaccines should not be administered whilst the patient is receiving trial treatment and during the 30 day follow up period. An increased risk of infection by the

administration of live virus and bacterial vaccines has been observed with conventional chemotherapy drugs and the effects with olaparib are unknown.

PLEASE REFER TO APPENDICES 8 (OLAPARIB) AND 9 (AZD6738) FOR PROHIBITED CONCURRENT MEDICATIONS.

OLAPARIB

It is recommended that known potent inhibitors/inducers of CYP3A are not co-administered with olaparib. Please see Appendix 8 for details. The required washout period prior to starting study treatment is 2 weeks for inhibitors, 3 weeks for inducers, and 5 weeks for enzalutamide or phenobarbital.

Caution should be exercised when substrates of CYP3A4 or UGT1A1 are combined with olaparib, in particular those with a narrow therapeutic margin. Caution should be exercise if olaparib is administered in combination with P-gp inhibitors which may increase exposure to olaparib. Caution should be exercised if olaparib is administered in combination with any statin. Please see Appendix 8 for details.

Based on limited *in vitro* data, olaparib may increase the exposure to substrates of CYP3A4, P-gp, OATP1B1, OCT1, OCT2, OAT3, MATE1 and MATE2K. Based on limited *in vitro* data, olaparib may reduce the exposure to substrates of CYP3A4, CYP1A2, 2B6, 2C9, 2C19 and P-gp. Please see Appendix 8 for examples of substrates.

The efficacy of hormonal contraceptives may be reduced if co-administered with olaparib.

AZD6738

AZD6738 is an investigational drug for which no data on *in vivo* interactions are currently available. Potential interaction is considered on the basis of preclinical *in vitro* data only.

The lists of CYP and transporter inhibitors/inducers, and CYP and transporter substrates are available in Appendix 9. They are not exhaustive and the absence of a drug from these lists does not imply that its combination with AZD6738 is safe.

The principal enzyme for metabolizing AZD6738 is CYP3A. Patients should avoid concomitant drugs, herbal supplements and/or ingestion of foods known to modulate CYP3A activity from the time they enter the screening period until 28 days after the last dose of study treatment.

For patients taking potent inducers or inhibitors of CYP3A (examples provided in Appendix 9) the required washout period before starting AZD6738 is five half-lives; except for St. John’s wort, which is 3 weeks.

If there is no suitable alternative concomitant medication other than a potent inhibitor of CYP3A, the Investigator must interrupt AZD6738 for the duration of the potent CYP3A inhibitor and wait for the required washout period (five half-lives) before dosing AZD6738 again. If potent CYP3A inducers are considered necessary for the patient’s safety and welfare, this may diminish the clinical efficacy of

AZD6738 and the patient should be monitored carefully for any change in the efficacy of study treatment. Please refer to Appendix 9 for additional guidance.

AZD6738 is a P-gp substrate. Co-administration of P-gp inhibitors or inducers may affect exposure to AZD6738 and, therefore, should not be co-administered with AZD6738. If the use of any inhibitors or inducers of P-gp are considered necessary for the patient's safety and welfare, the investigator must interrupt AZD6738 for the duration of the P-gp inhibitor or inducer and wait for the required washout period of the P-gp modulator (five half-lives) before dosing AZD6738 again. Please refer to Appendix 9 for additional guidance.

AZD6738 is also a substrate of BCRP. Co-administration of BCRP inhibitors or inducers may affect exposure to AZD6738; therefore, it is recommended that the investigators must interrupt AZD6738 for the duration of the BCRP inhibitor or inducer and wait for the required washout period of the BRCP modulator (five half-lives) before dosing AZD6738 again. Please refer to Appendix 9 for additional guidance.

AZD6738 is a potential inducer of CYP3A4 and CYP2B6. Caution should be applied with co-administration of drugs that are either completely metabolized by CYP3A4 and/or CYP2B6, or that are substrates of CYP3A4 and/or CYP2B6 and also have a narrow therapeutic index. Investigators should be aware that the exposure of other drugs metabolised by CYP3A4 and/or CYP2B6 may be reduced. Please refer to Appendix 9 for additional guidance.

AZD6738 is an inhibitor of OATP1B1 and BCRP. Caution should be applied with co-administration of substrates of OATP1B1 and/or BCRP as AZD6738 may increase their exposure. Please refer to Appendix 9 for additional guidance.

The use of herbal supplements or 'folk remedies' (and medications and foods that significantly modulate CYP3A activity) should be discouraged. If deemed necessary, such products may be administered with caution and the reason for use documented in the CRF.

E6.7. Cohort E Additional Cautions

Because the AEs related to olaparib may include asthenia, fatigue and dizziness, patients should be advised to use caution while driving or using machinery if these symptoms occur.

Phototoxicity is not designated an important risk, but AZD6738 is an inhibitor of DNA damage repair it is probably reasonable to advise patients to avoid excessive sun exposure while receiving AZD6738.

E6.8. Cohort E Dose Modifications

Every effort should be made to administer olaparib and AZD6738 at the planned dose and schedule. Any toxicity observed during the course of the trial treatment should be managed by interruption of olaparib and AZD6738, as deemed appropriate by the Investigator. Please see Section E6.9 for further guidance on dose interruptions. However, patients experiencing toxicities related to the trial treatment may have their dose modified as outlined in this section. Further guidance on specific toxicities requiring dose modifications are described in Section E6.8.1.

Any dose reductions should be applied to subsequent cycles unless a further dose reduction is required. Once a reduction is made the patient should not increase back to a higher dose level.

Table E1. Cohort E olaparib and AZD6738 dose reductions guidelines

Dose Level	Olaparib	AZD6738
**Starting dose**	300mg BD Days 1-28	160mg OD Days 1-7
**First dose reduction**		
For haematological toxicity	250mg BD Days 1-28	160mg OD Days 1-4
For non-haematological toxicity	250mg BD Days 1-28	120mg OD Days 1-7
**Second dose reduction**	200mg BD Days 1-28	120mg OD Days 1-4
**Third dose reduction**	Discontinue trial treatment	

The haematological criteria for initiation of a treatment cycle are blood counts on Day 1 of any cycle (within 72 hours pre-treatment):

- ANC ≥1500/mm^3 (≥1.5 x 10^9/L)
- Platelet count ≥75,000/mm^3 (≥75x 10^9/L)
- Haemoglobin ≥8g/dL (≥80g/L) (with the exception of Cycle 1 where Hb ≥10)

The haematological criteria for continuation of treatment on Cycle 1 Day 8 (-/+ 1 day) and Cycle 1 Day 15 (within 72 hours pre-treatment):

- ANC ≥1000/mm^3 (≥1.0 x 10^9/L)
- Platelet count ≥50,000/mm^3 (≥50 x 10^9/L)
- Haemoglobin ≥8g/dL (≥80g/L)

E6.8.1. Specific Toxicities Requiring Olaparib and AZD6738 Dose Modifications

Haematological toxicity

Table E2. Cohort E haematological toxicity management guidelines

Toxicity	Action
**Any Grade 3 haematological toxicity**	<u>**First occurrence**</u><br>• Withhold dose for up to 28 days until recovery to Grade ≤1 then resume both olaparib and AZD6738 at the same dose level.<br>• If symptoms do not recover to Grade ≤1 within 28 days, discontinue both olaparib and AZD6738.<br><br><u>**Second occurrence**</u><br>• Withhold dose for up to 28 days until recovery to Grade ≤1 then reduce olaparib and AZD6738 to the first dose reduction level for haematological toxicity.<br>• If symptoms do not recover within 28 days to Grade ≤1, discontinue both olaparib and AZD6738.<br><br><u>**Third occurrence**</u><br>• Withhold dose for up to 28 days until recovery to Grade ≤1 then reduce

Toxicity	Action
	both olaparib and AZD6738 to the second dose reduction level for haematological toxicity.<br>• If symptoms do not recover within 28 days to Grade ≤1, discontinue both olaparib and AZD6738.<br><br>**<u>Fourth occurrence</u>**<br>• Discontinue both olaparib and AZD6738.
**Any Grade 4 haematological toxicity**	**<u>First occurrence</u>**<br>• Withhold dose for up to 28 days until recovery to Grade ≤1 then reduce olaparib and AZD6738 to the first dose reduction level for haematological toxicity.<br>• If symptoms do not recover to Grade ≤1, discontinue both olaparib and AZD6738.<br><br>**<u>Second occurrence</u>**<br>• Withhold dose for up to 28 days until recovery to Grade ≤1 then reduce both olaparib and AZD6738 to the second dose reduction level for haematological toxicity.<br>• If symptoms do not recover to Grade ≤1, discontinue both olaparib and AZD6738.<br><br>**<u>Third occurrence</u>**<br>• Discontinue both olaparib and AZD6738.
**Anaemia**	• Common treatable causes of anaemia (e.g. iron, vitamin B12 or folate deficiencies and hypothyroidism) should be investigated and appropriately managed. In some cases management of anaemia may require blood transfusions. The use of erythropoietin is not allowed at any stage.
Haemoglobin (Hb) <10 but ≥8g/dL<br>(Grade 2)	General management<br>• Give appropriate supportive treatment and investigate causality.<br>• Investigator judgement to continue trial treatment with supportive treatment (e.g. transfusion) or interrupt dose for a maximum of 28 days, for investigations and supportive treatment.<br>• Before initiation of any treatment cycle (Day 1) the Hb must be >8g/dL (80g/L) (with the exception of Cycle 1, where Hb must be >10g/dL (100g/L).<br><br>**<u>First occurrence</u>**<br>• General management as above, and resume both olaparib and AZD6738 at the same dose level.<br><br>**<u>Second occurrence</u>**<br>• General management as above then reduce both olaparib and AZD6738 to the first dose reduction level for haematological toxicity.<br><br>**<u>Third occurrence</u>**<br>• General management as above then reduce both olaparib and AZD6738 to the second dose reduction level for haematological toxicity.<br><br>**<u>Fourth occurrence</u>**<br>• Discontinue both olaparib and AZD6738.
Hb <8g/dL<br>(Grade ≥3)	**<u>General management</u>**<br>• Appropriate supportive treatment should be given (e.g. transfusion) and causality investigated.<br>• Interrupt olaparib and AZD6738 until Hb≥8g/dL (i.e., Grade ≤2).

Toxicity	Action
	**First occurrence**<br>• General management as above, and reduce both olaparib and AZD6738 to the first dose reduction level for haematological toxicity.<br><br>**Second occurrence**<br>• General management as above, and reduce both olaparib and AZD6738 to the second dose reduction level for haematological toxicity.<br><br>**Third occurrence**<br>• Discontinue both olaparib and AZD6738.
**Neutropenia, leukopenia and thrombocytopenia**	• Neutropenia, leukopenia and thrombocytopenia should be managed as deemed appropriate by the Investigator with close follow up.<br>• For toxicity of Grades 1-2, appropriate supportive treatment should be given and causality investigated. Trial treatment can be interrupted for a maximum of 28 days at the Investigator's discretion.<br>• If Grade ≥3 neutropenia occurs interrupt trial treatment and follow the instructions for any haematological toxicity above as appropriate.<br>• Primary prophylaxis with granulocyte colony-stimulating factor (G-CSF) is not recommended, however, if a patient develops febrile neutropenia, trial treatment should be interrupted for a maximum of 28 days and appropriate management including G-CSF should be given according to local hospital guidelines. Please note that G-CSF should not be used within at least 24 hours (7 days for pegylated G-CSF) of the last dose of trial treatment unless absolutely necessary.<br>• Platelet transfusions, if indicated, should be done according to local hospital guidelines.
**Prolonged haematological toxicity such as ≥2 week interruption/delay in trial treatment due to Grade ≥3:**<br>• Anaemia and/or development of blood transfusion dependence<br>• Neutropenia (ANC <1 x 10^9/L)<br>• Thrombocytopenia and/or development of platelet transfusion dependence (platelet count <50 x 10^9/L)	• Check weekly differential blood counts (including reticulocytes and peripheral blood smear).<br>• If any blood parameters remain clinically abnormal after 28 days of dose interruption, the patient should be referred to a haematologist for further investigations. Bone marrow analysis and/or blood cytogenetic analysis should be considered at this stage according to standard local haematological practice. Bone marrow analysis should include an aspirate for cellular morphology, cytogenetic analysis and flow cytometry, and a core biopsy for bone marrow cellularity. If it is not possible to conduct cytogenetic analysis or flow cytometry on the bone marrow aspirate, then attempts should be made to carry out the tests on a blood sample. If findings are consistent with MDS/AML, study drug should be discontinued and a full description of findings should be submitted in an SAE report.<br>• Trial treatment should be discontinued if blood counts do not recover to Grade ≤1 within 28 days of dose interruption.<br>• Development of a confirmed MDS or other clonal blood disorder should be reported as an SAE, please refer to Section 12.<br>• Trial treatment should be discontinued if the diagnosis of MDS and/or AML is confirmed.

Non-haematological toxicity

Table E3. Cohort E non-haematological toxicity management guidelines

Toxicity	Action
**Any non-haematological toxicity Grade ≥3**	**First occurrence**<br>• Withhold dose for up to 28 days until recovery to Grade ≤1. For Grade 3

Toxicity	Action
	toxicity both olaparib and AZD6738 may be resumed at the same dose level at the discretion of the Investigator. In the case of Grade 4 toxicity both olaparib and AZD6738 should be reduced to the first dose reduction level for non-haematological toxicity.<br>• If symptoms do not recover to Grade ≤1 (or recover to baseline level if baseline was Grade 2) within 28 days, discontinue both olaparib and AZD6738. Patients with alopecia may continue on trial treatment.<br><br>**Second occurrence**<br>• Withhold dose for up to 28 days until recovery to Grade ≤1 then reduce both olaparib and AZD6738 to the next dose reduction level for non-haematological toxicity.<br>• If symptoms do not recover to Grade ≤1 (or recover to baseline level if baseline was Grade 2) within 28 days discontinue both olaparib and AZD6738. Patients with alopecia may continue on therapy.<br><br>**Third occurrence**<br>• Withhold dose for up to 28 days until recovery to Grade ≤1 then reduce both olaparib and AZD6738 to the second dose reduction level for non-haematological toxicity. Patients who are currently receiving the second dose reduction level should discontinue both olaparib and AZD6738.<br>• If symptoms do not recover to Grade ≤1 within 28 days, discontinue both olaparib and AZD6738.<br><br>**Fourth occurrence**<br>• Discontinue both olaparib and AZD6738.
**New or worsening pulmonary symptoms**<br><br>**NB. Pneumonitis is an important potential risk for olaparib**	• If new or worsening pulmonary symptoms (e.g. dyspnoea) or radiological abnormalities occur in the absence of a clear diagnosis, an interruption in trial treatment dosing is recommended and further diagnostic workup (including a high resolution CT scan) should be performed to exclude pneumonitis.<br>• The following assessments, and additional assessments if required, should be performed to enhance the investigation and diagnosis of potential cases of pneumonitis:<br>o Physical examination and signs and symptoms (cough, shortness of breath and pyrexia, etc.) including auscultation for lung field.<br>o Saturation of peripheral oxygen (SpO_2)<br>o The following markers should be measured where possible: ILD markers (KL-6, SP-D) and β-D-glucan; tumour markers related to disease progression.<br>• Following investigation, if no evidence of abnormality is observed on CT imaging and symptoms resolve, then trial treatment can be restarted, if deemed appropriate by the Investigator.<br>• If significant pulmonary abnormalities are identified, these should be raised with the plasmaMATCH Trial Team in the first instance for discussion with the Chief or Coordinating Investigator.<br>• If Grade ≥2 pneumonitis is confirmed, olaparib and AZD6738 treatment should be discontinued and the patient treated appropriately.<br>• Any event of pneumonitis should be reported as an SAE, please refer to Section 12.
**Nausea and vomiting**	• Events of nausea and vomiting are known to be associated with olaparib treatment. They are generally mild to moderate (Grade ≤2) severity, intermittent and manageable on continued treatment. The first onset generally occurs in the first month of treatment with the incidence of nausea

Toxicity	Action
	and vomiting not showing an increase over the subsequent treatment cycles.<br>• If the patient vomits after taking a dose of trial treatment they should be advised to resume treatment at the next scheduled dose<br>• No routine prophylactic anti-emetic treatment is required at the start of trial treatment, however patients should receive appropriate anti-emetic treatment at the first onset of nausea or vomiting and as required thereafter, in accordance with local treatment practice guidelines.
**Diarrhoea**	• Loperamide 4mg should be administered at the first onset of diarrhoea and then 2mg every 2 hours until diarrhoea-free for at least 12 hours. The first dose of loperamide could be lowered to 2mg if the diarrhoea is recurrent and if, in the opinion of the treating physician, the diarrhoea is not severe.<br>• Patients should be instructed to notify the Investigator or research staff of the occurrence of bloody or black stools, symptoms of dehydration, fever, inability to take liquids by mouth, and inability to control diarrhoea within 24 hours of using loperamide or other prescribed anti diarrhoeal medications.<br>• If diarrhoea is severe (i.e., requiring intravenous rehydration) and/or associated with fever or severe neutropenia (Grade 3 or 4), broad-spectrum antibiotics must be prescribed. Patients with severe diarrhoea or any diarrhoea associated with severe nausea or vomiting should be hospitalised for intravenous hydration and correction of electrolyte imbalances.
**Myelodysplastic syndrome (MDS)/Acute Myeloid Leukaemia (AML)**<br><br>**NB. MDS/AML are important potential risks for olaparib**	• Olaparib and AZD6378 treatment should be discontinued if the diagnosis of MDS and/or AML is confirmed and the patient should be treated appropriately.<br>• Development of a confirmed MDS and/or AML whilst on trial treatment with olaparib or following treatment discontinuation should be reported as an SAE, please refer to Section 12.
**Cardiovascular effects**	• Treatment with olaparib and AZD6738 should be interrupted **immediately** at any occurrence of clinically significant changes in blood pressure until all abnormalities return to normal or to their baseline state.<br>• Patients with Grade 3 changes in blood pressure, or recurrent Grade 2 changes in blood pressure, should reduce both olaparib and AZD6738 to the next dose reduction level.
**Renal impairment**	• If creatinine increases to Grade ≥2, creatinine clearance should be calculated by Cockcroft and Gault equation (see Appendix 5). If calculated creatinine clearance is >51 ml/min treatment should be continued at the current dose.<br>• A dose reduction is recommended for patients who develop moderate renal impairment (calculated creatinine clearance by Cockcroft-Gault equation of between 31 and 50 ml/min) for any reason during trial treatment. The dose of olaparib should be reduced to 200mg BD.<br>• Because the creatinine clearance determination is only an estimate of renal function, in instances where the creatinine clearance falls to between 31 and 50 ml/min, the Investigator should use their discretion in determining whether a dose change or discontinuation of trial treatment is warranted.<br>• Caution should be used in patients with a creatinine clearance less than 30ml/min as safety and efficacy have not been evaluated in this population.<br>• The safety and efficacy of trial treatment has not been evaluated in patients with severe renal impairment (creatinine clearance ≤30 ml/min) or end-stage renal disease; if patients develop severe impairment or end stage disease is it recommended that all trial treatment be discontinued.
**Hepatic toxicity**	• Please refer to Table E4 for hepatic toxicity management guidelines.

Hepatic toxicity

Table E4. Cohort E hepatic toxicity management guidelines

Event	Actions
• Grade 3 ALT/AST or Grade 3 bilirubin	• Withhold olaparib and AZD6378 for up to 28 days until recovery to Grade ≤1, or Grade ≤2 in patients with Grade 2 ALT at baseline.<br>• Look for alternative causes.<br>• Reduce olaparib and AZD6738 to the next dose reduction level.<br>• If symptoms do not recover to Grade ≤1 or Grade ≤2 in patients with Grade 2 ALT at baseline within 28 days, discontinue both olaparib and AZD6738.
• Grade 4 ALT/AST or Grade 4 bilirubin	• Permanently discontinue olaparib and AZD6378.<br>• Evaluate alternative cause.
• AST or ALT >3 x ULN<br>AND<br>• Total bilirubin >2 x ULN<br>AND<br>• Alkaline phosphatase <2 x ULN	• Withhold olaparib and AZD6378.<br>• Evaluate the patient as soon as possible (within 48 hours if possible). All cases confirmed on repeat testing with no alternative explanation for abnormal liver function should be considered potential Hy's law cases.<br>• Report as an SAE.<br>• Discontinue if Hy's law confirmed.<br>• If the criteria for Hy's law are not met, follow the guidance above for Grade 3/4 ALT/AST or bilirubin.

E6.9. Cohort E Dose Interruptions

In addition to the specific toxicity management guidelines within Section E6.8.1, olaparib and AZD6378 treatment should be interrupted if patients experience any Grade ≥3 toxicity.

Following an interruption for Grade ≥3 toxicity, trial treatment should be delayed for up to 28 days until these toxicities have resolved to Grade ≤1 or returned to baseline.

Repeat dose interruptions are allowed as required, for a maximum of 28 days on each occasion. If a patient remains off trial treatment for >28 days, trial treatment should be permanently discontinued.

See Section E6.5 for details of dose interruptions for palliative radiotherapy and planned surgery.

E6.10. Cohort E Missed Doses

The scheduled trial treatment dose can be taken up to 2 hours after the scheduled dose time. If greater than 2 hours has passed, the missed dose should not be taken. If a dose is missed, trial treatment should be resumed at the next scheduled dose. Missed doses should not be made up. If the patient vomits after taking a dose of trial treatment they should be advised to resume treatment at the next scheduled dose.

E6.11. Cohort E Overdoses

There is currently no specific treatment in the event of an overdose with olaparib and possible symptoms of overdose are not established. If overdose occurs, this should be managed symptomatically. Please contact the ICR-CTSU for advice.

There is no known antidote to AZD6738. If overdose occurs, this should be managed symptomatically. Please contact the ICR-CTSU for advice.

E6.12. Cohort E Discontinuation and Subsequent Therapy

Treatment should continue until disease progression, unacceptable toxicity or withdrawal of patient consent. Discontinuation of trial treatment for any reason requires discontinuation of both olaparib and AZD6738. Patients who discontinue trial treatment in the absence of disease progression should continue to have CT and/or MRI scans to assess disease status according to the trial assessment schedule, provided that the patient has not withdrawn their consent to further trial assessments.

E6.13. Cohort E Compliance

Patients must be asked to bring all their trial medication every time they attend the clinic for the purposes of treatment compliance assessment and drug accountability. Every effort should be made to encourage patients to return the unused medication and empty bottles. The unused tablets should be collected by the Investigator/Research Nurse and counted to ascertain patient compliance, medication will then be returned to pharmacy for drug accountability. Drug destruction should only be carried out with prior approval from ICR-CTSU and according to local destruction policy.

E6.14. Cohort E Supply and Distribution of Olaparib and AZD6738

Olaparib and AZD6738 are manufactured and provided free of charge by AstraZeneca to participating sites.

No drug will be distributed to participating sites unless ICR-CTSU is satisfied that the required approvals and agreements and initiation procedures are complete.

E6.15. Cohort E Formulation, Packaging, Storage Conditions and Labelling

Olaparib will be supplied as oval film-coated tablets in HDPE bottles containing desiccant. Bottles are secured with a child-resistant closure; induction-sealed membranes provide tamper evidence. Olaparib tablets should be stored below 30°C. Tablets should be stored in the bottles provided and taken according to the instructions on the label.

AZD6738 will be supplied as tablets that should be stored below 30°C in induction sealed HDPE bottles until use.

The drug distribution company is responsible for labelling olaparib and AZD6738 in accordance with the MHRA approved plasmaMATCH label. Pharmacies may add their own hospital dispensing label to the trial drug but should not obscure the existing label on the drug packaging.

E6.16. Cohort E Pharmacy Responsibilities and Drug Accountability

Olaparib and AZD6738 supplied for the plasmaMATCH trial must not be used outside the context of the plasmaMATCH protocol. Records must be kept of all deliveries, dispensing and destruction in accordance with the plasmaMATCH Pharmacy Guidance Notes. These records may be requested by

ICR-CTSU during the trial to monitor supply and usage of stock. Account must be given of any discrepancies, and certificates of delivery and destruction must be signed and dated.

REFERENCES

1. Cancer Research UK. Cancer mortality for common cancers. http://www.cancerresearchuk.org/health-professional/cancer-statistics/statistics-by-cancer-type/breast-cancer
2. Dawood S, Broglio K, Buzdar AU, Hortobagyi GN, Giordano SH. Prognosis of Women With Metastatic Breast Cancer by HER2 Status and Trastuzumab Treatment: An Institutional-Based Review. Journal of Clinical Oncology. 2010;28(1):92-8.
3. André F, Bachelot T, Commo F, Campone M, Arnedos M, Dieras V, et al. Comparative genomic hybridisation array and DNA sequencing to direct treatment of metastatic breast cancer: a multicentre, prospective trial (SAFIR01/UNICANCER). The Lancet Oncology. 2014;15(3):267-74.
4. Curtis C, Shah SP, Chin S-F, Turashvili G, Rueda OM, Dunning MJ, et al. The genomic and transcriptomic architecture of 2,000 breast tumours reveals novel subgroups. Nature. 2012;486(7403):346-52.
5. Shah SP, Roth A, Goya R, Oloumi A, Ha G, Zhao Y, et al. The clonal and mutational evolution spectrum of primary triple-negative breast cancers. Nature. 2012;486(7403):395-9.
6. Stephens PJ, Tarpey PS, Davies H, Van Loo P, Greenman C, Wedge DC, et al. The landscape of cancer genes and mutational processes in breast cancer. Nature. 2012;486(7403):400-4.
7. Toy W, Shen Y, Won H, Green B, Sakr RA, Will M, et al. ESR1 ligand-binding domain mutations in hormone-resistant breast cancer. Nat Genet. 2013;45(12):1439-45.
8. Wagle N, Lin NU, Richardson AL, Leshchiner I, Mayer IA, Forero-Torres A, et al. Whole-exome sequencing (WES) of HER2+ metastatic breast cancer (MBC) from patients (pts) treated with prior trastuzumab (T): A correlative analysis of TBCRC003. J Clin Oncol. 2014;32:5s((suppl; abstr 536)).
9. Thompson A, Jordan L, Quinlan P, Anderson E, Skene A, Dewar J, et al. Prospective comparison of switches in biomarker status between primary and recurrent breast cancer: the Breast Recurrence In Tissues Study (BRITS). Breast Cancer Research. 2010;12(6):R92.
10. Bettegowda C, Sausen M, Leary RJ, Kinde I, Wang Y, Agrawal N, et al. Detection of Circulating Tumor DNA in Early- and Late-Stage Human Malignancies. Science Translational Medicine. 2014;6(224):224ra24.
11. Dawson S-J, Tsui DWY, Murtaza M, Biggs H, Rueda OM, Chin S-F, et al. Analysis of Circulating Tumor DNA to Monitor Metastatic Breast Cancer. New England Journal of Medicine. 2013;368(13):1199-209.
12. Turner NC, Garcia-Murillas I, Schiavon G, Hrebien S, Osin P, Nerurkar A, et al. Tracking tumor-specific mutations in circulating-free DNA to predict early relapse after treatment of primary breast cancer. J Clin Oncol. 2014;32:5s((suppl; abstr 511)).
13. Thierry AR, Mouliere F, El Messaoudi S, Mollevi C, Lopez-Crapez E, Rolet F, et al. Clinical validation of the detection of KRAS and BRAF mutations from circulating tumor DNA. Nat Med. 2014;20(4):430-5.
14. Higgins MJ, Jelovac D, Barnathan E, Blair B, Slater S, Powers P, et al. Detection of Tumor PIK3CA Status in Metastatic Breast Cancer Using Peripheral Blood. Clinical Cancer Research. 2012;18(12):3462-9.
15. Forshew T, Murtaza M, Parkinson C, Gale D, Tsui DWY, Kaper F, et al. Noninvasive Identification and Monitoring of Cancer Mutations by Targeted Deep Sequencing of Plasma DNA. Science Translational Medicine. 2012;4(136):136ra68.
16. Gerlinger M, Rowan AJ, Horswell S, Larkin J, Endesfelder D, Gronroos E, et al. Intratumor heterogeneity and branched evolution revealed by multiregion sequencing. The New England journal of medicine. 2012;366(10):883-92.
17. Mok TS, Wu Y-L, Thongprasert S, Yang C-H, Chu D-T, Saijo N, et al. Gefitinib or Carboplatin–Paclitaxel in Pulmonary Adenocarcinoma. New England Journal of Medicine. 2009;361(10):947-57.
18. Wils J, Bliss JM, Coombes R, Amadori D, Fountzilas G, Klein H, et al., editors. Phase I-II study of sequential high dose methotrexate (MTX) and 5-fluorouracil (F) combined with epirubicin (E) (FEMTX) in advanced gastric cancer. Proceeds of the European Society of Medical Oncology; 1995.

19. Douillard JY, Ostoros G, Cobo M, Ciuleanu T, McCormack R, Webster A, et al. First-line gefitinib in Caucasian EGFR mutation-positive NSCLC patients: a phase-IV, open-label, single-arm study. British journal of cancer. 2014;110(1):55-62.
20. Fribbens C, Garcia Murillas I, Beaney M, Hrebien S, O'Leary B, Kilburn L, et al. Tracking evolution of aromatase inhibitor resistance with circulating tumour DNA analysis in metastatic breast cancer. Annals of oncology : official journal of the European Society for Medical Oncology. 2018;29(1):145-53.
21. Jeselsohn R, Yelensky R, Buchwalter G, Frampton G, Meric-Bernstam F, Gonzalez-Angulo AM, et al. Emergence of constitutively active estrogen receptor-alpha mutations in pretreated advanced estrogen receptor-positive breast cancer. Clin Cancer Res. 2014;20(7):1757-67.
22. Li S, Shen D, Shao J, Crowder R, Liu W, Prat A, et al. Endocrine-therapy-resistant ESR1 variants revealed by genomic characterization of breast-cancer-derived xenografts. Cell Rep. 2013;4(6):1116-30.
23. Howell A, Robertson JFR, Quaresma Albano J, Aschermannova A, Mauriac L, Kleeberg UR, et al. Fulvestrant, Formerly ICI 182,780, Is as Effective as Anastrozole in Postmenopausal Women With Advanced Breast Cancer Progressing After Prior Endocrine Treatment. Journal of Clinical Oncology. 2002;20(16):3396-403.
24. Osborne CK, Pippen J, Jones SE, Parker LM, Ellis M, Come S, et al. Double-Blind, Randomized Trial Comparing the Efficacy and Tolerability of Fulvestrant Versus Anastrozole in Postmenopausal Women With Advanced Breast Cancer Progressing on Prior Endocrine Therapy: Results of a North American Trial. Journal of Clinical Oncology. 2002;20(16):3386-95.
25. Leo AD, Jerusalem G, Petruzelka L, Torres R, Bondarenko IN, Khasanov R, et al. Final Overall Survival: Fulvestrant 500mg vs 250mg in the Randomized CONFIRM Trial. Journal of the National Cancer Institute. 2014;106(1).
26. Robertson JF, Llombart-Cussac A, Rolski J, Feltl D, Dewar J, Macpherson E, et al. Activity of fulvestrant 500 mg versus anastrozole 1 mg as first-line treatment for advanced breast cancer: results from the FIRST study. J Clin Oncol. 2009;27(27):4530-5.
27. Howell A, Osborne CK, Morris C, Wakeling AE. ICI 182,780 (Faslodex™). Cancer. 2000;89(4):817-25.
28. Bose R, Kavuri SM, Searleman AC, Shen W, Shen D, Koboldt DC, et al. Activating HER2 Mutations in HER2 Gene Amplification Negative Breast Cancer. Cancer Discovery. 2013;3(2):224-37.
29. Rabindran SK, Discafani CM, Rosfjord EC, Baxter M, Floyd MB, Golas J, et al. Antitumor activity of HKI-272, an orally active, irreversible inhibitor of the HER-2 tyrosine kinase. Cancer research. 2004;64(11):3958-65.
30. Burstein HJ, Sun Y, Dirix LY, Jiang Z, Paridaens R, Tan AR, et al. Neratinib, an irreversible ErbB receptor tyrosine kinase inhibitor, in patients with advanced ErbB2-positive breast cancer. J Clin Oncol. 2010;28(8):1301-7.
31. Chan A, Delaloge S, Holmes FA, Moy B, Iwata H, Harvey VJ, et al. Neratinib after adjuvant chemotherapy and trastuzumab in HER2-positive early breast cancer: Primary analysis at 2 years of a phase 3, randomized, placebo-controlled trial (ExteNET). J Clin Oncol 33, 2015 (suppl; abstr 508). 2015.
32. Hyman D. Neratinib for ERBB2 mutant, HER2 non-amplified, metastatic breast cancer: preliminary analysis from SUMMIT – a multicenter, open-label, multi-histology phase II basket trial. San Antonio Breast Cancer Symposium. 2015.
33. Ben-Baruch NE, Bose R, Kavuri SM, Ma CX, Ellis MJ. HER2-Mutated Breast Cancer Responds to Treatment With Single-Agent Neratinib, a Second-Generation HER2/EGFR Tyrosine Kinase Inhibitor. Journal of the National Comprehensive Cancer Network : JNCCN. 2015;13(9):1061-4.
34. Ellis M. Genome-Directed Therapeutics for Endocrine Therapy Resistant ER+ Breast Cancer San Antonio Breast Cancer Symposium. 2014.

35. Wong KK, Fracasso PM, Bukowski RM, Lynch TJ, Munster PN, Shapiro GI, et al. A phase I study with neratinib (HKI-272), an irreversible pan ErbB receptor tyrosine kinase inhibitor, in patients with solid tumors. Clin Cancer Res. 2009;15(7):2552-8.
36. Dowsett M, Harper-Wynne C, Boeddinghaus I, Salter J, Hills M, Dixon M, et al. HER-2 amplification impedes the antiproliferative effects of hormone therapy in estrogen receptor-positive primary breast cancer. Cancer research. 2001;61(23):8452-8.
37. Emde A, Mahlknecht G, Maslak K, Ribba B, Sela M, Possinger K, et al. Simultaneous Inhibition of Estrogen Receptor and the HER2 Pathway in Breast Cancer: Effects of HER2 Abundance. Translational oncology. 2011;4(5):293-300.
38. Giuliano M, Hu H, Wang YC, Fu X, Nardone A, Herrera S, et al. Upregulation of ER Signaling as an Adaptive Mechanism of Cell Survival in HER2-Positive Breast Tumors Treated with Anti-HER2 Therapy. Clin Cancer Res. 2015;21(17):3995-4003.
39. Chu I, Blackwell K, Chen S, Slingerland J. The dual ErbB1/ErbB2 inhibitor, lapatinib (GW572016), cooperates with tamoxifen to inhibit both cell proliferation- and estrogen-dependent gene expression in antiestrogen-resistant breast cancer. Cancer research. 2005;65(1):18-25.
40. Morrison G, Fu X, Shea M, Nanda S, Giuliano M, Wang T, et al. Therapeutic potential of the dual EGFR/HER2 inhibitor AZD8931 in circumventing endocrine resistance. Breast cancer research and treatment. 2014;144(2):263-72.
41. Burstein HJ, Cirrincione CT, Barry WT, Chew HK, Tolaney SM, Lake DE, et al. Endocrine therapy with or without inhibition of epidermal growth factor receptor and human epidermal growth factor receptor 2: a randomized, double-blind, placebo-controlled phase III trial of fulvestrant with or without lapatinib for postmenopausal women with hormone receptor-positive advanced breast cancer-CALGB 40302 (Alliance). J Clin Oncol. 2014;32(35):3959-66.
42. Cancer Genome Atlas Network. Comprehensive molecular portraits of human breast tumours. Nature. 2012;490(7418):61-70.
43. Carpten JD, Faber AL, Horn C, Donoho GP, Briggs SL, Robbins CM, et al. A transforming mutation in the pleckstrin homology domain of AKT1 in cancer. Nature. 2007;448(7152):439-44.
44. Banerji U, Ranson M, Schellens JH, Esaki T, Dean E, Zivi A, et al. Results of two Phase 1 multicenter trials of AZD5363, an inhibitor of AKT1, 2 and 3: biomarker and early clinical evaluation in Western and Japanese patients with advanced solid tumors. . AACR 2013 annual meeting. 2013;(Presentation LB-66).
45. Davies BR, Greenwood H, Dudley P, Crafter C, Yu D-H, Zhang J, et al. Preclinical Pharmacology of AZD5363, an Inhibitor of AKT: Pharmacodynamics, Antitumor Activity, and Correlation of Monotherapy Activity with Genetic Background. Molecular Cancer Therapeutics. 2012;11(4):873-87.
46. Lin J, Sampath D, Nannini MA, Lee BB, Degtyarev M, Oeh J, et al. Targeting Activated Akt with GDC-0068, a Novel Selective Akt Inhibitor That Is Efficacious in Multiple Tumor Models. Clinical Cancer Research. 2013;19(7):1760-72.
47. Cheung LWT, Hennessy BT, Li J, Yu S, Myers AP, Djordjevic B, et al. High Frequency of PIK3R1 and PIK3R2 Mutations in Endometrial Cancer Elucidates a Novel Mechanism for Regulation of PTEN Protein Stability. Cancer Discovery. 2011;1(2):170-85.
48. Tamura K, Hashimoto J, Tanabe Y, Kodaira M, Yonemori K, Seto T, et al. Safety and tolerability of AZD5363 in Japanese patients with advanced solid tumors. Cancer Chemother Pharmacol. 2016;77(4):787-95.
49. Farmer H, McCabe N, Lord CJ, Tutt AN, Johnson DA, Richardson TB, et al. Targeting the DNA repair defect in BRCA mutant cells as a therapeutic strategy. Nature. 2005;434(7035):917-21.
50. Graeser M, McCarthy A, Lord CJ, Savage K, Hills M, Salter J, et al. A marker of homologous recombination predicts pathologic complete response to neoadjuvant chemotherapy in primary breast cancer. Clin Cancer Res. 2010;16(24):6159-68.

51. Peasland A, Wang LZ, Rowling E, Kyle S, Chen T, Hopkins A, et al. Identification and evaluation of a potent novel ATR inhibitor, NU6027, in breast and ovarian cancer cell lines. British journal of cancer. 2011;105(3):372-81.
52. Yap TA, Krebs MG, Postel-Vinay S, Bang YJ, El-Khoueiry A, Abida W, et al. Phase I modular study of AZD6738, a novel oral, potent and selective ataxia telangiectasia Rad3-related (ATR) inhibitor in combination (combo) with carboplatin, olaparib or durvalumab in patients (pts) with advanced cancers. European Journal of Cancer. 2016;69:S2 Abstr. 1LBA. Presented at the EORTC–NCI–AACR Symposium on Molecular Targets and Cancer Therapeutics, November 2016.
53. Olaparib (AZD2281) Investigator's Brochure Edn 14, 10 March 2017.

APPENDIX 1: GLOSSARY

AE	Adverse Event
ACE	Angiotensin Converting Enzyme
ALT	Alanine Aminotransferase
AML	Acute Myeloid Leukaemia
ANC	Absolute Neutrophil Count
AST	Aspartate Aminotransferase
ATM	Ataxia Telangiectasia Mutated
AUC	Area Under Curve
BID	Twice Daily
CI	Chief Investigator
CI	Confidence Interval
CNS	Central Nervous System
CR	Complete Response
CRC	Clinical Research Committee
CTU	Clinical Trials Unit
ctDNA	Circulating Tumour DNA
ECG	Electrocardiogram
ECHO	Echocardiogram
ECOG	Eastern Cooperative Oncology Group
eCRF	Electronic Case Report Form
EGFR	Epidermal Growth Factor Receptor
EMA	European Medicines Agency
ER	Estrogen Receptor
FSH	Follicle Stimulating Hormone
G-CSF	Granulocyte Colony-Stimulating Factor
GGT	Gamma-glutamyl Transferase
Hb	Haemoglobin
HbA1c	Glycosylated Haemoglobin
HDL	High-Density Lipoprotein
HDPE	High-Density Polyethylene
HR	Homologous Recombination
ICF	Informed Consent Form
ICR	The Institute of Cancer Research
ICR-CTSU	ICR Clinical Trials and Statistics Unit
IDMC	Independent Data Monitoring Committee
IM	Intramuscular
IMP	Investigational Medicinal Product
LD	Longest Diameters
LDL	Low-Density Lipoprotein
LHRH	Luteinizing Hormone-Releasing Hormone
LMWH	Low Molecular Weight Heparin
LVEF	Left Ventricular Ejection Fraction
MBC	Metastatic Breast Cancer
MDS	Myelodysplastic Syndromes

MDT	Multi-Disciplinary Team
MHRA	Medicines and Healthcare Products Regulatory Agency
NCI CTCAE	National Cancer Institute Common Terminology Criteria for Adverse Events
NCRAS	National Cancer Registration and Analysis Service
NE	Not Evaluable
NHS CRS	NHS Care Records Service
NIHR CRN	National Institute for Health Research Clinical Research Network
OD	Once Daily
PARP	Polyadenosine 5’diphosphoribose Polymerase
PD	Progressive Disease
PFS	Progression Free Survival
PgR	Progesterone Receptor
PI	Principal Investigator
PIS	Patient Information Sheet
PK	Pharmacokinetic
PR	Partial Response
R&D	Research and Development
REC	Research Ethics Committee
SAE	Serious Adverse Event
SAR	Serious Adverse Reaction
SERD	Selective Estrogen Receptor Degrader
SD	Stable Disease
SmPC	Summary of Product Characteristics
SRC	Safety Review Committee
SUSAR	Suspected Unexpected Serious Adverse Reaction
TID	Three Times Daily
TMG	Trial Management Group
TNBC	Triple Negative Breast Cancer
TSC	Trial Steering Committee
ULN	Upper Limit of Normal

APPENDIX 2: SAMPLE COLLECTION AND TRANSLATIONAL RESEARCH

All plasmaMATCH trial samples should be collected, processed, stored and shipped as detailed in the plasmaMATCH Investigator Laboratory Manual.

With the exception of the blood samples collected for ctDNA screening, the plasmaMATCH trial samples (for example the archival tumour biopsies and plasma samples taken during trial treatment) will be analysed retrospectively and the results will not be made available to local sites.

BLOOD SAMPLES FOR ctDNA SCREENING

Blood samples for ctDNA screening will be collected as described in Section 7.1.

ON-TREATMENT BLOOD SAMPLES

Please refer to the plasmaMATCH Investigator Laboratory Manual for details on the handling, labelling, storage and shipment of on-treatment blood samples.

Plasma for ctDNA

20ml blood samples should be taken in EDTA tubes on Cycle 1 Days 1 and 15, Cycle 2 onwards on Day 1, and at the Treatment Discontinuation Visit. Blood should be spun at site within 1 hour of collection, plasma aliquoted and frozen at -80ºC.

Samples will be used to extract ctDNA for mutation analysis by digital PCR and/or sequencing to examine for changes in mutation abundance as a surrogate of tumour response, and to examine mechanisms of resistance.

Biomarker bloods

10ml blood samples should be taken in serum tubes on Cycle 1 Days 1 and 15 and Cycle 2 Day 1 for analysis of markers of drug efficacy.

Whole blood for germline DNA

A 3ml blood sample should be taken in EDTA tubes at Cycle 1 Day 1 for DNA extraction for analysis of germline genetic variation using PCR and/or sequencing, as a control for tumour sequencing subtraction and to examine germline genetic variation that may contribute to treatment efficacy or toxicity.

Pharmacokinetic bloods

Cohort A: Blood samples for pharmacokinetic (PK) analysis in Cohort A should be collected prior to treatment on Cycle 2 Day 1, Cycle 3 Day 1 and Cycle 4 Day 1. Fulvestrant drug levels will be assayed and compared to historical controls.

Cohort B: Blood samples for PK analysis in Cohort B should be collected prior to treatment on Cycle 1 Day 1, Cycle 2 Day 1, Cycle 3 Day 1 and Cycle 4 Day 1.

TUMOUR SAMPLES

Please refer to the plasmaMATCH Investigator Laboratory Manual for details on the handling, labelling, storage and shipment of tumour samples.

Primary tumour tissue

Archival primary tissue should be provided for each patient entered into a treatment cohort. If an archival primary tumour sample is not available but another previously obtained sample is available, this should be provided instead.

Baseline tumour biopsy

Patients will be required to provide a baseline recurrent tumour biopsy unless this is deemed unsafe by the PI, in which case an archival biopsy of recurrent disease should be provided instead. If it is deemed unsafe to proceed with a baseline biopsy and no archival recurrent disease biopsy is available the patient will not be eligible for entry into a treatment cohort. For patients who provide a baseline biopsy, the sample should be collected prior to trial treatment and an archival biopsy of recurrent disease should also be provided if available.

Recurrent tumour core biopsies can be ultrasound assisted or CT scan guided dependent on metastatic site and according to local practice. A total of four research cores should be obtained if possible. Two core biopsies should be taken with a 14G core biopsy needle for formalin fixation and standard paraffin embedding, and two core biopsies should be fresh frozen. For formalin fixation, the biopsy sample should be placed in a 20ml universal container containing 10% neutral buffered formalin and sent for embedding in paraffin to the local Histopathology Department.

Archival tumour samples should preferably be in the form of a Formalin-Fixed Paraffin-Embedded (FFPE) blocks. If this is not possible, slides may be provided – please refer to the plasmaMATCH Investigator Laboratory Manual for details.

Tumour biopsies will be analysed at the central laboratory. DNA extracted from archival or fresh tumour material will be analysed for mutations, and comparison will be made with results of ctDNA analysis.

Optional on-treatment and end of treatment biopsies

Patients whose tumours are easily and safely amenable to biopsy should be asked to provide consent for collection of the optional on-treatment recurrent tumour biopsies. These samples should be collected on Cycle 1 Day 15 +/- 2 days and/or at the End of Treatment visit 30 days after the last dose of trial treatment.

Recurrent tumour core biopsies can be ultrasound assisted or CT scan guided dependent on metastatic site and according to local practice. A total of four research cores should be obtained if possible. Two core biopsies should be taken with a 14G core biopsy needle for formalin fixation and standard paraffin embedding, and two core biopsies should be fresh frozen. For formalin fixation biopsy sample should be placed in a 20ml universal container containing 10% neutral buffered formalin and sent for embedding in paraffin to the local Histopathology Department.

Tumour biopsies will be analysed at the central laboratory. DNA extracted from archival or fresh tumour material will be analysed for mutations, and comparison will be made with results of ctDNA analysis.

TRANSLATIONAL RESEARCH

Nucleic acid extractions and analyses

Nucleic acids including DNA and RNA will be extracted from tissue samples, and DNA will be extracted from normal buffy coat and plasma. DNA samples will be subjected to digital PCR or sequencing analysis or other molecular techniques to identify mutations relevant to cancer biology.

Protein analysis

Tissue sections from the trial will be analysed by immunohistochemistry, or immunofluorescence, or other techniques for analysis of proteins. Tissue samples may be processed for analysis of proteins using alternative techniques.

Assessment of intra-tumoural heterogeneity and clonality

Exploratory assessment will be made on whether a targetable mutation is apparently clonal (likely present in all cancer cells in the tumour) or sub-clonal (present in only a subset of cancer cells in the body). The criteria to define clonal and sub-clonal will be detailed in the plasmaMATCH Investigator Laboratory Manual. Evidence of sub-clonality may include discordance in the mutation between ctDNA screening blood sample and tissue biopsy, or evidence that the mutation has reduced allele frequency compared to other mutations present in the ctDNA.

APPENDIX 3: RESPONSE EVALUATION CRITERIA IN SOLID TUMOURS (RECIST) VERSION 1.1

Response Evaluation Criteria in Solid Tumours (RECIST) Version 1.1 should be used for the assessment of treatment outcomes. A summary is given below but Investigators should always refer to the published guidelines.

EVALUATION OF MEASURABLE AND NON-MEASURABLE LESIONS

- **Measurable disease** – the presence of at least one measurable lesion. If the measurable disease is restricted to a solitary lesion, its neoplastic nature should be confirmed by cytology/histology.
- **Measurable lesions** – lesions that can be accurately measured in at least one dimension with the longest diameter ≥20mm by chest X-ray, or ≥10mm by CT/MRI scan or clinical exam.
- Lytic bone lesions or mixed lytic-blastic lesions, with identifiable soft tissue components, that can be evaluated by cross sectional imaging techniques such as CT or MRI can be considered as measurable lesions if the soft tissue component meets the definition of measurability described above.
- 'Cystic lesions' thought to represent cystic metastases can be considered as measurable lesions, if they meet the definition of measurability described above. However, if non-cystic lesions are present in the same patient, these are preferred for selection as target lesions.
- Malignant lymph nodes must be ≥15mm in the short axis when assessed by CT scan to be considered measurable.
- **Non-measurable lesions** – all other lesions, including small lesions and malignant lymph nodes (longest diameter <10mm, or pathological lymph nodes with ≥10 to <15mm short axis) i.e. leptomeningeal disease, ascites, pleural/pericardial effusion, inflammatory breast disease, lymphangitis cutis/pulmonis, cystic lesions, blastic bone lesions and also abdominal masses that are not confirmed and followed by imaging techniques.
- Tumour lesions situated in a previously irradiated area, or in an area subjected to other loco-regional therapy, are usually not considered measurable unless there has been demonstrated progression in the lesion.
- The utilisation of endoscopy and laparoscopy for objective tumour evaluation is not advised. The utilisation of such techniques should be restricted to confirming complete pathological response when biopsies are obtained.

BASELINE DOCUMENTATION OF TARGET AND NON-TARGET LESIONS

- Measurable lesions up to a maximum of 2 lesions per organ and 5 lesions in total, representative of all involved organs, should be identified as target lesions and recorded and measured at baseline.
- Target lesions should be selected on the basis of their size (lesions with the longest diameter), be representative of all involved organs, but in addition should be those that lend themselves to reproducible repeated measurements. It may be the case that, on occasion, the largest lesion does not lend itself to reproducible measurement in which circumstance the next largest lesion which can be measured reproducibly should be selected.
- All measurements should be taken and recorded in metric notation, using a ruler or calipers. All screening evaluations should be performed as closely as possible to the beginning of treatment and never more than 4 weeks before the beginning of treatment.

- The same method of assessment and the same technique should be used to characterise each identified and reported lesion at baseline and during follow up.
- A sum of the longest diameters (LD) for all target lesions will be calculated and reported as the baseline sum of LD. The baseline sum LD will be used as reference by which to characterise the objective tumour.
- All other lesions (or sites of disease) should be identified as non-target lesions and should also be recorded at baseline. Measurements of these lesions are not required, but the presence or absence of each should be noted throughout follow up. In addition, it is possible to record multiple non-target lesions involving the same organ as a single item on the case record form (e.g. 'multiple enlarged pelvic lymph nodes' or 'multiple liver metastases').

RESPONSE CRITERIA

Documentation of new lesions

- The presence of a new lesion should be unequivocal: i.e. not attributable to differences in scanning technique, change in imaging modality or findings thought to represent something other than tumour (for example, some 'new' bone lesions may be simply healing or flare of pre-existing lesions).
- A lesion identified at a follow-up visit in an anatomical location that was not scanned at baseline is considered a new lesion and will indicate disease progression.

Lesions that become 'too small to measure'

- If lesions or lymph nodes recorded as target lesions at baseline become too faint on CT scan to assign an exact measure, a default value of 5mm should be assigned. This default value is derived from the 5mm CT slice thickness.
- If it is the opinion of the radiologist that the lesion has likely disappeared, the measurement should be recorded as 0mm.

Evaluation of target lesions

Appendix 3, Table 1. RECIST v1.1 evaluation of target lesions

Response criteria	Evaluation of target lesions
Complete response (CR)	Disappearance of all target lesions (lymph nodes must be <10mm short axis)
Partial response (PR)	At least 30% decrease in the sum of LD of target lesions, taking as reference the baseline sum of LD
Progressive disease (PD)	At least 20% increase in the sum of LD of target lesions, taking as reference the smallest sum LD recorded since the treatment started and at least 5mm absolute increase in this sum or the appearance of one or more new lesions
Stable disease (SD)	Neither sufficient shrinkage to qualify for PR nor sufficient increase to qualify for PD, taking as reference the smallest sum LD since the treatment started

Evaluation of non-target lesions

Appendix 3, Table 2. RECIST v1.1 evaluation of non-target lesions

Response criteria	Evaluation of non-target lesions
Complete response (CR)	Disappearance of all non-target lesions
Incomplete response/ stable disease (SD)	Persistence of one or more non-target lesions
Progressive disease (PD)	Appearance of one or more new lesions and/or unequivocal progression of existing non-target lesions*

*To achieve 'unequivocal progression' on the basis of the non-target disease, there must be an overall level of substantial worsening in non-target disease such that, even in presence of SD or PR in target disease, the overall tumour burden has increased sufficiently to merit discontinuation of therapy. A modest 'increase' in the size of one or more non-target lesions is usually not sufficient to quality for unequivocal progression status. Although a clear progression of a non-target lesion is exceptional, in such circumstances, the opinion of the treating physician should prevail and the progression status should be confirmed later on by the review panel (or CI).

Evaluation of overall response

- The table below provides a summary of the overall response calculation at each time point.

Appendix 3, Table 3. RECIST v1.1 evaluation of overall response

Target lesions	Non-target lesions	New lesions	Overall response
CR	CR	No	CR
CR	Incomplete response/SD	No	PR
CR	Not-evaluated*	No	PR
PR	Non-PD or not all evaluated	No	PR
SD	Non-PD or not all evaluated	No	SD
Not all evaluated	Non-PD	No	Not evaluable
PD	Any	Yes or No	PD
Any	PD	Yes or No	PD
Any	Any	Yes	PD

*When no imaging/measurement is done at all at a particular time point, the patient is not evaluable (NE) at that time point. If only a subset of lesion measurements are made at an assessment, usually the case is also considered NE at that time point, unless a convincing argument can be made that the contribution of the individual missing lesion(s) would not change the assigned time point response.

Confirmation of disease progression

For equivocal findings of progression (e.g. very small and uncertain new lesions; cystic changes or necrosis in existing lesions), treatment may continue until the next scheduled assessment. If at the next scheduled assessment, progression is confirmed, the date of progression should be the earlier date when progression was suspected.

Patients with a global deterioration of health status requiring discontinuation of treatment without objective evidence of disease progression at that time should be classified as having "symptomatic deterioration". Every effort should be made to document the objective progression even after discontinuation of treatment.

In some circumstances it may be difficult to distinguish residual disease from normal tissue. When the evaluation of CR depends on this determination, it is recommended that the residual lesion be investigated (fine needle aspirate/biopsy) to confirm the CR status.

Duration of overall response

The duration of overall response is measured from the time measurement criteria are met for CR or PR (whichever status is recorded first) until the first date that recurrence or PD is objectively documented, taking as reference for PD the smallest measurements recorded since the treatment started.

Duration of stable disease

SD is measured from the start of the treatment until the criteria for disease progression are met, taking as reference the smallest measurements recorded since the treatment started.

Evaluation of best overall response

The best overall response is the best response recorded from the start of the trial treatment until disease progression. The patient's best overall response assignment will depend on the findings of both target and non-target disease and the appearance of new lesions.

Best overall response is defined as the best response across all time points. For example, a patient who has SD at first assessment, PR at second assessment, and PD on last assessment has a best overall response of PR.

Central review

Blinded central review of objective responses will be conducted in order to protect against any reporting bias. CT and/or MRI scans will be collected for all patients at baseline and throughout the duration of trial treatment until disease progression.

APPENDIX 4: ECOG PERFORMANCE STATUS

Appendix 4, Table 1. ECOG performance status

Score	Activity performance description
0	Fully active, able to carry out all normal activity without restriction
1	Restricted in physically strenuous activity but ambulatory and able to carry out work of a light or sedentary nature, for example, light housework, office work
2	Ambulatory and capable of all self-care but unable to carry out any work activities; up and about more than 50% of waking hours
3	Capable of only limited self-care; confined to bed or chair more than 50% of waking hours
4	Completely disabled; cannot carry on any self-care; totally confined to bed or chair

APPENDIX 5: CREATININE CLEARANCE CALCULATION

COCKCROFT & GAULT CALCULATION

Women

$$\text{Estimated creatinine clearance} = \frac{(140\text{-age [years]}) \times \text{weight (kg)}}{\text{serum creatinine (mg/dL)} \times 72} \times 0.85$$

Please note, sites should calculate Cockcroft & Gault formula as per routine practice.

APPENDIX 6: COHORT B – POTENTIAL FOR DRUG–DRUG INTERACTIONS

Appendix 6, Table 1. Cohort B – Drugs associated with risk of QT/QTc prolongation leading to Torsade de pointes

Drugs Reported to Prolong QT Interval		
Analgesics		
Celecoxib (Celebrex)	Methadone (e.g. Dolophine, Methadose)	
Anesthetic agents		
Enflurane (e.g. Ethrane)	Halothane	
Isoflurane (e.g. Forane)		
Antiarrhythmic agents		
Class IA	Class III	
Disopyramide (e.g. Norpace)*	Amiodarone (e.g. Cordarone)*[b]	
Procainamide (e.g. Procanbid)*	Bretylium*	
Quinidine*	Dofetilide (Tikosyn)*[b]	
Class IC	Ibutilide (Corvert)*[b]	
Flecainide (e.g. Tambocor)*[a]	Sotalol (e.g. Betapace)*[b]	
Propafenone (e.g. Rythmol)*[b]		
Anticonvulsants		
Felbamate (Felbatol)*	Fosphenytoin (Cerebyx)	
Antiemetics		
Dolasetron (Anzemet)[b]	Droperidol (e.g. Inapsine)*[b]	Ondansetron (Zofran)
Antihistamines		
Desloratadine (Clarinex)[b] (overdose)	Fexofenadine (Allegra)	
Diphenhydramine (e.g. Benadryl)	Hydroxyzine (Atarax)	
Anti-infectives		
Amantadine (e.g. Symmetrel)*	Macrolides and related antibiotics	
Antimalarials	Azithromycin (e.g. Zithromax)	
Mefloquine (e.g. Lariam)[b]	Clarithromycin (e.g. Biaxin)*[b]	
Quinine*	Erythromycin (e.g. Ery-Tab, EES)*[b]	
Antivirals	Telithromycin (Ketek)[b]	
Efavirenz (Sustiva)*	Troleandomycin	
Azole antifungal agents	Pentamidine (e.g. Pentam 300, Nebupent)*	
Fluconazole (e.g. Diflucan)*[b]	Quinolones	
Itraconazole (e.g. Sporanox)	Gatifloxacin (e.g. Tequin)*[b]	
Ketoconazole (e.g. Nizoral)	Levofloxacin (e.g. Levaquin)*[a, b]	
Voriconazole (Vfend)[b]	Moxifloxacin (e.g. Avelox)[b]	
Chloroquine (e.g. Aralen)*	Ofloxacin (e.g. Floxin)*[b]	
Clindamycin (e.g. Cleocin)	Sparfloxacin (Zagam)[b]	
Foscarnet (Foscavir)	Trimethoprim/sulfamethoxazole (e.g. Bactrim)*	
Antineoplastics		
Arsenic trioxide (Trixenox)*[b]	Doxorubicin (e.g. Adriamycin)	Tamoxifen (e.g. Nolvadex)
Bronchodilators		
Albuterol (e.g. Proventil)[b]	Salmeterol (Serevent)[b]	
Formoterol (Foradil)[b]	Terbutaline (e.g. Brethine)[b]	
Isoproterenol (e.g. Isuprel)		

Continued overleaf

Appendix 6, Table 1 continued. Cohort B – Drugs associated with risk of QT/QTc prolongation leading to Torsade de pointes

Drugs Reported to Prolong QT Interval		
Calcium channel blockers		
Isradipine (DynaCirc)	Nicardipine (e.g. Cardene)	
Contrast media		
Ionic contrast media*	Non-ionic contrast media: Iohexol (Omnipaque)	
Corticosteroids		
Prednisolone (e.g. Prelone)	Prednisone (e.g. Deltasone)*	
Diuretics		
Furosemide (e.g. Lasix)	Indapamide (e.g. Lozol)	
Gastrointestinal agents		
Cisapride (Propulsid)*[b]	Famotidine (e.g. Pepcid)*	
Immunosuppressants		
Tacrolimus (Protopic)*[b] (postmarketing)		
Miscellaneous		
Levomethadyl	Papaverine (e.g. Pavaden three times daily [TID])*	
Moexipril/Hydrochlorothiazide (Uniretic)	Probucol (Lorelco)*	
Octreotide (Sandostatin)[b]	Vasopressin (e.g. Pitressin)*	
Oxytocin (e.g. Pitocin; intravenous bolus)		
Psychotropics		
Droperidol (e.g. Inapsine)*	Primozide (Orap)*[b]	Trazodone (e.g. Desyrel)
Haloperidol (e.g. Haldol)*	Quetiapine (Seroquel)[b]	Tricyclic antidepressants
Lithium (e.g. Eskalith)*	Risperidone (Risperdal)[b] (overdose)	Amitriptyline*
Maprotiline*	Serotonin Reuptake Inhibitors (SRIs)	Clomipramine (e.g. Anafranil)
Phenothiazines	Citalopram (e.g. Celexa)*	Desipramine (e.g. Norpramin)*
Chrlorpromazine (e.g. Thorazine)*	Fluoxetine (e.g. Prozac)*[a]	Doxepin (e.g. Sinequan)*
Fluphenazine (e.g. Prolixin)*	Paroxetine (e.g. Paxil)*	Imipramine (e.g. Tofranil)*
Perphenazine	Sertraline (Zoloft)*[a, b] (postmarketing)	Nortriptyline (e.g. Pamelor)
Thioridazine (Mellaril)*[b]	Venlafaxine (Effexor)[b] (postmarketing)	
Trifluopherazine		
Serotonin 5-HT^{1} agonists		
Naratriptan (Amerge)	Sumatriptan (Imitrex)[b]	Zolmitriptan (Zomig)[b]
Skeletal muscle relaxants		
Tizanidine (e.g. Zanaflex)[b] (animals)		

*Drugs for which Torsades de Pointes has also been reported

[a] Association unclear

[b] QT, QTc and/or Torsades de Pointes association listed in FDA-approved product labeling

Source: Tatro DS, Drug Interaction Facts. The Authority on Drug Interactions. Wolters Kluwer Health 2012.

Appendix 6, Table 2. Cohort B – Substrates and inhibitors of P-glycoprotein (P-gp)

**P-glycoprotein Substrates**		
Amiodarone (e.g. Cordarone)	Fluphenazine (e.g. Prolixin)	Progesterone (e.g. Prometrium)
Chlorpromazine (e.g. Thorazine)	Hydrocortisone (e.g. Cortef)	Promethazine (e.g. Phenergan)
Clarithromycin (e.g. Biaxin)	Indinavir (Crixivan	Quinidine
Cyclosporine (e.g. Neoral)	Itraconazole (e.g. Sporanox)	Reserpine
Dactinomycin (Cosmegen)	Ketoconazole (e.g. Nizoral)	Ritonavir (Norvir)
Daunorubicin (e.g. Cerubidine)	Lidocaine (e.g. Xylocaine)	Saquinavir (e.g. Fortovase)
Dexamethasone (e.g. Decadron)	Loperamide (e.g. Imodium)	Sirolimus (Rapamune)
Digoxin (e.g. Lanoxin)	Lovastatin (e.g. Mevacor)	Tacrolimus (Prograf)
Diltiazem (e.g. Cardizem)	Mifepristone (Mifeprex)	Tamoxifen (e.g. Nolvadex)
Doxorubicin (e.g. Adriamycin)	Mitoxantrone (Novantrone)	Teniposide (Vumon)
Erythromycin (e.g. Ery-Tab)	Nelfinavir (Viracept)	Testosterone Delatestryl)
Estradiol (e.g. Estrace)	Nicardapine (e.g. Cardene)	Trifluoperazine
Etoposide (e.g. Vepesid)	Nifedipine (e.g. Procardia)	Verapamil (e.g. Calan)
Felodipine (Plendil)	Ondansetron (Zofran)	Vinblastine (e.g. Velban)
Fexofenadine (Allegra)	Paclitaxel (e.g. Taxol)	Vincristine (e.g. Vincasar PFS)
**P-glycoprotein Inhibitors**		
Amiodarone (e.g. Cordarone)	Indinavir (Crixivan)	Quinidine
Atorvastatin (Lipitor)	Itraconazole (e.g. Sporanox)	Reserpine
Chlorpromazine (e.g. Thorazine)	Ketoconazole (e.g. Nizoral)	Ritonavir (Norvir)
Clarithromycin (e.g. Biaxin)	Lidocaine (e.g. Xylocaine)	Saquinavir (e.g. Fortovase)
Cyclosporine (e.g. Neoral)	Mifepristone (Mifeprex)	Tacrolimus (Prograf)
Diltiazem (e.g. Cardizem)	Nelfinavir (Viracept)	Tamoxifen (e.g. Nolvadex)
Erythromycin (e.g. Ery-Tab)	Nicardipine (e.g. Cardene)	Testosterone (Delatestryl)
Felodipine (Plendil)	Nifedipine (e.g. Procardia)	Trifluoperazine
Fluphenazine (e.g. Prolixin)	Progesterone (e.g. Prometrium)	Verapamil (e.g. Calan)
Hydrocortisone (e.g. Cortef)	Propranolol (e.g. Inderal)	

Source: Tatro DS, Drug Interaction Facts: The Authority on Drug Interactions. Wolters Kluwer Health 2012.

Appendix 6, Table 3. Cohort B – Inhibitors and inducers of the cytochrome P450 isoenzymes

CYP3A4 Inducers		
Carbamazepine	Macrolide antibiotics	Rifabutin
Efavirenz	Phenobarbital	Rifampin
Glucocorticoids:	Phenylbutazone	Rifapentine
Dexamethasone	Phenytoin	St. John's Wort
Prednisone	Primidone	Sulfinpyrazone
**CYP3A4 Inhibitors**		
Amprenavir	Grapefruit juice	Paroxetine
Anastrozole	Indinavir	Propranolol
Cimetidine	Itraconazole	Quinidine
Clarithromycin	Ketoconazole	Quinine
Clotrimazole	Mibefradil	Ranitidine
Danazol	Miconazole	Ritonavir
Delavirdine	Mirtazapine (weak)	Saquinavir
Diethyldithiocarbamate	Nefazodone	Sertraline
Diltiazem	Nelfinavir	Sildenafil (weak)
Erythromycin	Nevirapine	Troglitazone
Fluconazole	Norfloxacin	Troleandomycin
Fluoxetine	Norfluoxetine	Zafirlukast
Fluvoxamine		
**CYP3A5-7 Inducers**		
Phenobarbital	Primidone	Rifampin
Phenytoin		

Source: Tatro DS, Drug Interaction Facts: The Authority on Drug Interactions. Wolters Kluwer Health 2012.

APPENDIX 7: COHORTS C AND D – AZD5363 CONCOMITANT TREATMENT CAUTIONS AND RESTRICTIONS

DRUGS AFFECTING CYP3A4 METABOLISM THAT ASTRAZENECA STRONGLY RECOMMEND ARE NOT COMBINED WITH AZD5363

There are currently no data confirming that there are any PK interactions between any agents and AZD5363. The potential interactions detailed below are considered on the basis of the preclinical data only. The following lists are not intended to be exhaustive, and a similar restriction will apply to other agents that are known to strongly modulate CYP3A4 activity. Appropriate medical judgment is required. Please contact ICR-CTSU with any queries on this issue.

Appendix 7, Table 1. Cohorts C and D – Strong CYP3A4 inhibitors that may increase exposure to AZD5363 more than 5-fold

Drug	Guidance
Ketoconazole<br>Protease inhibitors (danoprevir, ritonavir, saquinavir, indanavir, tapranavir, telaprevir, elvitegravir, lopinavir, nelfinavir, bocepravir)<br>Cobicistat<br>Conivaptan<br>Nefazodone<br>Mebefradil	Minimum of 2 weeks washout prior to AZD5363 administration and for 2 weeks following discontinuation of AZD5363.
Itraconazole<br>Posaconazole<br>Voriconazole<br>Clarithromycin<br>Telithromycin<br>Troleandomycin	Minimum of 1 week washout prior to AZD5363 administration and for 2 weeks following discontinuation of AZD5363.

Appendix 7, Table 2. Cohorts C and D – Potent Inducers of CYP3A4 that may reduce exposure to AZD5363 by more than 5-fold

Drug	Guidance
Phenobarbital<br>Carbamazepine<br>Phenytoin<br>Rifampicin<br>Rifabutin<br>Mitotane<br>Enzalutamide	Minimum of 2 weeks washout prior to AZD5363 administration and for 2 weeks following discontinuation of AZD5363.
St John’s Wort	Minimum of 3 weeks washout prior to AZD5363 administration and for 2 weeks following discontinuation of AZD5363.

DRUGS AFFECTING CYP3A4 METABOLISM THAT ASTRAZENECA CONSIDERS MAY BE ALLOWED WITH CAUTION

Appendix 7, Table 3. Cohorts C and D – Moderate Inhibitors of CYP3A4 that may increase exposure to AZD5363

Drug	Guidance
Diltiazem<br>Verapamil<br>Erythromycin<br>Fluconazole<br>Aprepitant	Drugs are permitted but caution should be exercised and patients monitored closely for possible drug interactions. Please refer to full prescribing information for all drugs prior to co-administration with AZD5363.
Grapefruit juice<br>Seville oranges (and other products containing Seville oranges)	Patients should abstain from eating large amounts of grapefruit and Seville oranges (and other products containing these fruits e.g. grapefruit juice or marmalade) during the trial (e.g. no more than a small glass of grapefruit juice (120ml) or half a grapefruit or 1–2 teaspoons (15g) of Seville orange marmalade daily).

MEDICINES THAT ARE SIGNIFICANTLY METABOLISED BY CYP3A4 THAT ASTRAZENECA STRONGLY RECOMMEND ARE NOT COMBINED WITH AZD5363

There are currently no data confirming that there are any PK interactions between AZD5363 and the following CYP3A4 substrates. The potential interactions detailed below are considered on the basis of the preclinical data only. The following list is not intended to be exhaustive, and a similar restriction will apply to other agents that are known to be sensitive to CYP3A4 inhibitors. Appropriate medical judgment is required. Please contact ICR-CTSU with any queries on this issue.

Appendix 7, Table 4. Cohorts C and D – Exposure, pharmacological action and toxicity that may be increased by inhibition of CYP3A4 by AZD5363

Drug	Guidance
Alfentanil<br>Cyclosporin<br>Diergotamine<br>Ergotamine<br>Fentanyl<br>Sirolimus<br>Tacrolimus<br>Atorvastatin<br>Lovastatin<br>Simvastatin<br>Cerivastatin	Minimum of 1 week washout prior to AZD5363 administration and for 2 weeks following discontinuation of AZD5363.
Carbamazepine	Minimum of 2 weeks washout prior to AZD5363 administration and for 2 weeks following discontinuation of AZD5363.

MEDICINES THAT ARE SIGNIFICANTLY METABOLISED BY CYP3A4 THAT ASTRAZENECA CONSIDERS MAY BE ALLOWED WITH CAUTION

Appendix 7, Table 5. Cohorts C and D – Exposure, pharmacological action and toxicity that may be increased by inhibition of CYP3A4 by AZD5363

Drug	Guidance
Erythromycin<br>Trazodone<br>Tamoxifen<br>Alprazolam<br>Midazolam<br>Triazolam<br>Felodipine<br>Isradipine<br>Nifedipine<br>Methylprednisolone<br>Pimozide<br>Quinidine<br>Domperidone	Drugs are permitted but caution should be exercised and patients monitored closely for possible drug interactions. Please refer to full prescribing information for all drugs prior to co-administration with AZD5363.

AGENTS THAT ARE SENSITIVE TO CYP2D6 INHIBITION THAT ASTRAZENECA STRONGLY RECOMMEND ARE NOT COMBINED WITH AZD5363

There are currently no data confirming that there are any PK interactions between AZD5363 and the following CYP2D6 substrates. The potential interactions detailed below are considered on the basis of the preclinical data only. The following list is not intended to be exhaustive, and a similar restriction will apply to other agents that are known to be sensitive to CYP2D6 inhibitors. Appropriate medical judgment is required. Please contact ICR-CTSU with any queries on this issue.

Appendix 7, Table 6. Cohorts C and D – Exposure, pharmacological action and toxicity that may be increased by inhibition of CYP2D6 by AZD5363

Drug	Guidance
Amitryptyline<br>Desipramine<br>Trimipramine<br>Doxepin	Minimum of 2 weeks washout prior to AZD5363 administration and for 2 weeks following discontinuation of AZD5363.
Atomoxetine<br>Metoprolol<br>Nefazodone<br>Nebivolol<br>Perphenazine<br>Tropisetron<br>Tolterodine	Minimum of 1 week washout prior to AZD5363 administration and for 2 weeks following discontinuation of AZD5363.

AGENTS THAT ARE SENSITIVE TO CYP2D6 INHIBITION THAT ASTRAZENECA CONSIDERS MAY BE ALLOWED WITH CAUTION

Appendix 7, Table 7. Cohorts C and D – Exposure, pharmacological action and toxicity that may be increased by inhibition of CYP2D6 by AZD5363

Drug	Guidance
Venlafaxine<br>Paroxetine<br>Fluoxetine	Drugs are permitted but caution should be exercised and patients monitored closely for possible drug interactions. Please refer to full prescribing information for all drugs prior to co-administration with AZD5363.

AGENTS THAT ARE SENSITIVE TO COMBINED CYP3A4 AND CYP2D6 INHIBITION THAT ASTRAZENECA STRONGLY RECOMMEND ARE NOT COMBINED WITH AZD5363

There are currently no data confirming that there is a PK interaction between AZD5363 and the following agents; a potential interaction is considered on the basis of the preclinical data only. This list is not intended to be exhaustive, and a similar restriction will apply to other agents with narrow therapeutic windows that are known to depend on combined CYP3A4 and CYP2D6 metabolism. Appropriate medical judgment is required. Please contact ICR-CTSU with any queries on this issue.

Appendix 7, Table 8. Cohorts C and D – Exposure, pharmacological action and toxicity that may be increased by inhibition of CYP3A4 and CYP2D6 by AZD5363

Drug	Guidance
Haloperidol	Minimum of 2 weeks washout prior to AZD5363 administration and for 2 weeks following discontinuation of AZD5363.
Tramadol	Minimum of 1 week washout prior to AZD5363 administration and for 2 weeks following discontinuation of AZD5363.

GUIDANCE FOR DRUGS THAT ARE THAT ARE SIGNIFICANTLY METABOLISED BY CYP2B6, CYP2C9 OR CYP2C19 AND HAVE A NARROW THERAPEUTIC MARGIN THAT ASTRAZENECA CONSIDERS MAY BE ALLOWED WITH CAUTION

Weak signals for competitive inhibition of CYP2B6, CYP2C9 and CYP2C19 cytochrome P450 activities have been demonstrated by *in vitro* laboratory investigations. There are currently no data confirming that there is a PK interaction between AZD5363 and substrates of these isoforms; a potential interaction is considered on the basis of the preclinical data only. The following list is intended to identify known sensitive substrates of CYP2B6, CYP2C9 and CYP2C19 that have a narrow therapeutic margin. The list is not intended to be exhaustive, and a similar restriction should be applied to any other sensitive substrate with narrow therapeutic margin. Appropriate medical judgment is required. Please contact ICR-CTSU with any queries on this issue.

Appendix 7, Table 9. Cohorts C and D – Exposure, pharmacological action and toxicity that may be increased by inhibition of CYP2B6, CYP2C9 and CYP2C19 by AZD5363

Drug	Guidance
• CYP2B6<br>Bupropion<br>• CYP2C9<br>Warfarin<br>• CYP2C19<br>Clobazam	Drugs are permitted but caution should be exercised and patients monitored closely for possible drug interactions. Please refer to full prescribing information for all drugs prior to co-administration with AZD5363.

GUIDANCE FOR STATINS THAT ARE METABOLISED BY CYP3A4 THAT ASTRAZENECA CONSIDERS MAY BE ALLOWED WITH CAUTION

Time-dependent inhibition of cytochrome P450 (CYP) 3A4/5 was observed during the non-clinical *in vitro* evaluation of the metabolism of AZD5363.

The CYP3A4 isozyme is responsible for the metabolism of atorvastatin (ATV), cerivastatin (CRV), lovastatin (LOV), and simvastatin (SMV), including combinations with ezetimibe (SMV/ezetimibe [SMV/EZE]), and their exposure, pharmacological action and toxicity may increase by inhibition of CYP3A4 and the potential for CYP-mediated Drug-Drug interactions (DDIs) is high.

However, there is minimal metabolism of fluvastatin (FLV), pravastatin (PRV), or rosuvastatin (RSV) by CYP3A4 thus plasma levels are minimally influenced by CYP3A4 inhibitors, conveying a relatively low potential for clinically significant DDIs via this mechanism.

Emerging *in vitro* data has revealed that AZD5363 has a potential to inhibit the OATP1B1 transporter. This transporter is implicated in the distribution and clearance of many of the statins. Of the statins that are minimally affected by CYP3A4 inhibition, RSV and PRV (but not FLV) can be affected by OATP1B1 inhibition. Based on an assessment of the potential for AZD5363 to inhibit OATP1B1 based on the *in vitro* signal the AUC of these drugs may be increased by 1.3-fold for PRV and 1.5-fold for RSV (static assessment based on maximal free liver inlet concentration of AZD5363). As a conservative response to this emerging data it is recommended that doses of RSV be capped to 10mg once daily and PRV be capped to 40mg once daily when combined with AZD5363, and for a 2 week period before and after AZD5363 treatment.

In summary, RSV (up to 10mg once daily), PRV (up to 40mg once daily) and FLV are appropriate agents, to be used in patients included in AZD5363 studies who require statin therapy.

REFERENCES

Michalets EL. Update: clinically significant cytochrome P-450 drug interactions. Pharmacother. 1998;18(1):84-112. http://medicine.iupui.edu/clinpharm/ddis/table.asp. Washington School of Pharmacy Drug Interaction Database resources (online)

APPENDIX 8: COHORT E – OLAPARIB CONCOMITANT TREATMENT CAUTIONS AND RESTRICTIONS

STRONG OR MODERATE CYP3A INHIBITORS

Known strong CYP3A inhibitors (e.g., itraconazole, telithromycin, clarithromycin, boosted protease inhibitors, indinavir, saquinavir, nelfinavir, boceprevir, telaprevir) or moderate CYP3A inhibitors (ciprofloxacin, erythromycin, diltiazem, fluconazole, verapamil) should not be taken with olaparib. If there is no suitable alternative concomitant medication then the dose of olaparib should be reduced for the period of concomitant administration.

Strong CYP3A inhibitors – reduce the dose of olaparib to 100mg bd for the duration of concomitant therapy with the strong inhibitor and for 5 half lives afterwards.

Moderate CYP3A inhibitors – reduce the dose of olaparib to 150mg bd for the duration of concomitant therapy with the moderate inhibitor and for 3 half lives afterwards.

After the washout of the inhibitor is complete, the olaparib dose can be re-escalated.

STRONG OR MODERATE CYP3A INDUCERS

Strong (e.g., phenobarbital, phenytoin, rifampicin, rifabutin, rifapentine, carbamazepine, nevirapine, enzalutamide and St John's Wort) and moderate CYP3A inducers (eg. bosentan, efavirenz, modafinil) of CYP3A should not be taken with olaparib. If the use of any strong or moderate CYP3A inducers are considered necessary for the patient's safety and welfare this could diminish the clinical efficacy of olaparib. If a patient requires use of a strong or moderate CYP3A inducer or inhibitor then they must be monitored carefully for any change in efficacy of olaparib.

P-gp INHIBITORS

It is possible that co-administration of P-gp inhibitors (e.g. amiodarone, azithromycin) may increase exposure to olaparib. Caution should therefore be observed.

EFFECT OF OLAPARIB ON OTHER DRUGS

Based on limited *in vitro* data, olaparib may increase the exposure to substrates of CYP3A4, P-gp, OATP1B1, OCT1, OCT2, OAT3, MATE1 and MATE2K. Based on limited *in vitro* data, olaparib may reduce the exposure to substrates of CYP3A4, CYP1A2, 2B6, 2C9, 2C19 and P-gp.

The efficacy of hormonal contraceptives may be reduced if co administered with olaparib.

Caution should therefore be observed if substrates of these isoenzymes or transporter proteins are co-administered. Examples of substrates include:

- CYP3A4 – hormonal contraceptive, simvastatin, cisapride, cyclosporine, ergot alkaloids, fentanyl, pimozide, sirolimus, tacrolimus and quetiapine
- CYP1A2 – duloxetine, melatonin
- CYP2B6 – bupropion, efavirenz
- CYP2C9 – warfarin
- CYP2C19 – lansoprazole, omeprazole, S-mephenytoin
- P-gp – simvastatin, pravastatin, digoxin, dabigatran, colchicine

- OATP1B1 – bosentan, glibenclamide, repaglinide, statins and valsartan
- OCT1, MATE1, MATE2K – metformin
- OCT2 – serum creatinine
- OAT3 – furosemide, methotrexate

APPENDIX 9: COHORT E – AZD6738 CONCOMITANT TREATMENT CAUTIONS AND RESTRICTIONS

RESTRICTIONS REGARDING DRUGS AFFECTING CYP3A METABOLISM

There are currently no data confirming that there is a pharmacokinetic (PK) interaction between these agents and AZD6738; a potential interaction is considered on the basis of preclinical and *in vitro* data only. AZD6738 is predominantly eliminated via CYP3A metabolism (approximately 60%), therefore CYP3A inhibitors or inducers may increase or decrease exposure to AZD6738, respectively. Potent inhibitors or inducers of CYP3A should not be combined with AZD6738.

These lists are not intended to be exhaustive, and similar restrictions will apply to other agents that are known to modulate CYP3A activity. Please refer to full prescribing information for all drugs prior to co-administration with AZD6738.

Appendix 9, Table 1. Drugs known to be inhibitors and inducers of CYP3A

**Potent CYP3A inhibitors**			
Boceprevir	Clarithromycin	Conivaptan	Elvitegravir/RIT
Fluconazole	Grapefruit juice	Indinavir	Itraconazole
Ketoconazole	Lopinavir/ RIT	Mibefradil	Nefazodone
Nelfinavir	Posaconazole	Ritonavir	Saquinavir
Telaprevir	Telithromycin	Tipranavir/RIT	Troleandomycin
Voriconazole			
**Potent CYP3A inducers**			
Avasimibe	Carbamazepine	Enzalutamide	Mitotane
Phenobarbital	Phenytoin	Rifabutin	Rifampin
St John's Wort			
List created using the University of Washington Drug-Drug Interaction Database January 2013.<br>RIT = Ritonivir. Ritonavir has dual effects of simultaneous CYP3A inhibition and induction, and the net pharmacokinetic outcome during chronic ritonavir therapy is inhibition of CYP3A activity			

DRUGS KNOWN TO BE INHIBITORS OR INDUCERS OF P-gp AND/OR BCRP

Appropriate monitoring should be undertaken if co-administration is necessary. AZD6738 is a substrate of P-gp and BCRP. Co-administration of P-gp inhibitors/inducers or BCRP inhibitors/inducers may affect exposure to AZD6738 therefore it is recommended that these are not co-administered with AZD6738.

These lists are not intended to be exhaustive, and similar restrictions will apply to other agents that are known to modulate P-gp activity or BCRP activity. Please refer to full prescribing information for all drugs prior to co-administration with AZD6738.

Appendix 9, Table 2. Drugs known to be inhibitors or inducers of P-gp

Drugs known to be inhibitors of P-gp[a]		
Amiodarone	Azithromycin	Captopril
Carvedilol	Clarithromycin	Conivaptan
Cremophor	Curcumin	Diltiazem
Dronedarone	Elacridar	Erythromycin
Felodipine	Fluvoxamine	Ginkgo
Indinavir	Itraconazole	Ketoconazole
Lapatinib	Lopinavir and ritonivir	Mibefradil
Milk thistle	Mirabegron	Nelfinavir
Nifedipine	Nitrendipine	Paroxetine
Quercetin	Quinidine	Ranolazine
Rifampin	Ritonavir	Saquinavir/ritonavir
Schisandra chinensis extract	St Johns Wort	Talinolol
Telaprevir	Telmisartan	Ticagrelor
Tipranavir/ritonavir	Tolvaptan	Valspodar (PSC 833)
Verapamil		
**Drugs known to be inducers of P-gp[b]**		
Avasimibe	Carbamazepine	Efavirenz
Genistein	Phenytoin	Rifampin
St John's Wort		
[a] Inhibitors listed for P-gp are those that showed >25% increase in exposure to a P-gp substrate (e.g. digoxin)<br>[b] Inducers listed for P-gp are those that showed >20 % decrease in exposure to a P-gp substrate (e.g. digoxin)		

Appendix 9, Table 3. Drugs known to be inhibitors or inducers of BCRP

Drugs known to be inhibitors of BCRP		
Afatinib	Aripiprazole	Curcumin
Cyclosporine	Elacridar	Erlotinib
Fluvastatin	Fumitremorgin	Gefitinib
Ivermectin	Lapatinib	Nilotinib
Novobiocin	Pantoprazole	Pitavastatin
Ponatinib	Quercetin	Quizartinib
Rabeprazole	Regorafenib	Rilpivirine
Sulfasalazine	Sunitinib	Tacrolimus
Teriflunomide	Trametinib	Trifluoperazine
Vismodegib	eltrombopag	Atazanavir
Lopinavir	Ritonavir	Tipranavir
Omeprazole	Estrone	17b-estradiol
Imatinib mesylate		
**Drugs known to be inducers of BCRP**		
Please check individual drugs on a case by case basis		
List created using http://dmd.aspetjournals.org/content/dmd/43/4/490.full.pdf<br>Note: Although BCRP is involved in a number of clinically relevant DDIs, none of the cited inhibitors above is truly specific for this transporter		

DRUGS KNOWN TO BE SUBSTRATES OF CYP3A4 AND/OR CYP2B6

Appropriate monitoring should be undertaken if co-administration is necessary. AZD6738 is a potential inducer of CYP3A4 and CYP2B6. Therefore caution should be applied with co administration of drugs that are either completely metabolised by CYP3A4 and/or CYP2B6, or that are substrates of CYP3A4 and/or CYP2B6 and also have a narrow therapeutic index. Investigators should be aware that the exposure of other drugs metabolised by CYP3A4 and/or CYP2B6 may be reduced.

Appendix 9, Table 4. Drugs known to be metabolised by CYP3A4 and have a narrow therapeutic index

Drugs known to be metabolised by CYP3A4 and have a narrow therapeutic index		
Alfentanil	Cyclosporine	Dihydroergotamine
Ergotamine	Fentanyl	Pimozide
Quinidine	Sirolimus	Tacrolimus
Astemizole	Cisapride	Terfenadine

Appendix 9, Table 5. Drugs known to be metabolised by CYP2B6 and have a narrow therapeutic index

Drugs known to be metabolised by CYP2B6 and have a narrow therapeutic index		
Cyclophosphoamide	Ifosfamide	Efavirenz
Bupropion	Propofol	Thiotepa
Sorafenib	Alfentanil	Ketamine
Methadone	Methoxetamine	Nevirapine
Propofol	Selegiline	Sertraline
Sorafenib	Tamoxifen	Valproic acid
From Flockhart DA (2007). "Drug Interactions: Cytochrome P450 Drug Interaction Table". Indiana University School of Medicine		

DRUGS KNOWN TO BE SUBSTRATES OF OATP1B1 AND BCRP

Appropriate monitoring should be undertaken if co-administration is necessary. AZD6738 is also an inhibitor of OATP1B1 and BCRP. Caution should be applied with co-administration of substrates of OATP1B1 and/or BCRP as AZD6738 may increase their exposure.

These lists are not intended to be exhaustive and appropriate medical judgment is required. Please contact AstraZeneca with any queries you have on this issue. Please refer to full prescribing information for all drugs prior to co-administration with AZD6738.

Appendix 9, Table 6. Drugs known to be substrates of OATP1B1

Drugs known to be substrates of OATP1B1		
Atorvastatin	Fluvastatin	Lovastatin
Pitavastatin	Pravastatin	Rosuvastatin
Simvastatin	Ezemibe	Simvastatin
Methotrexate	Rifampin	Bosentan
Glyburide	Repaglinide	Valsartan
Olmesartan	Atrasentan	
List created using https://www.solvobiotech.com/transporters/OATP1B1		

Appendix 9, Table 7. Drugs known to be substrates of BCRP

Drugs known to be substrates of BCRP		
Anthracyclines	Daunorubicin	Doxorubicin
Topotecan	SN-38	Irinotecan
Methotrexate	Imatinib	Irinotecan
Mitoxantrone	Nucleoside analogs	Prazosin
Pantoprazole	Topotecan	Rosuvastatin and other statins
Teriflunomide	Chlorothiazide	
List created using https://www.solvobiotech.com/transporters/bcrp		
